# LARP1 post-transcriptionally regulates mTOR and contributes to cancer progression

**DOI:** 10.1038/onc.2014.428

**Published:** 2014-12-22

**Authors:** M Mura, T G Hopkins, T Michael, N Abd-Latip, J Weir, E Aboagye, F Mauri, C Jameson, J Sturge, H Gabra, M Bushell, A E Willis, E Curry, S P Blagden

**Affiliations:** 1 Division of Cancer, Department of Surgery and Cancer, Ovarian Cancer Action Research Centre, Imperial College London, Hammersmith Campus, London, UK; 2 Department of Cellular Pathology, Imperial College Healthcare NHS Trust, Charing Cross Hospital, London, UK; 3 Division of Cancer, Department of Surgery and Cancer, Cancer Research UK Laboratories, Imperial College London, Hammersmith Campus, London, UK; 4 Department of Histopathology, Centre for Pathology, Imperial College London, Hammersmith Campus, London, UK; 5 Department of Histopathology, University College Hospital, London, UK; 6 School of Biological, Biomedical & Environmental Sciences, The Allam Building, University of Hull, Hull, UK; 7 MRC Toxicology Unit, Hodgkin Building, University of Leicester, Leicester, UK

## Abstract

RNA-binding proteins (RBPs) bind to and post-transcriptionally regulate the stability of mRNAs. La-related protein 1 (LARP1) is a conserved RBP that interacts with poly-A-binding protein and is known to regulate 5′-terminal oligopyrimidine tract (TOP) mRNA translation. Here, we show that LARP1 is complexed to 3000 mRNAs enriched for cancer pathways. A prominent member of the LARP1 interactome is mTOR whose mRNA transcript is stabilized by LARP1. At a functional level, we show that LARP1 promotes cell migration, invasion, anchorage-independent growth and *in vivo* tumorigenesis. Furthermore, we show that LARP1 expression is elevated in epithelial cancers such as cervical and non-small cell lung cancers, where its expression correlates with disease progression and adverse prognosis, respectively. We therefore conclude that, through the post-transcriptional regulation of genes such as mTOR within cancer pathways, LARP1 contributes to cancer progression.

## Introduction

RNA-binding proteins (RBPs) regulate the decay kinetics and translational efficiency of mRNA transcripts by accelerating their degradation or prolonging their cytoplasmic half-life.^1^ In this way, the abundance of mRNAs and their encoded proteins can be altered in a manner that is independent from gene transcription. As RBPs are themselves activated by growth factors and cell signals, this tightly regulated post-transcriptional mechanism enables the cell to rapidly adjust levels of protein expression in response to intrinsic and extracellular signals.^2, 3^ In addition, RBPs can interact with up to thousands of mRNA transcripts, allowing the coordinated synthesis of multiple proteins involved in a single physiological function (termed an RNA operon).^4^ However, when the expression of an RBP is disrupted, it can potentially disrupt cellular homeostasis and autonomously drive pathological processes by uncoupling the regulation of mRNA stability from cell signaling cues.^5^ A protein recently identified as being an RBP is La-related protein 1 (LARP1).^6^ LARP1 belongs to the LARP family, three members of which: LARP1, LARP3 (or genuine La protein) and LARP7 have so far been implicated in cancer. An elevated expression of LARP1 has been shown to correlate with clinical outcome in hepatocellular carcinoma,^7^ LARP3 protein expression is increased in cervical cancer and higher levels have been shown to correlate with adverse outcome in lung cancer.^8, 9^ In contrast, LARP7 is a potential tumor suppressor in gastric and cervical tumors.^10, 11^

LARP1 was first identified in *Drosophila melanogaster*, where it was shown to bind poly-A-binding protein (PABP) and was required for embryonic development and fertility.^12^ Proteomic screens conducted in human embryonic cell lines have subsequently shown that LARP1 contributes to the stability and translation of 5′-terminal oligopyrimidine tract (TOP) mRNAs by simultaneously interacting with their 5′-cap components and their 3′-untranslated regions (UTRs).^13, 14^ TOP mRNAs are required for ribosome biogenesis and are regulated downstream of the mammalian (or mechanistic) target of rapamycin (mTOR) complex 1 (mTORC1) kinase. The recent discovery that LARP1 is itself regulated downstream of mTORC1 signaling has placed it as a key player within the mTOR pathway and as a regulator of cellular growth and proliferation.^14, 15, 16^ As perturbation of mTOR signaling is a common occurrence in cancer, we chose two tumor types: squamous cervical and non-small cell lung cancers, in which mTOR pathway signaling is frequently disrupted, to explore the relationship between LARP1 and mTOR.^17, 18, 19^ We demonstrate that, rather than solely acting downstream of mTOR, LARP1 also post-transcriptionally regulates mTOR by binding and stabilizing its encoding mRNA. Moreover, we show that the regulatory activity of LARP1 is not just confined to mTOR, but that LARP1 is complexed to many other mRNA transcripts involved in cancer-related functions. We demonstrate that LARP1 expression correlates with clinical outcome in cervical and non-small cell lung cancers and, at functional level, regulates cell migration, invasion, anchorage-independent and *in vivo* tumor growth.

## Results

### LARP1 expression is increased in epithelial cancers and correlates with clinical variables

**Figure 1:**
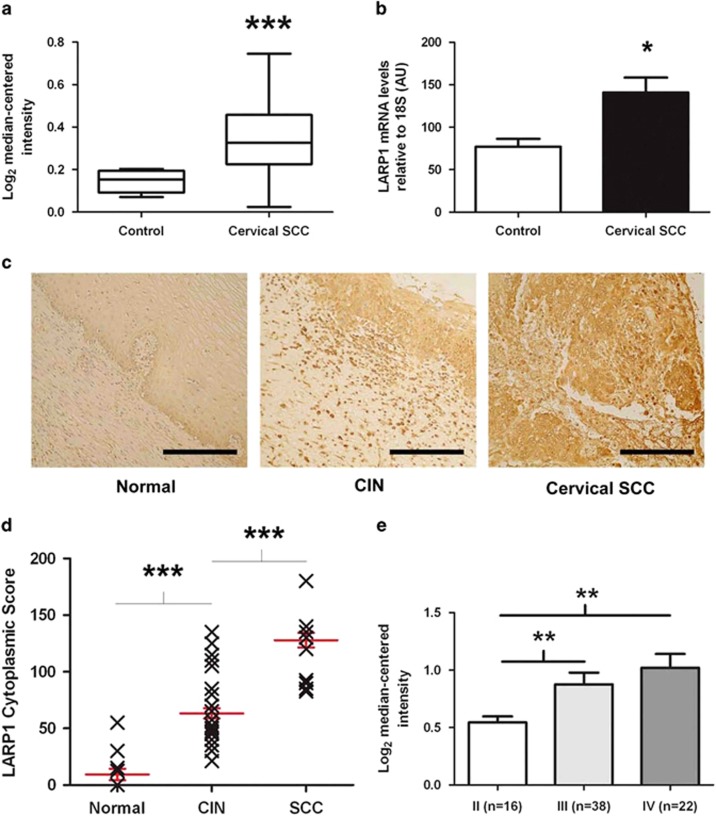
LARP1 correlates with clinical variables in cervical cancer. (**a**) Expression dataset comparing LARP1-mRNA levels in cervical squamous cell carcinoma (SCC) and non-cancer tissue. (**b**) Real-time PCR on RNA extracted from non-cancer cervical tissue (*n*=4) and cervical SCC (*n*=7). LARP1-mRNA levels relative to 18S ribosomal RNA (*P*=0.012, Student's *t*-test). (**c**) LARP1 immunostaining (brown) of normal cervical epithelium (N12), cervical intraepithelial neoplasia (CIN; N35) and invasive cervical SCC (N36), counterstained with haematoxylin. (**d**) LARP1 cytoplasmic scores for CIN compared to normal samples (*P*<0.0001) and in invasive cervical SCC compared to CIN samples (*P*<0.0001). (**e**) Expression dataset showing LARP1 expression in cervical SCC, stratified according to stage. *P⩽0.05, **P⩽ 0.001 and ***P⩽0.0001.

We examined the expression of LARP1 across multiple cancers using a systematic review of datasets in the Oncomine repository (available at http://www.oncomine.org).^20^ LARP1-mRNA expression was increased across almost all epithelial malignancies (Supplementary Figure 1a and Supplementary Table 1). Of these, we chose to focus predominantly on cervical (SCC) as a model system. Cervical SCC not only is associated with aberrant mTOR^19^ activation, but also has a clearly defined progression from pre-malignant cervical intraepithelial neoplasia to established cancer, characterized by increasing invasiveness. Searching Oncomine for expression data comparing cancer and non-cancer samples revealed a single study (*n*=45) where tissue from normal cervix was compared to cervical cancer.^10^ This demonstrated significantly higher LARP1-mRNA levels in cervical (SCC; Figure 1a). To validate this, we obtained total RNA from surgical samples of human cervical SCC and non-cancer cervical tissue (Imperial College Tissue Bank, London, UK). Real-time PCR demonstrated that LARP1-mRNA expression was significantly increased in cervical SCC (Figure 1b).

Using immunohistochemistry, we quantified LARP1 expression in a tissue microarray comprising tissue cores (*n*=83) from normal cervical epithelium, cervical intraepithelial neoplasia and invasive cervical SCC. We found significant increased levels of LARP1 in cervical intraepithelial neoplasia versus normal epithelium and cervical SCC versus cervical intraepithelial neoplasia, confirming that cytoplasmic LARP1 significantly correlated with progression of cervical cancer (Figures 1c and d). In support of this finding, publicly available expression data (*n*=76) demonstrated that LARP1 levels significantly increased with increasing stage of invasive cancer^21^ (Figure 1e).

To validate our findings, we assessed LARP1 levels in a second epithelial cancer type in which mTOR is known to be dysregulated^17, 18^ with a similarly poor clinical outcome. Consistent with our findings in cervical SCC, using pooled survival analysis of expression array data for 1405 non-small cell lung cancer patients we observed that high LARP1-mRNA expression significantly correlated with adverse prognosis (Supplementary Figure 1b).^22^

### The LARP1 interactome is enriched for multiple pathways involved in cancer

**Figure 2:**
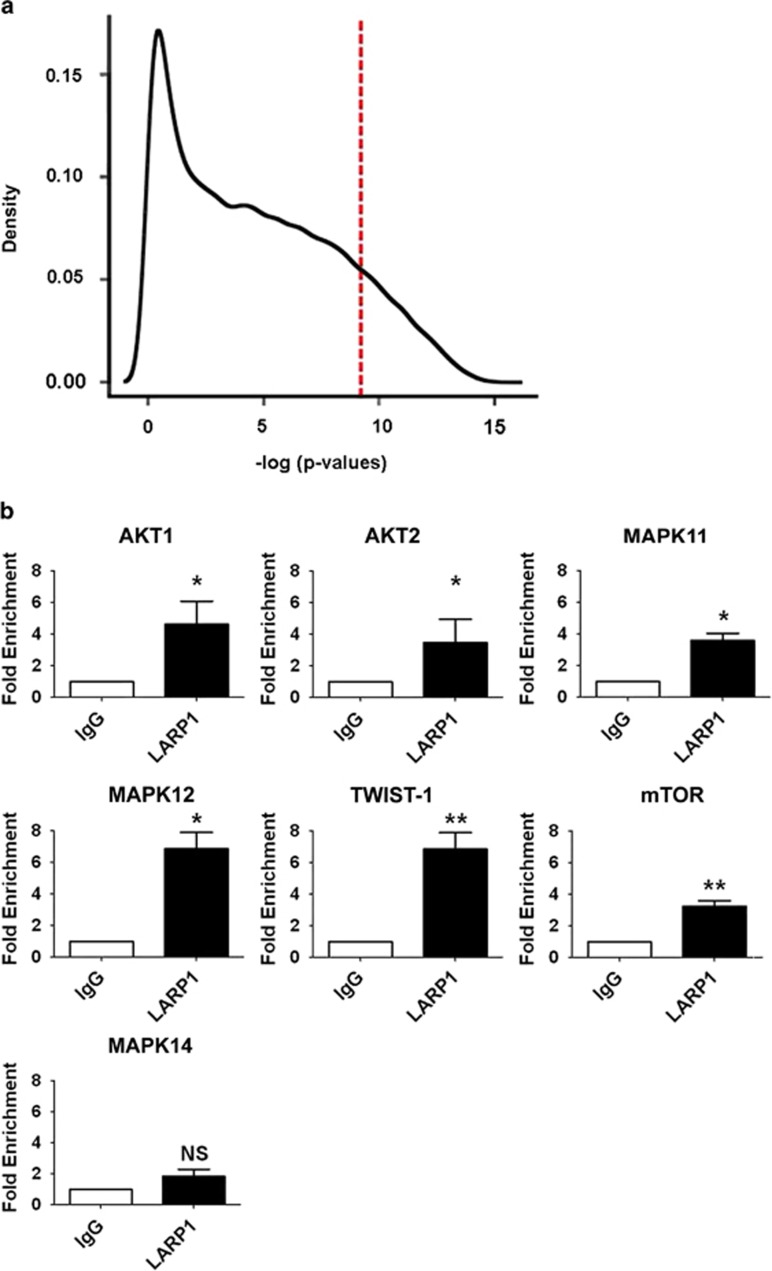
LARP1 exists in complex with mRNAs. (**a**) Histogram demonstrating relative transcript density across significance thresholds for LARP1-IP-enriched mRNAs (red line, *P*=1 × 10^−4^). (**b**) RIP-Chip validation by qRT–PCR showing fold enrichment of selected target mRNAs immunoprecipitated with LARP1 antibody, normalized to immunoprecipitation with IgG isotype control. Experiments were performed ⩾3 times. Data are mean±s.e.m. *P⩽0.05 and **P⩽0.001.

To further explore the association with LARP1 expression and outcome we then wished to identify the mRNAs that were in complex with LARP1. We performed RNA immunoprecipitation and microarray profiling (RIP-Chip) using anti-LARP1 antibody in HeLa cells (Supplementary Figure 2a). This approach has been shown to enrich for stable and functionally significant interactions.^23^ A robust and reproducible list of LARP1-mRNA associations was obtained from four individual experiments, analyzed in duplicate, with the highest density of transcript interaction within a significant threshold (*P*=1 × 10^−4^; Figure 2a). We named these the ‘LARP1 interactome' (Supplementary Table 2). Functional enrichment analysis of transcripts revealed associations with cancer-related pathways. As well as those controlling extracellular matrix receptor interactions, regulation of the actin cytoskeleton, focal adhesion, PI3K, MAPK, VEGF signaling, we discovered members of the mTOR signaling cascade within the LARP1 interactome (Supplementary Table 3).

To validate these findings, we selected seven mRNA targets from within the LARP1 interactome based on their *P*-values: AKT1, AKT2 and MAPK11 were highly ranked, while MAPK12, mTOR and Twist-1 were middle to bottom ranked. As a negative control, we selected MAPK14 which was absent from the interactome. Real-time PCR (RT–PCR), performed using RNA immunoprecipitated with LARP1 antibody versus IgG isotype control, confirmed that all interactome mRNAs including mTOR were LARP1 bound (Figure 2b).

### LARP1 affects mTOR transcript stability

**Figure 3:**
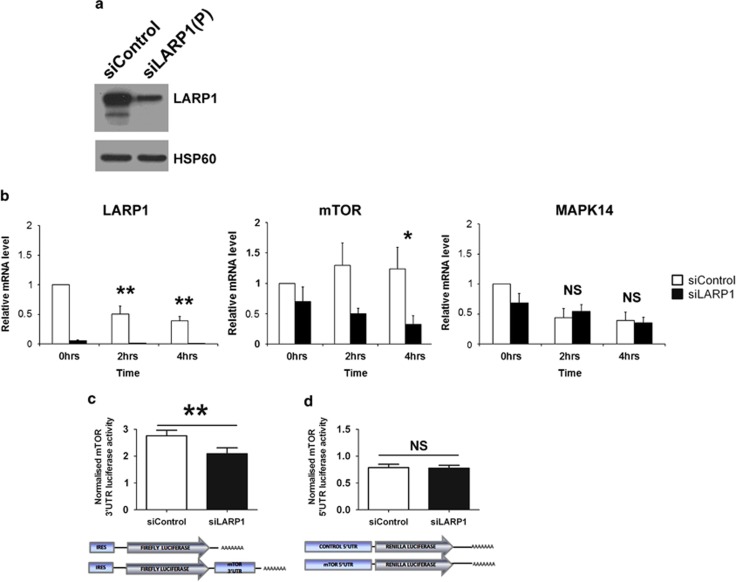
LARP1 regulate the stability of mTOR mRNA. (**a**) Western blot showing LARP1 knockdown with pooled siRNA. HSP60 was used as loading control. (**b**) Real-time PCR following transcription inhibition by actinomycin-D treatment in HeLa cells transfected with two pooled LARP1 siRNA or control siRNA. Fold changes relative to 18S, normalized to untreated control. (**c**) Luciferase production in HeLa cells transfected with firefly-luciferase reporter construct containing mTOR 3′UTR sequence normalized to the relative control plasmid (bottom panel: representation of the constructs used) in HeLa cells previously transfected with pooled siRNA to LARP1 or non-targeting siRNA. (**d**) Luciferase production in HeLa cells transfected with a renilla-luciferase reporter construct containing mTOR 5′UTR sequence normalized to the relative control plasmid (bottom panel: representation of constructs used) in HeLa cells previously transfected with pooled siRNA to LARP1 or non-targeting siRNA. Experiments were performed ⩾3 times. Data are mean±s.e.m. *P⩽0.05 and **P⩽0.001.

To further investigate our finding of mTOR within the interactome, we explored the effect of LARP1 on mTOR transcript stability by quantitating mRNA levels after drug inhibition of *de novo* RNA transcription. Forty-eight hours after LARP1 knockdown with two pooled siRNA sequences (Figure 3a) cells were treated with actinomycin-D. Total RNA was extracted and relative expression quantified by RT–qPCR. Two hours following drug treatment, LARP1-mRNA levels in control siRNA-transfected samples decreased by 50%, while in cells transfected with LARP1 siRNA the total mRNA level was constantly decreased by >80%. Interestingly, mTOR mRNA appeared to be stable and did not decrease during the time-course treatment with actinomycin-D, an effect that has been observed previously^24, 25^ (Figure 3b). In contrast, LARP1-depleted cells treated with actinomycin-D for 4 h displayed a significant decrease in mTOR mRNA (Figure 3b). We then evaluated the effect of LARP1 on candidates that interact with high efficiency, such as MAPK12 and TWIST1. No significant difference was observed during the time course of the experiment (0–4 h; Supplementary Figures S3a and b), indicating that LARP1 has a neutral impact on the stability of some of its interactome targets. mRNA levels of MAPK14, which was absent from the LARP1 interactome, decreased by 50% following treatment with actinomycin-D, but no significant difference between LARP1-depleted and control cells was observed during the time course of the experiment (0–4 h; Figure 3b). These findings indicate that LARP1 is required to maintain the stability of mTOR transcripts.

UTRs of mRNAs carry elements responsible for their stability.^26^ To investigate the effect of LARP1 on mTOR transcript stability, we analyzed the effect of LARP1 knockdown on mTOR 3′UTR luciferase-reporter construct and relative control. After LARP1 depletion, a significant decrease (24.3%) in firefly-luciferase activity was observed for the mTOR 3′UTR construct compared to non-targeting siRNA control (Figure 3c). We then investigated the effect of LARP1 knockdown on mTOR 5′UTR using a construct containing the mTOR 5′UTR sequence and relative control. In contrast to the 3′UTR experiment, no significant difference was observed in the 5′-renilla-luciferase activity between LARP1 knockdown and non-targeting control (Figure 3d). This suggests that LARP1 effect on the stability of mTOR transcripts is controlled via elements contained within its 3′UTR.

### Protein expression of LARP1-interactome targets is LARP1 dependent

**Figure 4:**
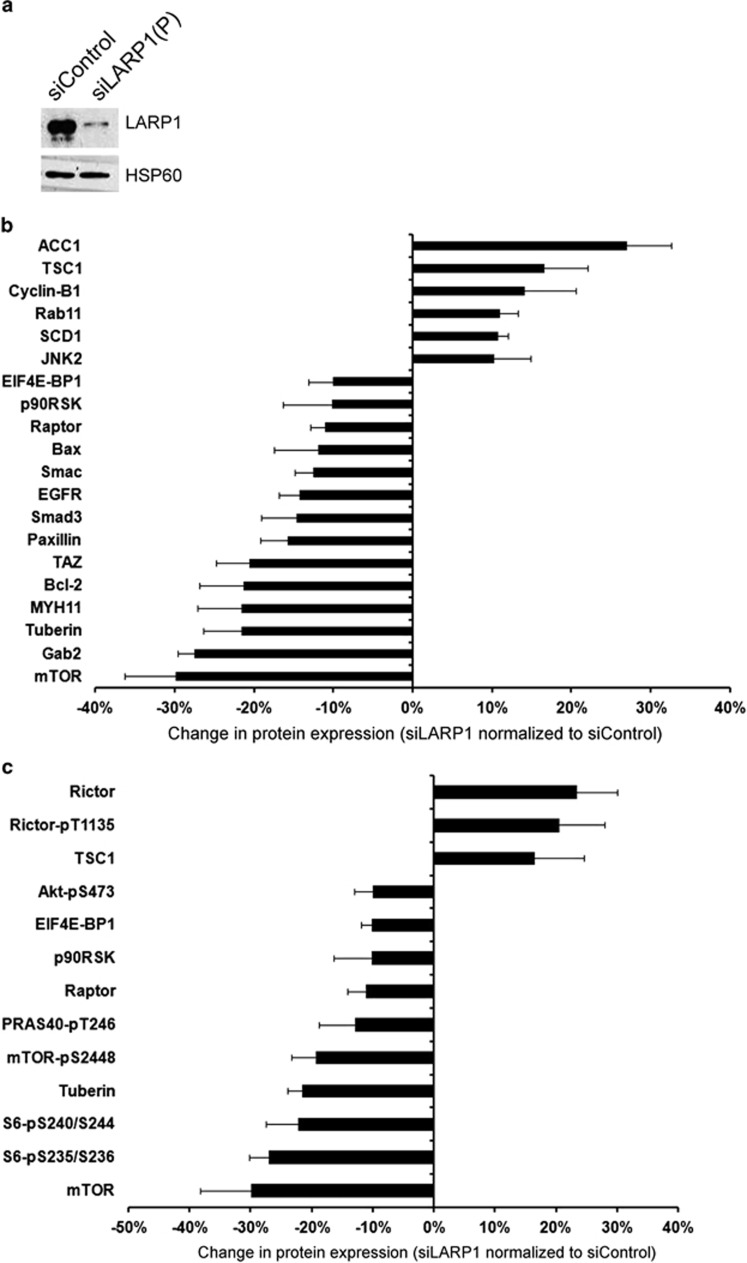
Depletion of LARP1 alters protein expression of interactome components, with wider effects on mTOR signaling pathway. (**a**) Level of LARP1 in HeLa cells transfected with control siRNA and two independent pooled siRNA sequences (siLARP1-1 and siLARP1-2). HSP60 was used as loading control. (**b**) Reverse-phase protein array after LARP1 knockdown (data normalized to control siRNA) shows altered expression of protein products of corresponding mRNAs identified as existing in complex with LARP1. (**c**) Change in protein expression and phosphorylation of mTOR signaling pathway components after LARP1 knockdown (data normalized to control siRNA). Experiments were performed ⩾3 times. Data are mean±s.e.m.

Having demonstrated that LARP1 has an effect on the stability of mTOR mRNA, we wished to quantitate the effect of LARP1 knockdown (Figure 3a) on the expression of mTOR protein and other protein components of the mTOR signaling cascade. To do this, we used a standardized, high-throughput and validated reverse-phase protein array, representing 138 proteins and 44 phosphoproteins found to be dysregulated in cancer. Of the 45 protein targets on the reverse-phase protein array identified as part of the full LARP1 interactome 44% were altered after LARP1 knockdown (Figure 4b). Corresponding with its effect on mTOR mRNA, we confirmed that knockdown of LARP1 resulted in reduced mTOR protein expression as well as reduced expression of a number of other proteins and phosphoproteins within the mTOR cascade, such as P-mTOR-S2448, RAPTOR, p-RPS6-S235/236, pRPS6-S240/244 and p90RSK (Figure 4c). Tuberin (TSC2) a negative regulator of mTORC1/2 decreased on LARP1 knockdown, although TCS1 showed the opposite trend, perhaps altering the stability of the TSC1/TSC2 complex. Of interest, RICTOR, a component of the mTORC2, was increased after LARP1 knockdown.

**Figure 5:**
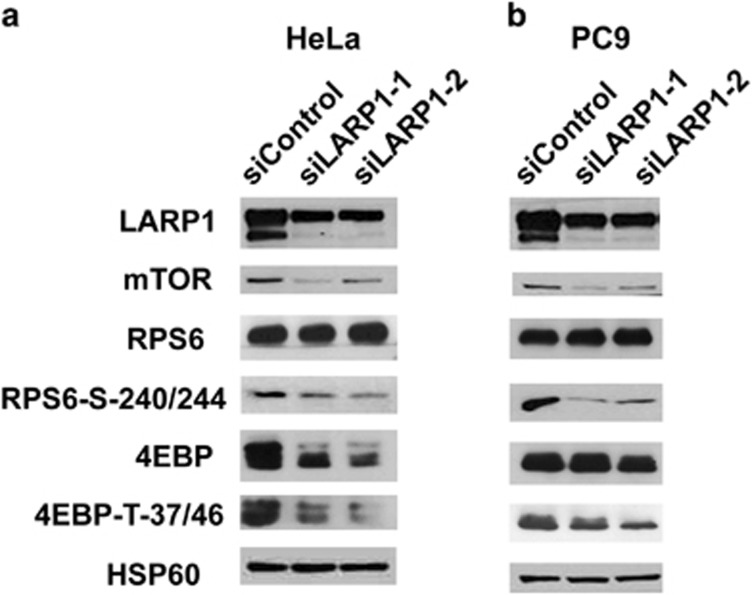
LARP1 knockdown decreases mTOR protein level and its downstream targets P-RPS6 and P-4EBP in HeLa and PC9 cell lines. Western blotting analysis of LARP1 in HeLa (**a**) and PC9 (**b**) cell lines after LARP1 knockdown using two independent siRNA oligos showing decrease in expression of mTOR, P-4EBP and P-RPS6, but not total RPS6 ribosomal protein. Total 4EBP showed a moderate decrease in HeLa cells. HSP60 was used as a loading control.

To further validate the role of LARP1 in regulating mTOR, we performed and quantified western blots of mTOR protein, and downstream components of the mTORC1 signaling cascade in HeLa and PC9 cell lines (Figures 5a and b and Supplementary Figures S4a and b). In both lines, knockdown of LARP1 with two independent siRNAs resulted in reduction of mTOR protein, phosphorylated RPS6-S240/244 and 4E-BP1-T37/46. In contrast, LARP1 knockdown did not alter levels of total RPS6 or total 4E-BP1, the latter showing a moderate decrease in HeLa cells. Our findings confirm that, by positively regulating mTOR transcript stability, LARP1 affects the expression of a number of downstream mTOR signaling pathway components.

### LARP1 regulates components distinct from the mTOR pathway

By reverse-phase protein array, we identified additional targets whose protein expression was altered by LARP1 knockdown beyond the known interactome targets (Supplementary Figures 5a and b). As LARP1 exists in complex with several transcripts encoding transcription factors, kinases and regulatory proteins, this indirect effect was not unexpected, and supports a general pro-oncogenic role for LARP1. Our protein screen demonstrated that LARP1 positively regulates anti-apoptotic factors (B Cell lymphoma 2 (BCL2) and BCL2-associated X protein (BAX)), growth factor receptors/adapters (epidermal growth factor receptor (EGFR), vascular endothelial growth factor receptor (VEGFR) and GRB2-associated binding protein (GAB)), focal adhesion (paxillin), epithelial-mesenchymal transition EMT effectors (β-catenin) and transcription factors (Y box binding protein 1 (YB1), tafazzin (TAZ) and small body size/mothers against decapentaplegic 3 (SMAD3)). These data suggest that the pro-oncogenic phenotype driven by LARP1 can be caused by the simultaneous aberration of multiple signaling pathways. Proteins whose expressions are unchanged after LARP1 knockdown are summarized in Supplementary Table S4.

### LARP1 promotes cell migration and invasion

**Figure 6:**
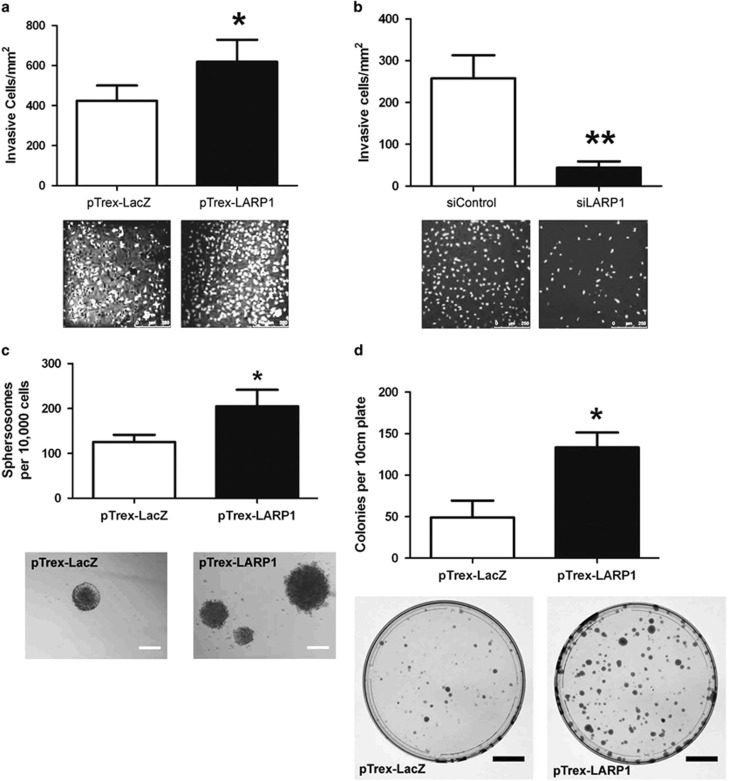
LARP1 promotes invasion and anchorage-independent growth. (**a**) Invasion assay in HeLa cells stably transfected with pTrex-LARP1 and pTrex-LacZ cells or (**b**) with pooled siRNA to LARP1 and non-targeting control. Graphs are counts of the number of invasive cells per cm^2^. Representative images of DAPI-stained invasive cells are shown (scale bars, 250 μm). (**c**) Anchorage-independent growth assay using ultra-low attachment plates in HeLa cells stably transfected with pTrex-LARP1 and pTrex-LacZ. Representative images of spherosomes (scale bars, 200 μm). (**d**) Number of colonies generated by dissociated spherosomes plated in adherent conditions. Scale bars, 2 cm. Experiments were performed ⩾3 times; data are mean±s.e.m. *P⩽0.05 and **P⩽0.001.

As the mTOR pathway is known to be a key effector of invasion and migration we wished to explore the functional effect of altered LARP1 expression on these cellular processes.^27^ First, we performed a scratch repair assay using HeLa and PC9 cells transfected with either full-length LARP1 (pTrex-LARP1) or a control vector (pTrex-LacZ; Supplementary Figures 6a and 7a). Overexpression of LARP1 significantly enhanced cell motility, with LARP1-overexpressing HeLa and PC9 cells migrating 37 and 40% faster, respectively, than controls (Supplementary Figures S6b and 7b). We then showed, using Transwell Matrigel invasion assays (BD Biosciences, Franklin Lakes, NJ, USA), that LARP1 overexpression significantly enhanced the invasive capabilities of HeLa cells (Figure 6a). A more pronounced effect was seen after LARP1 knockdown, with an 85% reduction in cell invasion (Figure 6b). Invasion assays performed in PC9 cells after LARP1 knockdown also showed a significant decrease in the number of invasive cells (Supplementary Figure S7c). These results indicate that the disruption of LARP1 expression significantly alters cancer cell invasion. There was no significant difference in viability or proliferative ability detected between *in vitro* cell populations, either for overexpression or knockdown in either PC9 (Supplementary Figures 7d and e) or HeLa cells during the time course of the experiments (Supplementary Figures S8a–c).

### LARP1 enhances spherosome formation

To investigate the effect of LARP1 on cell survival in anchorage-independent conditions, we then cultured LARP1-overexpressing cells in ultra-low attachment plates. Under these conditions, single cells form floating colonies termed spherosomes. LARP1 overexpression significantly increased both the total number of spherosomes formed (Figure 6c) and the number of viable colonies identified when spherosomes were dissociated to a single-cell suspension and re-plated under adherent conditions (Figure 6d).

### LARP1 promotes tumor growth *in vivo*

**Figure 7:**
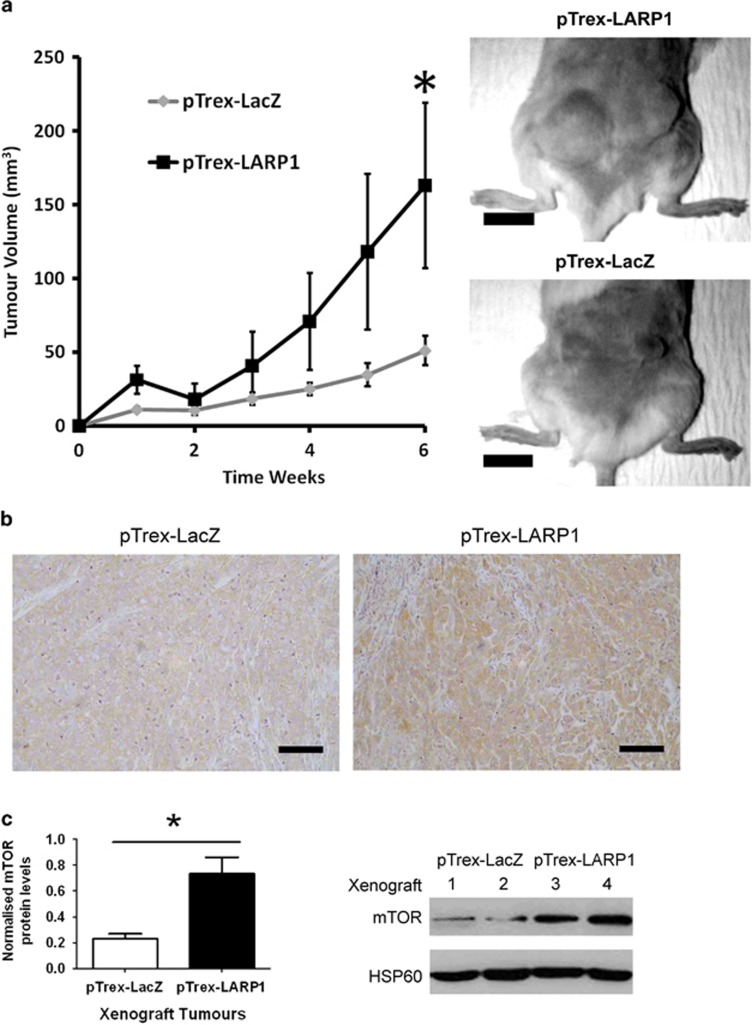
LARP1 promotes tumor progression *in vivo* and is associated with increased mTOR expression in xenograft tumors. (**a**) Two million HeLa pTrex-LacZ or pTrex-LARP1 cells were injected subcutaneously into the posterior flank of non-obese diabetic-severe combined immunodeficiency mice and tumor volume was monitored by caliper measurements. Representative mice from each cohort are shown (scale bars, 1 cm). (**b**) Immunohistochemistry analysis of mTOR protein level in xenograft tumors (scale bars, 200 μm). (**c**) Relative quantification of mTOR protein levels in xenograft tumors generated from pTrex-LacZ and pTrex-LARP1 cells with a corresponding western blot shown (sequential numbers represent tumors from different mice). Data are means±s.e.m. *P⩽0.05.

To investigate whether LARP1 regulates tumorigenicity *in vivo* we assessed the tumor-forming ability of control and LARP1-overexpressing HeLa cells. Two million cells were injected subcutaneously into the flanks of non-obese diabetic-severe combined immunodeficiency mice (*n*=12 tumors per cohort). The experiment was terminated when any mouse reached pre-set welfare limits. LARP1 overexpression resulted in significantly more rapid tumor growth, with a mean final tumor volume of 162.8 mm^3^, compared to 51.0 mm^3^ in the control group. These results show that LARP1 overexpression increases tumorigenicity (Figure 7a). Similar results were obtained when the experiment was repeated in BALB/c nude mice (Supplementary Figure S9a). We subjected xenograft tumors from non-obese diabetic-severe combined immunodeficiency mice to further histological examination. Ki67 positivity was ⩾90% in both LARP1-overexpressing tumors and controls (Supplementary Figure S9b), as has been previously demonstrated for HeLa xenografts.^28^ There was no significant difference in intratumoral vessel density between tumors, as determined by CD31 staining, but there was a trend toward increased vascularity at the periphery of LARP1-overexpressing tumors (Supplementary Figure 9c). Immunohistochemistry staining showed that the level of mTOR was increased in LARP1-overexpressing xenograft tumors (Figure 7b). To further validate this result, total protein was extracted from frozen tumor and expression of mTOR measured by western blotting, where it was confirmed to have increased (Figure 7c).

## Discussion

RBPs contribute to the post-transcriptional regulation of gene expression by binding and regulating the stability of target mRNAs.^29, 30^ We show here using RNA-immunoprecipitation, exon-microarray and bioinformatic analysis that the RBP LARP1 is complexed to an interactome of over 3000 mRNAs, many of which are known to be dysregulated in cancer. To explore the effect of LARP1 interaction on these mRNAs, we examined the relationship between LARP1 and mTOR, one member of the LARP1 interactome. We showed that an association with LARP1 promotes the stability of mTOR messenger RNA leading to enhancement of its protein expression and that this effect is mediated through the 3′UTR of mTOR mRNA. Loss of LARP1 causes a reduction in mTOR protein as well as downstream members of the mTORC1 signaling pathway, including phosphorylated proteins such as RPS6 and 4E-BP1.

Although mTOR is critical regulator of normal processes within the cell, such as metabolism and growth, it is a central component of the PI3K/AKT/mTOR signaling pathway which is frequently dysregulated in cancer.^27^ Increased expression of mTOR and its downstream signaling pathways has previously been shown to lead directly to tumorigenesis and selective inhibitors of mTOR protein are now in use as cancer treatments.^31^ However, relatively little is known about the post-transcriptional regulation of mTOR.^32, 33^ Although 3′UTRs are generically known to contain *cis*-elements, which determine RBP-complex binding or micro-RNA pairing,^26^ the precise nature of (and other components involved in) the binding mechanism between LARP1 and mTOR is as yet undetermined. LARP1 has previously been reported to be a binding partner of the 3′-associated protein PABP and to exist in complex with polyadenylated mRNA.^12, 13, 14, 34^ PABP itself plays a fundamental role in the stability and translation of mRNA by both protecting the transcript from deadenylation and facilitating its circularization.^35, 36^ It is possible that LARP1 acts synergistically with PABP perhaps by strengthening the interaction between PABP and the 5′-cap components. This would support previous research showing LARP1 protein is in complex with eIF4E and eIF4G, in addition to PABP.^34^

Recently, in benign cell lines, LARP1 has been reported to regulate 5′-TOP mRNA transcripts by stabilizing them and/or enhancing their translation.^13, 14^ Consistent with these findings, we identified a number of 5′-TOP transcripts within the HeLa-LARP1 interactome. However, we show that, in HeLa at least, LARP1 does not associate with all TOP mRNAs and some, such as RPS6, were absent from the interactome. In our hands, LARP1 binds a wider population of mRNAs, such as those involved in focal adhesion, actin remodeling and extracellular matrix interactions, as well as components of the VEGF and MAPK signaling pathways. This implies that in cancer cells the LARP1 interactome is more extensive than has been described previously.

Overall, we see that LARP1 is involved in modulating the tumorigenicity of cancer cells and its overexpression drives tumor progression *in vivo*. Although mTOR overexpression has been shown to contribute to oncogenic transformation, it is likely that LARP1 affects tumorigenicity via an effect on multiple signaling pathways. In addition, by affecting key proteins such as P-RPS6 and P-4E-BP1 downstream of the mTORC1 signaling pathway, LARP1 indirectly affects global protein synthesis.^14, 34^

It is probable that RBPs like LARP1 do not act alone but work collectively with other RNA-associated factors to stabilize or destabilize its target mRNAs. Although we show the net effect of LARP1 in these cancer cells is to stabilize and enrich protein expression, confirmed here at transcript level with mTOR, it is possible that LARP1 destabilizes some transcripts while stabilizing others. This has been observed with other RBPs such as HuR, which plays a predominant role in mRNA stability but can also promote the decay of p16INK4 and c-Myc.^23, 37, 38^ In *Arabidopsis*, the net effect of LARP1 is to cause mRNA decay in response to cell stress, via an interaction with the 5′–3′ exonuclease enzyme exoribonuclease 4 (XRN4).^39^ In human embryonic kidney cells the net effect of LARP1 is also positive, stabilizing TOP mRNAs and ensuring the ongoing manufacture of proteins required for growth-related ribosome biogenesis. This implies that the function of LARP1, either as an activator or repressor of mRNA stability is context specific.

In non-small cell lung and cervical cancer, higher levels of LARP1 protein correlate with tumor progression and adverse survival. Cervical cancer is known to be a genomically complex tumor with considerable intratumoral heterogeneity.^40^ Our findings support the importance of post-transcriptional regulation in the progression of cervical cancer and demonstrate that, through its regulation of target mRNAs such as mTOR, LARP1 plays a central role. Two independent reports have shown that LARP1 is phosphorylated by mTOR and is an important effector of mTORC1 signaling.^15, 16^ We speculate that LARP1 regulates the activity of mTORC1 in a positive-feedback loop by being a downstream substrate of mTORC1 and simultaneously regulating the stability of its mRNA transcript.

As LARP1 is highly expressed in multiple epithelial cancer types, its upregulation may represent a common feature of tumor progression. RBPs like LARP1, which selectively regulate the stability of multiple mRNA transcripts, are exciting potential therapeutic targets, as their inhibition could simultaneously target multiple oncogenic pathways.^5^ By acting at a point of convergence of multiple cancer signaling cascades, LARP1 is an important post-transcriptional regulator of cancer progression and may prove to be a target for therapeutic intervention.

## Materials and methods

### Oncomine data analysis

Expression data for LARP1 were downloaded from Oncomine.^20^ Fold change was calculated as median-centered intensity of each cancer sample divided by the mean of non-cancer samples.

### Immunohistochemistry

Two cervical cancer tissue arrays were obtained commercially from US Biomax (Rockville, MD, USA) were stained as described by Shi *et al.*^41^ Cytoplasmic scores were calculated as a sum of staining intensity (0–3, with 3 being most intense) multiplied by the percentage of stained cells (0–100%), giving a range of 0–300. All scorings were performed ‘blind' by consultant clinical histopathologists. All images were captured using a Nikon Eclipse ME600 (Nikon, Kingston Upon Thames, UK) (antibodies: LARP1-SDIX, mTOR-CST, Ki67-Leica and CD31-NovusBiological).

### RNA from patient samples

RNA from cervical cancer tissue samples were obtained from the Imperial College Healthcare Tissue Bank. Samples were collected under the College's Human Tissue Authority license approved by the local Research and Ethics Committee and the Tissue Management Committee.

### Cell culture

The identity of cervical cancer (HeLa) and non-small cell lung cancer (PC9) cell lines was confirmed by genotyping (Genetica DNA Laboratories, Burlington, NC, USA). Cells were cultured with complete Dulbecco's Modified Eagle's medium (DMEM) (HeLa) or RPMI-1640 (PC9) in a 5% CO_2_ atmosphere, at 37 °C.

### RNA immunoprecipitation and expression array analysis

RNA was immunoprecipitated with 15-μg LARP1 polyclonal antibody (SDIX) or IgG isotype control (CST) following the method described by Keene *et al.*^42^ RNA was extracted with Trizol (Life Technologies, Carlsbad, CA, USA) and purified with RNA clean-up and concentration micro kit (Norgen Biotek, Thorold, ON, Canada). Input and immunoprecipitated RNA obtained from four independent experiments were analyzed in duplicate on Agilent exon arrays by Oxford Gene Technology (Begbroke, UK). RNA was quality assessed in a bioanalyser with the RNA 6000 pico kit (Agilent Technologies, Santa Clara, CA, USA), labeled with Agilent low-input QuickAmp WT labeling kit (Agilent Technologies) and hybridized to the Agilent 4x180 K Exon arrays (Agilent Technologies) as per manufacturer protocols.

### RIP-Chip data analysis

Data from Exon arrays were normalized in Agilent's Feature Extraction software (Agilent Technologies), according to the manufacturer's instructions. The normalized log2 intensity ratios of RIP fraction over total RNA were obtained for each replicate array. Statistical significance of enrichment was estimated by calculating empirical Bayes moderated *t*-statistics using Linear Models for Microarray Data (LIMMA),^43^ as implemented in Bioconductor (http://www.bioconductor.org). Benjamini-Hochberg correction was used to adjust *P*-values for multiple testing. Probes with a fold change >2 and an adjusted *P*-value <1 × 10^−4^ were considered to be significantly enriched in the immuno-precipitated RNA, and members of the LARP1 interactome.

### Actinomycin-D treatment

HeLa cells were transfected with control siRNA (GGUCCGGCUCCCCCAAAUG) or a mixture of two different LARP1-targeting siRNA (siRNA1- AGACUCAAGCCAGACAUCA and siRNA2- GAAUGGAGAUGAGGAUUGC) as described earlier.^6^ Each siRNA targets both LARP1 isoforms (150 and 130 kD).^44^ Forty-eight hours after the first transfection, cells were treated with 5 μg/ml of actinomycin-D for up to 4 h. RNA was extracted and processed as described below.

### UTRs luciferase assay

mTOR 3′UTR and its relative control plasmid with firefly-luciferase reporter system were purchased from Origene Technologies (Rockville, MD, USA). mTOR 5′UTR (GGGGCCTGAAGCGGCGGTACCGGTGCTGGCGGCGGCAGCTGAGGCCTTGGCCGAAGCCGCGCGAACCTCAGGGCAAG) was cloned into the pLightSwitch_5UTR (SwitchGear Genomics, Carlsbad, CA, USA) with renilla luciferase reporter system. Beta actin 5′UTR was used as a control. HeLa cells were transfected with plasmid DNA using Effectene (Qiagen, Limburg, Netherlands) according to the manufacturer's instructions, following transient siRNA knockdown of LARP1. Renilla and firefly control vectors were co-transfected respectively with the firefly and renilla-UTR vectors in a ratio of 25:75 to take into account the efficiency of transfection and cell numbers. Luciferase activity of the construct carrying either the 5′ or 3′ UTR was normalized to the relative control construct. Luciferase activity was assessed with the Dual-Luciferase Reporter assay system (Promega, Madison, WI, USA). Luminescence was measured using the Lumistar OPTIMA plate reader (BMG Labtech, Aylesbury, UK).

### Q-RT–PCR

RNA from patients and from immunoprecipitated samples was retrotranscribed with SensiScript RT kit (Qiagen). RNA from cells treated with actinomycin-D was extracted with RNeasy plus mini kit (Qiagen) and retrotranscribed using MMLV reverse transcriptase (Promega). RT–PCR was performed with TaqMan assay primers and probes (Applied Biosystems, Life Technologies), and run on the ABI 7900HT. 18S rRNA was used as control reference as it remained stable after LARP1 knockdown and was not affected by actinomycin-D treatment. Relative mRNA levels were calculated using the ΔΔCt formula. The fold enrichment of immunoprecipitated RNA was measured by comparing the Ct values of LARP1-IP fraction to the IgG-IP fraction and normalized using the ΔΔCt formula (primers: LARP1-HS00369275_S1, AKT1-HS00178289_m1, AKT2-HS01086102_m1, MAPK11-HS011558727_gH, MAPK12-HS00268060_m1, MAPK14-HS01051152_m1, TWIST1-HS00361152_m1, MTOR-HS01042412_m1 and 18-HS99999901_s1).

### Protein extraction and western blotting

Western blotting was performed as previously described.^34^ Xenograft tumors were homogenized with a polypropylene pellet pestle (Sigma-Aldrich, St Louis, MO, USA) and lysates cleared using centrifugation. Clean-Blot IP detection reagent (Pierce Biotechnology, Rockford, IL, USA) was used for detection of immunoprecipitated LARP1 (antibodies: LARP1-SDIX, mTOR-CST, RPS6-CST, RPS6-p240/244-CST, 4EBP1-CST, 4EBP-P37/46-CST and HSP60-ABCAM).

### Reverse-phase protein array

HeLa cells were transfected with control non-targeting or a mixture of two different LARP1-targeting siRNAs as described earlier.^34^ Samples were analyzed by the MD Anderson Cancer Center Proteomic Facility (Houston, Texas, USA), as previously described by Tibes *et al.*^45^ Normalized Log2 intensity of fluorescence was used to calculate changes in protein expression. A change of 10% relative to control was considered to be functionally relevant.

### Stable clone generation

LARP1 cDNA was obtained from the pOTB7-LARP1 vector (Life Technologies). PCR primers were designed for gene amplification with the addition of attB sites (forward: 5′-GgggacaagtttgtacaaaaaagcaggcttcgaccatggGTCAAAGAGGCCTCCTTTCC-3′ and reverse: 5′-GgggaccactttgtacaagaaagctgggtcctagctaTTTCACTTTCCCAAAGTCTG-3′). The PCR product attB-LARP1 was cloned into the Gateway Technology System expression plasmid pTrex-DEST30 according to the manufacturer's instructions (Life Technologies). HeLa and PC9 cells were transfected with 1 μg of pTrex-LARP1 and pTrex-LacZ using Effectene (Qiagen) according to the manufacturer's instructions. To generate stable clones, 48 h after transfection HeLa cells were cultured in DMEM containing Geneticin (Life Technologies) at a 700 μg/ml final concentration until at least 200 individual colonies were formed. Pooled clones were used for subsequent experiments.

### Migration assay

pTrex-LARP1 and pTrex-LacZ HeLa cell-derived stable clones or pTrex-LARP1 and pTrex-LacZ transiently transfected PC9 cells were cultured to confluence and serum-starved overnight (1% Fetal Calf Serum (FCS)) before a scratch was applied with a pipette tip. Pictures were acquired with a Nikon Eclipse TE-2000U microscope and surface area of the open wound was measured using ImageJ (http://imagej.nih.gov/ij).

### Invasion assay

Approximately 1 × 10^5^ cells in serum-free medium were plated in the upper insert of a BD BioCoat Matrigel Invasion Chamber (BD Biosciences) with 8-μm pores, while the bottom well was filled with medium supplemented with 10% FCS. After 24-h incubation, non-invasive cells were removed from the upper surface of the membrane. Cells in the lower surface of the membrane were fixed with ice-cold methanol and stained with 4′,6-diamidino-2-phenylindole (DAPI). Images were acquired with Leica 500 confocal microscope and processed with Leica LAS AF lite software (Leica Camera AG, Solms, Germany). DAPI-stained nuclei were counted with ImageJ.

### Anchorage-independent growth assay

Approximately 1 × 10^3^/ml cells were seeded in ultra-low attachment surface 96-well plates (Corning Incorporated, Corning, NY, USA). Single-cell suspensions were incubated in complete medium for 2.5 weeks. Spherosomes were counted and then dissociated with trypsin to a single-cell suspension. Colonies formed after 3 weeks were fixed with ice-cold methanol and stained with 0.5% crystal violet. Plates were photographed using a GE ImageQuant LAS 4000 (GE Healthcare Life Sciences, Little Chalfont, UK) and colonies were counted using ImageJ software.

### MTT enzymatic conversion assay

Cells were cultured at 37 °C and labeled with 10 μl of 3-(4,5-Dimethylthiazol-2-yl)-2,5-Diphenyltetrazolium Bromide MTT (Sigma) at 3 mg/ml for 2 h. The precipitate was solubilized overnight with 10% SDS in 0.01-m HCl. Absorbance at 562 nm was recorded using an OPTImax microplate reader (Molecular Devices, Winnersh, UK).

### *In vivo* work

All animal experiments were performed by licensed investigators in accordance with the United Kingdom Home Office Guidance on the Operation of the Animal (Scientific Procedures) Act 1986 and within the newly published guidelines for the welfare and use of animals in cancer research.^46^ Female non-obese diabetic-severe combined immunodeficiency or BALB/c nude mice (aged 6–8 weeks; Charles River Laboratories, Wilmington, MA, USA) were used. HeLa pTrex-LARP1 and pTrex-LacZ cells (2 × 10^6^) were injected subcutaneously on the flanks of mice (at least five per cohort). Tumor dimensions were measured continuously using a caliper and tumor volumes were calculated by the equation: volume=(π/6) × *a* × *b* × *c*, where *a*, *b* and *c* represent three orthogonal axes of the tumor. The experiment was terminated before any mouse reached pre-set welfare limits. Tumors were collected and either immediately snap-frozen and preserved at −80 °C, or fixed in 10% formalin for 48 h before paraffin embedding and sectioning.

### Statistical analysis

Statistical analysis was performed using GraphPad Prism software (GraphPad Software, San Diego, CA, USA), using Student's *t*-test unless otherwise stated in the Materials and methods section. S.e.m. was used as the measure of variation from the population mean in all our experiments; degree of statistical significance was represented using asterisks: *P*⩽0.05 (*), *P*⩽0.001 (**) and *P*⩽0.0001 (***).
