# Supplementary material for: LARP1 post-transcriptionally regulates mTOR and contributes to cancer progression
Source: Oncogene. 2014 Dec 22;34(39):5025–36. doi: 10.1038/onc.2014.428 (PMC4430325; doi:10.1038/onc.2014.428)
Supplement: Supplementary Figures and Tables legend [file onc2014428x1.doc]

# **Supplementary figures and tables legend**

# **Supplementary Figure 1. LARP1 mRNA levels are elevated across a range of human carcinomas and correlate with adverse prognosis in non-small lung cancer.**

# (a) A systematic survey of expression studies in the Oncomine portal comparing LARP1 mRNAlevels between cancer and non-cancer tissue for multiple carcinomas, including intraductal (ID) breast, hepatocellular (HCC) and lung adenocarcinomas. All p-values <0.0001, except TCGA ovarian (p = 0.002), TCGA brain (p = 0.004) and Hou Lung Adenocarcinoma (p=0.0002) datasets. For full data see Supplementary Table 1. (b) Kaplan-Meier survival analysis generated with *kmplot* of 1,405 lung cancer patients stratified into high and low LARP1 expressors based on expression array values (p<0.0001).

# **Supplementary Table 1. LARP1 mRNA levels are elevated across a range of human carcinomas as revealed by Oncomine analysis**.

Search criteria for expression studies comparing LARP1 mRNA levels in cancer with relevant control, together with studies identified, relevant fold changes and p-values.

# **Supplementary Figure 2. LARP1 Immunoprecipitation**

(a) Western blotting showing LARP1 immunoprecipitation versus IgG immunoprecipitation and input.

# **Supplementary Table 2. The LARP1 interactome transcripts**.

# LARP1 RIP-enriched transcripts (fold change>2, p-value<0.05) ranked by the significance of enrichment.

# **Supplementary Table 3. The LARP1 interactome is enriched for pathways dysregulated in cancer.**

# Functional enrichment analysis of transcripts in complex with LARP1 with Database for Annotation, Visualization and Integrated Discovery (DAVID) (raw p-values and Benjamini-Hochberg adjusted).

Supplementary Figure 3. mRNA stability assay on MAPK12 and TWIST1.

Real-time PCR following transcription inhibition by actinomycin- D treatment in HeLa cellstransfected with 2 pooled LARP1 siRNA sequences or control siRNA . Fold changes for MAPK12 (a) and TWIST1 (b) relative to 18S, normalized to untreated control. Experiments were performed ≥ 3 times. Data are mean ±SEM.

Supplementary Figure 4. Quantification of mTOR pathway protein level after LARP1 knockdown.

Quantification measured by densitometry of protein bands in x-ray films normalized to HSP60 loading control band in HeLa (a) and PC9 (b) cell lines, using ImageJ softwsare.

# **Supplementary Figure 5. LARP1 alters the expression of protein and phosphoproteins beyond the interactome targets.**

# (a) Reverse phase protein array analysis (10% change threshold) after LARP1 knockdown with pooled siRNA of proteins beyond LARP1-interactome. (b) Reverse phase protein array shows altered expression in phosphorylated proteins.

Supplementary Table 4. Summary of protein corresponding to targets present in LARP1 interactome and beyond LARP1 interactome which are unaltered after LARP1 knock-down.

Supplementary Figure 6 . LARP1 promotes migration in HeLa cells

(a) Western blotting showing protein level of LARP1 in HeLa cells stably transfected using pTrexLacZ and pTrex-LARP1 (b) Wound healing assays in HeLa cells stably over-expressing LARP1 (pTrex-LARP1) vs control (pTrex-LacZ). The graph shows the quantification of the unhealed area at given time points.

# **Supplementary Figure 7. LARP1 promotes migration and invasion in PC9 cells**

(a) Western blotting showing protein level of LARP1 in PC9 cells transfected using pTrex-LacZ and pTrex-LARP1 constructs. (b) Wound healing assays in PC9 cells over-expressing LARP1 (pTrex-LARP1) vs control (pTrex-LacZ). The graph shows the quantification of the unhealed area at given time points. (c) Invasion assay performed with PC9 cells transfected with siLARP1-1 and siLARP1-2 compared to siRNA control. Graphs are counts of the number of invasive cells per cm^2^. Representative images of DAPI-stained invasive cells are shown (Scale bar, 250µM). (d) Cell viability assay using MTT in PC9 transfected using pTrex-LacZ and pTrex-LARP1 (e) Number of viable cells do not change after LARP1 knockdown during the time course of the experiments. Experiments were performed ≥ 3 times; Data are mean ± SEM

# **Supplementary Figure 8. LARP1 viability in HeLa cell lines.**

(a) Viability assay using MTT and (b) CellTiter-Glo shows no effect on proliferation in pTrex-LARP1 and pTrex-LacZ clones. (c) Viable cell number does not change after LARP1 knockdown during the time course of the experiments.

# **Supplementary Figure 9. LARP1 promotes tumorigenesis *in vivo* and markers of proliferation and angiogenesis are unaltered.**

(a) Final tumor volumes of pTrex-LacZ and pTrex-LARP1 xenografts in BALB/c nude mice (b) IHC staining of the proliferation marker Ki67 in pTrex-LARP1 and pTrex-LacZ xenograft tumors (Scale bar, 200µm) (c) IHC staining of mouse CD31 shows no significant differences in intratumoral vessel density, but a trend towards increased vascularity at the tumor periphery in LARP1-overexpressing tumors (Scale bar, 50 µm).
