# Supplementary figures and images for: LARP1 post-transcriptionally regulates mTOR and contributes to cancer progression
Source: Oncogene. 2014 Dec 22;34(39):5025–36. doi: 10.1038/onc.2014.428 (PMC4430325; doi:10.1038/onc.2014.428)

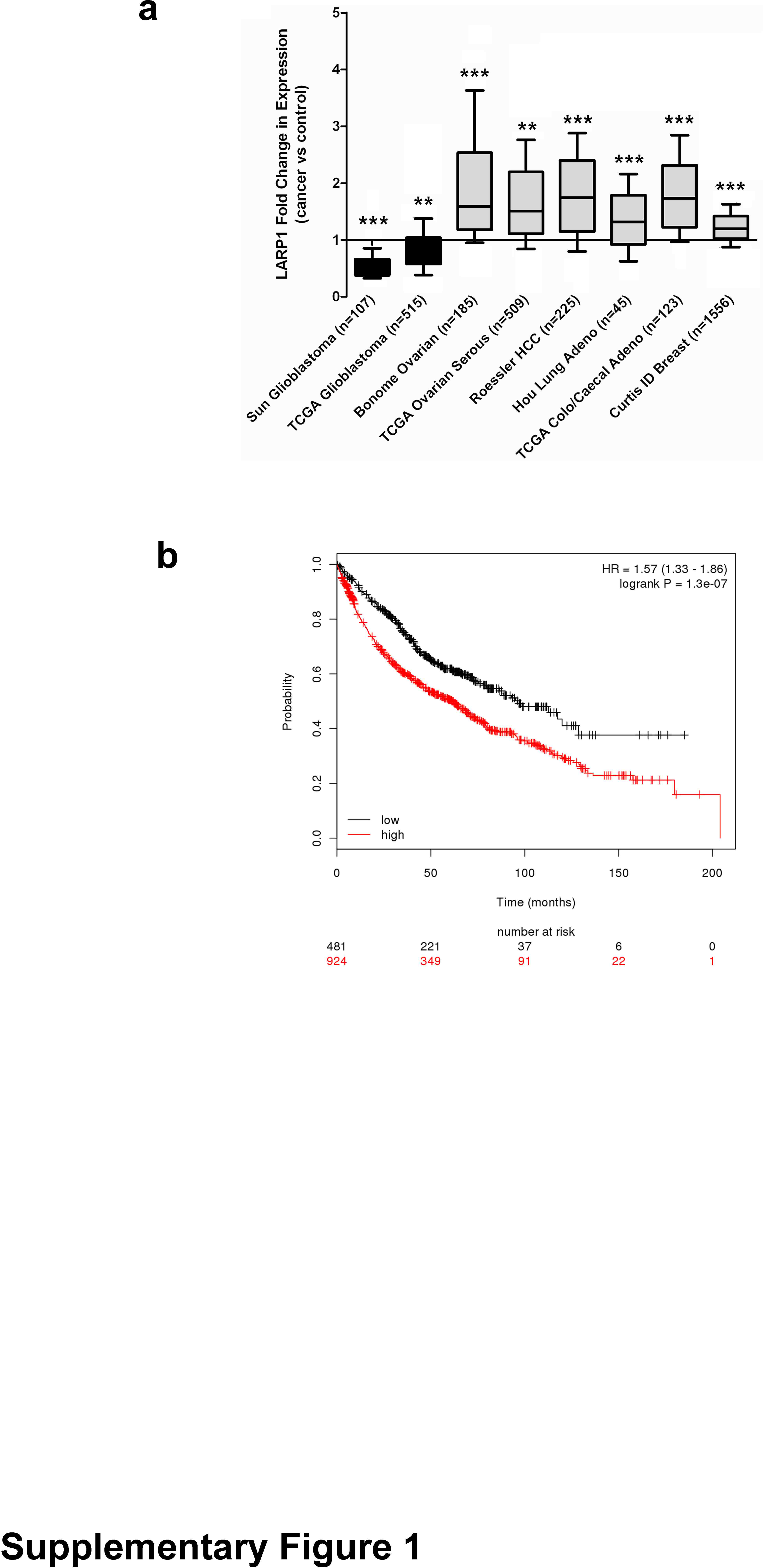

Supplement: Supplementary Figure 1 [file onc2014428x2.tif]

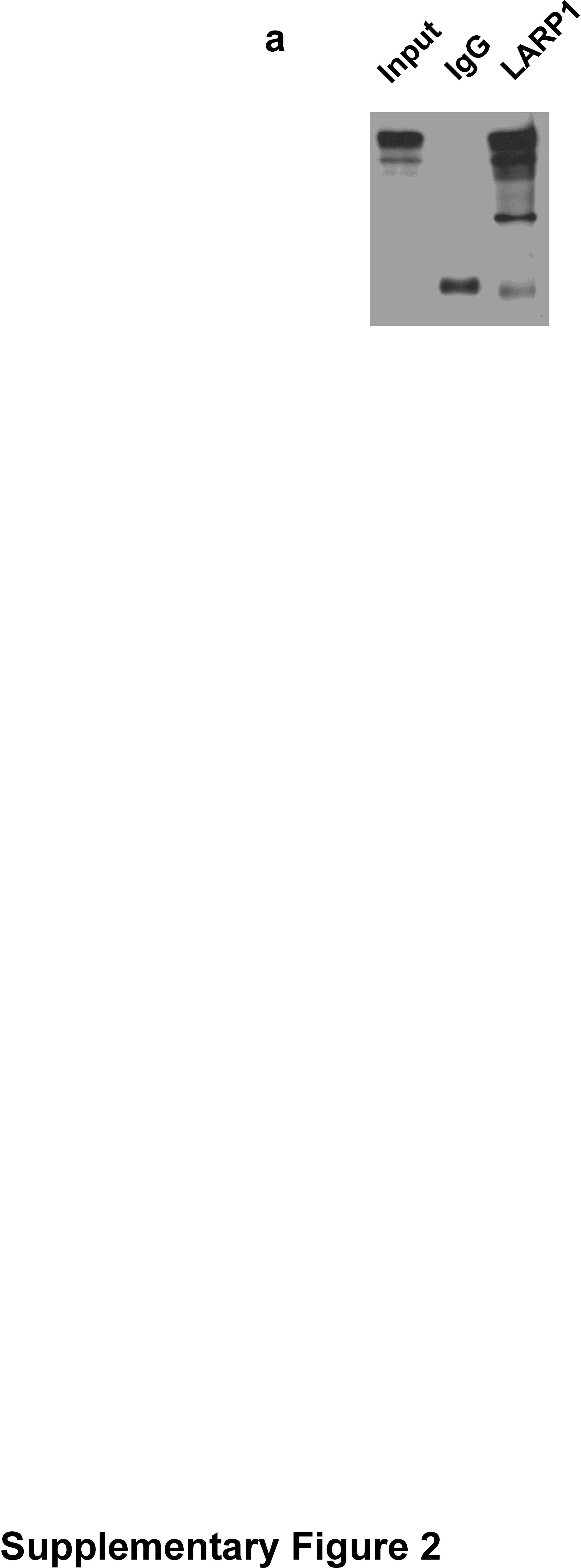

Supplement: Supplementary Figure 2 [file onc2014428x3.tif]

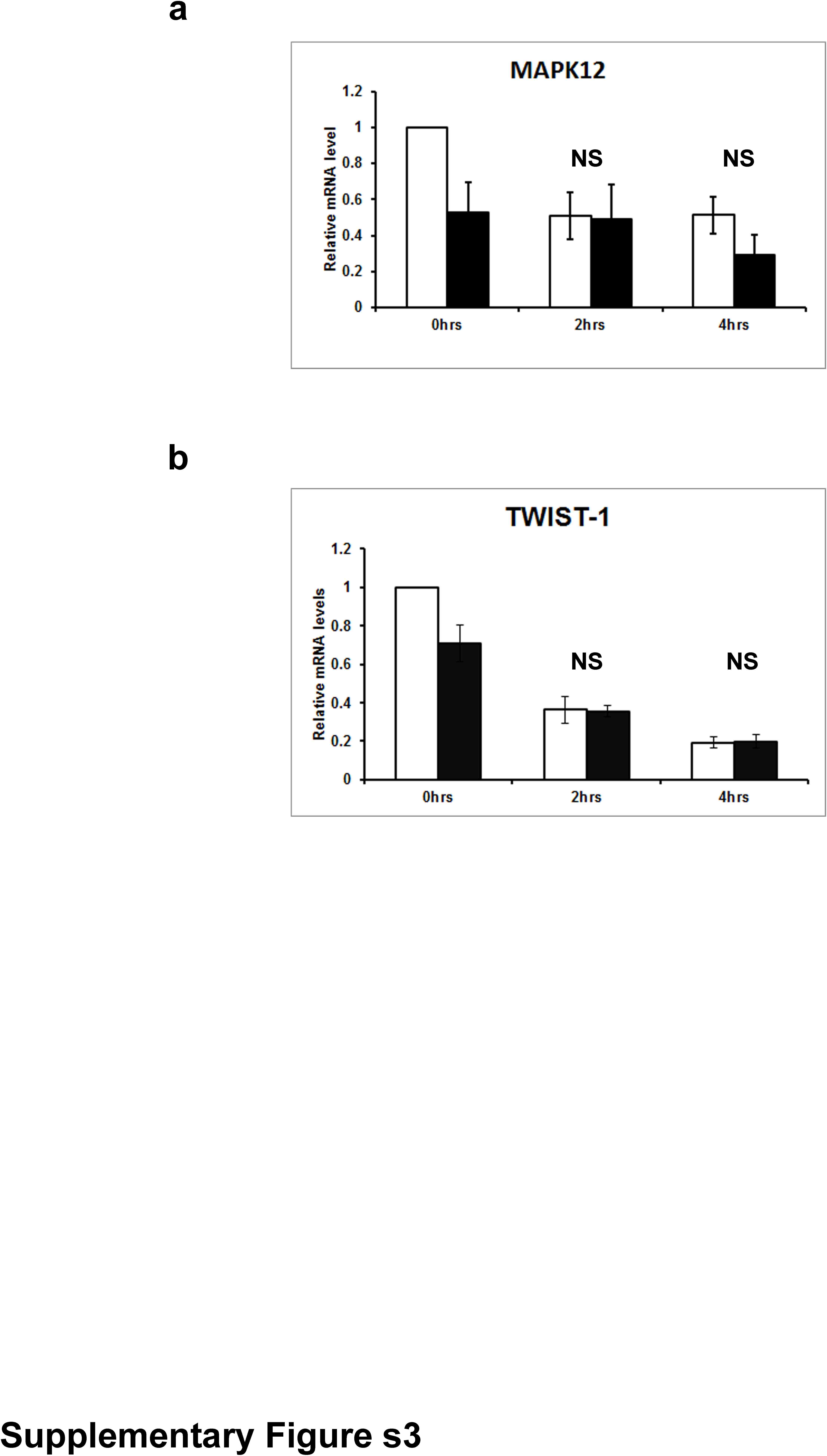

Supplement: Supplementary Figure 3 [file onc2014428x4.tif]

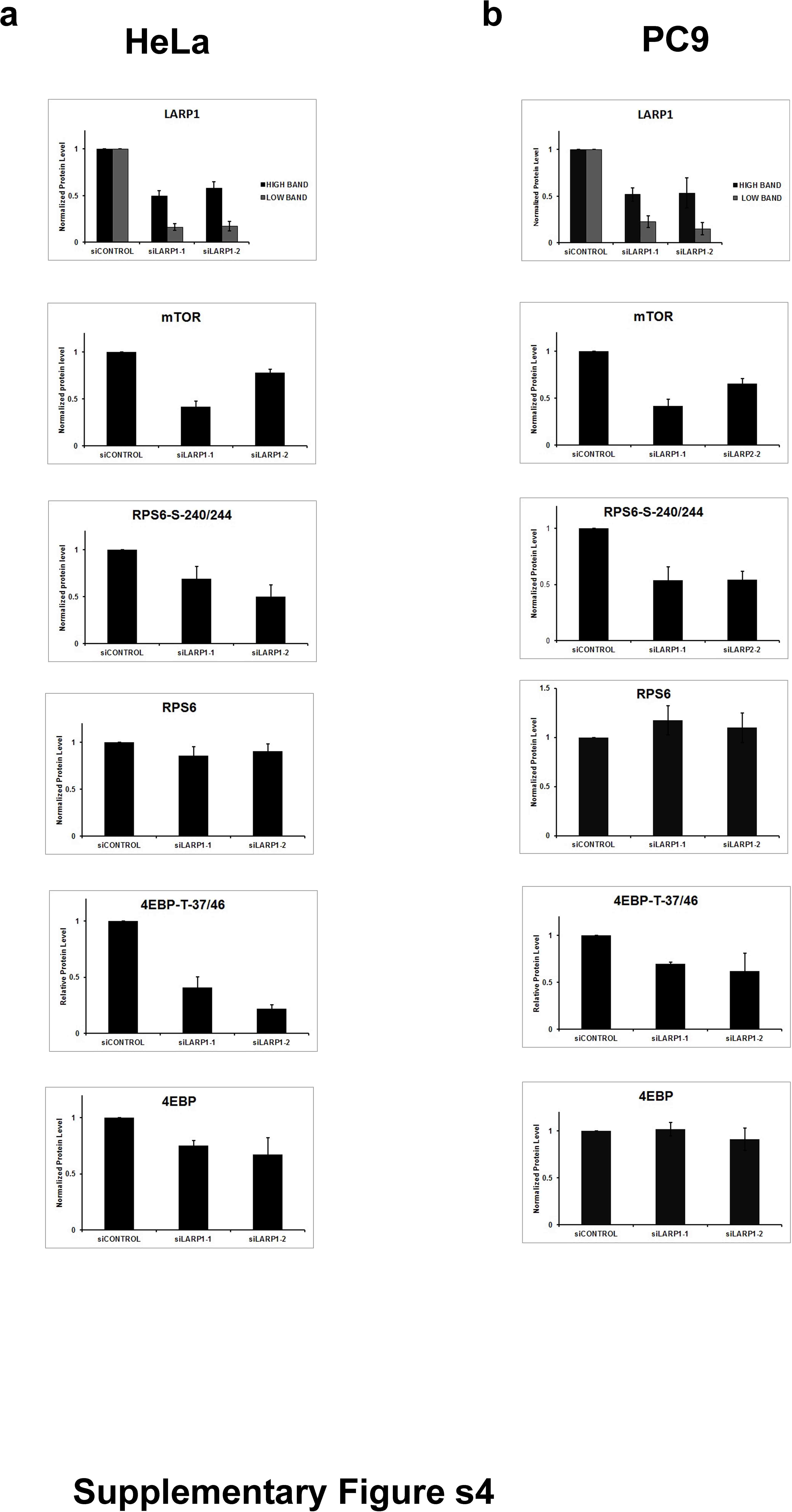

Supplement: Supplementary Figure 4 [file onc2014428x5.tif]

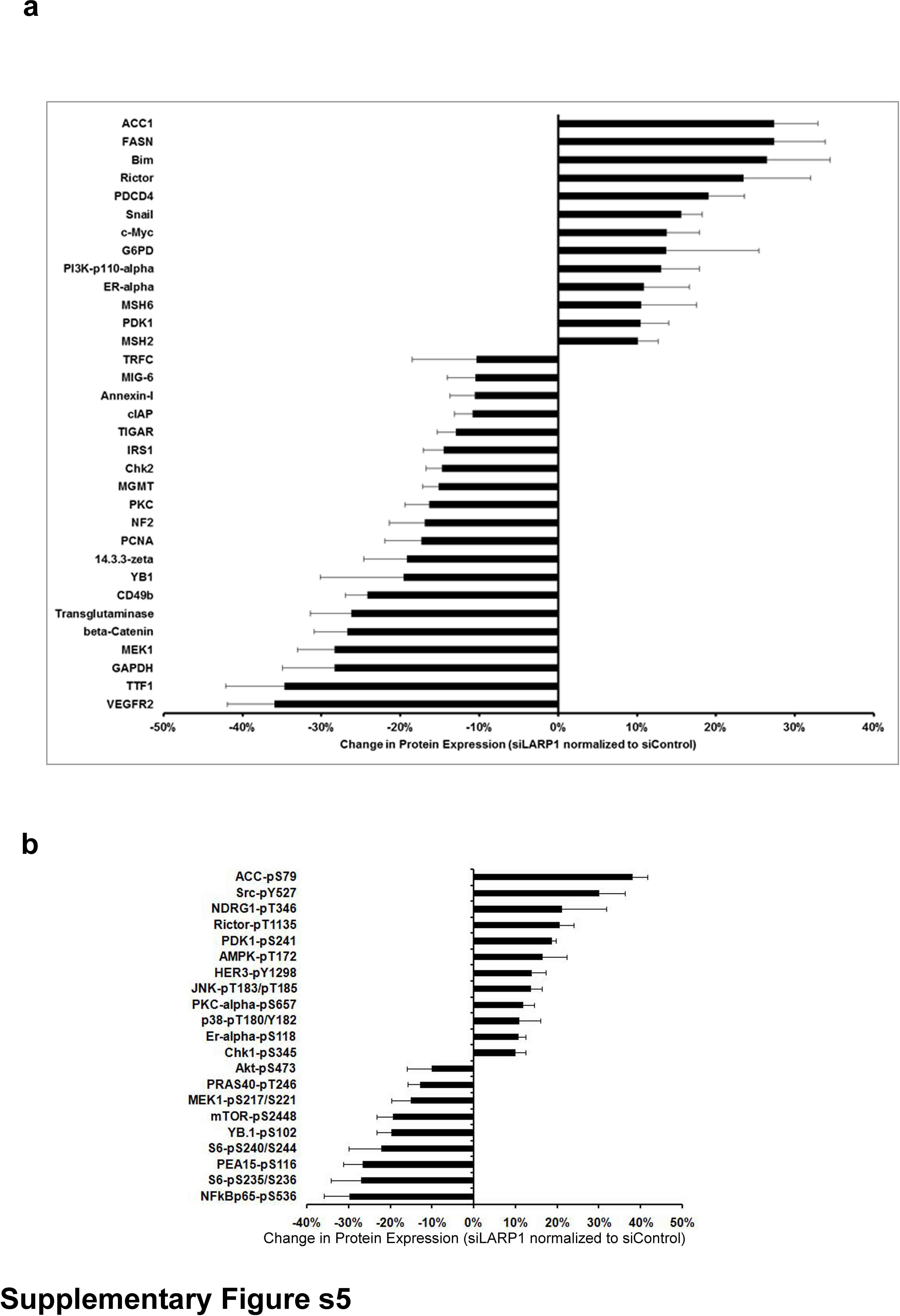

Supplement: Supplementary Figure 5 [file onc2014428x6.tif]

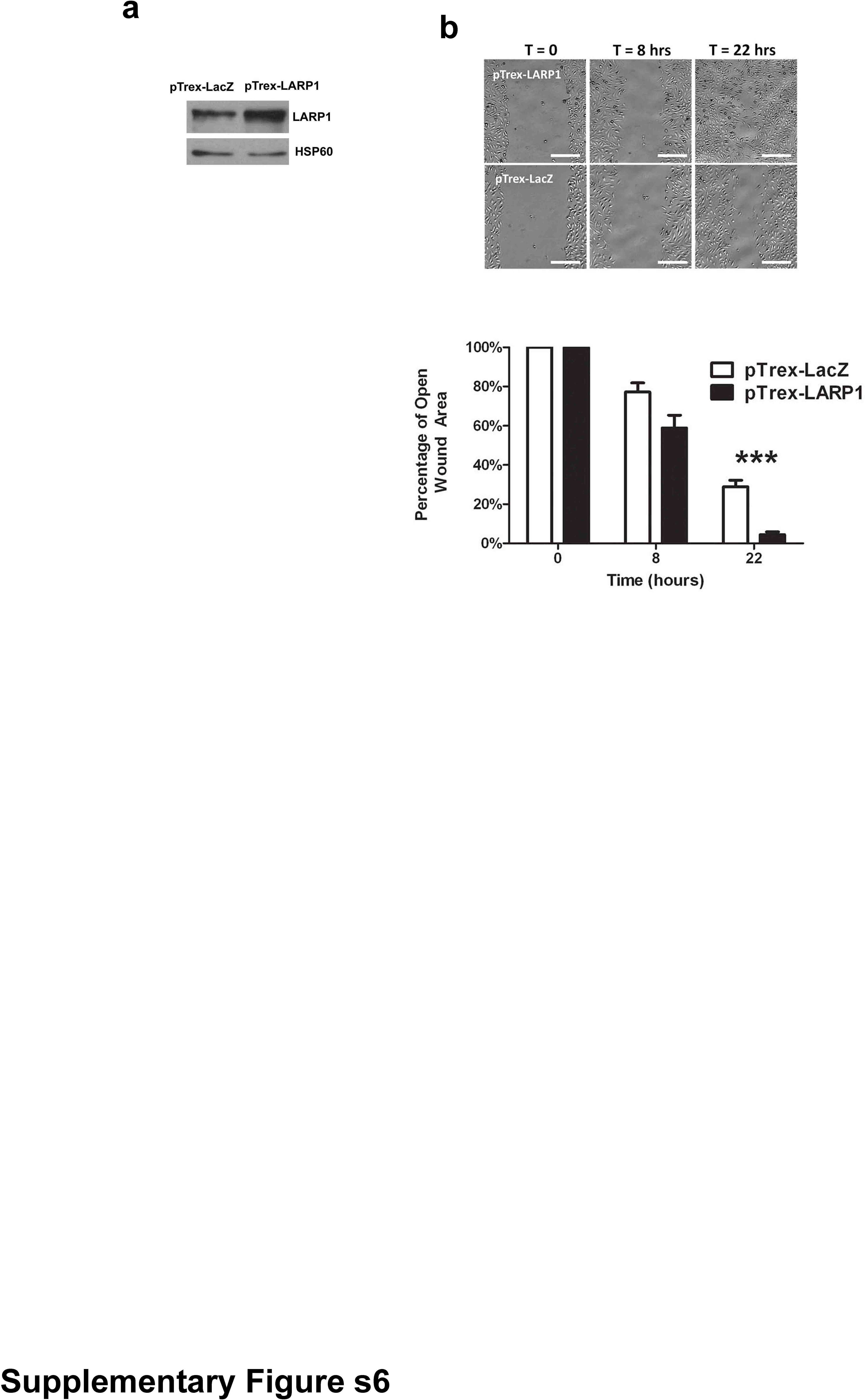

Supplement: Supplementary Figure 6 [file onc2014428x7.tif]

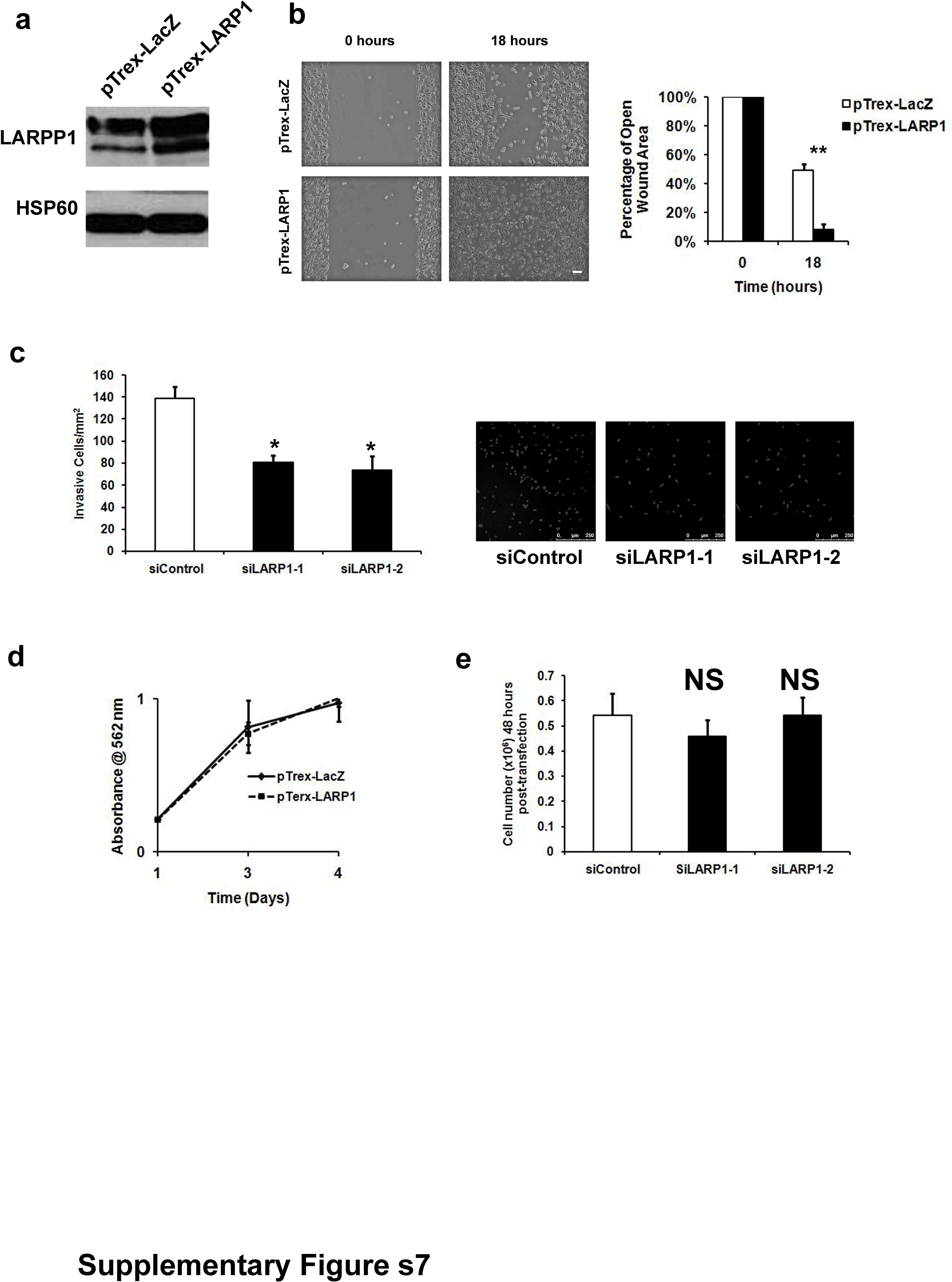

Supplement: Supplementary Figure 7 [file onc2014428x8.tif]

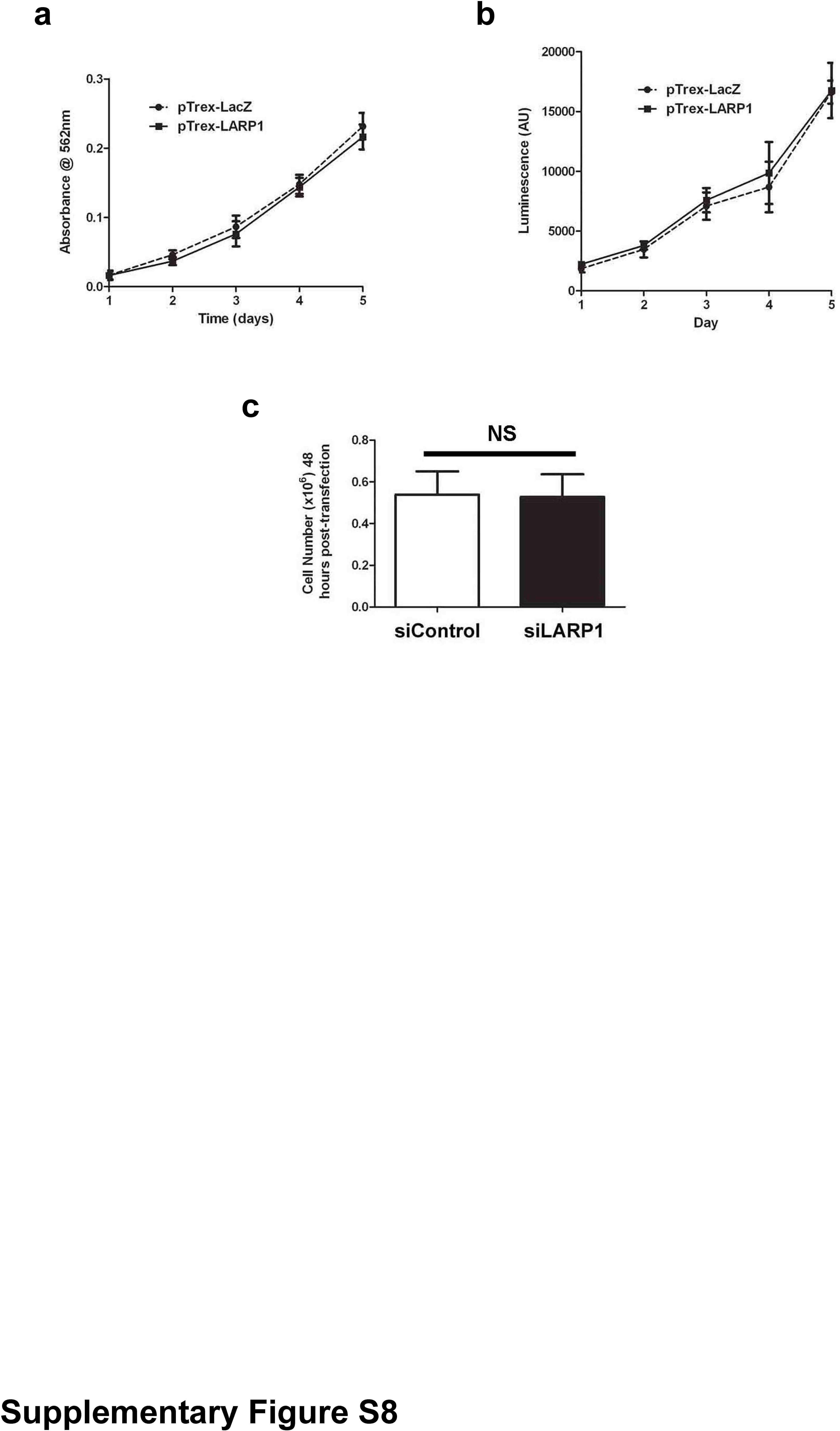

Supplement: Supplementary Figure 8 [file onc2014428x9.tif]

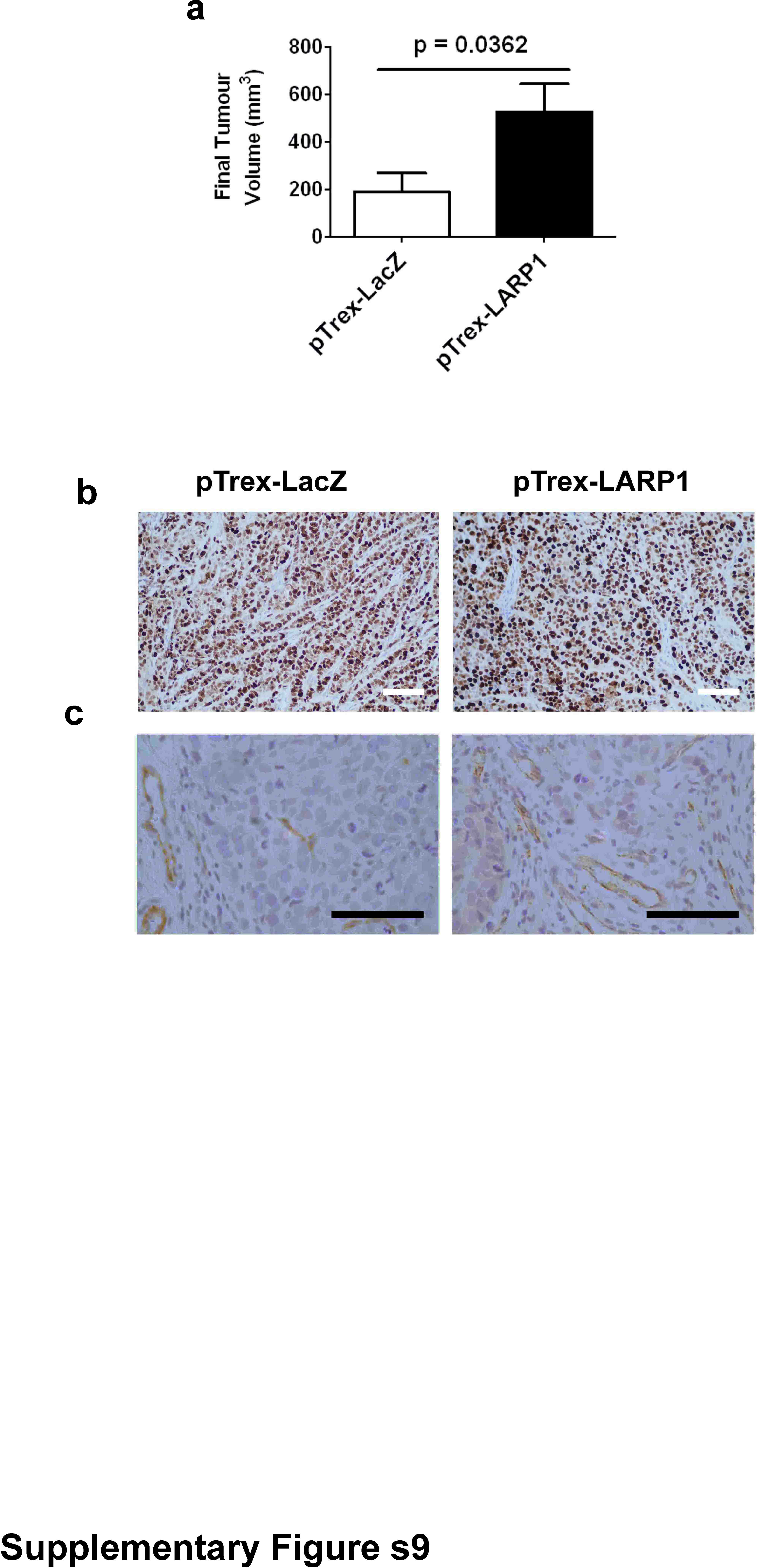

Supplement: Supplementary Figure 9 [file onc2014428x10.tif]
