# Supplementary material for: LARP1 post-transcriptionally regulates mTOR and contributes to cancer progression
Source: Oncogene. 2014 Dec 22;34(39):5025–36. doi: 10.1038/onc.2014.428 (PMC4430325; doi:10.1038/onc.2014.428)
Supplement: Supplementary Table 1 [file onc2014428x11.pdf]

Supplementary Table 1

ONCOMINE SEARCH CRITERIA/THRESHOLDS

Cancer	Adult Carcinomas
Dataset Size	151+ (minimum 40 per cancer subtype)
Analysis	Cancer vs Normal
Data Type	mRNA
Platform	Affymetrix U133 (where possible)
Fold change	>1.1 or <0.9
P value	less that 0.01

	Oncomine Database Name (Data Source)	Platform	Cancer Type Investigated	Controls	Cases	Probe	Fold Change	P-Value
1	Bonome Ovarian (Cancer Res 2008/07/01)	Affymetrix U133	Ovarian Carcinoma	10	185	212193_s_at	**1.98**	***< 0.0001***
2	Haferlach Leukaemia (J Clin Oncol 2010/05/20)	Affymetrix U133 plus 2.0	EXCLUDED					
3	Roessler Liver 2 (Cancer Res 2010/12/15)	Affymetrix U133	Hepatocellular Carcinoma	220	225	212193_s_at	**1.84**	***< 0.0001***
4	Hou Lung (PLoS One 2010/04/22)	Affymetrix U133 plus 2.0	Lung Adenocarcinoma	65	45	212193_s_at	**1.40**	***0.0002***
5	TCGA Ovarian (TCGA 2011/03/24)	Affymetrix U133	Ovarian Serous Carcinoma	8	509	212193_s_at	**1.70**	***0.0024***
6	Agnelli Myeloma (Genes Chrom Cancer 2009/07/01)	Affymetrix U133	EXCLUDED					
7	Sun Brain (Cancer Cell 2006/04/01)	Affymetrix U133 plus 2.0	Glioblastoma	23	81	212193_s_at	**0.56**	***< 0.0001***
8	TCGA Brain (TCGA 2012/03/01)	Affymetrix U133	Glioblastoma	10	515	212193_s_at	**0.83**	***0.0037***
9	Coustan-Smith Leukaemia (Lancet Oncol 2009/10/02)	Affymetrix U133	EXCLUDED					
10	Valk Leukaemia (N Engl J Med 2004/04/15)	Affymetrix U133	EXCLUDED					
11	Sanchez-Carbayo Bladder 2 (J Clin Oncol 2006/02/10)	Affymetrix U133	Infiltrating Bladder Uroepithelial Cancer	48	81	212193_s_at	**1.50**	***< 0.0001***
12	Barretina Sarcoma (Nat Genet 2010/07/04)	Affymetrix U133	EXCLUDED					
	NON-AFFYMETRIX STUDIES (largest studies added to include common cancers otherwise not represented)							
13	TCGA Colorectal (TCGA 2011/09/08)	Agilent 244K Microarray (AMDID019760)	Colon/Caecal Adenocarcinoma	22	123	A_24_P7212	**1.82**	***< 0.0001***
14	Curtis Breast (Nature 2012/06/21)	IlluminaHT	Invasive Ductal Breast Cancer	144	1556	ILMN_1681590	**1.18**	***< 0.0001***
