# Supplementary material for: LARP1 post-transcriptionally regulates mTOR and contributes to cancer progression
Source: Oncogene. 2014 Dec 22;34(39):5025–36. doi: 10.1038/onc.2014.428 (PMC4430325; doi:10.1038/onc.2014.428)
Supplement: Supplementary Table 2 [file onc2014428x12.pdf]

Transcript Rank	Probe ID	Adjusted p-value	Gene
1	A_37_P185851	2.70E-007	TRABD
2	A_37_P020165	3.08E-007	SLC25A38
3	A_37_P260611	3.61E-007	NAT15
4	A_37_P143081	3.80E-007	BAX
5	A_37_P186119	3.95E-007	CERK
6	A_37_P385863	4.02E-007	C7orf23
7	A_37_P053025	4.16E-007	RNPEPL1
8	A_37_P315524	4.16E-007	NPDC1
9	A_37_P173553	4.16E-007	CLN6
10	A_37_P146214	4.16E-007	HCN2
11	A_37_P387872	4.31E-007	FASTK
12	A_33_P3259722	4.34E-007	TMEM222
13	A_37_P376226	4.34E-007	STX10
14	A_37_P177940	4.40E-007	HOMER2
15	A_37_P002367	4.41E-007	ERGIC1
16	A_37_P169867	4.44E-007	MAF1
17	A_37_P081035	5.02E-007	FAM100B
18	A_37_P063199	5.33E-007	ZNF385A
19	A_37_P145214	5.45E-007	EPN1
20	A_37_P276212	5.47E-007	PTPN18
21	A_37_P400400	5.47E-007	HCN4
22	A_37_P078564	5.55E-007	WDR45L
23	A_37_P240648	5.60E-007	DUSP7
24	A_37_P440454	5.83E-007	GAS6
25	A_37_P103607	6.10E-007	AKT1
26	A_37_P104201	6.16E-007	EVL
27	A_37_P074365	6.20E-007	UBL4A
28	A_37_P118238	6.31E-007	KIAA1522
29	A_23_P315286	6.31E-007	C19orf22
30	A_37_P171821	6.39E-007	SORBS3
31	A_37_P300835	6.41E-007	PRAF2
32	A_37_P226623	6.57E-007	MOCS2
33	A_37_P188013	6.78E-007	TMEM189
34	A_37_P064104	6.84E-007	MAPKAPK5
35	A_37_P332938	7.02E-007	ADAM15
36	A_37_P193232	7.02E-007	CCNY
37	A_37_P243259	7.05E-007	HYAL3
38	A_37_P148004	7.08E-007	BTBD2
39	A_37_P162296	7.09E-007	C7orf50
40	A_37_P140053	7.09E-007	RPP21
41	A_37_P187794	7.10E-007	DTD1
42	A_37_P320533	7.14E-007	C9orf21
43	A_37_P369370	7.19E-007	FSTL3
44	A_37_P124250	7.20E-007	SHC1
45	A_37_P196821	7.21E-007	SFXN4
46	A_37_P312364	7.28E-007	FAM195B
47	A_37_P071095	7.28E-007	EMD
48	A_37_P098640	7.30E-007	NELF
49	A_37_P394970	7.33E-007	TMEM66
50	A_37_P149343	7.33E-007	RFX1
51	A_37_P218962	7.46E-007	STK24
52	A_37_P092061	7.46E-007	ST6GALNAC4
53	A_37_P159155	7.51E-007	LIMK1
54	A_37_P010648	7.60E-007	RASSF1
55	A_33_P3344201	7.60E-007	AKT1S1
56	A_37_P031830	7.66E-007	BCKDK
57	A_37_P153321	7.76E-007	FKBP8
58	A_37_P346707	7.81E-007	C1orf85
59	A_37_P074686	7.88E-007	CDK16
60	A_23_P258093	7.91E-007	AGPAT1

Transcript Rank	Probe ID	Adjusted p-value	Gene
3391	A_37_P183173	0.00017305	GRAMD4
3392	A_37_P434120	0.00017365	RRAS2
3393	A_37_P430012	0.00017373	LGALS12
3394	A_37_P036722	0.00017382	MSLN
3395	A_37_P057473	0.00017441	FMNL3
3396	A_37_P086797	0.00017445	RAB37
3397	A_37_P044099	0.00017467	DGKD
3398	A_37_P218480	0.00017484	C13orf18
3399	A_37_P018899	0.00017484	PTPN23
3400	A_37_P131396	0.00017503	MBOAT1
3401	A_37_P113067	0.00017508	CLCNKB
3402	A_23_P142389	0.00017508	LSR
3403	A_24_P916656	0.00017527	CRCP
3404	A_37_P183318	0.00017558	HSCB
3405	A_37_P157616	0.00017588	NSUN5
3406	A_37_P260054	0.00017624	RPL3L
3407	A_23_P17242	0.0001765	ABHD1
3408	A_37_P029128	0.00017667	JAKMIP1
3409	A_37_P006666	0.00017675	PPARGC1B
3410	A_37_P423403	0.00017679	FAM171A1
3411	A_37_P132592	0.00017681	SNX3
3412	A_37_P021782	0.00017721	SEMA5B
3413	A_37_P186773	0.00017736	C20orf152
3414	A_24_P567298	0.00017744	CSAG2
3415	A_37_P068353	0.00017753	TUBA1C
3416	A_37_P080999	0.00017771	MKS1
3417	A_37_P147066	0.00017781	DHPS
3418	A_37_P046426	0.00017798	HOXD4
3419	A_37_P142834	0.000178	EPHX3
3420	A_37_P280573	0.000178	CGREF1
3421	A_37_P023638	0.00017803	CC2D2A
3422	A_37_P032118	0.00017857	CTU2
3423	A_37_P124474	0.00017884	SLC2A7
3424	A_37_P215595	0.00017887	ERG
3425	A_37_P151829	0.0001789	SNRPA
3426	A_37_P269870	0.00017924	HADHB
3427	A_37_P012166	0.00017924	MFI2
3428	A_37_P219629	0.00017936	SERPINE3
3429	A_37_P203181	0.00017941	ALDH3B2
3430	A_37_P119563	0.00017941	BEST4
3431	A_37_P085648	0.00017943	SPDYE4
3432	A_37_P312719	0.00017956	CD300LF
3433	A_37_P425498	0.00017964	ATHL1
3434	A_37_P206415	0.00017998	ZBTB3
3435	A_37_P201306	0.00018004	CLCF1
3436	A_37_P346496	0.00018107	NFASC
3437	A_23_P120435	0.00018127	WFDC3
3438	A_37_P284308	0.00018145	ERBB3
3439	A_37_P285906	0.00018149	PLBD1
3440	A_37_P176136	0.00018151	IQCH
3441	A_37_P129636	0.00018155	TRNP1
3442	A_37_P185441	0.00018165	CARD10
3443	A_37_P101673	0.00018243	RNF165
3444	A_37_P126795	0.00018249	FAF1
3445	A_37_P359876	0.00018261	KIAA0776
3446	A_33_P3416668	0.00018266	VWA1
3447	A_37_P211085	0.00018316	SLC37A2
3448	A_24_P159227	0.00018323	PAK6
3449	A_37_P223948	0.00018345	AHRR
3450	A_37_P420626	0.0001835	SLIT1

61	A_37_P143735	7.96E-007	CCDC106
62	A_37_P346440	7.98E-007	FAM189B
63	A_37_P319366	7.98E-007	NEK6
64	A_37_P189261	8.01E-007	MYBL2
65	A_37_P017915	8.12E-007	IFRD2
66	A_37_P143586	8.16E-007	GAMT
67	A_37_P031547	8.27E-007	ARL2BP
68	A_37_P079510	8.29E-007	CDK3
69	A_37_P033022	8.32E-007	CPNE2
70	A_37_P130199	8.34E-007	USP21
71	A_37_P280832	8.34E-007	RARG
72	A_37_P212736	8.35E-007	FIBP
73	A_37_P224282	8.35E-007	CLPTM1L
74	A_37_P313430	8.35E-007	PEMT
75	A_37_P139421	8.37E-007	SLC35B2
76	A_33_P3297217	8.37E-007	NAAA
77	A_37_P098133	8.37E-007	TESK1
78	A_37_P069386	8.38E-007	ARAF
79	A_37_P096329	8.38E-007	SDCCAG3
80	A_37_P147551	8.38E-007	UBXN6
81	A_37_P155084	8.38E-007	ARF5
82	A_37_P019715	8.45E-007	CAMK1
83	A_37_P319827	8.50E-007	PBX3
84	A_37_P146324	8.50E-007	HMG20B
85	A_37_P032901	8.50E-007	COTL1
86	A_37_P408502	8.50E-007	C22orf9
87	A_37_P123872	8.51E-007	CDK18
88	A_37_P191212	8.51E-007	TCFL5
89	A_37_P142366	8.51E-007	RNF126
90	A_37_P040641	8.60E-007	ABI2
91	A_37_P086851	8.89E-007	PYCR1
92	A_37_P225639	8.89E-007	ANKH
93	A_37_P085761	8.89E-007	POLDIP2
94	A_37_P110189	9.08E-007	PHC2
95	A_37_P105850	9.08E-007	C14orf1
96	A_37_P050852	9.09E-007	NPAS2
97	A_23_P50872	9.09E-007	NDUFB7
98	A_37_P257912	9.09E-007	GLIS2
99	A_37_P091090	9.34E-007	C9orf86
100	A_37_P084342	9.39E-007	MXRA7
101	A_37_P299557	9.51E-007	FAM3A
102	A_37_P283718	9.53E-007	GLTP
103	A_37_P380297	9.62E-007	CPSF4
104	A_37_P185822	9.73E-007	TOMM22
105	A_37_P036709	9.81E-007	MPG
106	A_37_P064058	9.82E-007	LTBR
107	A_37_P121526	9.92E-007	MAPKAPK2
108	A_37_P149808	9.93E-007	TGFB1
109	A_37_P145291	9.93E-007	MKNK2
110	A_37_P206242	1.00E-006	B3GAT3
111	A_37_P125492	1.00E-006	POMGNT1
112	A_37_P343831	1.01E-006	HES4
113	A_37_P121576	1.02E-006	DVL1
114	A_37_P136313	1.02E-006	KLHDC3
115	A_37_P310546	1.02E-006	ETV4
116	A_37_P148343	1.02E-006	LPPR2
117	A_37_P401421	1.02E-006	ULK3
118	A_37_P372029	1.02E-006	SGTA
119	A_37_P281290	1.02E-006	FOXM1
120	A_37_P098459	1.03E-006	FBXW5
121	A_37_P234157	1.03E-006	NME6

3451	A_37_P284676	0.00018357	FICD
3452	A_37_P273856	0.00018363	MRPL33
3453	A_37_P202315	0.00018381	C11orf86
3454	A_37_P118366	0.00018409	MRPL55
3455	A_37_P115489	0.00018438	SIKE1
3456	A_37_P206812	0.00018438	C11orf93
3457	A_37_P023232	0.00018442	TUBB4Q
3458	A_23_P24922	0.0001845	LIPT2
3459	A_37_P139539	0.00018495	C6orf64
3460	A_37_P112551	0.00018513	COL9A2
3461	A_37_P315788	0.00018539	DAB2IP
3462	A_37_P262466	0.00018544	FAM38A
3463	A_37_P173573	0.00018546	RASGRP1
3464	A_37_P164725	0.00018549	KIAA1147
3465	A_37_P008371	0.00018554	THG1L
3466	A_23_P161324	0.00018554	NUDT13
3467	A_37_P177256	0.0001856	HERC2
3468	A_37_P142646	0.0001856	KLK5
3469	A_37_P008033	0.00018566	SPINK5
3470	A_37_P147005	0.00018582	KLF2
3471	A_37_P192576	0.00018587	BTBD16
3472	A_37_P203115	0.00018642	CNIH2
3473	A_37_P173478	0.00018647	ARID3B
3474	A_37_P138518	0.00018675	OGFRL1
3475	A_32_P86739	0.00018722	C10orf114
3476	A_37_P136041	0.00018732	ITPR3
3477	A_37_P195743	0.00018778	INPP5F
3478	A_37_P345240	0.00018778	MARK1
3479	A_37_P142516	0.00018865	RASAL3
3480	A_37_P242249	0.00018892	C3orf67
3481	A_37_P096941	0.0001891	C9orf68
3482	A_33_P3278941	0.00018912	REC8
3483	A_37_P044530	0.00018929	DYNC2LI1
3484	A_37_P431710	0.00018936	HSPA8
3485	A_37_P019130	0.00018943	IQCB1
3486	A_37_P417237	0.00018994	ALDH18A1
3487	A_37_P256849	0.00019005	CTRB1
3488	A_37_P146105	0.00019051	GTPBP3
3489	A_33_P3423027	0.00019092	NRL
3490	A_37_P361125	0.00019093	HLA-DOA
3491	A_37_P200020	0.00019098	SUV39H2
3492	A_37_P051017	0.00019108	PTPRN
3493	A_37_P044635	0.00019135	EFR3B
3494	A_33_P3217704	0.00019144	KIAA1539
3495	A_37_P071619	0.00019148	GAGE10
3496	A_37_P409088	0.00019148	SGSM1
3497	A_37_P018826	0.00019283	PRKCD
3498	A_37_P297680	0.00019291	HS6ST2
3499	A_33_P3288754	0.00019366	C19orf48
3500	A_37_P179957	0.00019384	SLC28A1
3501	A_37_P231600	0.00019398	SLC6A7
3502	A_37_P123072	0.00019402	C1orf87
3503	A_37_P299698	0.00019409	FAM70A
3504	A_23_P106898	0.00019456	ORAI3
3505	A_37_P131942	0.00019457	TEAD3
3506	A_37_P070510	0.00019459	PRKX
3507	A_37_P257957	0.00019468	GPR114
3508	A_37_P062660	0.00019495	DHX37
3509	A_37_P144176	0.0001952	CLEC17A
3510	A_37_P154521	0.00019525	ACCN3
3511	A_37_P140879	0.000196	KCNK16

122	A_37_P016927	1.03E-006	MAPKAPK3
123	A_37_P160952	1.03E-006	MEST
124	A_37_P371931	1.03E-006	UBE2S
125	A_37_P081859	1.03E-006	GPS2
126	A_37_P133599	1.04E-006	CNPY3
127	A_37_P372926	1.04E-006	C19orf6
128	A_37_P185897	1.04E-006	TSPO
129	A_37_P233365	1.04E-006	RYK
130	A_37_P001811	1.06E-006	MGAT4B
131	A_37_P015575	1.09E-006	BAP1
132	A_23_P68628	1.09E-006	NECAB3
133	A_37_P217386	1.10E-006	FARP1
134	A_37_P076875	1.11E-006	WDR13
135	A_37_P289136	1.11E-006	PGAM5
136	A_37_P237574	1.11E-006	NT5DC2
137	A_37_P146286	1.12E-006	GSK3A
138	A_37_P201660	1.12E-006	ARL2
139	A_37_P012852	1.14E-006	TIMP4
140	A_37_P215610	1.14E-006	PRMT2
141	A_37_P144681	1.16E-006	DAZAP1
142	A_37_P004430	1.17E-006	TNIP1
143	A_37_P118738	1.17E-006	B4GALT3
144	A_37_P134441	1.17E-006	NRM
145	A_37_P154497	1.17E-006	RARRES2
146	A_37_P150122	1.17E-006	PIN1
147	A_37_P126855	1.17E-006	RUSC1
148	A_37_P398777	1.17E-006	ANXA2
149	A_33_P3243264	1.18E-006	DNLZ
150	A_37_P151635	1.18E-006	PCSK4
151	A_37_P087728	1.18E-006	SLC25A10
152	A_37_P080424	1.19E-006	TAX1BP3
153	A_37_P118406	1.19E-006	MRPL9
154	A_37_P231805	1.19E-006	SRD5A1
155	A_37_P435729	1.19E-006	TMEM179B
156	A_23_P54728	1.19E-006	FAM173A
157	A_37_P091528	1.20E-006	CERCAM
158	A_37_P419440	1.20E-006	INPP5A
159	A_37_P381586	1.22E-006	GALNT11
160	A_37_P037239	1.22E-006	NUTF2
161	A_37_P388684	1.23E-006	FIS1
162	A_37_P091341	1.23E-006	AGPAT2
163	A_37_P413177	1.23E-006	NPEPL1
164	A_37_P201267	1.23E-006	ALDH3B1
165	A_37_P142960	1.24E-006	TEAD2
166	A_37_P036448	1.25E-006	METTL9
167	A_37_P428399	1.28E-006	ARFIP2
168	A_37_P148901	1.28E-006	MRPS12
169	A_37_P184677	1.29E-006	C22orf13
170	A_37_P034686	1.29E-006	INO80E
171	A_37_P095011	1.29E-006	PTGES2
172	A_37_P178052	1.29E-006	MAP2K5
173	A_37_P130242	1.30E-006	VAMP3
174	A_37_P114630	1.31E-006	DPH2
175	A_37_P072355	1.32E-006	GTPBP6
176	A_37_P146280	1.32E-006	RDH13
177	A_37_P302578	1.32E-006	C17orf81
178	A_37_P376554	1.33E-006	MAP2K2
179	A_37_P085124	1.33E-006	RFNG
180	A_37_P123226	1.33E-006	SPOCD1
181	A_23_P15073	1.33E-006	JMJD8
182	A_37_P221290	1.33E-006	BCAS4
3512	A_37_P167859	0.00019616	SLC20A2
3513	A_37_P055221	0.00019617	TCF7L1
3514	A_37_P425587	0.0001963	ATP5L
3515	A_37_P051130	0.00019641	ENST00000475027
3516	A_37_P327058	0.0001966	ADCY4
3517	A_37_P203207	0.00019705	ROBO4
3518	A_37_P242071	0.00019736	C3orf22
3519	A_37_P197296	0.00019788	C10orf72
3520	A_37_P266665	0.00019793	ST3GAL5
3521	A_37_P177928	0.00019851	LRRK1
3522	A_37_P052343	0.00019862	PROM2
3523	A_37_P028600	0.00019893	RASL11B
3524	A_37_P021980	0.00019903	ZBTB47
3525	A_37_P143650	0.0001991	COL5A3
3526	A_37_P154387	0.00019949	ABCA13
3527	A_37_P107212	0.00019957	IPO4
3528	A_37_P098425	0.00019959	FBXO10
3529	A_37_P342239	0.00019961	MXRA8
3530	A_37_P198419	0.0002	CDHR1
3531	A_37_P311481	0.00020022	FAM171A2
3532	A_33_P3276693	0.00020023	PGF
3533	A_37_P422553	0.00020063	PNLIPRP1
3534	A_37_P214370	0.00020087	SLC19A1
3535	A_37_P349513	0.00020101	RABGGTB
3536	A_24_P7584	0.00020149	LY6G5C
3537	A_37_P111502	0.00020163	C1orf93
3538	A_37_P088296	0.00020163	CCDC57
3539	A_37_P347347	0.00020178	PAFAH2
3540	A_37_P328951	0.00020181	ZBTB25
3541	A_37_P284564	0.00020187	FBRSL1
3542	A_37_P069883	0.00020245	PHKA2
3543	A_37_P024034	0.00020261	LEF1
3544	A_37_P090758	0.00020281	CNTFR
3545	A_37_P433674	0.00020292	RAD9A
3546	A_37_P186891	0.00020319	TMEM90B
3547	A_37_P412990	0.00020343	MYH7B
3548	A_37_P204626	0.00020347	FDX1
3549	A_37_P313717	0.00020367	ZSWIM7
3550	A_37_P111077	0.0002038	C1orf113
3551	A_37_P057516	0.00020386	ABCB9
3552	A_37_P332577	0.00020387	CFL2
3553	A_23_P330461	0.00020403	TMC4
3554	A_37_P180120	0.00020435	SNRPN
3555	A_24_P67585	0.00020463	VGLL3
3556	A_37_P436307	0.00020522	USP35
3557	A_37_P368535	0.00020522	DKKL1
3558	A_37_P036603	0.00020525	C16orf79
3559	A_37_P037215	0.00020556	NUDT7
3560	A_37_P214756	0.00020562	MRPL39
3561	A_37_P058700	0.00020585	KRT78
3562	A_23_P304386	0.00020588	HIGD2A
3563	A_37_P042439	0.00020625	C2orf85
3564	A_37_P065457	0.00020644	SPSB2
3565	A_37_P008427	0.00020648	TRIM36
3566	A_37_P189548	0.00020691	C20orf132
3567	A_24_P363100	0.00020707	RGMB
3568	A_33_P3342653	0.00020721	ADAM33
3569	A_37_P168561	0.00020725	IKBKB
3570	A_37_P212418	0.00020729	SIK3
3571	A_37_P196817	0.00020747	SFTPD
3572	A_37_P410739	0.00020751	ADRA1D

183	A_37_P256648	1.33E-006	ROGDI
184	A_37_P133881	1.34E-006	SERPINB6
185	A_37_P328321	1.34E-006	NUDT14
186	A_37_P060260	1.34E-006	DTX3
187	A_37_P375755	1.34E-006	SSBP4
188	A_37_P349199	1.35E-006	NADK
189	A_37_P313005	1.35E-006	SNF8
190	A_37_P066095	1.36E-006	PXN
191	A_37_P210418	1.37E-006	RPS6KA4
192	A_37_P152278	1.38E-006	TBC1D17
193	A_37_P039009	1.38E-006	STUB1
194	A_37_P070180	1.38E-006	CD99
195	A_37_P362929	1.38E-006	C6orf145
196	A_37_P150126	1.38E-006	PKN1
197	A_37_P443533	1.38E-006	TFDP1
198	A_37_P168508	1.38E-006	HSF1
199	A_23_P101342	1.38E-006	ATG4D
200	A_37_P164736	1.40E-006	CDK5
201	A_37_P264138	1.40E-006	ZNRF1
202	A_33_P3324909	1.40E-006	JUND
203	A_37_P162052	1.40E-006	IMPDH1
204	A_37_P096457	1.40E-006	POLR1E
205	A_37_P073236	1.40E-006	TFE3
206	A_37_P170491	1.41E-006	SHARPIN
207	A_37_P291261	1.41E-006	STRAP
208	A_37_P185666	1.41E-006	SYNGR1
209	A_37_P105896	1.41E-006	C14orf126
210	A_37_P181978	1.41E-006	CDC42EP1
211	A_37_P201173	1.41E-006	ADRBK1
212	A_37_P408449	1.42E-006	PARVB
213	A_37_P228059	1.42E-006	HDAC3
214	A_37_P111857	1.42E-006	ADIPOR1
215	A_37_P384233	1.43E-006	POLR2J2
216	A_37_P371484	1.43E-006	SLC25A23
217	A_37_P243524	1.43E-006	SELT
218	A_37_P187900	1.43E-006	ENTPD6
219	A_37_P347063	1.43E-006	PABPC4
220	A_37_P190330	1.45E-006	SLC35C2
221	A_37_P404299	1.45E-006	TMED3
222	A_37_P045962	1.45E-006	GPN1
223	A_37_P116810	1.45E-006	HAX1
224	A_37_P197550	1.45E-006	PTPLA
225	A_37_P197877	1.46E-006	MSRB2
226	A_37_P315602	1.46E-006	COQ4
227	A_37_P336840	1.48E-006	CMPK1
228	A_37_P143395	1.50E-006	C19orf60
229	A_37_P168749	1.50E-006	DGAT1
230	A_32_P115130	1.50E-006	MRPL41
231	A_37_P155007	1.50E-006	AP1S1
232	A_37_P039998	1.50E-006	NETO2
233	A_37_P078047	1.52E-006	ARRB2
234	A_24_P268015	1.52E-006	MEAF6
235	A_37_P157035	1.54E-006	GNA12
236	A_37_P037916	1.54E-006	RAB11FIP3
237	A_37_P360288	1.54E-006	BAK1
238	A_37_P212658	1.56E-006	VEGFB
239	A_37_P087507	1.58E-006	SENP3
240	A_37_P128188	1.58E-006	SNX7
241	A_37_P017903	1.59E-006	NUDT16
242	A_37_P039140	1.60E-006	SYT17
243	A_37_P207063	1.62E-006	BSCL2
3573	A_37_P153576	0.00020757	ZNF317
3574	A_23_P48826	0.00020851	TRIM69
3575	A_23_P109171	0.00020863	BFSP1
3576	A_24_P703830	0.00020895	NANOS3
3577	A_37_P158953	0.00020906	KLHL7
3578	A_37_P252234	0.00020932	EVC2
3579	A_37_P433101	0.00020984	IGSF9B
3580	A_37_P347369	0.00020985	PADI3
3581	A_37_P049096	0.0002101	XDH
3582	A_37_P322410	0.00021064	USP20
3583	A_37_P338603	0.0002109	EIF2C4
3584	A_37_P336820	0.0002112	LAPTM5
3585	A_23_P115022	0.00021134	TMEM125
3586	A_37_P097562	0.00021155	FAM166B
3587	A_24_P358591	0.00021234	C2orf70
3588	A_37_P259730	0.00021244	MVD
3589	A_37_P168644	0.00021244	PLAT
3590	A_37_P038782	0.00021249	CCDC102A
3591	A_37_P193555	0.00021255	COL13A1
3592	A_37_P248380	0.00021279	LNX1
3593	A_37_P036285	0.00021313	MAPK8IP3
3594	A_37_P143909	0.00021347	CD97
3595	A_37_P349644	0.00021349	RAVER2
3596	A_37_P117456	0.00021376	IFI44
3597	A_37_P029385	0.00021419	SORCS2
3598	A_37_P026075	0.00021434	KIAA1530
3599	A_37_P184765	0.00021497	IGLL1
3600	A_37_P103577	0.00021497	GCH1
3601	A_37_P211631	0.00021521	SYT12
3602	A_37_P142363	0.00021528	SEMA6B
3603	A_37_P162184	0.00021533	C7orf31
3604	A_37_P022662	0.00021535	ANXA10
3605	A_37_P074046	0.00021541	EDA2R
3606	A_37_P137402	0.00021584	HLA-DMA
3607	A_37_P189760	0.00021599	SDC4
3608	A_37_P125692	0.00021635	PVRL4
3609	A_37_P040348	0.00021639	ZNF205
3610	A_37_P131858	0.00021662	ARMC2
3611	A_37_P366108	0.00021681	ACPT
3612	A_37_P375180	0.00021687	CAPN12
3613	A_37_P191894	0.00021808	BC127746
3614	A_37_P115303	0.00021811	GOLT1A
3615	A_37_P323745	0.00021846	ELAC1
3616	A_37_P037088	0.0002185	NLRC5
3617	A_37_P141078	0.00021859	TCF19
3618	A_37_P115770	0.00021875	MMEL1
3619	A_37_P186737	0.00021878	SLC4A11
3620	A_37_P199149	0.00021898	RET
3621	A_37_P007856	0.00021985	SLC6A19
3622	A_37_P106769	0.00022059	PCK2
3623	A_37_P012206	0.00022078	CRELD1
3624	A_37_P187895	0.00022085	ZNF343
3625	A_23_P139648	0.00022092	IAPP
3626	A_37_P194169	0.00022104	NRAP
3627	A_37_P010895	0.00022115	SLC12A8
3628	A_37_P375080	0.00022115	SCAF1
3629	A_33_P3349637	0.00022115	PCDH1
3630	A_37_P117842	0.00022119	ITGA10
3631	A_24_P401870	0.00022137	C9orf139
3632	A_37_P032267	0.00022152	CA7
3633	A_37_P166051	0.00022203	BNIP3L

244	A_37_P359351	1.63E-006	HMGA1
245	A_37_P085261	1.63E-006	MRPL12
246	A_33_P3286616	1.63E-006	IRAK1
247	A_37_P161936	1.64E-006	EPHB4
248	A_37_P330024	1.64E-006	MTA1
249	A_37_P092949	1.64E-006	WDR34
250	A_37_P211453	1.67E-006	ST3GAL4
251	A_37_P202929	1.67E-006	NOX4
252	A_37_P084726	1.67E-006	ORMDL3
253	A_37_P107590	1.67E-006	ITPK1
254	A_37_P040555	1.67E-006	CHN1
255	A_37_P100242	1.68E-006	IMPA2
256	A_37_P427971	1.68E-006	ESRRA
257	A_37_P115419	1.68E-006	FAM54B
258	A_37_P002919	1.72E-006	NUDCD2
259	A_37_P327686	1.72E-006	TINF2
260	A_37_P098264	1.72E-006	SIGMAR1
261	A_37_P329133	1.73E-006	BEGAIN
262	A_37_P001349	1.73E-006	AGXT2L2
263	A_37_P210377	1.74E-006	RPL27A
264	A_37_P208851	1.74E-006	NUDT22
265	A_37_P366008	1.75E-006	MBD3
266	A_37_P202999	1.75E-006	COMMD9
267	A_37_P144264	1.76E-006	AKT2
268	A_37_P090993	1.76E-006	C9orf142
269	A_33_P3267280	1.76E-006	CTDSP2
270	A_37_P253837	1.77E-006	SEL1L3
271	A_37_P388322	1.77E-006	TTYH3
272	A_37_P131082	1.77E-006	ZNF593
273	A_37_P436685	1.79E-006	PEX16
274	A_37_P072691	1.80E-006	DHRSX
275	A_23_P30464	1.80E-006	PRR7
276	A_37_P118466	1.80E-006	AURKAIP1
277	A_37_P316752	1.80E-006	FXN
278	A_37_P265986	1.80E-006	C2orf28
279	A_37_P145386	1.81E-006	FAM125A
280	A_37_P231109	1.81E-006	RAB24
281	A_37_P142825	1.81E-006	ABHD8
282	A_23_P10518	1.83E-006	TFDP3
283	A_37_P043745	1.84E-006	CTDSP1
284	A_37_P165793	1.84E-006	FOXH1
285	A_23_P416686	1.84E-006	GPR137
286	A_37_P398028	1.84E-006	CHP
287	A_24_P737939	1.84E-006	C6orf154
288	A_37_P432303	1.84E-006	POLD4
289	A_37_P018328	1.84E-006	PIGX
290	A_37_P114820	1.85E-006	EFHD2
291	A_37_P258118	1.85E-006	HAGHL
292	A_37_P391928	1.85E-006	GRINA
293	A_37_P443386	1.86E-006	CARS2
294	A_37_P032863	1.86E-006	CMTM3
295	A_37_P094135	1.86E-006	DFNB31
296	A_37_P386426	1.86E-006	TMED4
297	A_37_P203698	1.87E-006	DGKZ
298	A_37_P084466	1.87E-006	TRIM16L
299	A_37_P072174	1.87E-006	HPRT1
300	A_37_P159115	1.87E-006	LFNG
301	A_23_P149259	1.89E-006	TMEM79
302	A_37_P095409	1.89E-006	MRPS2
303	A_37_P295700	1.89E-006	ASB9
304	A_37_P191008	1.89E-006	TCEA2

3634	A_37_P216988	0.00022208	COL4A2
3635	A_37_P141711	0.00022218	UHRF1BP1
3636	A_37_P239655	0.00022234	BCL6
3637	A_37_P296033	0.00022236	NUP62CL
3638	A_33_P3285456	0.00022293	C1orf68
3639	A_37_P098543	0.00022313	SEMA4D
3640	A_23_P67367	0.00022321	DHDH
3641	A_23_P31224	0.00022355	PILRA
3642	A_37_P281198	0.00022387	AMDHD1
3643	A_37_P377064	0.00022419	TMEM143
3644	A_37_P084076	0.00022421	HES7
3645	A_37_P103175	0.00022421	C14orf28
3646	A_37_P304066	0.00022425	DHRS7B
3647	A_37_P306799	0.0002243	LLGL1
3648	A_37_P075931	0.00022467	SSR4
3649	A_37_P180018	0.00022495	CATSPER2
3650	A_37_P080003	0.00022533	GGT6
3651	A_37_P021145	0.00022595	TMEM45A
3652	A_37_P103093	0.0002261	C14orf159
3653	A_37_P301091	0.0002261	SSH2
3654	A_37_P150632	0.00022627	PRR19
3655	A_37_P017361	0.0002264	MYD88
3656	A_37_P151567	0.00022671	SIN3B
3657	A_37_P152388	0.0002269	NDUFA7
3658	A_24_P418408	0.00022731	FAM89A
3659	A_37_P308818	0.00022738	RFFL
3660	A_37_P091443	0.00022754	CENPP
3661	A_37_P120862	0.00022786	MYBPH
3662	A_37_P308959	0.00022797	MRC2
3663	A_37_P011811	0.00022849	CLDN16
3664	A_37_P112336	0.00022857	CD53
3665	A_37_P156564	0.00022893	CPA4
3666	A_37_P103657	0.00022949	SERPINA9
3667	A_37_P230211	0.00023011	PLEKHG4B
3668	A_37_P218521	0.00023079	C13orf38
3669	A_37_P043089	0.00023083	TMEM17
3670	A_37_P295201	0.0002309	UXT
3671	A_37_P410332	0.00023094	BPIL1
3672	A_37_P198364	0.00023124	PAPSS2
3673	A_37_P243079	0.0002313	RNF123
3674	A_37_P376021	0.00023158	UNC13A
3675	A_37_P088281	0.00023189	KCNH4
3676	A_37_P187712	0.00023217	DNAJC5
3677	A_37_P083018	0.00023231	DHRS7C
3678	A_37_P199571	0.00023258	SFXN2
3679	A_23_P94128	0.00023259	NEIL2
3680	A_37_P305937	0.00023272	MRPL45
3681	A_37_P095441	0.00023333	PNPLA7
3682	A_37_P429030	0.0002338	DCHS1
3683	A_37_P128060	0.0002346	CC2D1B
3684	A_37_P103482	0.00023618	TEP1
3685	A_37_P067850	0.00023669	NDUFA4L2
3686	A_37_P365911	0.00023679	PEX6
3687	A_37_P350295	0.00023717	RPL11
3688	A_37_P059007	0.00023721	AGAP2
3689	A_37_P338417	0.0002373	DUSP27
3690	A_37_P146903	0.00023743	KIAA1543
3691	A_37_P280026	0.00023774	SLC40A1
3692	A_37_P166353	0.00023784	CA3
3693	A_37_P021452	0.00023786	THRB
3694	A_37_P348725	0.00023809	PQLC2

305	A_37_P409795	1.90E-006	MAPK11
306	A_37_P194417	1.90E-006	FAM149B1
307	A_37_P224300	1.90E-006	CLTB
308	A_37_P213876	1.90E-006	AGPAT3
309	A_37_P088146	1.90E-006	STARD3
310	A_37_P143133	1.90E-006	BCL3
311	A_37_P046442	1.91E-006	HPCAL1
312	A_37_P294004	1.91E-006	TIMM17B
313	A_23_P26439	1.92E-006	DBNDD1
314	A_37_P163734	1.92E-006	ST7
315	A_23_P20107	1.92E-006	GSTK1
316	A_37_P046747	1.93E-006	INPP1
317	A_23_P57474	1.93E-006	OSBP2
318	A_37_P191022	1.94E-006	TGIF2-C20ORF24
319	A_37_P126274	1.94E-006	RRAGC
320	A_37_P374829	1.95E-006	ISOC2
321	A_37_P336953	1.95E-006	LASS2
322	A_37_P265788	1.95E-006	ACTR1B
323	A_37_P091157	1.96E-006	C9orf96
324	A_37_P213015	1.96E-006	CHID1
325	A_37_P050868	1.96E-006	TMBIM1
326	A_37_P189192	1.97E-006	E2F1
327	A_37_P366834	1.97E-006	BCL2L12
328	A_37_P099384	1.97E-006	RBFA
329	A_33_P3209591	1.97E-006	AQP3
330	A_37_P410100	1.98E-006	ABHD12
331	A_37_P033501	1.98E-006	E2F4
332	A_37_P151063	1.99E-006	TMED1
333	A_37_P185539	1.99E-006	JOSD1
334	A_37_P044626	1.99E-006	EFHD1
335	A_37_P039606	1.99E-006	TRAPPC2L
336	A_37_P078903	2.00E-006	GABARAP
337	A_37_P407149	2.02E-006	KCTD17
338	A_37_P039600	2.02E-006	TRAF7
339	A_37_P087875	2.02E-006	LSM12
340	A_37_P121491	2.02E-006	HPCAL4
341	A_37_P151436	2.03E-006	PPAP2C
342	A_37_P141102	2.03E-006	CCNC
343	A_37_P074451	2.03E-006	ELF4
344	A_37_P097912	2.04E-006	SSNA1
345	A_37_P057719	2.04E-006	CLEC4E
346	A_37_P407123	2.05E-006	SLC25A1
347	A_37_P416465	2.07E-006	C10orf26
348	A_37_P067758	2.08E-006	TCTN1
349	A_37_P159542	2.09E-006	BCL7B
350	A_37_P050919	2.10E-006	STK25
351	A_37_P032026	2.10E-006	C16orf57
352	A_37_P075588	2.11E-006	FAM127B
353	A_37_P189631	2.11E-006	C20orf27
354	A_37_P219883	2.13E-006	TMCO3
355	A_37_P091328	2.13E-006	GBA2
356	A_37_P080746	2.13E-006	MIF4GD
357	A_37_P362555	2.13E-006	PHF1
358	A_37_P170055	2.15E-006	MRPL15
359	A_37_P094459	2.16E-006	WDR85
360	A_37_P267654	2.16E-006	MGAT4A
361	A_37_P087044	2.17E-006	RHOT1
362	A_37_P033829	2.19E-006	APRT
363	A_37_P009693	2.19E-006	ABHD14A
364	A_37_P032804	2.19E-006	GDE1
365	A_37_P096180	2.20E-006	SHB

3695	A_37_P235362	0.00023855	UBA7
3696	A_37_P075723	0.00023859	SLC25A5
3697	A_37_P142284	0.00023859	PGLYRP2
3698	A_37_P089413	0.00023883	UNC45B
3699	A_37_P093324	0.00023886	GRIN1
3700	A_37_P385533	0.00023886	PIK3CG
3701	A_37_P120625	0.00023929	DPH5
3702	A_37_P190413	0.00023932	RPS21
3703	A_37_P423532	0.00023982	SNCG
3704	A_37_P293449	0.00024008	FUNDC1
3705	A_33_P3397288	0.00024028	EDN3
3706	A_37_P003713	0.00024093	IRX1
3707	A_37_P344127	0.00024118	PTCH2
3708	A_33_P3272698	0.00024118	DUSP23
3709	A_37_P179076	0.0002416	PML
3710	A_37_P154274	0.0002416	ZNF836
3711	A_37_P140640	0.00024183	LTB
3712	A_37_P149128	0.00024191	C19orf46
3713	A_37_P000885	0.00024209	ADAMTS12
3714	A_37_P437371	0.00024235	KDM5D
3715	A_37_P118470	0.00024237	KLHL17
3716	A_37_P087340	0.0002426	RSAD1
3717	A_37_P335112	0.00024303	C1orf69
3718	A_37_P071367	0.0002433	CXorf36
3719	A_37_P235031	0.0002433	CASR
3720	A_37_P131427	0.00024366	KIF6
3721	A_37_P146131	0.00024426	GZMM
3722	A_23_P4899	0.00024426	NTF4
3723	A_23_P424582	0.00024455	EGFL8
3724	A_37_P011961	0.00024457	ALDH1L1
3725	A_33_P3254216	0.00024478	MAPK10
3726	A_23_P103104	0.00024537	MFNG
3727	A_37_P179972	0.00024556	SLC28A2
3728	A_37_P205005	0.0002457	SLC22A24
3729	A_37_P232446	0.00024591	PDGFRB
3730	A_37_P170981	0.00024601	PROSC
3731	A_37_P100352	0.00024606	KIAA0802
3732	A_37_P322016	0.00024614	FBP1
3733	A_37_P015320	0.00024614	SLCO2A1
3734	A_37_P237402	0.00024615	FLNB
3735	A_23_P143006	0.00024617	PRLH
3736	A_37_P228455	0.00024648	DND1
3737	A_37_P103663	0.00024648	CMTM5
3738	A_37_P208570	0.00024679	NCAM1
3739	A_37_P214911	0.00024713	MAFIP
3740	A_37_P054981	0.0002473	RTKN
3741	A_37_P261556	0.0002475	ERN2
3742	A_37_P028257	0.00024815	C4orf3
3743	A_23_P102117	0.00024816	WNT10A
3744	A_37_P175247	0.0002483	FAM154B
3745	A_37_P183898	0.00024861	RPL3
3746	A_37_P169418	0.0002488	MTSS1
3747	A_37_P187280	0.00024891	CEP250
3748	A_37_P078089	0.00024915	PHF12
3749	A_37_P280928	0.00024915	ACADS
3750	A_37_P103750	0.00024925	CPNE6
3751	A_37_P168191	0.00024956	GPT
3752	A_37_P365466	0.00025045	MAP3K7
3753	A_37_P397644	0.00025153	C15orf61
3754	A_37_P035023	0.00025254	TOX3
3755	A_37_P342554	0.00025259	LHX9

366	A_37_P372266	2.21E-006	MARK4
367	A_37_P059917	2.22E-006	DERA
368	A_37_P069207	2.23E-006	SLC10A3
369	A_37_P020664	2.23E-006	STAC
370	A_37_P097596	2.23E-006	SLC2A8
371	A_23_P75800	2.23E-006	RAB3IL1
372	A_37_P318726	2.23E-006	DPM2
373	A_37_P250681	2.24E-006	MXD4
374	A_37_P379771	2.24E-006	CCM2
375	A_37_P330949	2.25E-006	SDR39U1
376	A_37_P013811	2.25E-006	TPRA1
377	A_37_P189646	2.26E-006	C20orf30
378	A_37_P091930	2.26E-006	CREB3
379	A_37_P006125	2.26E-006	TBC1D9B
380	A_37_P379575	2.27E-006	SNX8
381	A_37_P057904	2.27E-006	ARL6IP4
382	A_37_P125350	2.28E-006	PRUNE
383	A_23_P50775	2.28E-006	LRFN3
384	A_37_P013092	2.28E-006	EIF4A2
385	A_37_P116866	2.30E-006	HCN3
386	A_37_P095024	2.31E-006	DPP7
387	A_37_P079255	2.31E-006	RNFT1
388	A_37_P205561	2.31E-006	HTATIP2
389	A_32_P150391	2.31E-006	C17orf100
390	A_37_P379345	2.31E-006	C7orf26
391	A_37_P256745	2.31E-006	CPNE7
392	A_33_P3385002	2.31E-006	ELK1
393	A_37_P120258	2.31E-006	OPN3
394	A_37_P129780	2.31E-006	SRM
395	A_24_P307869	2.31E-006	LLGL2
396	A_37_P147599	2.31E-006	DMWD
397	A_37_P121096	2.31E-006	ORC1
398	A_37_P215019	2.31E-006	DONSON
399	A_37_P097414	2.32E-006	UCK1
400	A_37_P018748	2.32E-006	PPM1M
401	A_37_P281768	2.32E-006	MCRS1
402	A_37_P024438	2.32E-006	CTBP1
403	A_37_P129188	2.32E-006	TMEM183A
404	A_37_P075706	2.34E-006	SLC25A14
405	A_37_P128128	2.34E-006	SMYD2
406	A_23_P370625	2.34E-006	SEPN1
407	A_37_P201384	2.35E-006	ANKRD13D
408	A_37_P263949	2.37E-006	MAPK3
409	A_37_P087668	2.37E-006	RAB34
410	A_37_P372566	2.38E-006	MPND
411	A_37_P037205	2.38E-006	NUBP2
412	A_24_P284523	2.39E-006	MAP3K10
413	A_37_P088047	2.39E-006	SPATA20
414	A_37_P015083	2.39E-006	KCTD6
415	A_37_P184325	2.39E-006	MTFP1
416	A_37_P031603	2.39E-006	ATMIN
417	A_37_P038092	2.39E-006	RPUSD1
418	A_37_P161288	2.42E-006	EIF2AK1
419	A_37_P149069	2.42E-006	C19orf28
420	A_37_P038511	2.44E-006	CARHSP1
421	A_37_P407358	2.46E-006	HDAC10
422	A_37_P042929	2.46E-006	CCNYL1
423	A_37_P197168	2.49E-006	C10orf125
424	A_37_P261066	2.49E-006	PAQR4
425	A_37_P014746	2.49E-006	ATP13A5
426	A_33_P3417695	2.50E-006	ODF3B

3756	A_37_P425079	0.00025291	FOSL1
3757	A_37_P173249	0.0002532	ADAMTSL3
3758	A_37_P390955	0.00025329	CSMD3
3759	A_33_P3221090	0.00025335	GPR156
3760	A_37_P096763	0.00025353	RALGPS1
3761	A_37_P149923	0.00025364	RGL3
3762	A_37_P117959	0.00025383	KCNC4
3763	A_37_P291895	0.00025396	STX2
3764	A_37_P226179	0.00025418	FAM105A
3765	A_37_P185490	0.00025438	RHBDD3
3766	A_37_P263359	0.0002546	SLC38A8
3767	A_37_P001228	0.00025464	CATSPER3
3768	A_37_P077773	0.00025549	NLRP1
3769	A_37_P091760	0.00025569	COL15A1
3770	A_33_P3336720	0.00025599	HAMP
3771	A_23_P432573	0.00025631	MRGPRF
3772	A_23_P45560	0.00025652	GPR143
3773	A_37_P148364	0.00025659	HNRNPL
3774	A_37_P210874	0.00025671	SLC22A11
3775	A_37_P314739	0.00025685	C9orf30
3776	A_37_P064681	0.00025685	RPLP0
3777	A_37_P155292	0.00025717	NOD1
3778	A_37_P017439	0.00025724	ZDHHC19
3779	A_37_P029955	0.00025731	TMEM175
3780	A_37_P406997	0.00025739	PLA2G3
3781	A_37_P118824	0.00025772	LGR6
3782	A_37_P314049	0.00025791	KLF4
3783	A_33_P3393801	0.00025809	PDZK1IP1
3784	A_37_P144129	0.00025875	CIC
3785	A_37_P143892	0.00025899	CD37
3786	A_37_P155766	0.00025924	C7orf65
3787	A_37_P394505	0.00025967	POLR3D
3788	A_37_P217875	0.00026048	ATP7B
3789	A_37_P161749	0.00026113	POLR2J
3790	A_37_P099377	0.00026138	C18orf21
3791	A_37_P107647	0.00026169	SIX6
3792	A_37_P067147	0.00026173	FAM19A2
3793	A_37_P277997	0.00026227	CCDC108
3794	A_33_P3238671	0.00026241	C20orf196
3795	A_37_P355737	0.00026253	BAI3
3796	A_23_P162547	0.00026261	MYL2
3797	A_37_P109222	0.00026275	PGLYRP4
3798	A_37_P111214	0.00026325	C1orf175
3799	A_37_P181267	0.00026336	ZNF592
3800	A_37_P432486	0.00026349	NPAS4
3801	A_37_P430492	0.00026456	RELA
3802	A_37_P249558	0.00026518	ATP5I
3803	A_37_P118274	0.00026537	KIAA1614
3804	A_37_P100736	0.00026547	HSBP1L1
3805	A_37_P191817	0.0002655	ADAMTS14
3806	A_37_P276300	0.0002655	PASK
3807	A_37_P124779	0.00026563	MYOM3
3808	A_37_P139748	0.0002658	POPDC3
3809	A_37_P027406	0.00026639	MND1
3810	A_37_P055747	0.0002665	TRIB2
3811	A_37_P339892	0.00026659	TSPAN2
3812	A_37_P268099	0.00026692	DOK1
3813	A_37_P100150	0.00026738	CYB5A
3814	A_37_P248033	0.00026741	GNRHR
3815	A_37_P114422	0.00026768	SLC35E2
3816	A_37_P098381	0.00026806	CDC14B

427	A_37_P012959	2.50E-006	ECE2
428	A_37_P020936	2.51E-006	TEX264
429	A_37_P079631	2.53E-006	COPS3
430	A_37_P368210	2.53E-006	CSNK1G2
431	A_37_P198329	2.54E-006	STK32C
432	A_37_P097396	2.55E-006	FAM102A
433	A_37_P182281	2.55E-006	TMEM184B
434	A_23_P105545	2.57E-006	VAMP1
435	A_37_P226293	2.59E-006	PITX1
436	A_37_P152779	2.60E-006	MAN2B1
437	A_37_P112769	2.61E-006	CFHR5
438	A_37_P099010	2.62E-006	MAPKAP1
439	A_37_P147910	2.63E-006	PLEKHJ1
440	A_37_P086867	2.63E-006	RAMP2
441	A_37_P185873	2.63E-006	TRIOBP
442	A_37_P314014	2.66E-006	CIZ1
443	A_37_P182858	2.67E-006	FBXO7
444	A_37_P321790	2.67E-006	FANCG
445	A_37_P029637	2.67E-006	TBC1D1
446	A_37_P094243	2.67E-006	ZMYND19
447	A_37_P154619	2.67E-006	ADCK2
448	A_33_P3316026	2.68E-006	USF2
449	A_37_P208089	2.68E-006	MRPL23
450	A_37_P097483	2.68E-006	LRRC26
451	A_37_P017966	2.68E-006	IFT57
452	A_37_P059752	2.69E-006	ANAPC5
453	A_37_P143621	2.70E-006	AES
454	A_37_P127530	2.70E-006	SHISA4
455	A_37_P149072	2.70E-006	PODNL1
456	A_37_P202311	2.71E-006	C11orf84
457	A_37_P425833	2.72E-006	C11orf2
458	A_37_P153315	2.74E-006	U2AF1L4
459	A_37_P202782	2.74E-006	CCDC88B
460	A_37_P156224	2.74E-006	TMUB1
461	A_37_P375850	2.74E-006	SULT2B1
462	A_37_P129194	2.74E-006	TMEM183B
463	A_37_P102327	2.74E-006	PPP4R1
464	A_37_P209921	2.75E-006	RAB1B
465	A_37_P309628	2.76E-006	NKIRAS2
466	A_37_P395238	2.76E-006	SCARA3
467	A_37_P055927	2.78E-006	TTL
468	A_37_P334333	2.78E-006	B4GALT2
469	A_37_P008830	2.78E-006	TSPAN17
470	A_37_P434380	2.78E-006	SDHD
471	A_37_P103275	2.78E-006	TTC7B
472	A_33_P3215178	2.78E-006	PIGQ
473	A_37_P117776	2.79E-006	DEDD
474	A_37_P163923	2.79E-006	SUMF2
475	A_37_P133243	2.79E-006	LAMA4
476	A_37_P001185	2.80E-006	RNF44
477	A_37_P038843	2.80E-006	KARS
478	A_37_P033682	2.81E-006	USP31
479	A_37_P116433	2.81E-006	GNPAT
480	A_37_P151116	2.81E-006	RPL28
481	A_37_P122921	2.84E-006	ICMT
482	A_37_P162633	2.85E-006	RHBDD2
483	A_37_P211957	2.85E-006	TMEM9B
484	A_37_P054598	2.87E-006	SPEG
485	A_37_P124505	2.87E-006	PLEKHM2
486	A_37_P047331	2.87E-006	AUP1
487	A_24_P280029	2.88E-006	PDXP

3817	A_37_P145525	0.00026884	FCHO1
3818	A_37_P032974	0.00026903	LCAT
3819	A_24_P16036	0.00026903	C2orf79
3820	A_37_P119536	0.00026945	UBXN11
3821	A_37_P179851	0.00026975	SH3GL3
3822	A_37_P019092	0.00026985	RARB
3823	A_37_P012037	0.00027016	COL6A6
3824	A_37_P026752	0.00027039	C4orf52
3825	A_37_P234289	0.00027109	ATP2C1
3826	A_37_P183537	0.0002711	ZMAT5
3827	A_37_P202490	0.00027116	ADAMTS8
3828	A_37_P190312	0.00027342	RBPJL
3829	A_37_P069446	0.00027367	ARR3
3830	A_37_P058188	0.00027448	BTBD11
3831	A_37_P137106	0.00027453	TFEB
3832	A_37_P002970	0.00027515	GPR98
3833	A_37_P110558	0.0002753	RNF19B
3834	A_37_P129678	0.00027532	KIAA0562
3835	A_37_P234190	0.00027545	ACAD11
3836	A_37_P214538	0.00027589	APP
3837	A_37_P094663	0.00027598	BNC2
3838	A_33_P3214310	0.00027604	FOXP1
3839	A_37_P102379	0.00027739	WDR7
3840	A_37_P147473	0.00027771	BCAT2
3841	A_37_P158353	0.00027783	GTF2IRD1
3842	A_37_P181728	0.000278	TTLL1
3843	A_37_P287595	0.00027844	LRP1
3844	A_24_P168574	0.00027844	ENST00000481768
3845	A_33_P3345186	0.00027908	CHST13
3846	A_37_P225894	0.00027921	PROP1
3847	A_37_P309394	0.00027953	NAGLU
3848	A_37_P425693	0.00027954	BBS1
3849	A_37_P137774	0.00027977	LST1
3850	A_37_P078244	0.00028071	ACCN1
3851	A_37_P379677	0.0002812	CCDC129
3852	A_37_P004774	0.00028124	C5orf56
3853	A_37_P051027	0.00028131	ZNF513
3854	A_37_P077602	0.00028158	ADORA2B
3855	A_37_P356924	0.00028172	NOTCH4
3856	A_37_P066473	0.00028215	CACNA2D4
3857	A_33_P3401422	0.00028233	LIPM
3858	A_33_P3377194	0.00028235	ADRA1A
3859	A_37_P373511	0.00028264	PALM
3860	A_37_P041461	0.00028285	CLK1
3861	A_37_P077411	0.00028308	ACE
3862	A_37_P075913	0.00028345	SRPX2
3863	A_37_P391379	0.00028358	GOT1L1
3864	A_37_P362858	0.00028362	PPP2R5D
3865	A_37_P129175	0.00028372	CD1B
3866	A_37_P057521	0.00028372	PRDM4
3867	A_37_P050725	0.00028407	NEU4
3868	A_37_P000867	0.00028423	TRIM7
3869	A_37_P247777	0.00028517	CYP2U1
3870	A_37_P243958	0.00028538	CBLB
3871	A_37_P182908	0.00028543	CYB5R3
3872	A_37_P116061	0.00028566	GPR157
3873	A_37_P008522	0.00028649	FBN2
3874	A_37_P338744	0.0002868	EPHB2
3875	A_37_P057139	0.00028686	PRB3
3876	A_37_P326338	0.00028712	ADCK1
3877	A_37_P226207	0.00028715	FAM151B

488	A_37_P028660	2.88E-006	QDPR
489	A_37_P147243	2.88E-006	LIN7B
490	A_23_P10156	2.90E-006	CHMP6
491	A_37_P023832	2.90E-006	COQ2
492	A_37_P160645	2.93E-006	VOPP1
493	A_37_P066257	2.93E-006	RFX4
494	A_37_P214675	2.94E-006	IL10RB
495	A_37_P179343	2.96E-006	RCN2
496	A_37_P023997	2.96E-006	MFSD10
497	A_37_P093753	2.97E-006	IL11RA
498	A_37_P419955	2.97E-006	ZDHHC6
499	A_37_P067985	2.98E-006	TMEM120B
500	A_37_P207808	2.99E-006	MAPK8IP1
501	A_37_P317546	2.99E-006	DENND1A
502	A_37_P033629	2.99E-006	LITAF
503	A_37_P367617	2.99E-006	TIMM13
504	A_37_P430258	2.99E-006	DKK3
505	A_37_P391847	3.00E-006	GPAA1
506	A_37_P357970	3.00E-006	DUSP22
507	A_37_P009985	3.01E-006	CLCN2
508	A_37_P184806	3.01E-006	RPS19BP1
509	A_37_P200688	3.01E-006	ZDHHC16
510	A_37_P097375	3.03E-006	RXRA
511	A_37_P095733	3.04E-006	NOXA1
512	A_37_P053694	3.05E-006	FAM119A
513	A_37_P435416	3.05E-006	PDE2A
514	A_37_P238985	3.05E-006	KLHDC8B
515	A_37_P155358	3.06E-006	FTSJ2
516	A_37_P440818	3.06E-006	CRYL1
517	A_37_P155119	3.07E-006	ARMC10
518	A_23_P12329	3.07E-006	APH1A
519	A_23_P40880	3.07E-006	CMTM8
520	A_37_P151502	3.07E-006	SLC35E1
521	A_37_P080168	3.08E-006	GIT1
522	A_37_P087881	3.09E-006	FAM18B2
523	A_37_P388729	3.11E-006	YKT6
524	A_37_P269362	3.14E-006	TEX261
525	A_37_P186719	3.14E-006	C20orf108
526	A_37_P141114	3.15E-006	CCND3
527	A_37_P037800	3.16E-006	PRR14
528	A_37_P200401	3.16E-006	UBTD1
529	A_37_P286854	3.16E-006	HEBP1
530	A_37_P053859	3.16E-006	FAM136A
531	A_24_P322229	3.19E-006	RASL10B
532	A_37_P375601	3.20E-006	KANK3
533	A_37_P069750	3.21E-006	WDR45
534	A_33_P3258747	3.22E-006	C21orf63
535	A_37_P082093	3.23E-006	PCYT2
536	A_37_P089138	3.25E-006	TRAF4
537	A_37_P056003	3.26E-006	UBE2F
538	A_37_P431344	3.27E-006	LRFN4
539	A_37_P211097	3.27E-006	SLC39A13
540	A_24_P160001	3.27E-006	FKBP1A
541	A_24_P19175	3.27E-006	ZNF358
542	A_37_P056766	3.27E-006	ZFAND2B
543	A_37_P272058	3.28E-006	RABL2A
544	A_37_P211530	3.28E-006	STX3
545	A_37_P139501	3.28E-006	C6orf27
546	A_37_P378256	3.28E-006	RASA4
547	A_37_P078441	3.28E-006	WIPI1
548	A_37_P140798	3.30E-006	STK19

3878	A_37_P062056	0.00028721	ATP5G2
3879	A_24_P339514	0.00028741	CYP2B6
3880	A_37_P155087	0.00028743	KLRG2
3881	A_37_P135044	0.00028752	SLC22A2
3882	A_37_P129736	0.00028885	TSPAN1
3883	A_37_P038453	0.00028901	SETD6
3884	A_37_P339378	0.00028927	FGGY
3885	A_24_P313993	0.00028942	CAPS
3886	A_37_P209725	0.00028945	PRDM11
3887	A_37_P116876	0.00028945	HCRTR1
3888	A_37_P015072	0.00028945	MRPL3
3889	A_37_P034666	0.00028966	IL4R
3890	A_37_P130398	0.0002898	VWA5B1
3891	A_23_P11729	0.00028999	ZBTB7B
3892	A_37_P059687	0.00029033	SLC6A13
3893	A_37_P165801	0.00029042	SLC45A4
3894	A_37_P399220	0.00029058	KIAA0101
3895	A_37_P146982	0.00029087	KIRREL2
3896	A_37_P143114	0.00029134	LPPR3
3897	A_37_P312745	0.00029223	TNFSF12
3898	A_37_P180491	0.00029277	THSD4
3899	A_37_P287783	0.00029307	LYRM5
3900	A_37_P188623	0.00029308	LIME1
3901	A_37_P148177	0.0002931	PTGER1
3902	A_37_P187477	0.00029313	ZNF335
3903	A_37_P028666	0.00029326	QRFPR
3904	A_33_P3326235	0.0002933	HBM
3905	A_37_P057932	0.00029469	CLLU1OS
3906	A_24_P315056	0.0002949	C10orf122
3907	A_37_P185551	0.00029515	SMTN
3908	A_37_P335420	0.00029517	CA6
3909	A_37_P207339	0.00029595	MYBPC3
3910	A_37_P179119	0.00029605	STOML1
3911	A_37_P306645	0.00029642	AZI1
3912	A_37_P346967	0.00029672	SLC2A1
3913	A_37_P081447	0.00029696	FN3K
3914	A_37_P153819	0.00029703	ZNF559
3915	A_32_P133840	0.00029704	TMCC2
3916	A_37_P097606	0.00029779	SLC31A2
3917	A_37_P354865	0.00029862	KIF21B
3918	A_37_P411985	0.00029897	SLC23A2
3919	A_37_P027285	0.00029897	HPSE
3920	A_37_P051210	0.00029996	POLR1A
3921	A_37_P143789	0.00030008	NOTCH3
3922	A_37_P047187	0.00030015	HAAO
3923	A_37_P200799	0.00030042	ZNF365
3924	A_37_P065292	0.00030047	SLC2A14
3925	A_33_P3244283	0.00030085	CAMK2N2
3926	A_37_P162394	0.00030085	PVRIG
3927	A_37_P198067	0.00030108	NET1
3928	A_37_P194643	0.00030122	CUBN
3929	A_33_P3217517	0.00030125	C5orf55
3930	A_37_P414950	0.00030149	UBE2C
3931	A_37_P403285	0.00030212	RYR3
3932	A_37_P241013	0.0003022	TM4SF19
3933	A_23_P255328	0.00030236	PRDM8
3934	A_37_P321348	0.00030263	CBWD1
3935	A_37_P108537	0.00030284	CDCA4
3936	A_37_P126105	0.00030365	IQGAP3
3937	A_37_P260837	0.00030438	NOD2
3938	A_37_P192259	0.00030489	FRMPD2

549	A_37_P086824	3.30E-006	RAC3
550	A_37_P025106	3.30E-006	UBE2D3
551	A_37_P047124	3.33E-006	DTYMK
552	A_32_P827528	3.33E-006	S1PR2
553	A_37_P147415	3.34E-006	YIPF2
554	A_23_P50389	3.34E-006	NAT14
555	A_37_P035999	3.34E-006	SLC6A8
556	A_37_P366619	3.34E-006	ARRDC2
557	A_37_P434846	3.35E-006	SIGIRR
558	A_37_P310848	3.35E-006	RNF167
559	A_37_P034918	3.36E-006	KATNB1
560	A_37_P049397	3.36E-006	TSSC1
561	A_37_P098238	3.37E-006	TMEM141
562	A_37_P351005	3.37E-006	SERINC2
563	A_37_P127752	3.38E-006	SLC25A33
564	A_37_P122940	3.38E-006	C1orf43
565	A_37_P084768	3.38E-006	DUSP3
566	A_37_P109471	3.38E-006	AGTRAP
567	A_23_P98844	3.39E-006	ARHGEF25
568	A_37_P122084	3.39E-006	MSTO1
569	A_33_P3275350	3.40E-006	NCS1
570	A_33_P3266828	3.41E-006	TMEM8B
571	A_37_P342308	3.41E-006	ZNF496
572	A_37_P205764	3.41E-006	MRPL11
573	A_37_P373014	3.41E-006	NCLN
574	A_37_P143435	3.42E-006	TMEM160
575	A_37_P109923	3.43E-006	APOA1BP
576	A_37_P044999	3.43E-006	FAM134A
577	A_37_P128386	3.43E-006	RHOC
578	A_24_P627306	3.44E-006	EDEM2
579	A_37_P322389	3.44E-006	URM1
580	A_37_P330338	3.44E-006	PACS2
581	A_37_P104850	3.45E-006	JDP2
582	A_37_P019765	3.45E-006	SEC22C
583	A_24_P349466	3.45E-006	PRR13
584	A_24_P205213	3.45E-006	ARSB
585	A_37_P129833	3.45E-006	SLC39A1
586	A_37_P373813	3.45E-006	PIK3R2
587	A_37_P178183	3.47E-006	C15orf17
588	A_37_P245302	3.48E-006	UBE2E2
589	A_37_P150499	3.48E-006	ERF
590	A_37_P399209	3.48E-006	FURIN
591	A_37_P117051	3.48E-006	SCMH1
592	A_37_P098879	3.49E-006	RAPGEF1
593	A_37_P419025	3.51E-006	ASB13
594	A_37_P372597	3.51E-006	MRPL4
595	A_37_P049465	3.53E-006	THAP4
596	A_37_P056857	3.53E-006	SIX2
597	A_33_P3381338	3.53E-006	TNXB
598	A_37_P298187	3.53E-006	NXT2
599	A_33_P3250671	3.53E-006	TCF7
600	A_37_P030828	3.54E-006	CHIC2
601	A_37_P184457	3.54E-006	NCAPH2
602	A_37_P081474	3.55E-006	FOXK2
603	A_37_P265293	3.56E-006	ARPC2
604	A_37_P332572	3.57E-006	ZFYVE21
605	A_37_P111149	3.57E-006	C1orf144
606	A_37_P266735	3.59E-006	MEMO1
607	A_37_P161296	3.60E-006	POLD2
608	A_37_P189061	3.60E-006	SLMO2
609	A_24_P104119	3.60E-006	RHOF

3939	A_37_P043035	0.00030505	CEP68
3940	A_37_P270279	0.00030516	IGFBP2
3941	A_37_P189594	0.00030516	C20orf194
3942	A_37_P037570	0.00030585	SHCBP1
3943	A_37_P069593	0.00030609	AVPR2
3944	A_37_P008579	0.00030618	CD74
3945	A_37_P145955	0.00030695	SLC8A2
3946	A_37_P249231	0.0003078	HGFAC
3947	A_37_P138055	0.00030783	MTHFD1L
3948	A_37_P204994	0.00030785	SF1
3949	A_37_P055465	0.0003083	CD207
3950	A_37_P173154	0.00030835	ACAN
3951	A_37_P020342	0.00030857	NCKIPSD
3952	A_37_P132619	0.00030912	SERAC1
3953	A_37_P231512	0.00030964	SLC36A1
3954	A_37_P104960	0.00030974	PSMB5
3955	A_37_P152594	0.0003098	SIGLEC8
3956	A_37_P007826	0.00030999	SLC4A9
3957	A_37_P302716	0.00031039	NOS2
3958	A_37_P105927	0.00031045	VIPAR
3959	A_37_P411182	0.0003109	GHRH
3960	A_23_P116587	0.00031124	OMP
3961	A_37_P118005	0.00031154	KCNK2
3962	A_23_P166	0.00031174	MOBKL2C
3963	A_37_P367004	0.00031193	CATSPERG
3964	A_37_P031273	0.00031197	NLRC3
3965	A_37_P301612	0.000313	CLEC10A
3966	A_37_P185424	0.000313	SHANK3
3967	A_23_P104617	0.00031313	GYLTL1B
3968	A_37_P202865	0.00031325	CD6
3969	A_23_P67355	0.00031334	PRRG2
3970	A_37_P019419	0.00031393	RNF7
3971	A_23_P350574	0.00031431	FCRLB
3972	A_23_P379034	0.00031431	BAIAP2L2
3973	A_37_P409745	0.00031449	RANGAP1
3974	A_37_P185347	0.00031449	SFI1
3975	A_37_P209979	0.00031539	XRRA1
3976	A_37_P088680	0.00031615	TRPV3
3977	A_37_P190995	0.00031698	KCNQ2
3978	A_37_P340351	0.0003174	ASAP3
3979	A_37_P071005	0.00031785	DRP2
3980	A_37_P131038	0.0003184	MATN1
3981	A_37_P202520	0.00031865	CABP4
3982	A_37_P035127	0.00031915	LAT
3983	A_37_P417704	0.00031959	DMBT1
3984	A_37_P083034	0.00031984	RBFOX3
3985	A_23_P257649	0.00032022	RBP1
3986	A_37_P181305	0.00032027	ZWILCH
3987	A_23_P113656	0.00032052	TUBB4
3988	A_37_P122245	0.00032087	RFWD2
3989	A_33_P3417626	0.00032106	ENHO
3990	A_37_P026311	0.00032153	LIAS
3991	A_37_P380070	0.0003219	CLEC2L
3992	A_37_P135050	0.00032196	SLC22A23
3993	A_37_P132994	0.0003223	C6orf48
3994	A_37_P294879	0.00032238	FAAH2
3995	A_37_P106293	0.00032248	MRPL52
3996	A_37_P428464	0.00032283	MMP10
3997	A_37_P166861	0.00032295	CTHRC1
3998	A_37_P426239	0.00032306	GALNTL4
3999	A_37_P054988	0.00032322	SULT1C2

610	A_37_P169519	3.60E-006	SLC25A32
611	A_37_P311265	3.61E-006	CANT1
612	A_37_P340355	3.62E-006	GSTM1
613	A_37_P175242	3.63E-006	FAM108C1
614	A_37_P166892	3.63E-006	TOP1MT
615	A_37_P204840	3.64E-006	CYBASC3
616	A_37_P380487	3.65E-006	TMEM184A
617	A_24_P130962	3.65E-006	TOR3A
618	A_33_P3422133	3.66E-006	ADAP1
619	A_37_P399912	3.66E-006	ISG20
620	A_37_P124489	3.67E-006	SLC30A2
621	A_37_P149888	3.67E-006	PDE4A
622	A_37_P134050	3.68E-006	GMDS
623	A_37_P171020	3.69E-006	PTDSS1
624	A_37_P208330	3.70E-006	MUS81
625	A_37_P007239	3.70E-006	RMND5B
626	A_24_P397515	3.72E-006	HMGN4
627	A_37_P151498	3.73E-006	CLASRP
628	A_37_P208878	3.73E-006	OAF
629	A_37_P367120	3.75E-006	C19orf63
630	A_37_P303749	3.75E-006	PIGS
631	A_37_P029536	3.75E-006	STK32B
632	A_37_P146408	3.75E-006	HSD11B1L
633	A_37_P058919	3.76E-006	CACNB3
634	A_37_P241228	3.76E-006	HYAL2
635	A_37_P263757	3.76E-006	VPS4A
636	A_37_P349049	3.76E-006	ERI3
637	A_37_P271924	3.77E-006	BIN1
638	A_37_P053124	3.78E-006	RPIA
639	A_37_P128030	3.78E-006	JMJD4
640	A_37_P064122	3.82E-006	Mar-09
641	A_37_P245965	3.82E-006	AADAT
642	A_37_P127484	3.82E-006	SH3BGRL3
643	A_23_P151820	3.82E-006	RIN3
644	A_37_P181672	3.82E-006	PHF5A
645	A_37_P129631	3.83E-006	TRNAU1AP
646	A_37_P329281	3.85E-006	ZNF219
647	A_37_P071209	3.85E-006	TMEM185A
648	A_37_P054877	3.85E-006	STK16
649	A_37_P407855	3.85E-006	PMM1
650	A_23_P368896	3.86E-006	SNX12
651	A_37_P439033	3.86E-006	C21orf56
652	A_37_P011779	3.89E-006	CHRD
653	A_37_P207908	3.90E-006	HRAS
654	A_37_P374136	3.90E-006	ERCC1
655	A_37_P425010	3.90E-006	KLHL35
656	A_23_P150249	3.91E-006	CCDC85B
657	A_24_P259276	3.92E-006	ZDHHC24
658	A_37_P260201	3.92E-006	C16orf42
659	A_23_P55926	3.93E-006	NR1H2
660	A_37_P242303	3.93E-006	SHISA5
661	A_37_P213984	3.94E-006	BACE2
662	A_37_P186723	3.95E-006	C20orf11
663	A_37_P186556	3.97E-006	ACOT8
664	A_23_P62371	3.98E-006	TAZ
665	A_37_P305440	4.00E-006	NT5C3L
666	A_37_P130943	4.00E-006	RUNX3
667	A_37_P155737	4.00E-006	C7orf59
668	A_37_P172363	4.01E-006	FBXL6
669	A_23_P64499	4.03E-006	MOB2
670	A_37_P124538	4.04E-006	PLEKHO1

4000	A_37_P073511	0.00032327	OPHN1
4001	A_23_P94840	0.00032332	DYNLRB2
4002	Rat-GAPDH_5	0.00032425	Rat-GAPDH_5
4003	A_37_P010345	0.00032615	ARPC4
4004	A_37_P020136	0.00032634	SLC22A14
4005	A_37_P037714	0.00032727	C16orf93
4006	A_33_P3416588	0.00032745	RIT2
4007	A_37_P144896	0.00032748	ANKRD27
4008	A_37_P040198	0.00032807	WWP2
4009	A_37_P094824	0.00032844	TLN1
4010	A_33_P3246829	0.00032888	IL1RN
4011	A_37_P143170	0.00032906	XAB2
4012	A_37_P414813	0.00032914	TMC2
4013	A_37_P183996	0.00032969	PLXNB2
4014	A_37_P376620	0.00033086	TMIGD2
4015	A_37_P144815	0.00033101	LHB
4016	A_33_P3259022	0.00033127	SLC6A2
4017	A_37_P125058	0.0003316	PRAMEF6
4018	A_37_P291592	0.00033172	RIMBP2
4019	A_37_P096547	0.00033181	PRSS3
4020	A_37_P237484	0.00033276	FXR1
4021	A_37_P042253	0.00033318	UNC80
4022	A_37_P332712	0.00033319	PGBD5
4023	A_37_P072711	0.00033423	ZMYM3
4024	A_37_P160162	0.00033478	MGAM
4025	A_37_P075290	0.00033492	RIBC1
4026	A_23_P118203	0.00033493	ZG16B
4027	A_37_P124323	0.00033498	IL22RA1
4028	A_37_P295765	0.00033505	HDAC6
4029	A_37_P152595	0.00033544	TMEM190
4030	A_37_P201646	0.00033588	ARHGEF17
4031	A_37_P280627	0.00033601	NGEF
4032	A_37_P353806	0.00033611	UFC1
4033	A_37_P003867	0.00033652	SPARC
4034	A_37_P016465	0.00033681	PLSCR2
4035	A_37_P167754	0.00033735	FAM92A1
4036	A_37_P038146	0.00033782	RLTPR
4037	A_33_P3360972	0.00033807	C14orf73
4038	A_37_P175166	0.00033853	A_37_P175166
4039	A_37_P142000	0.00033894	NFKBIE
4040	A_37_P135225	0.00033946	DAXX
4041	A_37_P118116	0.00033964	KIAA0467
4042	A_37_P045615	0.00034006	TMEM194B
4043	A_37_P398159	0.00034048	MFGE8
4044	A_37_P030372	0.00034083	FGG
4045	A_37_P142481	0.00034144	CKM
4046	A_37_P031632	0.00034171	ATP2C2
4047	A_37_P035345	0.00034205	BCAR1
4048	A_37_P077161	0.00034225	ZNF81
4049	A_37_P407999	0.00034291	PNPLA5
4050	A_37_P187300	0.00034343	CHGB
4051	A_37_P067441	0.00034348	STAB2
4052	A_37_P379720	0.00034381	CCDC136
4053	A_37_P054554	0.00034427	SPATA3
4054	A_37_P192842	0.0003447	C10orf78
4055	A_37_P096724	0.00034537	SCAI
4056	A_37_P421540	0.00034549	MRC1L1
4057	A_37_P300171	0.00034561	CD99L2
4058	A_37_P186235	0.00034645	MB
4059	A_37_P089428	0.00034647	UNK
4060	A_37_P210901	0.00034657	SLC22A18

671	A_37_P402973	4.04E-006	LPCAT4
672	A_37_P398176	4.05E-006	COMMD4
673	A_37_P422229	4.05E-006	PDSS1
674	A_33_P3423365	4.05E-006	GSN
675	A_37_P038424	4.05E-006	TMEM8A
676	A_37_P308379	4.07E-006	DUS1L
677	A_37_P148358	4.08E-006	LRP3
678	A_37_P135290	4.08E-006	GTF2H4
679	A_37_P367025	4.09E-006	C19orf24
680	A_37_P104830	4.10E-006	ISCA2
681	A_37_P029659	4.13E-006	TBC1D14
682	A_33_P3266410	4.13E-006	MAZ
683	A_37_P210764	4.14E-006	SIDT2
684	A_37_P131474	4.18E-006	RARS2
685	A_37_P022028	4.19E-006	SLC41A3
686	A_23_P396062	4.22E-006	RAB40C
687	A_37_P088765	4.22E-006	TIMM22
688	A_37_P053541	4.23E-006	SCLY
689	A_33_P3408320	4.23E-006	LASS1
690	A_37_P315311	4.24E-006	SURF1
691	A_37_P102924	4.24E-006	CNIH
692	A_37_P113671	4.26E-006	CREG1
693	A_37_P131550	4.26E-006	FLOT1
694	A_23_P398372	4.27E-006	C9orf69
695	A_23_P338325	4.28E-006	ELK3
696	A_37_P353653	4.28E-006	UAP1
697	A_37_P206694	4.29E-006	BET1L
698	A_37_P137942	4.29E-006	MICA
699	A_37_P087090	4.30E-006	RNASEK
700	A_33_P3258946	4.30E-006	SIRT2
701	A_37_P236605	4.30E-006	DVL3
702	A_24_P92367	4.31E-006	YDJC
703	A_37_P079528	4.31E-006	CDR2L
704	A_37_P171449	4.31E-006	RNF139
705	A_37_P015591	4.32E-006	ZDHHC3
706	A_37_P251905	4.32E-006	PAQR3
707	A_37_P183585	4.33E-006	YPEL1
708	A_37_P311241	4.33E-006	CAMTA2
709	A_24_P386771	4.34E-006	PPP1CC
710	A_37_P226840	4.37E-006	GRK6
711	A_37_P069347	4.37E-006	APEX2
712	A_37_P132850	4.37E-006	RRP36
713	A_37_P396639	4.37E-006	ZDHHC2
714	A_37_P087485	4.38E-006	PCGF2
715	A_37_P411481	4.38E-006	TBC1D20
716	A_37_P020849	4.39E-006	POC1A
717	A_37_P350015	4.40E-006	RNF11
718	A_37_P164781	4.40E-006	UPP1
719	A_33_P3292794	4.41E-006	SMUG1
720	A_37_P191366	4.43E-006	SRMS
721	A_37_P152064	4.44E-006	FAM71E1
722	A_37_P167362	4.46E-006	E2F5
723	A_37_P350246	4.46E-006	MIIP
724	A_37_P117044	4.47E-006	HIAT1
725	A_37_P233603	4.47E-006	SSR3
726	A_37_P384834	4.47E-006	MOSPD3
727	A_37_P201927	4.48E-006	B4GALNT4
728	A_37_P193790	4.49E-006	NPM3
729	A_37_P034123	4.49E-006	MLST8
730	A_37_P193181	4.49E-006	PHYH
731	A_37_P300510	4.49E-006	VAMP7

4061	A_37_P131410	0.00034688	AARS2
4062	A_37_P068451	0.00034692	ULK1
4063	A_37_P145667	0.00034736	FTL
4064	A_37_P153345	0.0003474	ZBTB32
4065	A_37_P375760	0.00034775	LYL1
4066	A_37_P106426	0.00034798	MYH7
4067	A_37_P107431	0.00034806	RNF31
4068	A_33_P3289296	0.00034827	TMEM37
4069	A_23_P321703	0.0003486	BCL2A1
4070	A_37_P022198	0.00034868	NICN1
4071	A_37_P336492	0.00034906	CGN
4072	A_37_P090393	0.00034947	FNBP1
4073	A_37_P262682	0.00034953	CCDC154
4074	A_23_P82108	0.00034987	ZBTB2
4075	A_37_P399941	0.00035049	IVD
4076	A_37_P184471	0.00035089	NCF4
4077	A_37_P162040	0.00035216	POU6F2
4078	A_37_P309694	0.00035239	MYO18A
4079	A_37_P098719	0.00035246	CDKN2B
4080	A_37_P212039	0.00035261	TMEM45B
4081	A_33_P3835524	0.00035269	POU2F2
4082	A_37_P077235	0.00035279	AARSD1
4083	A_37_P085051	0.00035288	C17orf103
4084	A_37_P171733	0.00035295	SLC7A2
4085	A_37_P349737	0.00035425	RCOR3
4086	A_37_P149348	0.00035465	NDUFA13
4087	A_37_P014452	0.00035536	HTR3E
4088	A_37_P033053	0.00035632	CRAMP1L
4089	A_37_P118214	0.00035651	KIAA1324
4090	A_37_P292003	0.00035712	MAP3K12
4091	A_37_P190611	0.0003574	JAG1
4092	A_37_P132857	0.0003586	C6orf162
4093	A_37_P111796	0.00035898	CACNA1E
4094	A_37_P091217	0.00035937	CACNA1B
4095	A_37_P340470	0.0003595	MORN1
4096	A_37_P301968	0.00035953	B4GALNT2
4097	A_37_P190830	0.00035979	SPO11
4098	A_37_P041195	0.00036091	FN1
4099	A_37_P148745	0.00036104	HSD17B14
4100	A_37_P043439	0.00036127	COPS8
4101	A_37_P191355	0.00036129	SIRPD
4102	A_33_P3304691	0.00036227	KRTAP5-1
4103	A_37_P153870	0.00036275	ZNF584
4104	A_37_P067024	0.00036289	SLC39A5
4105	A_37_P106840	0.00036293	ENTPD5
4106	A_37_P253374	0.00036304	TBC1D19
4107	A_23_P349406	0.00036335	RIMKLA
4108	A_37_P277609	0.00036423	SLC11A1
4109	A_23_P8083	0.00036506	LY6G6C
4110	A_37_P166967	0.00036506	ANK1
4111	A_37_P000831	0.00036528	ADAM19
4112	A_37_P352970	0.00036549	TMEM39B
4113	A_23_P16063	0.00036585	GRIK5
4114	A_37_P209631	0.00036614	POU2F3
4115	A_33_P3368358	0.00036631	NEDD9
4116	A_37_P140416	0.00036638	SLC22A1
4117	A_37_P185055	0.00036673	SEC14L4
4118	A_37_P060657	0.00036777	ESPL1
4119	A_37_P178670	0.00036837	ELL3
4120	A_37_P144530	0.00036884	NPHS1
4121	A_37_P127949	0.00037079	SLC44A3

732	A_37_P333093	4.50E-006	S100A2
733	A_37_P210444	4.50E-006	RPS6KB2
734	A_37_P373823	4.50E-006	SHC2
735	A_37_P082316	4.50E-006	SPAG7
736	A_37_P189807	4.53E-006	PANK2
737	A_37_P251689	4.53E-006	PCGF3
738	A_23_P41204	4.53E-006	FAM131A
739	A_37_P130578	4.54E-006	WDTC1
740	A_37_P067799	4.54E-006	TEAD4
741	A_24_P126471	4.56E-006	SLC12A9
742	A_37_P207500	4.56E-006	C11orf59
743	A_37_P425128	4.56E-006	SAAL1
744	A_32_P99432	4.56E-006	TRAPPC5
745	A_37_P040652	4.57E-006	CHPF
746	A_37_P150919	4.57E-006	RELB
747	A_37_P019933	4.58E-006	ITIH4
748	A_37_P247166	4.58E-006	C4orf48
749	A_37_P020031	4.58E-006	SIAH2
750	A_23_P154256	4.58E-006	ELMOD3
751	A_37_P205605	4.59E-006	MPPED2
752	A_37_P202306	4.60E-006	C11orf83
753	A_37_P443401	4.65E-006	SLC25A15
754	A_37_P139169	4.66E-006	PPT2
755	A_37_P211334	4.66E-006	SLC37A4
756	A_37_P142949	4.68E-006	TMEM149
757	A_37_P326788	4.68E-006	C14orf153
758	A_37_P169817	4.68E-006	LY6E
759	A_37_P354204	4.69E-006	KIAA2013
760	A_37_P178527	4.69E-006	NGRN
761	A_37_P402079	4.69E-006	PTPN9
762	A_37_P425382	4.70E-006	ARNTL
763	A_37_P158155	4.70E-006	GNB2
764	A_37_P367959	4.72E-006	CLPTM1
765	A_37_P048811	4.74E-006	OLA1
766	A_37_P154247	4.74E-006	ZNF823
767	A_37_P436110	4.77E-006	CDCA5
768	A_37_P149996	4.77E-006	PGLS
769	A_37_P424165	4.77E-006	PPP3CB
770	A_37_P025431	4.78E-006	DCTD
771	A_37_P152072	4.78E-006	STRN4
772	A_32_P505133	4.78E-006	C19orf47
773	A_37_P028814	4.78E-006	RAB28
774	A_37_P133848	4.78E-006	SLC17A5
775	A_37_P198087	4.80E-006	NEURL
776	A_37_P289896	4.80E-006	RASSF3
777	A_24_P27977	4.81E-006	TRPM2
778	A_37_P186848	4.82E-006	C20orf20
779	A_33_P3335596	4.82E-006	C17orf62
780	A_37_P017637	4.82E-006	NDUFB5
781	A_37_P209372	4.83E-006	PELI3
782	A_37_P309416	4.86E-006	NARF
783	A_37_P059876	4.87E-006	CS
784	A_37_P316937	4.87E-006	GNG10
785	A_37_P071332	4.90E-006	FHL1
786	A_37_P306407	4.90E-006	PLCD3
787	A_37_P080224	4.90E-006	DBF4B
788	A_37_P186067	4.94E-006	PPPDE2
789	A_37_P194803	4.96E-006	FBXO18
790	A_37_P086122	4.97E-006	MYO19
791	A_37_P067875	4.99E-006	RILPL2
792	A_37_P351855	4.99E-006	SNAPIN

4122	A_37_P165068	0.0003708	ZAN
4123	A_37_P200278	0.00037085	TMEM72
4124	A_37_P109919	0.00037098	APITD1
4125	A_33_P3360271	0.00037243	EVX2
4126	A_37_P304783	0.00037355	PITPNM3
4127	A_37_P382793	0.0003736	ISPD
4128	A_33_P3351092	0.00037365	KRTAP20-2
4129	A_37_P266475	0.00037407	COL6A3
4130	A_37_P175126	0.00037414	GOLGA8B
4131	A_23_P62709	0.00037436	SPRR3
4132	A_23_P341325	0.00037518	RPL10L
4133	A_37_P081643	0.00037522	RAP1GAP2
4134	A_37_P108232	0.00037539	CCNB1IP1
4135	A_33_P3267230	0.00037578	TBC1D3G
4136	A_37_P303653	0.00037587	ALOXE3
4137	A_37_P042010	0.00037671	BRE
4138	A_37_P033195	0.00037693	TAT
4139	A_37_P338323	0.00037755	CSMD2
4140	A_37_P180185	0.00037766	SPATA8
4141	A_37_P129623	0.00037788	RIMS3
4142	A_37_P157347	0.00037791	AOAH
4143	A_37_P061883	0.00037889	IKZF4
4144	A_37_P130891	0.00037894	ZMYM4
4145	A_33_P3263664	0.000379	KRTAP6-2
4146	A_37_P263504	0.00037905	KIAA0895L
4147	A_37_P217650	0.00037926	GTF3A
4148	A_37_P068746	0.00037954	TUBA1A
4149	A_37_P184042	0.00038033	MAFF
4150	A_37_P099711	0.00038033	CNDP2
4151	A_37_P135321	0.00038156	MOXD1
4152	A_37_P401930	0.00038195	C15orf40
4153	A_37_P294869	0.00038305	F9
4154	A_37_P170733	0.00038337	PKIA
4155	A_37_P235567	0.00038338	CMTM7
4156	A_37_P142887	0.00038414	ARID3A
4157	A_37_P202229	0.00038475	C11orf63
4158	A_37_P134023	0.0003852	KHDC1L
4159	A_37_P182492	0.00038525	MICAL3
4160	A_33_P3405995	0.00038591	YBEY
4161	A_37_P397223	0.00038593	ARNT2
4162	A_37_P013272	0.00038655	EPHB1
4163	A_37_P397251	0.00038664	FRMD5
4164	A_37_P018878	0.00038696	PTH1R
4165	A_37_P289102	0.0003873	PFDN5
4166	A_37_P016644	0.0003873	PLXNB1
4167	A_37_P096142	0.00038734	PCSK5
4168	A_37_P185316	0.00038767	SEZ6L
4169	A_37_P202067	0.00038795	WT1
4170	A_37_P341954	0.00038856	TNFRSF25
4171	A_37_P043466	0.0003892	ALK
4172	A_37_P129369	0.00039105	TNFRSF8
4173	A_37_P061581	0.00039162	PLA2G1B
4174	A_37_P207404	0.00039224	C11orf45
4175	A_37_P158084	0.00039397	GHRHR
4176	A_37_P204074	0.00039435	EFCAB4A
4177	A_37_P280897	0.00039475	ACACB
4178	A_33_P3325404	0.0003951	CRIP3
4179	A_37_P088346	0.00039529	FAM83G
4180	A_23_P355447	0.00039592	ZDHHC22
4181	A_37_P364918	0.00039615	KHDC1
4182	A_37_P183523	0.00039745	L3MBTL2

793	A_23_P144877	4.99E-006	ATOX1
794	A_37_P048899	4.99E-006	HES6
795	A_37_P004046	4.99E-006	MRPS27
796	A_33_P3342917	4.99E-006	SYNGR2
797	A_37_P334796	5.00E-006	C1orf122
798	A_37_P046848	5.01E-006	GTF3C2
799	A_37_P027900	5.05E-006	PACRGL
800	A_37_P204369	5.05E-006	PITPNM1
801	A_37_P065073	5.07E-006	OBFC2B
802	A_37_P077669	5.09E-006	AFMID
803	A_37_P316914	5.10E-006	GLE1
804	A_37_P149676	5.10E-006	OCEL1
805	A_37_P096352	5.11E-006	PTRH1
806	A_37_P018266	5.13E-006	TBCCD1
807	A_37_P162444	5.13E-006	POMZP3
808	A_24_P22939	5.14E-006	ADAMTSL5
809	A_37_P332456	5.15E-006	DCAF11
810	A_37_P121449	5.15E-006	MANEAL
811	A_37_P084192	5.18E-006	SLC25A35
812	A_37_P171253	5.18E-006	TMEM64
813	A_37_P192983	5.18E-006	COL17A1
814	A_37_P291368	5.18E-006	TARBP2
815	A_37_P101107	5.19E-006	C18orf45
816	A_37_P145779	5.19E-006	GATAD2A
817	A_37_P162156	5.20E-006	C7orf25
818	A_37_P339997	5.20E-006	ARL8A
819	A_37_P165427	5.20E-006	ZP3
820	A_37_P232726	5.20E-006	KIAA1191
821	A_37_P090544	5.21E-006	CLIC3
822	A_37_P008711	5.22E-006	TRIM41
823	A_37_P011105	5.22E-006	NDUFAF3
824	A_37_P021529	5.24E-006	TTLL3
825	A_37_P273568	5.24E-006	MAPRE3
826	A_37_P080831	5.28E-006	EIF5A
827	A_37_P133383	5.28E-006	COL9A1
828	A_37_P117322	5.32E-006	DDOST
829	A_37_P125835	5.32E-006	SLC50A1
830	A_37_P132998	5.33E-006	C6orf57
831	A_23_P166051	5.33E-006	RBCK1
832	A_37_P151794	5.34E-006	JOSD2
833	A_37_P122345	5.34E-006	NBL1
834	A_37_P050246	5.34E-006	MTA3
835	A_37_P284442	5.35E-006	PITPNM2
836	A_37_P097839	5.38E-006	SPINK4
837	A_37_P102854	5.40E-006	ASPG
838	A_37_P036434	5.41E-006	C16orf13
839	A_37_P187255	5.41E-006	COMMD7
840	A_37_P191203	5.41E-006	PDRG1
841	A_33_P3382423	5.44E-006	ZNF428
842	A_37_P152411	5.44E-006	KDELR1
843	A_37_P002608	5.44E-006	FBXO4
844	A_37_P285388	5.44E-006	HNRNPA1
845	A_23_P126908	5.45E-006	TNFRSF14
846	A_37_P037764	5.45E-006	YPEL3
847	A_37_P151647	5.46E-006	SLC25A42
848	A_37_P233190	5.47E-006	ZNF346
849	A_37_P298710	5.49E-006	PORCN
850	A_37_P298613	5.49E-006	PLXNA3
851	A_37_P078105	5.49E-006	ASPSCR1
852	A_37_P245479	5.49E-006	VIPR1
853	A_23_P424597	5.49E-006	C19orf25

4183	A_37_P208247	0.00039754	MUC2
4184	A_37_P179091	0.000399	TGM5
4185	A_23_P218675	0.00039955	WFDC2
4186	A_37_P014983	0.00040128	KALRN
4187	A_37_P280923	0.00040136	ACAD10
4188	A_37_P116595	0.00040152	GRHL3
4189	A_23_P129433	0.00040229	SLC9A5
4190	A_37_P155196	0.00040234	ASL
4191	A_37_P098626	0.00040238	UNC13B
4192	A_37_P339450	0.0004025	RPA2
4193	A_37_P120568	0.0004025	ATAD3A
4194	A_37_P262233	0.00040291	SLC12A3
4195	A_37_P147096	0.00040296	ZNF506
4196	A_37_P004682	0.0004032	DNAJC18
4197	A_23_P300740	0.0004032	NPB
4198	A_37_P126045	0.00040357	RCSD1
4199	A_37_P250059	0.0004039	ZFYVE28
4200	A_37_P134780	0.00040426	MLN
4201	A_37_P161738	0.00040539	MYO1G
4202	A_37_P153654	0.00040564	ZNF444
4203	A_37_P039458	0.0004058	SNAI3
4204	A_37_P041401	0.00040594	AOX1
4205	A_37_P293518	0.00040598	PRICKLE3
4206	A_37_P020314	0.00040608	FAM19A4
4207	A_37_P303089	0.00040721	SLC13A5
4208	A_37_P190342	0.00040733	RIN2
4209	A_37_P315599	0.00040741	LCN8
4210	A_37_P249061	0.00040756	SCD5
4211	A_37_P181664	0.00040763	PHF21B
4212	A_37_P122321	0.00040777	NAV1
4213	A_23_P138480	0.00040816	C10orf95
4214	A_33_P3264815	0.00040828	TLN2
4215	A_37_P034354	0.00040841	GPT2
4216	A_37_P164641	0.00040871	KIAA0895
4217	A_33_P3332215	0.00040912	MUC1
4218	A_37_P130934	0.00040954	SLC41A1
4219	A_37_P184063	0.00040987	TAB1
4220	A_33_P3261246	0.00040991	RPL37
4221	A_37_P181454	0.00041064	AIFM3
4222	A_37_P284830	0.00041091	CYP27B1
4223	A_37_P438450	0.00041106	ATP5O
4224	A_37_P108662	0.0004113	STXBP6
4225	A_37_P124243	0.00041156	PIK3CD
4226	A_37_P164314	0.0004116	STX1A
4227	A_37_P146676	0.0004116	ITGB1BP3
4228	A_37_P124934	0.00041161	PPOX
4229	A_37_P261516	0.0004128	PRSS21
4230	A_37_P151594	0.00041282	SIPA1L3
4231	A_37_P029110	0.00041321	SLC25A4
4232	A_37_P110242	0.00041339	ATAD3C
4233	A_37_P110948	0.00041361	COL11A1
4234	A_37_P154300	0.00041417	ZNF91
4235	A_37_P049613	0.0004144	HNRPLL
4236	A_37_P208358	0.00041457	PTPN5
4237	A_24_P215804	0.000415	CKLF
4238	A_37_P257989	0.00041503	PKD1L2
4239	A_37_P234502	0.0004158	BSN
4240	A_37_P102717	0.00041608	AMN
4241	A_37_P293574	0.00041616	GABRA3
4242	A_37_P173032	0.00041661	NIPA1
4243	A_37_P214293	0.00041664	COL18A1

854	A_37_P359158	5.49E-006	HEBP2
855	A_37_P370443	5.49E-006	ATP4A
856	A_37_P310582	5.50E-006	RANGRF
857	A_37_P356157	5.50E-006	TUBE1
858	A_37_P143934	5.54E-006	CDC34
859	A_37_P055271	5.55E-006	KCTD18
860	A_37_P041634	5.55E-006	ASB1
861	A_37_P242508	5.57E-006	IMPDH2
862	A_33_P3411632	5.58E-006	TMEM121
863	A_37_P031247	5.60E-006	Sep-01
864	A_37_P121744	5.60E-006	MIB2
865	A_37_P199580	5.61E-006	SFXN3
866	A_37_P019979	5.61E-006	TMEM110-MUSTN1
867	A_37_P226961	5.65E-006	GRM6
868	A_37_P403949	5.66E-006	SQRDL
869	A_37_P081205	5.67E-006	FAM57A
870	A_37_P083503	5.67E-006	MAP2K3
871	A_37_P167579	5.67E-006	NSMAF
872	A_37_P058533	5.68E-006	RNF41
873	A_33_P3283971	5.69E-006	NFKBIL1
874	A_37_P338761	5.69E-006	EPHX1
875	A_37_P212358	5.70E-006	STX5
876	A_37_P128715	5.70E-006	TATDN3
877	A_37_P034140	5.70E-006	GFER
878	A_37_P237644	5.73E-006	GLYCTK
879	A_37_P119164	5.73E-006	YIPF1
880	A_37_P418614	5.73E-006	PITX3
881	A_37_P088699	5.73E-006	THRA
882	A_37_P143369	5.75E-006	C19orf50
883	A_37_P404651	5.75E-006	TYRO3
884	A_37_P262063	5.77E-006	FAM100A
885	A_37_P189038	5.78E-006	TNNC2
886	A_37_P151916	5.80E-006	SPHK2
887	A_37_P144707	5.80E-006	DDA1
888	A_37_P086318	5.80E-006	PDK2
889	A_37_P303901	5.80E-006	ST6GALNAC2
890	A_23_P78835	5.80E-006	ZNF787
891	A_37_P345705	5.82E-006	ECHDC2
892	A_37_P256661	5.82E-006	GFOD2
893	A_37_P327868	5.83E-006	SETD3
894	A_37_P153661	5.85E-006	ZNF446
895	A_37_P243611	5.85E-006	SENP2
896	A_37_P037447	5.88E-006	ZNF598
897	A_37_P163837	5.90E-006	STARD3NL
898	A_37_P387475	5.90E-006	TMEM120A
899	A_37_P087751	5.94E-006	SLC26A11
900	A_37_P145261	5.94E-006	AP2S1
901	A_37_P172278	5.96E-006	TNFRSF10C
902	A_37_P430050	5.97E-006	BAD
903	A_37_P352740	5.98E-006	THAP3
904	A_37_P051455	5.99E-006	C2orf68
905	A_33_P3369452	6.01E-006	C22orf25
906	A_37_P264674	6.01E-006	AGBL5
907	A_37_P143044	6.03E-006	AXL
908	A_37_P151716	6.04E-006	SLC44A2
909	A_37_P149690	6.05E-006	PAFAH1B3
910	A_37_P234726	6.07E-006	C3orf23
911	A_37_P158600	6.08E-006	IFRD1
912	A_37_P125747	6.08E-006	ETNK2
913	A_37_P204836	6.08E-006	PGAP2
914	A_37_P201726	6.08E-006	CLNS1A

4244	A_37_P204802	0.00041685	FOLR3
4245	A_37_P081697	0.00041719	TEX2
4246	A_37_P077134	0.00041719	ZNF673
4247	A_37_P369323	0.00041719	PPM1N
4248	A_23_P75430	0.00041751	C11orf75
4249	A_37_P419261	0.00041775	HK1
4250	A_37_P335528	0.00041802	CACYBP
4251	A_23_P67151	0.00041826	OLFM2
4252	A_37_P032881	0.00042065	CORO7
4253	A_37_P255973	0.00042066	GALNS
4254	A_37_P363954	0.00042118	SCUBE3
4255	A_37_P165348	0.00042135	ZNF775
4256	A_37_P193678	0.00042192	CELF2
4257	A_37_P087094	0.00042354	RND2
4258	A_32_P191527	0.00042487	SETD8
4259	A_37_P123369	0.00042506	PADI2
4260	A_37_P424132	0.00042506	UNC5B
4261	A_37_P065552	0.00042543	PLBD2
4262	A_37_P326425	0.00042549	KLHL33
4263	A_37_P212551	0.00042703	UPK2
4264	A_37_P135170	0.00042703	GPR126
4265	A_37_P375391	0.00042722	SLC27A1
4266	A_37_P125121	0.00042724	PRDM2
4267	A_37_P142756	0.00042824	KLK7
4268	A_37_P015531	0.00042883	LNP1
4269	A_37_P000283	0.00042901	FLT4
4270	A_37_P198539	0.00042945	PFKFB3
4271	A_37_P032507	0.00043037	CDH15
4272	A_37_P235716	0.00043098	CP
4273	A_37_P039720	0.00043124	TSC2
4274	A_37_P369518	0.00043154	PRSSL1
4275	A_37_P043330	0.00043171	PIGF
4276	A_37_P275497	0.00043298	PLCD4
4277	A_33_P3423931	0.00043322	LCN9
4278	A_37_P351478	0.00043341	SLC25A44
4279	A_37_P255042	0.00043348	ABCC11
4280	A_37_P419422	0.00043373	SYCE1
4281	A_37_P436428	0.00043424	VWA5A
4282	A_37_P105560	0.00043553	SFTA3
4283	A_37_P054791	0.00043611	PDE11A
4284	A_37_P208881	0.0004384	ODF3
4285	A_33_P3260451	0.00043893	USP17L2
4286	A_23_P345564	0.00043898	OPRL1
4287	A_37_P096301	0.00043929	EPB41L4B
4288	A_37_P430811	0.00043945	BTBD10
4289	A_37_P169442	0.00043945	BRF2
4290	A_24_P849801	0.00043962	RPL22
4291	A_37_P198682	0.00044112	PLCE1
4292	A_37_P069657	0.00044128	BCORL1
4293	A_37_P119974	0.00044151	PLEKHG5
4294	A_37_P227716	0.00044177	HAVCR1
4295	A_37_P298944	0.00044177	REPS2
4296	A_33_P3429864	0.00044212	EPN2
4297	A_37_P135944	0.00044262	IRF4
4298	A_37_P124758	0.00044343	EPS8L3
4299	A_37_P158740	0.00044397	IRF5
4300	A_37_P041510	0.0004447	ARHGAP25
4301	A_37_P181914	0.0004459	SLC5A4
4302	A_24_P30923	0.00044648	SNN
4303	A_37_P388237	0.00044695	TSPAN13
4304	A_23_P54041	0.00044716	THTPA

915	A_37_P080320	6.10E-006	PROCA1
916	A_37_P170866	6.12E-006	PPP1R16A
917	A_37_P142229	6.12E-006	ZNF76
918	A_37_P084200	6.12E-006	SLC25A39
919	A_33_P3257222	6.14E-006	COMTD1
920	A_37_P011219	6.14E-006	CACNA2D3
921	A_37_P205250	6.15E-006	NUDT8
922	A_37_P150781	6.16E-006	RAB4B
923	A_37_P018357	6.17E-006	C3orf21
924	A_23_P119714	6.21E-006	C19orf62
925	A_37_P244397	6.21E-006	TADA3
926	A_23_P94434	6.21E-006	HRCT1
927	A_37_P200349	6.22E-006	PDLIM1
928	A_24_P329353	6.24E-006	ORAI2
929	A_33_P3405966	6.25E-006	ST3GAL3
930	A_37_P182461	6.27E-006	SBF1
931	A_37_P275938	6.27E-006	PQLC3
932	A_37_P150805	6.27E-006	RAD23A
933	A_37_P000646	6.27E-006	B4GALT7
934	A_37_P039376	6.28E-006	THOC6
935	A_23_P200396	6.29E-006	C1orf91
936	A_37_P063737	6.29E-006	LOH12CR1
937	A_37_P149435	6.31E-006	ICAM3
938	A_37_P288991	6.33E-006	PCBP2
939	A_37_P182972	6.33E-006	GAS2L1
940	A_33_P3374623	6.33E-006	ABCA7
941	A_37_P321221	6.33E-006	SH3GL2
942	A_37_P212661	6.34E-006	SIRT3
943	A_37_P366950	6.35E-006	FUZ
944	A_37_P178470	6.39E-006	SPG21
945	A_37_P332111	6.39E-006	TRMT61A
946	A_33_P3414683	6.39E-006	KIFC2
947	A_37_P067965	6.41E-006	TMEM106C
948	A_37_P188305	6.41E-006	IFT52
949	A_37_P110969	6.42E-006	MECR
950	A_37_P097142	6.44E-006	AK8
951	A_23_P64630	6.44E-006	RNF26
952	A_37_P060982	6.46E-006	MMAB
953	A_37_P049744	6.47E-006	DTNB
954	A_37_P022152	6.47E-006	ZNF639
955	A_37_P308417	6.47E-006	MAP3K3
956	A_37_P038246	6.53E-006	ITFG1
957	A_37_P095405	6.54E-006	EDF1
958	A_37_P122946	6.54E-006	C1orf52
959	A_37_P066228	6.55E-006	RDH5
960	A_37_P275231	6.55E-006	C2orf81
961	A_37_P195529	6.57E-006	HHEX
962	A_37_P083444	6.57E-006	BECN1
963	A_37_P434361	6.60E-006	SCYL1
964	A_37_P184779	6.60E-006	C22orf40
965	A_37_P360281	6.62E-006	C6orf35
966	A_37_P148568	6.62E-006	MAST1
967	A_37_P081668	6.62E-006	TLCD1
968	A_23_P6413	6.62E-006	SELM
969	A_37_P094932	6.63E-006	WDR5
970	A_37_P047098	6.65E-006	KCNIP3
971	A_37_P149248	6.66E-006	C19orf70
972	A_37_P350397	6.66E-006	FABP3
973	A_37_P009710	6.66E-006	ABHD6
974	A_37_P421990	6.67E-006	OIT3
975	A_37_P018343	6.69E-006	C3orf17

4305	A_37_P431372	0.00044725	LRP5
4306	A_37_P179189	0.00044782	INO80
4307	A_23_P47168	0.00044782	FLRT1
4308	A_37_P015640	0.00044823	MST1R
4309	A_37_P193328	0.00044846	CDH23
4310	A_37_P016263	0.0004486	SCN5A
4311	A_37_P293780	0.00044978	L1CAM
4312	A_37_P435551	0.00044981	TECTA
4313	A_37_P113901	0.00044988	DCST1
4314	A_37_P183361	0.00044996	PLA2G6
4315	A_37_P051076	0.00045062	IFT172
4316	A_37_P002860	0.00045117	GABRP
4317	A_37_P023285	0.00045242	GABRA2
4318	A_37_P183148	0.00045345	ARSA
4319	A_37_P106915	0.00045396	PLEKHH1
4320	A_37_P082743	0.00045414	KIF19
4321	A_37_P099337	0.00045491	ATP9B
4322	A_37_P057415	0.00045513	RASAL1
4323	A_37_P153088	0.00045552	UPK1A
4324	A_37_P188473	0.00045666	ATP9A
4325	A_37_P190705	0.00045701	SLC9A8
4326	A_33_P3309911	0.00045795	PRAMEF5
4327	A_37_P018484	0.00045806	PLA1A
4328	A_37_P152112	0.00045926	SIGLEC14
4329	A_37_P111006	0.00045948	C1orf101
4330	A_37_P238120	0.00046112	HRASLS
4331	A_37_P291518	0.00046126	TENC1
4332	A_37_P191031	0.0004616	TGM3
4333	A_37_P087458	0.0004619	ITGAE
4334	A_37_P326210	0.00046271	MBD1
4335	A_37_P127989	0.00046273	SLC5A9
4336	A_37_P394014	0.00046343	NKAIN3
4337	A_37_P243722	0.00046409	ARHGEF26
4338	A_37_P066949	0.00046419	SLC25A3
4339	A_37_P160298	0.0004642	PLOD3
4340	A_37_P166245	0.00046479	C8orf40
4341	A_37_P240866	0.00046526	MLF1
4342	A_37_P093145	0.00046587	ARID3C
4343	A_37_P093408	0.00046594	GRIN3A
4344	A_37_P340290	0.00046599	GPX7
4345	A_37_P425735	0.00046622	BEST1
4346	A_37_P162945	0.00046669	CAMK2B
4347	A_37_P059093	0.00046669	LALBA
4348	A_37_P162604	0.00046696	LRCH4
4349	A_37_P152533	0.0004676	TMEM145
4350	A_37_P047377	0.00046766	KRTCAP3
4351	A_37_P288194	0.00046929	EFCAB4B
4352	A_37_P054835	0.00047015	TNS1
4353	A_37_P276541	0.00047019	PAX8
4354	A_24_P347378	0.00047085	ALOX5AP
4355	A_37_P188239	0.0004713	HNF4A
4356	A_37_P055053	0.00047209	LYPD1
4357	A_33_P3685216	0.00047246	A1BG
4358	A_33_P3266520	0.00047255	SPPL2B
4359	A_37_P169884	0.00047305	MAL2
4360	A_37_P025680	0.00047346	IDUA
4361	A_37_P190131	0.00047559	PROCR
4362	A_37_P219623	0.00047608	SERP2
4363	A_37_P136636	0.00047617	ZC3H12D
4364	A_37_P002965	0.00047736	ARSI
4365	A_33_P3422466	0.00047748	MORN3

976	A_23_P92349	6.71E-006	FGFRL1
977	A_37_P095521	6.72E-006	SH3GLB2
978	A_37_P186971	6.84E-006	SNX5
979	A_32_P210642	6.84E-006	EGFL7
980	A_37_P199448	6.85E-006	PCGF6
981	A_23_P154585	6.86E-006	SNX21
982	A_37_P152204	6.87E-006	SYDE1
983	A_37_P007689	6.88E-006	SLC22A5
984	A_37_P185384	6.88E-006	SGSM3
985	A_23_P47116	6.89E-006	RASSF7
986	A_37_P090599	6.89E-006	TSC1
987	A_37_P144026	6.90E-006	MEIS3
988	A_37_P069044	6.95E-006	CHFR
989	A_37_P185253	6.95E-006	FAM116B
990	A_23_P123454	6.96E-006	NUDT18
991	A_37_P085896	6.97E-006	ICAM2
992	A_33_P3243449	6.99E-006	CD70
993	A_37_P183922	7.02E-006	HIRA
994	A_37_P105769	7.02E-006	LRP10
995	A_37_P138032	7.04E-006	MRS2
996	A_37_P186518	7.04E-006	PREX1
997	A_37_P089601	7.05E-006	CENPV
998	A_37_P333051	7.11E-006	S100A16
999	A_37_P212701	7.12E-006	MAP4K2
1000	A_37_P202745	7.13E-006	CCDC84
1001	A_37_P063186	7.16E-006	ZNF384
1002	A_37_P153728	7.18E-006	ZNF530
1003	A_37_P153308	7.22E-006	WTIP
1004	A_37_P320421	7.24E-006	C9orf116
1005	A_37_P156050	7.29E-006	GATSL1
1006	A_37_P032561	7.30E-006	CDT1
1007	A_37_P082834	7.30E-006	ATXN7L3
1008	A_37_P047702	7.32E-006	OBSL1
1009	A_37_P190893	7.33E-006	SS18L1
1010	A_37_P431089	7.33E-006	C11orf24
1011	A_37_P144281	7.34E-006	CNN2
1012	A_37_P118747	7.37E-006	LDLRAP1
1013	A_37_P216855	7.39E-006	TMTC4
1014	A_37_P399304	7.39E-006	RBPMS2
1015	A_37_P112365	7.41E-006	CDA
1016	A_37_P165150	7.41E-006	ZMIZ2
1017	A_32_P150856	7.43E-006	LOC407835
1018	A_37_P372358	7.44E-006	MCOLN1
1019	A_37_P399300	7.46E-006	GCHFR
1020	A_37_P111122	7.47E-006	C1orf128
1021	A_37_P230509	7.47E-006	ERCC8
1022	A_37_P187411	7.48E-006	CRLS1
1023	A_37_P023708	7.50E-006	CCNG2
1024	A_37_P035084	7.50E-006	AXIN1
1025	A_37_P086400	7.50E-006	PITPNC1
1026	A_37_P259148	7.53E-006	BCL7C
1027	A_37_P087767	7.53E-006	SLC2A4
1028	A_37_P312488	7.53E-006	RAD51L3
1029	A_37_P207890	7.54E-006	MDK
1030	A_23_P124619	7.57E-006	S100A14
1031	A_37_P313237	7.57E-006	USP6
1032	A_37_P308756	7.63E-006	C17orf101
1033	A_37_P364747	7.64E-006	TBC1D22B
1034	A_37_P301602	7.66E-006	ANKRD13B
1035	A_37_P094336	7.67E-006	ZDHHC12
1036	A_23_P139998	7.67E-006	UBAC2

4366	A_37_P013533	0.00047807	FBLN2
4367	A_37_P024794	0.00047815	FGB
4368	A_37_P059986	0.00047815	GLS2
4369	A_37_P366344	0.00047816	KLK15
4370	A_37_P338128	0.00047861	SLC35E2B
4371	A_37_P090095	0.00047966	ABCA1
4372	A_37_P031026	0.00048062	ACSM2B
4373	A_37_P398363	0.00048194	CYP1A2
4374	A_37_P109157	0.00048252	SLAMF9
4375	A_37_P325708	0.00048298	TAF4B
4376	A_37_P031432	0.00048334	A_37_P031432
4377	A_37_P360209	0.00048358	B3GAT2
4378	A_37_P425102	0.00048381	YPEL4
4379	A_37_P061900	0.00048492	SLC24A6
4380	A_37_P071348	0.00048505	CXCR3
4381	A_37_P126513	0.0004859	ENST00000471420
4382	A_37_P143200	0.0004861	CELF5
4383	A_37_P210918	0.00048659	SLC22A20
4384	A_37_P265253	0.00048661	TUBA4A
4385	A_37_P394835	0.00048666	C8orf80
4386	A_37_P382204	0.00048829	HECW1
4387	A_37_P372719	0.00048892	MYH14
4388	A_37_P317961	0.00048923	LHX2
4389	A_23_P137173	0.00049014	TMSB15A
4390	A_37_P362200	0.00049016	RPS10
4391	A_33_P3399263	0.00049018	IL15RA
4392	A_37_P095153	0.0004922	LRSAM1
4393	A_37_P302637	0.00049264	C1QTNF1
4394	A_37_P191074	0.00049276	TM9SF4
4395	A_37_P266809	0.00049487	CHRND
4396	A_37_P094186	0.00049493	LAMC3
4397	A_37_P002089	0.00049549	DOCK2
4398	A_37_P209015	0.00049579	INS-IGF2
4399	A_37_P181770	0.00049605	C22orf32
4400	A_37_P156325	0.00049666	CLCN1
4401	A_37_P393985	0.00049688	EIF3E
4402	A_37_P183541	0.00049851	LIMK2
4403	A_37_P001762	0.00049964	CYFIP2
4404	A_37_P374865	0.00049983	RPS19
4405	A_37_P200515	0.00050025	VENTX
4406	A_37_P127140	0.00050157	SEC16B
4407	A_37_P358904	0.00050185	TREM2
4408	A_37_P052571	0.00050197	R3HDM1
4409	A_37_P148967	0.00050326	MYBPC2
4410	A_37_P043926	0.00050345	DBI
4411	A_37_P111501	0.0005041	C1orf92
4412	A_37_P017447	0.0005045	HYAL1
4413	A_37_P185994	0.00050528	CELSR1
4414	A_37_P186310	0.00050537	ACSS2
4415	A_37_P186269	0.00050631	ENST00000468532
4416	A_37_P191157	0.0005068	TP53INP2
4417	A_37_P423744	0.00050684	KCNMA1
4418	A_37_P020809	0.00050692	KCNMB3
4419	A_23_P118722	0.00050742	ASGR1
4420	A_37_P429125	0.00050778	HIPK3
4421	A_37_P339182	0.0005081	FAM76A
4422	A_33_P3308512	0.00050811	SLC16A10
4423	A_37_P069942	0.00050817	XKRX
4424	A_37_P364039	0.0005086	T
4425	A_37_P038279	0.00051021	FA2H
4426	A_37_P131666	0.00051084	SLC44A4

1037	A_37_P091496	7.68E-006	UBAC1
1038	A_37_P432163	7.69E-006	NADSYN1
1039	A_37_P334944	7.75E-006	C1orf182
1040	A_37_P066128	7.75E-006	SDS
1041	A_37_P163916	7.75E-006	RABL5
1042	A_37_P197629	7.78E-006	HPS1
1043	A_37_P430583	7.79E-006	ZNF259
1044	A_37_P084320	7.79E-006	METRNL
1045	A_37_P314360	7.83E-006	ATP6V1G1
1046	A_37_P196710	7.83E-006	BLOC1S2
1047	A_37_P185118	7.84E-006	THAP7
1048	A_37_P414068	7.86E-006	CABLES2
1049	A_37_P074853	7.86E-006	PLP2
1050	A_37_P392671	7.93E-006	ZNF250
1051	A_37_P031394	7.93E-006	LOC100132247
1052	A_23_P80382	7.93E-006	PRR5
1053	A_24_P80204	7.93E-006	MALL
1054	A_37_P140307	7.99E-006	THBS2
1055	A_37_P056446	8.05E-006	WASH1
1056	A_32_P198325	8.09E-006	C22orf36
1057	A_37_P068426	8.11E-006	CDK2AP1
1058	A_37_P058168	8.13E-006	WSB2
1059	A_37_P067711	8.13E-006	VPS37B
1060	A_37_P316264	8.13E-006	ANKS6
1061	A_37_P080264	8.14E-006	CRLF3
1062	A_37_P351640	8.15E-006	FAM176B
1063	A_37_P431424	8.16E-006	LRTOMT
1064	A_37_P098490	8.16E-006	RALGDS
1065	A_37_P247843	8.16E-006	DCK
1066	A_37_P243123	8.19E-006	UBP1
1067	A_37_P328505	8.19E-006	INF2
1068	A_37_P039330	8.20E-006	KCTD13
1069	A_37_P001292	8.23E-006	CCNB1
1070	A_37_P149740	8.23E-006	ELOF1
1071	A_37_P374485	8.24E-006	ETHE1
1072	A_37_P374473	8.24E-006	RAB11B
1073	A_37_P370925	8.25E-006	B9D2
1074	A_32_P149174	8.28E-006	C6orf226
1075	A_37_P115730	8.30E-006	FLAD1
1076	A_37_P109351	8.31E-006	ADC
1077	A_37_P168967	8.32E-006	ZNF251
1078	A_37_P168535	8.34E-006	GTF2E2
1079	A_37_P302144	8.34E-006	BIRC5
1080	A_37_P110024	8.34E-006	ARHGEF10L
1081	A_37_P140727	8.34E-006	FAM65B
1082	A_37_P411922	8.36E-006	HCK
1083	A_37_P110534	8.36E-006	ATP8B2
1084	A_37_P189782	8.37E-006	PABPC1L
1085	A_37_P084015	8.41E-006	PLEKHH3
1086	A_32_P831181	8.44E-006	BRI3BP
1087	A_37_P146050	8.45E-006	GRAMD1A
1088	A_23_P68922	8.46E-006	MICALL1
1089	A_37_P019271	8.46E-006	QARS
1090	A_37_P172568	8.47E-006	SLC39A4
1091	A_37_P114931	8.51E-006	CELA2A
1092	A_37_P033949	8.51E-006	PKD1
1093	A_37_P266721	8.55E-006	CENPA
1094	A_37_P360078	8.55E-006	MRPS18A
1095	A_37_P257634	8.58E-006	PRSS36
1096	A_37_P118295	8.59E-006	ATPAF1
1097	A_37_P187241	8.59E-006	CDS2

4427	A_37_P187817	0.0005109	EBF4
4428	A_37_P121168	0.00051092	LRRC42
4429	A_37_P313481	0.00051112	ZACN
4430	A_37_P173197	0.00051212	SEMA7A
4431	A_37_P440320	0.00051265	C13orf30
4432	A_37_P155575	0.00051299	TECPR1
4433	A_37_P102563	0.00051365	ABHD12B
4434	A_37_P151381	0.0005137	SCAMP4
4435	A_37_P046408	0.00051408	SLC23A3
4436	A_37_P037532	0.00051421	PLA2G15
4437	A_37_P153269	0.00051451	WDR62
4438	A_37_P301911	0.00051511	SLC47A2
4439	A_37_P061548	0.00051577	DCP1B
4440	A_37_P175427	0.00051634	SCAMP2
4441	A_37_P038624	0.00051643	SLC5A11
4442	A_37_P020493	0.00051683	LTF
4443	A_23_P433229	0.00051749	PHYHIP
4444	A_37_P099917	0.00051772	ST8SIA5
4445	A_37_P215777	0.00051802	RSPH1
4446	A_37_P183700	0.00051859	BID
4447	A_37_P015933	0.00051876	VGLL4
4448	A_33_P3328450	0.00051934	HMX1
4449	A_24_P228796	0.00051951	GAGE7
4450	A_37_P164064	0.00052	ENST00000447661
4451	A_37_P179766	0.00052006	SEMA4B
4452	A_37_P142499	0.00052091	ALDH16A1
4453	A_37_P141004	0.00052229	TAF8
4454	A_37_P039066	0.00052286	SULT1A4
4455	A_37_P044903	0.00052305	EPCAM
4456	A_32_P110872	0.00052442	A2LD1
4457	A_37_P026441	0.00052479	BBS7
4458	A_37_P128474	0.00052568	CCDC23
4459	A_37_P201587	0.0005266	AQP11
4460	A_37_P220025	0.00052774	THSD1
4461	A_37_P407985	0.00052845	MPPED1
4462	A_37_P441876	0.00052943	TRPC4
4463	A_37_P189029	0.00052967	PTGIS
4464	A_37_P179099	0.00052994	TGM7
4465	A_37_P153702	0.0005305	ZNF493
4466	A_37_P204169	0.00053097	EIF3M
4467	A_37_P167864	0.00053157	ARC
4468	A_37_P115959	0.00053287	ARHGAP30
4469	A_37_P082179	0.0005329	MPO
4470	A_37_P308852	0.00053337	OSBPL7
4471	A_37_P015611	0.00053367	MST1
4472	A_37_P433108	0.00053529	PGA5
4473	A_37_P186784	0.00053558	C20orf160
4474	A_37_P219913	0.00053633	TNFSF11
4475	A_37_P114124	0.00053709	CSF3R
4476	A_37_P302101	0.00053813	BCAS3
4477	A_37_P164046	0.00053819	TBXAS1
4478	A_37_P108306	0.00053836	TMEM63C
4479	A_37_P142973	0.00053901	RASGRP4
4480	A_37_P210365	0.00053988	PC
4481	A_37_P210711	0.00054135	LRRC32
4482	A_37_P061085	0.00054143	SPPL3
4483	A_32_P88719	0.00054162	RAB40AL
4484	A_37_P050032	0.00054198	C2orf88
4485	A_37_P159916	0.00054205	LOC154761
4486	A_37_P120131	0.00054239	HEYL
4487	A_37_P301088	0.00054298	ACAP1

1098	A_37_P087415	8.61E-006	FAM104A
1099	A_37_P054959	8.62E-006	STRADB
1100	A_37_P056266	8.63E-006	RNASEH1
1101	A_37_P101499	8.63E-006	PSMG2
1102	A_37_P252214	8.63E-006	INTS12
1103	A_37_P328786	8.63E-006	KLHDC2
1104	A_37_P241765	8.63E-006	P4HTM
1105	A_37_P036862	8.73E-006	HYDIN
1106	A_37_P087269	8.75E-006	NAT9
1107	A_37_P001952	8.76E-006	ANKRA2
1108	A_37_P023670	8.78E-006	CCDC109B
1109	A_37_P215099	8.78E-006	DSCR3
1110	A_37_P012289	8.81E-006	CTDSPL
1111	A_23_P210708	8.83E-006	SIRPA
1112	A_37_P248961	8.84E-006	GRK4
1113	A_37_P292273	8.84E-006	NELL2
1114	A_37_P195114	8.84E-006	MMS19
1115	A_23_P428842	8.85E-006	TMEM44
1116	A_37_P305978	8.86E-006	IGFBP4
1117	A_37_P049472	8.87E-006	WBP1
1118	A_37_P189947	8.87E-006	PIGT
1119	A_37_P152484	8.88E-006	TCF3
1120	A_37_P113481	8.89E-006	CROCC
1121	A_37_P013923	8.91E-006	GXYLT2
1122	A_37_P287426	8.96E-006	C12orf49
1123	A_37_P176800	8.96E-006	MTFMT
1124	A_37_P151759	8.96E-006	LSM7
1125	A_37_P067169	8.97E-006	SMARCD1
1126	A_37_P066072	8.98E-006	RAB5B
1127	A_37_P241046	8.98E-006	MTMR14
1128	A_37_P127594	8.99E-006	LRRC41
1129	A_37_P079552	9.02E-006	TRAPPC1
1130	A_37_P150841	9.03E-006	ZNF414
1131	A_37_P045287	9.04E-006	MMADHC
1132	A_37_P031709	9.07E-006	NOB1
1133	A_37_P061652	9.08E-006	DCTN2
1134	A_37_P388664	9.08E-006	WIPI2
1135	A_37_P120552	9.08E-006	TMEM167B
1136	A_37_P170182	9.15E-006	MYOM2
1137	A_37_P408599	9.16E-006	POLR3H
1138	A_37_P439874	9.18E-006	RWDD2B
1139	A_33_P3209895	9.19E-006	FAM58A
1140	A_37_P103208	9.23E-006	C14orf50
1141	A_37_P035116	9.24E-006	HCFC1R1
1142	A_37_P303568	9.25E-006	GGA3
1143	A_37_P076608	9.28E-006	PPP2R3B
1144	A_33_P3414591	9.29E-006	TWIST1
1145	A_37_P149647	9.29E-006	ZNF561
1146	A_37_P322210	9.30E-006	UBE2R2
1147	A_37_P053750	9.30E-006	CAPN13
1148	A_37_P144149	9.38E-006	CIRBP
1149	A_37_P187588	9.39E-006	DBNDD2
1150	A_37_P302815	9.40E-006	CACNA1G
1151	A_23_P50946	9.41E-006	RAMP1
1152	A_37_P044326	9.42E-006	DNAJB2
1153	A_23_P406131	9.43E-006	TMEM159
1154	A_37_P012214	9.49E-006	CRTAP
1155	A_37_P140615	9.51E-006	SMPD2
1156	A_37_P084028	9.52E-006	PLEKHM1
1157	A_23_P369471	9.53E-006	LCE3A
1158	A_37_P157532	9.53E-006	GATS

4488	A_37_P071637	0.00054307	GAGE12J
4489	A_37_P254566	0.00054436	RBFOX1
4490	A_37_P178193	0.00054504	C15orf29
4491	A_37_P090792	0.00054548	BSPRY
4492	A_37_P207704	0.00054777	LRRC56
4493	A_37_P387959	0.00054886	TMEM213
4494	A_37_P232078	0.00054934	THBS4
4495	A_23_P303203	0.00054969	CHMP7
4496	A_37_P127761	0.00054981	SLC25A34
4497	A_37_P147850	0.00055246	DNASE2
4498	A_37_P141459	0.00055316	SIM1
4499	A_37_P079930	0.00055329	ALOX15
4500	A_37_P135694	0.00055446	ATF6B
4501	A_37_P118589	0.00055513	DHRS3
4502	A_37_P088894	0.00055552	TMEM132E
4503	A_37_P037441	0.00055574	TPPP3
4504	A_37_P059816	0.00055602	DDX11
4505	A_37_P202178	0.00055632	C11orf41
4506	A_24_P916718	0.00055697	ZNF467
4507	A_37_P091920	0.00055703	CRB2
4508	A_37_P366903	0.00055756	BRSK1
4509	A_37_P421488	0.00055872	EBF3
4510	A_37_P033844	0.00055875	FBRS
4511	A_37_P038041	0.00055921	EXOC3L
4512	A_37_P138480	0.0005619	NT5E
4513	A_37_P157263	0.00056197	EMID2
4514	A_37_P141532	0.00056224	STX7
4515	A_37_P173614	0.00056261	SLC12A6
4516	A_37_P078502	0.00056287	KRT19
4517	A_37_P110114	0.00056329	TTC39A
4518	A_37_P212008	0.00056336	TMEM136
4519	A_23_P125772	0.00056466	SRPK3
4520	A_37_P437767	0.00056545	TIAM1
4521	A_37_P209880	0.00056737	PVRL1
4522	A_37_P195151	0.00056773	GLRX3
4523	A_37_P381117	0.00056798	TSGA13
4524	A_37_P276866	0.00056814	RPL37A
4525	A_37_P134889	0.00056902	FNDC1
4526	A_37_P133505	0.00056983	CFB
4527	A_37_P150619	0.00057082	PRR12
4528	A_37_P280229	0.00057331	FIGLA
4529	A_37_P081952	0.00057421	GSDMA
4530	A_37_P210079	0.00057445	SYT7
4531	A_23_P78099	0.00057481	VTN
4532	A_37_P340454	0.00057529	ENST00000469973
4533	A_37_P434596	0.00057533	SLC22A12
4534	A_33_P3399875	0.00057627	C20orf144
4535	A_37_P137065	0.00057633	PSORS1C2
4536	A_37_P416085	0.00057761	ATRNL1
4537	A_37_P356653	0.00057825	C6orf223
4538	A_37_P009774	0.00057825	SENP7
4539	A_37_P115197	0.00057901	FAAH
4540	A_37_P296353	0.00057986	DGKK
4541	A_37_P121818	0.00058035	MMP23B
4542	A_37_P173928	0.00058054	C15orf60
4543	A_37_P146708	0.00058208	DENND1C
4544	A_37_P132003	0.00058285	MDGA1
4545	A_37_P098964	0.00058433	FKBP15
4546	A_37_P119383	0.00058452	VAMP4
4547	A_37_P406409	0.00058496	PISD
4548	A_23_P434289	0.00058578	GPR62

1159	A_37_P037680	9.53E-006	POLR3E
1160	A_24_P179013	9.55E-006	FAM136BP
1161	A_37_P362914	9.55E-006	C6orf129
1162	A_37_P034143	9.61E-006	NTAN1
1163	A_37_P089404	9.63E-006	FDXR
1164	A_23_P258418	9.64E-006	TNIP2
1165	A_33_P3393408	9.64E-006	DECR2
1166	A_37_P143530	9.65E-006	ADCK4
1167	A_37_P032551	9.68E-006	CDK10
1168	A_37_P148933	9.68E-006	EEF2
1169	A_37_P179210	9.71E-006	PARP6
1170	A_37_P134648	9.71E-006	FBXO9
1171	A_37_P057560	9.73E-006	PHC1
1172	A_23_P411102	9.78E-006	DLG4
1173	A_37_P146117	9.78E-006	DBP
1174	A_37_P211894	9.79E-006	TRPT1
1175	A_37_P142995	9.83E-006	ATP5D
1176	A_37_P155059	9.85E-006	AQP1
1177	A_33_P3420816	9.85E-006	GDF1
1178	A_37_P095070	9.85E-006	TRIM14
1179	A_37_P007765	9.86E-006	SLC30A5
1180	A_37_P144455	9.89E-006	SARS2
1181	A_23_P390596	9.89E-006	PSKH1
1182	A_37_P087109	9.91E-006	RNF135
1183	A_37_P211954	9.92E-006	TM7SF2
1184	A_23_P337729	9.92E-006	TMEM180
1185	A_37_P157089	9.93E-006	DUS4L
1186	A_37_P205201	9.93E-006	GTF2H1
1187	A_37_P328739	9.94E-006	KIF26A
1188	A_37_P174562	9.95E-006	CSK
1189	A_37_P153091	9.96E-006	USE1
1190	A_23_P80839	9.98E-006	MAP6D1
1191	A_33_P3387050	1.00E-005	C8orf82
1192	A_37_P043718	1.00E-005	ALS2CR4
1193	A_37_P214662	1.00E-005	IFNGR2
1194	A_37_P037756	1.00E-005	PRKCB
1195	A_37_P149119	1.00E-005	C19orf42
1196	A_37_P006535	1.01E-005	C5orf13
1197	A_37_P404369	1.01E-005	TMEM62
1198	A_32_P152195	1.01E-005	STAC2
1199	A_37_P372832	1.01E-005	C19orf43
1200	A_33_P3350374	1.01E-005	C10orf58
1201	A_23_P320021	1.01E-005	TULP1
1202	A_37_P356734	1.02E-005	C6orf72
1203	A_37_P151617	1.02E-005	RAB3D
1204	A_37_P009716	1.02E-005	ABTB1
1205	A_37_P086022	1.02E-005	C1QL1
1206	A_37_P071644	1.02E-005	GAGE2B
1207	A_37_P406399	1.02E-005	LMF2
1208	A_37_P110085	1.02E-005	ARHGEF16
1209	A_37_P287916	1.02E-005	MFSD5
1210	A_37_P032071	1.02E-005	C16orf68
1211	A_37_P186182	1.02E-005	CHCHD10
1212	A_37_P106655	1.02E-005	PAPLN
1213	A_37_P374795	1.02E-005	TBXA2R
1214	A_37_P259399	1.03E-005	LOC342346
1215	A_37_P022101	1.03E-005	NGLY1
1216	A_37_P084649	1.03E-005	CTDNEP1
1217	A_37_P393477	1.03E-005	DSCC1
1218	A_37_P431360	1.03E-005	ORAOV1
1219	A_37_P441633	1.03E-005	DHRS12

4549	A_37_P432031	0.00058767	SCT
4550	A_37_P188537	0.00058789	L3MBTL1
4551	A_37_P196322	0.00058855	DIP2C
4552	A_37_P410895	0.00058864	CDH4
4553	A_37_P138080	0.00058941	EEF1A1
4554	A_37_P330851	0.00058949	PTGER2
4555	A_37_P171247	0.00059134	PYCRL
4556	A_37_P032445	0.00059151	CD19
4557	A_37_P389428	0.00059165	ADCK5
4558	A_37_P022516	0.00059246	PRDM5
4559	A_37_P071054	0.00059282	EFNB1
4560	A_37_P253314	0.00059399	TACC3
4561	A_37_P276687	0.00059612	RNF144A
4562	A_37_P411020	0.00059616	TASP1
4563	A_37_P274160	0.00059654	MYO7B
4564	A_37_P153788	0.00059729	ZNF549
4565	A_37_P325533	0.00059773	SLC14A2
4566	A_37_P211726	0.000598	TBC1D10C
4567	A_33_P3291796	0.00059913	CAMKV
4568	A_37_P065438	0.00059913	ZNF605
4569	A_33_P3214463	0.00059917	MESP2
4570	A_37_P052220	0.00059977	PUS10
4571	A_37_P120274	0.00060005	REN
4572	A_33_P3271755	0.00060104	SIRT5
4573	A_37_P180358	0.00060362	FAM96A
4574	A_37_P081775	0.00060362	CU687139
4575	A_32_P34138	0.00060399	FAM25A
4576	A_37_P211174	0.00060669	SLCO2B1
4577	A_37_P244502	0.00060705	SYN2
4578	A_37_P034660	0.00060837	IL34
4579	A_37_P241307	0.00060871	MYL3
4580	A_37_P053391	0.00060912	RSAD2
4581	A_37_P112058	0.0006098	LAD1
4582	A_37_P180165	0.00061127	LTK
4583	A_37_P127303	0.00061171	ENST00000490780
4584	A_37_P150396	0.00061171	PPFIA3
4585	A_37_P149054	0.00061263	C19orf26
4586	A_37_P017715	0.00061296	NISCH
4587	A_37_P275683	0.00061339	PNKD
4588	A_37_P386826	0.00061396	TNRC18
4589	A_37_P167846	0.0006154	FER1L6
4590	A_33_P3385656	0.00061579	C5orf40
4591	A_37_P312374	0.00061592	TRPV1
4592	A_37_P186502	0.00061696	ATRN
4593	A_37_P317696	0.00061719	KIAA1432
4594	A_37_P345209	0.00061739	MAP7D1
4595	A_37_P377504	0.00061768	ZNF554
4596	A_37_P392845	0.00061826	OC90
4597	A_37_P129270	0.00061943	SELP
4598	A_37_P347755	0.00061986	PEX14
4599	A_37_P202100	0.00062117	FXYD2
4600	A_37_P278405	0.00062235	SPTBN1
4601	A_37_P320935	0.00062332	TRPM3
4602	A_37_P213806	0.00062335	ABCG1
4603	A_37_P414034	0.00062365	NAPB
4604	A_37_P098127	0.00062537	TEK
4605	A_37_P175827	0.00062709	GRAMD2
4606	A_37_P210955	0.00062732	SLC22A9
4607	A_23_P167841	0.00062779	KCNQ5
4608	A_37_P032931	0.00062795	GDPD3
4609	A_37_P078321	0.00062856	BAIAP2

1220	A_37_P305549	1.03E-005	GOSR2
1221	A_37_P081227	1.03E-005	CUEDC1
1222	A_37_P113681	1.04E-005	TESK2
1223	A_37_P178764	1.04E-005	PAQR5
1224	A_37_P161419	1.04E-005	TFR2
1225	A_37_P255368	1.04E-005	SEPX1
1226	A_37_P297766	1.04E-005	MSL3
1227	A_32_P3534	1.04E-005	PGPEP1L
1228	A_37_P208703	1.05E-005	NLRP6
1229	A_33_P3234490	1.05E-005	BOLA2B
1230	A_33_P3213493	1.05E-005	VAMP2
1231	A_37_P081359	1.05E-005	FLJ35220
1232	A_37_P068844	1.05E-005	VSIG10
1233	A_37_P179339	1.05E-005	RCCD1
1234	A_37_P374698	1.05E-005	CABP5
1235	A_37_P077990	1.05E-005	UNC13D
1236	A_37_P144309	1.06E-005	MEX3D
1237	A_33_P3315906	1.06E-005	PTP4A3
1238	A_37_P127049	1.06E-005	SCCPDH
1239	A_33_P3260026	1.06E-005	AIF1L
1240	A_23_P161563	1.06E-005	RAB38
1241	A_37_P140912	1.06E-005	SYNJ2
1242	A_33_P3261625	1.06E-005	LSM2
1243	A_37_P031009	1.06E-005	ABCA3
1244	A_37_P181640	1.07E-005	RNF215
1245	A_37_P113782	1.07E-005	CYP4X1
1246	A_37_P031565	1.07E-005	CLN3
1247	A_23_P216766	1.07E-005	ISCA1
1248	A_37_P084278	1.07E-005	PLSCR3
1249	A_37_P014312	1.07E-005	HEMK1
1250	A_37_P071665	1.08E-005	TRMT2B
1251	A_37_P152613	1.08E-005	RTN2
1252	A_37_P073997	1.08E-005	PNCK
1253	A_37_P082329	1.08E-005	MRPL10
1254	A_37_P109778	1.08E-005	ANKRD35
1255	A_37_P211401	1.08E-005	CCDC153
1256	A_37_P332292	1.08E-005	CDH24
1257	A_37_P107138	1.09E-005	PTGDR
1258	A_37_P126996	1.09E-005	SAMD11
1259	A_37_P126650	1.10E-005	PBXIP1
1260	A_37_P215289	1.11E-005	C21orf2
1261	A_37_P278090	1.11E-005	CCDC115
1262	A_37_P074038	1.11E-005	PNPLA4
1263	A_37_P185886	1.11E-005	TRMU
1264	A_37_P055296	1.11E-005	NDUFA10
1265	A_37_P048862	1.11E-005	DNPEP
1266	A_23_P109269	1.11E-005	LAMA5
1267	A_23_P68899	1.12E-005	TXN2
1268	A_23_P254741	1.12E-005	SOD3
1269	A_37_P241942	1.12E-005	PDIA5
1270	A_37_P015556	1.12E-005	FAM86C
1271	A_37_P210120	1.12E-005	LRDD
1272	A_23_P51853	1.12E-005	ZDHHC18
1273	A_37_P098006	1.12E-005	TBC1D13
1274	A_37_P027587	1.12E-005	SPINK2
1275	A_37_P391308	1.12E-005	TSNARE1
1276	A_37_P353027	1.12E-005	TMEM69
1277	A_37_P171996	1.12E-005	LYPLA1
1278	A_37_P088775	1.13E-005	RILP
1279	A_33_P3300212	1.13E-005	BLCAP
1280	A_37_P190570	1.13E-005	SIRPB1

4610	A_37_P215903	0.00062862	URB1
4611	A_37_P439666	0.00062968	TRAPPC10
4612	A_37_P320837	0.00062997	C9orf95
4613	A_37_P138879	0.00063018	PI16
4614	A_37_P132748	0.00063041	COL21A1
4615	A_37_P332548	0.00063051	ZC3H14
4616	A_37_P313489	0.00063061	FLCN
4617	A_23_P50674	0.00063108	IZUMO4
4618	A_33_P3338341	0.00063127	PRODH
4619	A_37_P202575	0.00063209	KRTAP5-4
4620	A_37_P175152	0.00063242	GOLGA8F
4621	A_37_P163389	0.0006336	SLC4A2
4622	A_37_P198097	0.00063422	NFKB2
4623	A_37_P150069	0.00063696	FAM71E2
4624	A_37_P071132	0.00063724	PIR
4625	A_37_P161548	0.00063747	ELMO1
4626	A_23_P167017	0.00063816	POPDC2
4627	A_37_P202355	0.00063892	ME3
4628	A_37_P421036	0.00063915	C10orf79
4629	A_37_P155741	0.00063919	C7orf63
4630	A_33_P3350493	0.0006427	C6orf126
4631	A_37_P335242	0.0006434	C1QB
4632	A_37_P286121	0.0006438	KLRG1
4633	A_37_P067209	0.00064502	PPM1H
4634	A_37_P087686	0.00064657	SLC13A2
4635	A_37_P056910	0.00064691	Mar-04
4636	A_37_P440210	0.00064785	B3GALTL
4637	A_37_P021639	0.00064951	UPK1B
4638	A_23_P77415	0.00064979	OSGIN1
4639	A_23_P36484	0.00064997	RDH16
4640	A_37_P363615	0.00065018	CLVS2
4641	A_37_P216114	0.00065029	USP25
4642	A_37_P041172	0.00065082	ALPP
4643	A_23_P6546	0.00065128	TUBGCP6
4644	A_37_P272160	0.00065154	ARHGEF33
4645	A_37_P041860	0.0006521	MDH1B
4646	A_37_P309109	0.00065258	C17orf68
4647	A_37_P263286	0.00065347	TPSD1
4648	A_37_P046093	0.00065353	OST4
4649	A_37_P058808	0.00065411	COL2A1
4650	A_37_P148245	0.00065451	DPP9
4651	A_33_P3259861	0.00065453	REREP3
4652	A_37_P230990	0.0006547	IRX2
4653	A_37_P152022	0.0006547	RTBDN
4654	A_37_P058940	0.00065493	GALNT9
4655	A_37_P243985	0.00065907	SLC6A11
4656	A_37_P190873	0.00065919	SRC
4657	A_37_P049537	0.00066043	HS6ST1
4658	A_37_P189843	0.00066059	PCK1
4659	A_37_P089493	0.00066177	USP43
4660	A_37_P184105	0.00066289	HPS4
4661	A_33_P3304282	0.00066326	RPS15A
4662	A_37_P227803	0.00066365	LEAP2
4663	A_33_P3424222	0.00066391	HLA-DQB1
4664	A_37_P202519	0.00066408	TMPRSS5
4665	A_37_P063233	0.00066461	TMEM233
4666	A_37_P189797	0.00066502	EMILIN3
4667	A_37_P081170	0.0006656	TEX14
4668	A_37_P014440	0.0006663	HTR3D
4669	A_37_P422642	0.00066759	PRAP1
4670	A_37_P207366	0.00066778	C11orf16

1281	A_37_P143903	1.13E-005	CD79A
1282	A_37_P104531	1.13E-005	GLRX5
1283	A_33_P3371718	1.13E-005	01/08/12
1284	A_37_P286668	1.13E-005	HDAC7
1285	A_37_P081750	1.13E-005	ARL16
1286	A_37_P377562	1.13E-005	ZNF576
1287	A_37_P053991	1.14E-005	FAM178B
1288	A_23_P39647	1.14E-005	SLC4A3
1289	A_37_P160891	1.15E-005	MDFIC
1290	A_37_P230535	1.15E-005	PRELID1
1291	A_37_P198911	1.15E-005	PPP2R2D
1292	A_37_P353428	1.15E-005	TSEN15
1293	A_37_P046551	1.15E-005	MPV17
1294	A_37_P186769	1.16E-005	PPDPF
1295	A_37_P057432	1.16E-005	SMAGP
1296	A_37_P174203	1.17E-005	CD276
1297	A_37_P139426	1.17E-005	PRR3
1298	A_37_P051275	1.17E-005	SPRED2
1299	A_37_P102021	1.17E-005	TGIF1
1300	A_37_P186094	1.18E-005	MAPK12
1301	A_37_P153773	1.18E-005	ZNF544
1302	A_37_P078767	1.18E-005	C17orf39
1303	A_37_P387177	1.19E-005	SND1
1304	A_37_P039302	1.19E-005	TELO2
1305	A_37_P269990	1.19E-005	ASTL
1306	A_37_P086424	1.19E-005	PLD2
1307	A_37_P155640	1.19E-005	CCZ1
1308	A_37_P193609	1.19E-005	CREM
1309	A_23_P104073	1.19E-005	S100A3
1310	A_33_P3416574	1.20E-005	AIG1
1311	A_37_P118107	1.20E-005	DENND4B
1312	A_37_P034773	1.20E-005	ITFG3
1313	A_37_P149035	1.20E-005	C19orf12
1314	A_37_P153602	1.21E-005	ZNF333
1315	A_37_P061262	1.21E-005	SCARB1
1316	A_37_P117880	1.21E-005	UBE2J2
1317	A_37_P125760	1.21E-005	RAB4A
1318	A_37_P082211	1.21E-005	DCXR
1319	A_37_P261235	1.21E-005	RPS2
1320	A_37_P130972	1.21E-005	ZNF362
1321	A_37_P075192	1.22E-005	RBM3
1322	A_37_P145131	1.22E-005	EGLN2
1323	A_37_P233065	1.22E-005	MAPK9
1324	A_37_P004250	1.22E-005	DIMT1L
1325	A_37_P273440	1.22E-005	LYPD6
1326	A_37_P398132	1.22E-005	PIF1
1327	A_37_P116672	1.22E-005	GSTM5
1328	A_24_P392480	1.22E-005	MYPOP
1329	A_37_P124616	1.22E-005	POLR3GL
1330	A_37_P157242	1.22E-005	ELN
1331	A_37_P433564	1.22E-005	PTPRJ
1332	A_37_P054119	1.23E-005	SMPD4
1333	A_37_P179203	1.23E-005	PARP16
1334	A_37_P155439	1.23E-005	BZW2
1335	A_37_P116521	1.23E-005	GPR137B
1336	A_37_P366371	1.23E-005	ANKRD24
1337	A_37_P249861	1.24E-005	KLHL2
1338	A_37_P323129	1.24E-005	C18orf8
1339	A_33_P3360773	1.25E-005	TSSC4
1340	A_37_P096968	1.25E-005	STOML2
1341	A_37_P322204	1.25E-005	UBAP1

4671	A_37_P120163	0.00066978	SYNC
4672	A_23_P164011	0.00067079	SOX15
4673	A_37_P313373	0.00067149	WRAP53
4674	A_37_P178098	0.00067247	MAPKBP1
4675	A_33_P3266928	0.00067284	LMTK3
4676	A_37_P092131	0.00067481	DBH
4677	A_33_P3341601	0.00067506	WDR86
4678	A_23_P142447	0.00067539	MYO1F
4679	A_37_P034796	0.00067552	ITGAD
4680	A_37_P129827	0.00067579	TTLL10
4681	A_37_P170624	0.00067777	RPS20
4682	A_37_P163452	0.00068074	SMO
4683	A_33_P3291454	0.00068105	C10orf96
4684	A_37_P081631	0.00068239	TNFRSF13B
4685	A_37_P087782	0.00068253	SECTM1
4686	A_37_P052677	0.00068305	RAPGEF4
4687	A_33_P3379391	0.00068472	H2AFJ
4688	A_23_P146576	0.00068478	GBGT1
4689	A_37_P141733	0.00068533	RMND1
4690	A_24_P406132	0.0006867	MAPK13
4691	A_37_P081874	0.00068759	SCARF1
4692	A_37_P130637	0.0006879	XKR8
4693	A_37_P158622	0.00068924	ATG9B
4694	A_37_P188614	0.00068925	ZMYND8
4695	A_37_P289235	0.00068945	PIWIL1
4696	A_37_P283929	0.00068963	ANKS1B
4697	A_33_P3329984	0.00068964	GGN
4698	A_23_P157513	0.00069023	MOS
4699	A_37_P260087	0.00069094	MYH11
4700	A_37_P413280	0.0006936	C20orf165
4701	A_37_P099655	0.00069538	CIDEA
4702	A_37_P205556	0.00069592	ATG2A
4703	A_37_P366474	0.00069761	KLK8
4704	A_37_P139709	0.00069838	RBM24
4705	A_37_P103520	0.00069964	PRKD1
4706	A_37_P316494	0.00070024	FAM73B
4707	A_37_P158112	0.00070123	SVOPL
4708	A_37_P143545	0.00070135	MED16
4709	A_37_P066581	0.00070209	ITGA7
4710	A_37_P367185	0.0007037	RAVER1
4711	A_37_P082115	0.00070396	HEXDC
4712	A_37_P008828	0.00070527	TSLP
4713	A_37_P086979	0.00070577	EXOC7
4714	A_37_P155416	0.00070623	COBL
4715	A_37_P017926	0.00070758	OGG1
4716	A_37_P153833	0.00070941	ZNF57
4717	A_24_P270460	0.00071004	IFI27
4718	A_37_P170562	0.00071034	ENPP2
4719	A_37_P022092	0.00071176	MASP1
4720	A_37_P388976	0.00071272	TRPV6
4721	A_23_P143047	0.00071299	ATP6V1E2
4722	A_37_P186700	0.00071326	PRIC285
4723	A_37_P295987	0.0007134	IL9R
4724	A_37_P082652	0.00071348	RPTOR
4725	A_37_P042986	0.00071387	CDCA7
4726	A_37_P059132	0.00071617	CD4
4727	A_37_P191994	0.00071758	ALOX5
4728	A_23_P167168	0.00071881	IGJ
4729	A_37_P135869	0.00071918	IGF2R
4730	A_37_P212131	0.00071993	TPCN2
4731	A_37_P038588	0.00072186	CBFA2T3

1342	A_37_P250657	1.25E-005	MYL5
1343	A_37_P124213	1.25E-005	PIGV
1344	A_33_P3297244	1.26E-005	GAS2L2
1345	A_37_P288145	1.26E-005	MVK
1346	A_37_P214654	1.26E-005	IFNAR2
1347	A_37_P288806	1.26E-005	OGFOD2
1348	A_23_P1641	1.26E-005	RCE1
1349	A_37_P102644	1.26E-005	ADSSL1
1350	A_37_P146833	1.26E-005	RPL18
1351	A_37_P087962	1.26E-005	SNX11
1352	A_23_P369899	1.26E-005	TMEM158
1353	A_37_P098270	1.27E-005	TMEM38B
1354	A_24_P291401	1.27E-005	TMEM150A
1355	A_37_P092978	1.27E-005	FPGS
1356	A_37_P079016	1.27E-005	GALK1
1357	A_37_P082616	1.27E-005	KIAA0195
1358	A_37_P376268	1.27E-005	TMEM147
1359	A_37_P040445	1.28E-005	ZNF688
1360	A_37_P087865	1.28E-005	RAB40B
1361	A_37_P120530	1.28E-005	MUTYH
1362	A_37_P258661	1.28E-005	KCTD5
1363	A_37_P140731	1.28E-005	PDCD2
1364	A_37_P086957	1.28E-005	TMC6
1365	A_37_P001306	1.28E-005	CCNG1
1366	A_37_P190824	1.29E-005	SPAG4
1367	A_37_P188796	1.29E-005	BCL2L1
1368	A_37_P091554	1.30E-005	TTLL11
1369	A_37_P086343	1.30E-005	PFAS
1370	A_37_P034750	1.30E-005	PSMB10
1371	A_37_P232182	1.30E-005	TMCO6
1372	A_37_P086088	1.30E-005	NT5M
1373	A_37_P104600	1.30E-005	GPR137C
1374	A_37_P319956	1.31E-005	PGM5
1375	A_37_P134457	1.31E-005	UBE2CBP
1376	A_23_P422071	1.31E-005	B3GALT4
1377	A_37_P061113	1.31E-005	GABARAPL1
1378	A_37_P026229	1.32E-005	DGKQ
1379	A_23_P501745	1.32E-005	PEX10
1380	A_37_P146526	1.33E-005	IGFL2
1381	A_37_P214187	1.33E-005	SUMO3
1382	A_37_P046997	1.34E-005	ITM2C
1383	A_37_P211425	1.34E-005	ST14
1384	A_37_P396707	1.34E-005	ZNF7
1385	A_37_P057531	1.34E-005	PHB2
1386	A_37_P421316	1.34E-005	LZTS2
1387	A_23_P61960	1.35E-005	ATP6V0E2
1388	A_37_P044485	1.35E-005	LIMS2
1389	A_37_P144547	1.35E-005	GIPC1
1390	A_37_P091071	1.36E-005	C9orf7
1391	A_37_P259222	1.36E-005	TXNDC11
1392	A_33_P3332396	1.37E-005	TMEM53
1393	A_37_P353789	1.37E-005	FCRL5
1394	A_37_P052635	1.37E-005	RALB
1395	A_37_P428145	1.37E-005	CTSF
1396	A_37_P043504	1.37E-005	GGCX
1397	A_23_P7083	1.38E-005	C4orf42
1398	A_37_P218703	1.38E-005	EBPL
1399	A_23_P164148	1.38E-005	MLX
1400	A_37_P348708	1.38E-005	PPP2R5A
1401	A_37_P136709	1.38E-005	MTCH1
1402	A_37_P152759	1.38E-005	FBXL12

4732	A_37_P010737	0.00072194	BOC
4733	A_37_P156387	0.00072194	CNTNAP2
4734	A_37_P178460	0.00072285	SPG11
4735	A_37_P090929	0.000723	C9orf103
4736	A_37_P182354	0.00072473	DEPDC5
4737	A_37_P387533	0.00072503	STAG3L4
4738	A_37_P107522	0.00072506	RAB15
4739	A_37_P104053	0.00072782	DYNC1H1
4740	A_37_P021382	0.00072983	TRPC1
4741	A_37_P194405	0.00073122	TRDMT1
4742	A_33_P3311245	0.00073122	KRTAP19-7
4743	A_37_P218335	0.00073167	DOCK9
4744	A_23_P118065	0.00073295	HSD17B2
4745	A_37_P319241	0.00073328	PTPRD
4746	A_37_P039406	0.00073396	TMC5
4747	A_37_P153417	0.00073426	ZNF175
4748	A_37_P055817	0.00073443	TRPM8
4749	A_37_P132825	0.00073524	C6orf136
4750	A_37_P006473	0.00073543	RPS23
4751	A_37_P097942	0.00073545	ENST00000376458
4752	A_37_P209481	0.00073547	PIWIL4
4753	A_37_P171608	0.00073633	SFTPC
4754	A_23_P319572	0.0007369	NR1I3
4755	A_37_P155697	0.00073787	C7orf53
4756	A_37_P071868	0.00073795	RPGR
4757	A_37_P195624	0.00073803	SFMBT2
4758	A_37_P366090	0.00073863	NKPD1
4759	A_37_P416607	0.00073876	ADAM12
4760	A_37_P380387	0.00073878	GIGYF1
4761	A_37_P166287	0.00073963	C8orf46
4762	A_37_P256795	0.00074151	CRISPLD2
4763	A_37_P160394	0.00074154	HIBADH
4764	A_37_P179069	0.0007422	PLEKHO2
4765	A_37_P398208	0.00074249	LMAN1L
4766	A_37_P044174	0.00074253	ANKMY1
4767	A_37_P212272	0.00074314	TSPAN32
4768	A_37_P262563	0.0007443	SOLH
4769	A_37_P025754	0.00074434	DDX60L
4770	A_37_P142846	0.0007446	ARHGEF1
4771	A_37_P110176	0.00074513	KNCN
4772	A_23_P344594	0.00074529	TMEM174
4773	A_37_P318498	0.00074647	C9orf173
4774	A_37_P013522	0.00074744	PRRT3
4775	A_37_P087655	0.00074837	SHBG
4776	A_37_P184092	0.00074901	MAPK8IP2
4777	A_37_P207247	0.00074989	TP53I11
4778	A_37_P050980	0.00075072	SDC1
4779	A_33_P3267270	0.0007535	C1orf83
4780	A_37_P068039	0.00075416	TMEM19
4781	A_37_P009602	0.00075459	ZNF608
4782	A_37_P260923	0.00075652	NUDT16L1
4783	Mouse-GAPDH_3	0.00075652	Mouse-GAPDH_3
4784	A_37_P157694	0.00075732	MLXIPL
4785	A_37_P375769	0.0007585	TMEM86B
4786	A_37_P264978	0.00075857	ANKAR
4787	A_37_P103253	0.00075957	C14orf80
4788	A_37_P131375	0.00075975	PGC
4789	A_37_P310493	0.00076072	RAB11FIP4
4790	A_37_P183279	0.00076072	GSC2
4791	A_37_P153637	0.00076186	ZNF431
4792	A_37_P079954	0.00076197	CRHR1

1403	A_37_P402638	1.38E-005	PIGB
1404	A_37_P194778	1.38E-005	FBXL15
1405	A_37_P104776	1.39E-005	IFI27L1
1406	A_37_P210220	1.39E-005	TRIM29
1407	A_37_P148897	1.39E-005	MRPL54
1408	A_37_P059502	1.39E-005	LASS5
1409	A_37_P042894	1.40E-005	CCDC74A
1410	A_23_P253029	1.40E-005	BOK
1411	A_23_P310483	1.40E-005	C8orf58
1412	A_37_P110961	1.40E-005	AP3S1
1413	A_37_P092010	1.40E-005	CRAT
1414	A_37_P048275	1.40E-005	ODC1
1415	A_33_P3412095	1.40E-005	PEX7
1416	A_24_P207479	1.40E-005	DEDD2
1417	A_37_P151811	1.41E-005	SNAPC2
1418	A_37_P004334	1.41E-005	HCN1
1419	A_37_P366867	1.41E-005	WIZ
1420	A_33_P3214466	1.41E-005	MESP1
1421	A_37_P141963	1.41E-005	KIAA1949
1422	A_37_P175254	1.41E-005	CTSH
1423	A_24_P200942	1.42E-005	TSC22D4
1424	A_37_P135990	1.43E-005	UBE2J1
1425	A_37_P032873	1.43E-005	AKTIP
1426	A_37_P375109	1.43E-005	SCN1B
1427	A_37_P156748	1.44E-005	CUX1
1428	A_33_P3270445	1.44E-005	BAI2
1429	A_37_P162621	1.44E-005	REPIN1
1430	A_37_P099412	1.44E-005	C18orf55
1431	A_37_P242393	1.45E-005	PODXL2
1432	A_37_P155906	1.45E-005	CASD1
1433	A_37_P315560	1.45E-005	ALDOB
1434	A_37_P020143	1.46E-005	SLC25A26
1435	A_37_P033648	1.46E-005	ELMO3
1436	A_37_P092902	1.46E-005	FGD3
1437	A_37_P066336	1.46E-005	RNF10
1438	A_37_P353268	1.47E-005	KIAA0495
1439	A_37_P067175	1.47E-005	LTA4H
1440	A_37_P109765	1.47E-005	YRDC
1441	A_37_P062997	1.47E-005	UBE2N
1442	A_37_P223913	1.48E-005	AFAP1L1
1443	A_37_P433533	1.48E-005	PTDSS2
1444	A_37_P400504	1.48E-005	HDGFRP3
1445	A_37_P151421	1.48E-005	FAM108A1
1446	A_37_P149767	1.48E-005	PTPRS
1447	A_23_P387045	1.48E-005	CCDC107
1448	A_37_P258616	1.48E-005	JPH3
1449	A_37_P306518	1.48E-005	PSMC3IP
1450	A_37_P105897	1.48E-005	BBS5
1451	A_37_P132727	1.48E-005	C4A
1452	A_37_P154676	1.49E-005	AGAP3
1453	A_37_P255708	1.49E-005	FAM195A
1454	A_37_P150756	1.49E-005	QTRT1
1455	A_23_P39386	1.49E-005	HCST
1456	A_37_P172504	1.49E-005	UBXN8
1457	A_23_P31921	1.50E-005	ASS1
1458	A_37_P287888	1.50E-005	HRK
1459	A_37_P059610	1.50E-005	CREBL2
1460	A_37_P106165	1.50E-005	HAUS4
1461	A_32_P77098	1.51E-005	TMEM200B
1462	A_37_P442358	1.51E-005	MCF2L
1463	A_23_P107693	1.51E-005	ZNF586

4793	A_23_P257609	0.0007625	RPL29
4794	A_37_P146618	0.00076282	ATP13A1
4795	A_37_P046803	0.00076337	INPP5D
4796	A_37_P146315	0.00076429	HKR1
4797	A_37_P199765	0.0007643	FAM24B
4798	A_37_P293379	0.00076454	BGN
4799	A_37_P205674	0.00076585	IGHMBP2
4800	A_37_P058736	0.00076629	KRT81
4801	A_37_P022796	0.00076854	ABLIM2
4802	A_37_P016487	0.00076934	HHATL
4803	A_24_P336137	0.0007696	C22orf23
4804	A_37_P390024	0.00076973	C8orf74
4805	A_37_P179109	0.00077062	SLC30A4
4806	A_37_P248526	0.00077203	CXCL3
4807	A_37_P017860	0.00077219	NR1I2
4808	A_37_P126441	0.0007723	KAZ
4809	A_23_P26024	0.00077269	C15orf48
4810	A_23_P321511	0.00077312	Mar-03
4811	A_37_P145839	0.00077409	GIPR
4812	A_37_P141001	0.00077453	LYRM4
4813	A_37_P305208	0.00077523	ARHGAP27
4814	A_33_P3385488	0.00077538	RPS18
4815	A_37_P156681	0.00077655	GIMAP6
4816	A_37_P041472	0.00077659	USP40
4817	A_37_P068321	0.0007793	TSPAN11
4818	A_37_P094341	0.00078011	BARX1
4819	A_37_P161425	0.00078039	NPTX2
4820	A_37_P277061	0.00078092	LRP2
4821	A_23_P251795	0.00078258	GPC2
4822	A_37_P146575	0.00078293	IL28A
4823	A_37_P123776	0.00078724	PAX7
4824	A_37_P302064	0.00078752	KRT13
4825	A_37_P359918	0.0007896	KIAA1244
4826	A_37_P233731	0.00079115	ABCC5
4827	A_37_P336991	0.00079129	GFI1
4828	A_37_P121917	0.00079162	MPL
4829	A_37_P004644	0.0007928	DNAH5
4830	A_37_P047134	0.0007931	KHK
4831	A_37_P003188	0.00079318	SLC23A1
4832	A_37_P327379	0.00079637	NPC2
4833	A_37_P307012	0.0007967	HCRT
4834	A_37_P145395	0.00079707	FAM129C
4835	A_23_P411188	0.00079886	CHRNA10
4836	A_37_P147116	0.00079939	LASS4
4837	A_37_P067644	0.00080073	TAPBPL
4838	A_37_P146485	0.00080124	ICAM4
4839	A_37_P264847	0.00080516	ALMS1
4840	A_37_P422007	0.0008055	OPN4
4841	A_37_P209679	0.00080748	PPFIBP2
4842	A_37_P203374	0.00080843	AMBRA1
4843	A_37_P012551	0.00080858	DLEC1
4844	A_23_P207194	0.00080932	GH1
4845	A_37_P263463	0.00081112	TSNAXIP1
4846	A_23_P157007	0.00081188	TMEM176B
4847	A_37_P057658	0.00081454	ANO4
4848	A_32_P216734	0.00081652	SPDYE3
4849	A_37_P406148	0.00081753	LIF
4850	A_37_P197265	0.00081788	C10orf54
4851	A_37_P032691	0.00081788	CHP2
4852	A_37_P210234	0.00081812	TRIM3
4853	A_37_P205336	0.00081947	RCOR2

1464	A_37_P308590	1.51E-005	MED9
1465	A_37_P120127	1.51E-005	HES5
1466	A_37_P122895	1.51E-005	NFYC
1467	A_37_P183830	1.51E-005	DNAL4
1468	A_37_P203658	1.52E-005	DGAT2
1469	A_37_P103779	1.52E-005	XRCC3
1470	A_37_P435476	1.52E-005	TBRG1
1471	A_37_P374624	1.52E-005	REEP6
1472	A_37_P017786	1.52E-005	EIF4E3
1473	A_37_P143851	1.52E-005	CCNE1
1474	A_37_P031667	1.52E-005	ACD
1475	A_37_P192879	1.52E-005	UROS
1476	A_37_P031498	1.52E-005	SULT1A1
1477	A_37_P150966	1.52E-005	RRAS
1478	A_37_P166948	1.53E-005	DDHD2
1479	A_37_P127695	1.53E-005	SLC22A15
1480	A_37_P057026	1.53E-005	PFN4
1481	A_37_P212871	1.53E-005	TMEM134
1482	A_37_P372627	1.53E-005	MUM1
1483	A_37_P182786	1.54E-005	FAM118A
1484	A_37_P190755	1.54E-005	SMOX
1485	A_37_P110731	1.55E-005	ACP6
1486	A_23_P51376	1.55E-005	NKAIN1
1487	A_37_P356879	1.56E-005	CCDC28A
1488	A_37_P282950	1.56E-005	CHPT1
1489	A_37_P061323	1.56E-005	GNB3
1490	A_37_P394600	1.57E-005	PPP3CC
1491	A_37_P085409	1.57E-005	C17orf70
1492	A_37_P431203	1.57E-005	C11orf51
1493	A_37_P033859	1.57E-005	FBXL19
1494	A_23_P218646	1.57E-005	TNFRSF6B
1495	A_37_P211131	1.58E-005	SLC36A4
1496	A_37_P140601	1.58E-005	SMOC2
1497	A_37_P124122	1.58E-005	PGM1
1498	A_37_P050504	1.58E-005	C1D
1499	A_37_P035764	1.58E-005	MTSS1L
1500	A_37_P226922	1.58E-005	HARS2
1501	A_37_P031609	1.58E-005	ATP2A1
1502	A_37_P244552	1.58E-005	TATDN2
1503	A_37_P124984	1.59E-005	PPP1R8
1504	A_37_P142717	1.60E-005	AP2A1
1505	A_37_P212678	1.60E-005	VPS26B
1506	A_37_P206330	1.60E-005	BACE1
1507	A_37_P389943	1.60E-005	C8orf38
1508	A_37_P087544	1.60E-005	Sep-09
1509	A_37_P033462	1.60E-005	ANKS3
1510	A_37_P390253	1.60E-005	NIPAL2
1511	A_37_P124040	1.61E-005	PEX11B
1512	A_33_P3311979	1.61E-005	ZFPM1
1513	A_37_P149793	1.61E-005	PAK4
1514	A_37_P129144	1.61E-005	TAF12
1515	A_37_P376338	1.61E-005	TMEM91
1516	A_37_P287646	1.61E-005	LRRC23
1517	A_37_P101057	1.61E-005	C18orf10
1518	A_37_P107865	1.61E-005	SPATA7
1519	A_37_P100218	1.61E-005	HMSD
1520	A_24_P146892	1.61E-005	ORAI1
1521	A_33_P3256680	1.61E-005	MFHAS1
1522	A_37_P053809	1.62E-005	PKDCC
1523	A_37_P113505	1.62E-005	CPSF3L
1524	A_37_P178547	1.62E-005	UBL7

4854	A_37_P212071	0.0008201	CD3D
4855	A_37_P049659	0.00082174	LRRFIP1
4856	A_37_P242627	0.00082194	PTPRG
4857	A_37_P066173	0.00082311	NAP1L1
4858	A_37_P024737	0.00082322	PRSS12
4859	A_37_P220140	0.00082358	WDFY2
4860	A_37_P322216	0.00082435	FCN1
4861	A_23_P135381	0.00082507	SP5
4862	A_37_P203049	0.00082553	ST5
4863	A_37_P256119	0.00082579	CCL22
4864	A_33_P3266744	0.00082689	SYTL1
4865	A_37_P153524	0.00082695	ZNF264
4866	A_23_P501933	0.00082924	CACNG6
4867	A_37_P256236	0.00082931	CDH3
4868	A_37_P175771	0.00082954	NTRK3
4869	A_37_P380820	0.00083419	DYNC1I1
4870	A_37_P053642	0.00083419	ITGB1BP1
4871	A_37_P185025	0.00083643	SEC14L3
4872	A_37_P241215	0.00083644	NAALADL2
4873	A_37_P182959	0.00083791	TOP3B
4874	A_37_P087820	0.00083847	SLC5A10
4875	A_37_P210189	0.00083999	CABP2
4876	A_37_P376161	0.00084012	TJP3
4877	A_37_P276458	0.00084177	RBM44
4878	A_37_P035567	0.00084317	DNAH3
4879	A_37_P418000	0.00084331	CTBP2
4880	A_37_P115691	0.00084468	FHAD1
4881	A_37_P100112	0.00084524	FHOD3
4882	A_23_P214079	0.00084619	SPINK1
4883	A_23_P111194	0.00084634	SPDEF
4884	A_37_P426496	0.00084701	CCKBR
4885	A_37_P056060	0.00084707	SEMA4C
4886	A_33_P3390778	0.00084789	TRIM46
4887	A_37_P066101	0.00084811	RAD9B
4888	A_37_P440595	0.00084898	C13orf38-SOHLH2
4889	A_37_P098984	0.00085164	KIF12
4890	A_37_P056189	0.0008521	UPP2
4891	A_37_P235015	0.00085376	KY
4892	A_37_P229329	0.00085719	N4BP3
4893	A_37_P247224	0.00086138	COL25A1
4894	A_37_P032382	0.00086142	CCDC135
4895	A_37_P112702	0.00086256	AIM1L
4896	A_37_P156842	0.00086442	GLI3
4897	A_23_P101054	0.00086605	KRT34
4898	A_23_P56703	0.00087162	C2orf89
4899	A_37_P110771	0.00087162	BMP8A
4900	A_37_P375631	0.00087407	SPIB
4901	A_37_P211234	0.00087407	SORL1
4902	A_37_P079313	0.0008762	GAS7
4903	A_37_P285120	0.0008762	GPR162
4904	A_37_P228726	0.00087621	HK3
4905	A_37_P335990	0.00087885	CD1D
4906	A_37_P366309	0.00087931	KLK10
4907	A_37_P276087	0.00088114	LOXL3
4908	A_37_P140226	0.00088195	RGS17
4909	A_37_P213468	0.00088217	RPS4Y1
4910	A_37_P167778	0.00088413	FBXO25
4911	A_37_P143631	0.00088419	CARM1
4912	A_37_P299344	0.00088438	SHOX
4913	A_37_P165514	0.00088493	SLA
4914	A_37_P282507	0.00088559	ADCY6

1525	A_37_P114034	1.62E-005	LGTN
1526	A_37_P143309	1.63E-005	C19orf20
1527	A_37_P187577	1.63E-005	TMX4
1528	A_37_P338835	1.63E-005	MKNK1
1529	A_37_P329649	1.63E-005	C14orf143
1530	A_37_P153968	1.63E-005	ZNF419
1531	A_37_P174919	1.63E-005	DUT
1532	A_37_P084957	1.63E-005	MGAT5B
1533	A_37_P332795	1.63E-005	EPHX4
1534	A_37_P176225	1.63E-005	ITPKA
1535	A_37_P136678	1.64E-005	ABHD16A
1536	A_37_P036340	1.64E-005	TUBB3
1537	A_37_P109626	1.64E-005	ALPL
1538	A_37_P001024	1.65E-005	C5orf44
1539	A_37_P171335	1.65E-005	RGS20
1540	A_37_P087285	1.65E-005	CACNB1
1541	A_37_P267604	1.65E-005	ANAPC1
1542	A_37_P300807	1.65E-005	ZNF185
1543	A_37_P037360	1.66E-005	PARD6A
1544	A_37_P067971	1.66E-005	TMEM117
1545	A_37_P372496	1.66E-005	MLL4
1546	A_24_P91472	1.66E-005	ALKBH7
1547	A_37_P159045	1.66E-005	DGKI
1548	A_37_P388884	1.66E-005	STYXL1
1549	A_37_P183243	1.67E-005	ASCC2
1550	A_37_P268213	1.67E-005	DUSP28
1551	A_37_P376732	1.67E-005	FDX1L
1552	A_23_P37676	1.67E-005	GPR176
1553	A_37_P014913	1.67E-005	JAGN1
1554	A_33_P3376341	1.68E-005	LRRC24
1555	A_37_P178961	1.68E-005	PLA2G4B
1556	A_37_P018650	1.68E-005	PLXNA1
1557	A_37_P186349	1.68E-005	SNTA1
1558	A_37_P310684	1.68E-005	STRA13
1559	A_37_P204903	1.68E-005	FXC1
1560	A_37_P048713	1.69E-005	SFXN5
1561	A_37_P251036	1.69E-005	MED28
1562	A_37_P043933	1.69E-005	MFSD9
1563	A_37_P212077	1.69E-005	TMX2
1564	A_37_P063021	1.70E-005	TRIAP1
1565	A_37_P163190	1.70E-005	SH2B2
1566	A_37_P122190	1.70E-005	HSPG2
1567	A_37_P260630	1.70E-005	ZDHHC1
1568	A_37_P299742	1.70E-005	STARD8
1569	A_37_P420523	1.70E-005	SLC25A28
1570	A_24_P337657	1.70E-005	SRF
1571	A_37_P144439	1.71E-005	CRTC1
1572	A_37_P170765	1.71E-005	POLB
1573	A_37_P275169	1.72E-005	C2orf7
1574	A_37_P314970	1.72E-005	NOTCH1
1575	A_37_P065211	1.73E-005	P2RX4
1576	A_37_P196148	1.73E-005	LHPP
1577	A_23_P76078	1.73E-005	IL23A
1578	A_33_P3256920	1.73E-005	WNT7B
1579	A_37_P164201	1.73E-005	KEL
1580	A_37_P258435	1.73E-005	IQCK
1581	A_37_P217889	1.73E-005	MTUS2
1582	A_37_P007129	1.73E-005	REEP2
1583	A_37_P181365	1.74E-005	ACO2
1584	A_37_P003922	1.74E-005	DEPDC1B
1585	A_37_P202437	1.74E-005	TOLLIP

4915	A_37_P188174	0.00088571	05/08/12
4916	A_37_P004195	0.00088683	ZDHHC11
4917	A_37_P243935	0.0008874	LSAMP
4918	A_37_P088933	0.00088833	CD300C
4919	A_37_P094206	0.00088865	LCN1
4920	A_37_P108729	0.00088894	DCAF4
4921	A_37_P081912	0.00089105	GRAPL
4922	A_37_P063943	0.00089157	LRRC43
4923	A_33_P3401990	0.00089187	VPREB3
4924	A_37_P302358	0.00089256	KRT33B
4925	A_37_P391069	0.00089505	GNRH1
4926	A_37_P050842	0.00089544	NOTO
4927	A_37_P016721	0.00089654	TMEM40
4928	A_37_P138210	0.00089918	EHMT2
4929	A_37_P198670	0.00090021	PLAU
4930	A_37_P017193	0.00090147	CELSR3
4931	A_37_P105582	0.00090147	SLC25A29
4932	A_37_P156230	0.00090148	GCK
4933	A_37_P281789	0.00090179	B4GALNT3
4934	A_33_P3250887	0.00090483	C22orf41
4935	A_37_P207980	0.00090614	MICAL2
4936	A_37_P405433	0.00090704	USP18
4937	A_37_P200460	0.00091043	PDZD7
4938	A_37_P161391	0.00091096	NOS3
4939	A_32_P160896	0.00091147	FTMT
4940	A_37_P064547	0.00091159	MYO1H
4941	A_37_P278152	0.00091216	CCDC142
4942	A_37_P369438	0.00091266	CYP2A6
4943	A_37_P145855	0.0009132	STAP2
4944	A_37_P423826	0.00091325	TAF3
4945	A_37_P210796	0.00091411	SIPA1
4946	A_37_P301432	0.00091539	ABCA9
4947	A_37_P415461	0.00091838	ACADSB
4948	A_37_P115634	0.0009186	FCRLA
4949	A_37_P143721	0.0009186	PHLDB3
4950	A_37_P089071	0.00091955	TNRC6C
4951	A_37_P202815	0.00091962	AGBL2
4952	A_37_P071784	0.00092092	GLRA2
4953	A_37_P403370	0.00092115	SCG5
4954	A_37_P152854	0.00092189	TRPM4
4955	A_37_P193511	0.00092261	CNNM1
4956	A_37_P082719	0.00092414	ZNF286A
4957	A_37_P039131	0.00092514	FAM92B
4958	A_37_P202431	0.00092673	GAB2
4959	A_37_P118836	0.00092744	LHX8
4960	A_37_P438525	0.00092763	KRTAP10-7
4961	A_37_P224713	0.00092777	C5orf35
4962	A_37_P014800	0.00092821	TWF2
4963	A_37_P143581	0.00092968	CACNG8
4964	A_37_P135571	0.00093079	HLA-DPB1
4965	A_37_P135155	0.00093331	GPR115
4966	A_37_P160783	0.00093893	LRWD1
4967	A_37_P011466	0.0009411	CCR2
4968	A_37_P100746	0.00094137	CELF4
4969	A_33_P3300142	0.00094244	ANKFY1
4970	A_37_P129660	0.00094315	MAP1LC3C
4971	A_37_P154317	0.00094515	ZSCAN18
4972	A_37_P205068	0.00094549	GLYATL1
4973	A_23_P114210	0.00094681	POU3F4
4974	A_37_P111063	0.00094714	C1orf112
4975	A_37_P014595	0.00094779	IL17RE

1586	A_37_P214607	1.75E-005	RCAN1
1587	A_37_P125195	1.75E-005	PRKCZ
1588	A_37_P106509	1.75E-005	IFI27L2
1589	A_37_P407898	1.75E-005	MEI1
1590	A_37_P341019	1.75E-005	HPCA
1591	A_37_P089041	1.75E-005	TNFSF13
1592	A_37_P175223	1.75E-005	FAH
1593	A_37_P358055	1.75E-005	LMBRD1
1594	A_37_P008147	1.75E-005	CCDC69
1595	A_37_P084943	1.76E-005	MFSD11
1596	A_37_P087579	1.76E-005	TMEM101
1597	A_37_P362775	1.76E-005	PARK2
1598	A_37_P314734	1.76E-005	C9orf3
1599	A_37_P379962	1.77E-005	CHN2
1600	A_37_P436510	1.77E-005	NFRKB
1601	A_37_P170371	1.77E-005	PABPC1
1602	A_37_P156580	1.77E-005	GGCT
1603	A_37_P007140	1.78E-005	RELL2
1604	A_37_P185633	1.78E-005	RAC2
1605	A_37_P081781	1.78E-005	A_37_P081781
1606	A_37_P002404	1.78E-005	UBTD2
1607	A_37_P155924	1.78E-005	CAV2
1608	A_33_P3362781	1.78E-005	KLHL36
1609	A_37_P055023	1.78E-005	PDE1A
1610	A_37_P257130	1.78E-005	DNASE1L2
1611	A_33_P3291636	1.79E-005	ZNF74
1612	A_37_P175210	1.79E-005	AP3S2
1613	A_37_P027238	1.79E-005	MAN2B2
1614	A_37_P113847	1.79E-005	DAP3
1615	A_37_P212200	1.80E-005	RIN1
1616	A_37_P165298	1.80E-005	ZNF655
1617	A_37_P083131	1.80E-005	NXN
1618	A_37_P126477	1.80E-005	ISG20L2
1619	A_37_P149549	1.80E-005	NUCB1
1620	A_37_P197272	1.82E-005	RNLS
1621	A_37_P043813	1.82E-005	CREG2
1622	A_37_P129469	1.82E-005	TOMM40L
1623	A_37_P286245	1.83E-005	B4GALNT1
1624	A_37_P174847	1.83E-005	GNB5
1625	A_37_P063228	1.83E-005	CCDC42B
1626	A_37_P165080	1.84E-005	ZDHHC4
1627	A_37_P315566	1.84E-005	ALG2
1628	A_37_P380196	1.84E-005	COX19
1629	A_37_P058308	1.84E-005	C12orf39
1630	A_37_P190485	1.84E-005	RGS19
1631	A_37_P032301	1.84E-005	CACNA1H
1632	A_37_P383245	1.85E-005	VIPR2
1633	A_37_P105968	1.85E-005	C14orf147
1634	A_37_P367953	1.85E-005	CLPP
1635	A_37_P142953	1.86E-005	ATF5
1636	A_37_P064889	1.86E-005	NR4A1
1637	A_37_P148678	1.86E-005	DYRK1B
1638	A_37_P069816	1.86E-005	TSC22D3
1639	A_33_P3248329	1.87E-005	ZFAND3
1640	A_23_P202773	1.88E-005	C11orf20
1641	A_37_P114697	1.89E-005	DUSP12
1642	A_37_P324411	1.89E-005	PQLC1
1643	A_37_P181599	1.89E-005	ATF4
1644	A_23_P404667	1.90E-005	BIK
1645	A_37_P113078	1.90E-005	AKR7A3
1646	A_37_P140473	1.90E-005	SLC29A1

4976	A_37_P151087	0.00094786	RPL13A
4977	A_37_P286784	0.0009479	BIN2
4978	A_37_P189714	0.00094791	OGFR
4979	A_37_P024511	0.00094939	UGT2A3
4980	A_23_P130906	0.00095231	TEX101
4981	A_37_P263483	0.00095717	PDXDC1
4982	A_37_P247667	0.00095869	CPZ
4983	A_37_P216603	0.0009595	C13orf28
4984	A_37_P117340	0.00096204	HSD11B1
4985	A_37_P117492	0.00096268	NUP210L
4986	A_37_P048454	0.00096269	BOLA3
4987	A_37_P418724	0.00096336	FXYD4
4988	A_37_P087824	0.00096432	SMG6
4989	A_33_P3268622	0.00096456	LY6D
4990	A_37_P432929	0.00096511	C2CD3
4991	A_37_P260901	0.00096536	NTN3
4992	A_37_P020201	0.00096608	SLC38A3
4993	A_37_P426428	0.00096751	CCDC81
4994	A_37_P418990	0.00096799	GRK5
4995	A_37_P357343	0.00097087	ALDH8A1
4996	A_37_P275859	0.00097215	PPP1R1C
4997	A_24_P4334	0.00097274	RNF38
4998	A_37_P044618	0.00097322	EEF1B2
4999	A_37_P380545	0.0009743	DBNL
5000	A_37_P350460	0.00097637	RPS6KA1
5001	A_37_P066921	0.00097814	SLC17A8
5002	A_37_P424204	0.00098342	FGF8
5003	A_37_P071483	0.00098414	SLC7A3
5004	A_37_P086091	0.00098469	NTN1
5005	A_37_P149735	0.00098666	C3
5006	A_37_P313346	0.00098727	WNK4
5007	A_37_P417274	0.00099394	NPFFR1
5008	A_37_P113049	0.00099452	CLCNKA
5009	A_37_P066700	0.00099668	CAMKK2
5010	A_37_P239204	0.00099948	LMCD1
5011	A_37_P139256	0.0010001	PRICKLE4
5012	A_37_P387899	0.00100041	TMEM130
5013	A_37_P261311	0.00100247	PLEKHG4
5014	A_37_P024542	0.00100272	UGT2B17
5015	A_37_P339560	0.00100858	FMO3
5016	A_37_P420400	0.0010092	TLL2
5017	A_37_P388654	0.00101032	WEE2
5018	A_37_P133501	0.00101171	UNC5CL
5019	A_23_P308305	0.00101657	TTC39C
5020	A_37_P417487	0.00102285	PIK3AP1
5021	A_37_P215179	0.00102321	MX2
5022	A_37_P209496	0.00102462	IL18
5023	A_37_P069204	0.00102484	CITED1
5024	A_37_P124150	0.0010269	TMEM52
5025	A_37_P356260	0.001027	COL12A1
5026	A_37_P228595	0.00102718	HINT1
5027	A_37_P397156	0.00102726	AQP9
5028	A_37_P008468	0.00102867	FAT2
5029	A_37_P000919	0.00102933	ADAMTS2
5030	A_37_P019976	0.00102965	SETMAR
5031	A_37_P009248	0.00103289	WWC1
5032	A_37_P117933	0.00104019	TMEM206
5033	A_37_P120572	0.00104092	PTGER3
5034	A_37_P403019	0.00104175	SEC11A
5035	A_37_P185125	0.00104387	SELO
5036	A_37_P133100	0.00104508	CAP2

1647	A_23_P29124	1.90E-005	GP1BB
1648	A_23_P93311	1.90E-005	DDR1
1649	A_37_P414873	1.91E-005	TPD52L2
1650	A_37_P284223	1.91E-005	LMBR1L
1651	A_24_P19677	1.91E-005	IL28RA
1652	A_37_P165762	1.91E-005	AGPAT6
1653	A_37_P014236	1.92E-005	GYG1
1654	A_37_P199814	1.92E-005	FAM25C
1655	A_37_P003152	1.92E-005	DBN1
1656	A_37_P018160	1.92E-005	PARP3
1657	A_33_P3233871	1.92E-005	F12
1658	A_37_P105739	1.92E-005	METT11D1
1659	A_37_P069491	1.92E-005	ATG4A
1660	A_37_P256840	1.93E-005	AMFR
1661	A_37_P167424	1.93E-005	EIF4EBP1
1662	A_37_P410374	1.93E-005	ADA
1663	A_37_P296476	1.94E-005	NXF5
1664	A_37_P070144	1.95E-005	LAGE3
1665	A_37_P060707	1.95E-005	MLF2
1666	A_33_P3228460	1.95E-005	FXYD3
1667	A_37_P137535	1.96E-005	SYNE1
1668	A_37_P058762	1.96E-005	PRKAG1
1669	A_37_P038490	1.96E-005	LRRC29
1670	A_37_P035969	1.96E-005	DOK4
1671	A_37_P120029	1.97E-005	UBE2Q1
1672	A_37_P203517	1.97E-005	DAGLA
1673	A_23_P376735	1.98E-005	ZNF524
1674	A_37_P301469	1.99E-005	NLE1
1675	A_37_P160904	1.99E-005	PMS2
1676	A_37_P185140	1.99E-005	SAMM50
1677	A_37_P171090	2.00E-005	C8orf73
1678	A_37_P145271	2.01E-005	PIP5K1C
1679	A_37_P149163	2.02E-005	C19orf56
1680	A_37_P090615	2.02E-005	ARRDC1
1681	A_37_P204909	2.02E-005	GAL
1682	A_37_P019603	2.02E-005	PCBP4
1683	A_37_P087854	2.02E-005	CBX4
1684	A_37_P210165	2.03E-005	RPUSD4
1685	A_37_P210034	2.03E-005	PYGM
1686	A_33_P3317376	2.04E-005	RHOG
1687	A_37_P257412	2.04E-005	UNKL
1688	A_37_P152603	2.04E-005	TMEM38A
1689	A_37_P116136	2.05E-005	ARHGEF19
1690	A_37_P125636	2.05E-005	PTPRU
1691	A_37_P425980	2.06E-005	C11orf73
1692	A_37_P301742	2.06E-005	TRIM65
1693	A_37_P156840	2.06E-005	C7orf70
1694	A_37_P038696	2.07E-005	SLC9A3R2
1695	A_37_P420075	2.07E-005	OBFC1
1696	A_37_P302837	2.08E-005	CACNG4
1697	A_37_P326759	2.08E-005	C14orf109
1698	A_37_P138622	2.08E-005	RGL2
1699	A_37_P057236	2.08E-005	SSH1
1700	A_37_P133736	2.09E-005	LEMD2
1701	A_24_P89843	2.09E-005	CYHR1
1702	A_37_P352351	2.09E-005	TCEA3
1703	A_37_P258317	2.10E-005	HSF4
1704	A_37_P443128	2.10E-005	RNASEH2B
1705	A_37_P013817	2.10E-005	TUSC2
1706	A_37_P185648	2.10E-005	SUSD2
1707	A_33_P3398107	2.10E-005	SYS1

5037	A_23_P47410	0.001046	ESAM
5038	A_37_P148191	0.00104712	DPF1
5039	A_37_P359495	0.00104724	TREML1
5040	A_37_P124448	0.00104729	PLCH2
5041	A_37_P196830	0.00104948	DNAJB12
5042	A_37_P199513	0.00104973	SEC61A2
5043	A_37_P417636	0.00105088	DKK1
5044	A_37_P142140	0.00105388	ZNF187
5045	A_37_P120041	0.00105473	OLFML2B
5046	A_37_P168087	0.00105605	GLI4
5047	A_37_P187855	0.00105676	ENST00000440534
5048	A_37_P403957	0.00105716	PDCD7
5049	A_37_P311962	0.00105868	SMYD4
5050	A_37_P055868	0.00106199	FBXO41
5051	A_37_P023393	0.00106214	C4orf33
5052	A_37_P363147	0.00106224	PRSS16
5053	A_37_P060343	0.00106271	CSRNP2
5054	A_33_P3252635	0.0010635	ZNF254
5055	A_23_P71830	0.0010642	ZBTB26
5056	A_33_P3301709	0.00106446	GNG4
5057	A_23_P13548	0.0010651	CHRDL2
5058	A_37_P199826	0.00106722	RHOBTB1
5059	A_37_P374269	0.00107257	TNPO2
5060	A_37_P153685	0.00107322	ZNF473
5061	A_37_P333564	0.00107326	ANXA9
5062	A_37_P408469	0.00107529	PARVG
5063	A_37_P064229	0.00107726	MLXIP
5064	A_37_P244987	0.00107888	TP63
5065	A_37_P057601	0.00107936	ANKRD33
5066	A_37_P028677	0.00107939	RGS12
5067	A_37_P152214	0.00107967	LYPD5
5068	A_37_P427458	0.00108788	DIXDC1
5069	A_37_P143670	0.00109016	CBLC
5070	A_37_P048886	0.00109033	GPAT2
5071	A_37_P422000	0.00109079	OLAH
5072	A_37_P085694	0.00109172	NAGS
5073	A_33_P3277674	0.00109225	FBXL22
5074	A_37_P208112	0.00109272	MS4A10
5075	A_37_P055516	0.00109302	TMEM214
5076	A_33_P3307113	0.00109336	MSMP
5077	A_37_P314986	0.00109494	SOHLH1
5078	A_37_P071728	0.00109494	GDPD2
5079	A_37_P112857	0.0010957	TOMM20
5080	A_24_P414658	0.0010957	ENST00000359193
5081	A_37_P301331	0.00109667	ABCA6
5082	A_37_P340445	0.00109733	GUCA2B
5083	A_37_P045252	0.00109756	FER1L5
5084	A_23_P26457	0.0010996	HBA2
5085	A_37_P171631	0.0010998	FAM135B
5086	A_37_P384965	0.00109997	A_37_P384965
5087	A_37_P185244	0.00110078	LRP5L
5088	A_37_P233913	0.00110282	USP4
5089	A_37_P061775	0.00110527	DDX12
5090	A_37_P107355	0.00110764	SPTB
5091	A_37_P132284	0.00110846	BTN3A1
5092	A_37_P032096	0.00110867	C16orf71
5093	A_37_P202277	0.00111282	C11orf74
5094	A_37_P097089	0.00111473	RGS3
5095	A_37_P010249	0.00111656	UROC1
5096	A_37_P151027	0.00111712	CACNA1A
5097	A_33_P3421275	0.00111737	NKX6-3

1708	A_37_P033763	2.11E-005	CTRL
1709	A_37_P210489	2.11E-005	FADS3
1710	A_37_P074427	2.11E-005	IDS
1711	A_23_P374902	2.11E-005	CLDND2
1712	A_37_P085203	2.11E-005	C17orf61
1713	A_37_P132800	2.11E-005	C6orf115
1714	A_37_P091001	2.11E-005	C9orf16
1715	A_37_P289590	2.11E-005	ERP27
1716	A_37_P068832	2.11E-005	NFYB
1717	A_37_P182202	2.12E-005	CRYBB2
1718	A_37_P279271	2.12E-005	PDE6D
1719	A_33_P3210647	2.12E-005	COL16A1
1720	A_37_P171578	2.12E-005	PPAPDC1B
1721	A_37_P149158	2.13E-005	C19orf54
1722	A_37_P108569	2.13E-005	RAGE
1723	A_37_P387137	2.13E-005	RHEB
1724	A_37_P139593	2.13E-005	PTK7
1725	A_37_P245563	2.14E-005	FHIT
1726	A_37_P115064	2.14E-005	ERMAP
1727	A_37_P103243	2.14E-005	C14orf79
1728	A_37_P115939	2.19E-005	GPR153
1729	A_37_P257000	2.19E-005	DEF8
1730	A_37_P268117	2.19E-005	ANKRD39
1731	A_37_P102250	2.19E-005	FECH
1732	A_37_P372589	2.19E-005	ECSIT
1733	A_37_P341696	2.20E-005	DENND2D
1734	A_24_P383762	2.20E-005	RASGEF1C
1735	A_37_P093810	2.22E-005	INVS
1736	A_37_P148158	2.22E-005	SLC25A41
1737	A_37_P123438	2.22E-005	OBSCN
1738	A_33_P3411427	2.23E-005	ZNF837
1739	A_37_P056875	2.23E-005	KIF1A
1740	A_37_P051386	2.23E-005	C2orf64
1741	A_37_P024005	2.23E-005	MFSD7
1742	A_37_P429698	2.23E-005	KCNQ1
1743	A_37_P082416	2.23E-005	ITGB4
1744	A_37_P258340	2.24E-005	TFAP4
1745	A_37_P193696	2.24E-005	CUTC
1746	A_37_P310373	2.24E-005	INPP5K
1747	A_23_P162142	2.24E-005	TSKU
1748	A_37_P032006	2.25E-005	C16orf45
1749	A_37_P104250	2.26E-005	FAM177A1
1750	A_37_P030916	2.26E-005	CHMP1A
1751	A_37_P054519	2.26E-005	SPAG16
1752	A_37_P124163	2.27E-005	TMEM54
1753	A_37_P217744	2.27E-005	DCUN1D2
1754	A_37_P369190	2.27E-005	FAM98C
1755	A_37_P128424	2.28E-005	RABIF
1756	A_37_P208371	2.28E-005	PTPRCAP
1757	A_37_P263071	2.29E-005	TGFB1I1
1758	A_37_P326680	2.29E-005	BTBD6
1759	A_37_P031135	2.30E-005	KIFC3
1760	A_37_P270451	2.30E-005	INO80B
1761	A_37_P416365	2.31E-005	C10orf116
1762	A_24_P244699	2.31E-005	NUDT15
1763	A_37_P263264	2.31E-005	TAF1C
1764	A_37_P104537	2.31E-005	GMPR2
1765	A_37_P413914	2.32E-005	PTPRA
1766	A_37_P240993	2.32E-005	MRAS
1767	A_37_P131022	2.32E-005	TRIM62
1768	A_37_P176992	2.32E-005	AGPHD1

5098	A_37_P189772	0.00111757	SDCBP2
5099	A_37_P076579	0.00111761	UBA1
5100	A_37_P084370	0.00111808	MYBBP1A
5101	A_37_P062639	0.00111851	OASL
5102	A_37_P108859	0.0011187	NIN
5103	A_37_P040614	0.00111905	ABCG8
5104	A_37_P365763	0.00112008	KIF13A
5105	A_37_P218589	0.0011206	MYCBP2
5106	A_37_P202411	0.0011229	TRIM66
5107	A_37_P427119	0.0011234	CTNND1
5108	A_37_P150063	0.00112712	PIAS4
5109	A_37_P405627	0.00112757	CCDC157
5110	A_37_P267581	0.00112808	CYP27A1
5111	A_37_P077459	0.00112939	ACSF2
5112	A_37_P341768	0.0011319	NVL
5113	A_37_P268986	0.00113201	GPR113
5114	A_37_P060926	0.00113449	FBXW8
5115	A_33_P3221568	0.00113884	ARMC5
5116	A_37_P363378	0.00113949	IP6K3
5117	A_37_P305649	0.00114157	GRN
5118	A_37_P247631	0.00114167	PRKG2
5119	A_37_P280841	0.0011428	PRB1
5120	A_37_P015428	0.00114302	B4GALT4
5121	A_37_P104425	0.00114302	FNTB
5122	A_37_P213637	0.00114329	RPS4Y2
5123	A_37_P201323	0.00114395	AMPD3
5124	A_37_P166794	0.00114451	CPSF1
5125	A_37_P054749	0.00114577	SSFA2
5126	A_37_P392559	0.00114662	KIAA1967
5127	A_37_P081298	0.00114823	FGF11
5128	A_37_P036685	0.00114856	C16orf89
5129	A_33_P3236133	0.00114907	SHQ1
5130	A_37_P144140	0.00114941	CILP2
5131	A_37_P113434	0.00114941	CRB1
5132	A_24_P383609	0.00115007	NANOS1
5133	A_37_P092735	0.00115018	TMEM8C
5134	A_37_P310300	0.00115095	INCA1
5135	A_37_P079231	0.00115162	CARD14
5136	A_37_P185004	0.00115163	RAB36
5137	A_37_P154511	0.00115166	ABP1
5138	A_23_P130435	0.00115306	LIM2
5139	A_23_P85716	0.00115317	FCGR2A
5140	A_37_P293084	0.00115442	AR
5141	A_37_P385038	0.00115536	ICA1
5142	A_37_P026174	0.00115846	KLKB1
5143	A_37_P163868	0.00115916	STK31
5144	A_37_P058751	0.00116088	KRT83
5145	A_37_P170002	0.00116154	MED30
5146	A_37_P157540	0.00116305	FAM71F1
5147	A_37_P013248	0.00116336	EPHA6
5148	A_37_P174230	0.00116351	TARSL2
5149	A_37_P002131	0.00116658	GNB2L1
5150	A_37_P315740	0.00116735	TNC
5151	A_37_P001895	0.00117115	GLRA1
5152	A_37_P429871	0.00117144	KLC2
5153	A_37_P167166	0.0011729	DNAJC5B
5154	A_37_P415294	0.00117684	ZNF341
5155	A_37_P002450	0.0011778	CTNND2
5156	A_37_P171922	0.00117885	STMN2
5157	A_37_P262617	0.00117932	SPIRE2
5158	A_33_P3279470	0.00117957	AGRP

1769	A_37_P190664	2.33E-005	SLC2A4RG	5159	A_37_P078698	0.00118186	KRT36
1770	A_33_P3224027	2.33E-005	MBD6	5160	A_37_P030720	0.00118495	RWDD4
1771	A_37_P211118	2.33E-005	PCSK7	5161	A_37_P095180	0.00118904	PTPLAD2
1772	A_37_P372323	2.35E-005	MAST3	5162	A_37_P263726	0.00119006	VAT1L
1773	A_37_P134589	2.35E-005	FANCE	5163	A_37_P110701	0.00119108	BCAN
1774	A_37_P165531	2.35E-005	FLJ43860	5164	A_37_P078979	0.00119158	COL1A1
1775	A_37_P179226	2.36E-005	PSTPIP1	5165	A_37_P202921	0.00119496	AHNAK
1776	A_37_P273991	2.36E-005	OTOF	5166	A_37_P040057	0.00119741	VWA3A
1777	A_37_P094259	2.36E-005	BAG1	5167	A_37_P060559	0.00119754	EP400
1778	A_37_P139074	2.36E-005	PPARD	5168	A_37_P155235	0.00120048	NOBOX
1779	A_37_P375384	2.37E-005	JAK3	5169	A_37_P202039	0.00120341	FTH1
1780	A_37_P152900	2.38E-005	FBXO27	5170	A_37_P143075	0.00120344	ACER1
1781	A_37_P190502	2.38E-005	FAM113A	5171	A_37_P127533	0.00120594	PCNXL2
1782	A_37_P204352	2.38E-005	APBB1	5172	A_37_P399561	0.00120594	DAPK2
1783	A_37_P043303	2.38E-005	COX7A2L	5173	A_37_P380250	0.00120677	CPA1
1784	A_37_P383752	2.38E-005	PSMG3	5174	A_37_P064452	0.00120955	MYBPC1
1785	A_37_P214134	2.38E-005	C21orf70	5175	A_37_P061468	0.0012123	GRIN2B
1786	A_37_P253864	2.39E-005	FBXO8	5176	A_37_P155773	0.00121301	FAM188B
1787	A_24_P296457	2.39E-005	PHKG2	5177	A_37_P111097	0.00121429	C1orf125
1788	A_37_P285378	2.40E-005	HNF1A	5178	A_37_P338616	0.00121684	EIF3I
1789	A_37_P039536	2.40E-005	PAM16	5179	A_37_P109696	0.001218	A_37_P109696
1790	A_37_P336657	2.41E-005	CKS1B	5180	A_37_P304611	0.00121825	EPN3
1791	A_37_P402848	2.41E-005	PPCDC	5181	A_37_P261102	0.00121933	PDIA2
1792	A_37_P167612	2.42E-005	FAM160B2	5182	A_37_P413050	0.00122038	MYT1
1793	A_23_P52373	2.42E-005	NDST2	5183	A_37_P181282	0.00122053	ZNF710
1794	A_23_P87150	2.43E-005	LPXN	5184	A_37_P406876	0.00122103	ARVCF
1795	A_37_P161625	2.44E-005	SMARCD3	5185	A_23_P407614	0.00122276	PYDC1
1796	A_37_P219302	2.44E-005	NALCN	5186	A_37_P209429	0.00122424	PHLDB1
1797	A_37_P019843	2.44E-005	SEMA3F	5187	A_37_P033491	0.00122525	DUS2L
1798	A_37_P411992	2.45E-005	HSPA12B	5188	A_37_P011613	0.0012253	LAMB2
1799	A_37_P100920	2.46E-005	DYM	5189	A_37_P321404	0.00122911	CCBL1
1800	A_37_P059642	2.46E-005	CUX2	5190	A_37_P433663	0.00122998	ETS1
1801	A_33_P3289845	2.46E-005	IGFL1	5191	A_37_P084624	0.00123039	LRRC46
1802	A_37_P205808	2.47E-005	DEAF1	5192	A_37_P101434	0.00123144	MYOM1
1803	A_33_P3379669	2.47E-005	RNF208	5193	A_37_P216621	0.00123242	C13orf33
1804	A_37_P185009	2.47E-005	NAGA	5194	A_37_P121020	0.00123251	SH2D1B
1805	A_37_P034222	2.48E-005	GNPTG	5195	A_37_P085060	0.00123293	C17orf28
1806	A_37_P024456	2.48E-005	VEGFC	5196	A_37_P155825	0.00123328	COL28A1
1807	A_37_P169999	2.49E-005	HRSP12	5197	A_37_P271140	0.00123329	SCN3A
1808	A_37_P174529	2.50E-005	CRTC3	5198	A_37_P067863	0.00123631	PPP1R1A
1809	A_33_P3231613	2.51E-005	CSAG3	5199	A_37_P022168	0.00123877	PEX5L
1810	A_37_P039461	2.51E-005	TMEM188	5200	A_37_P088834	0.00124171	TMC8
1811	A_37_P041264	2.53E-005	FNDC4	5201	A_37_P046472	0.0012427	MPP4
1812	A_37_P290223	2.55E-005	R3HDM2	5202	A_37_P071692	0.00124357	ARHGEF9
1813	A_37_P408986	2.55E-005	Sep-05	5203	A_37_P206196	0.00124478	HBE1
1814	A_37_P260822	2.56E-005	NME4	5204	A_37_P043830	0.00124521	TMEM18
1815	A_37_P349391	2.57E-005	PUSL1	5205	A_37_P187637	0.00124532	DEFB115
1816	A_37_P376889	2.57E-005	VAV1	5206	A_33_P3406505	0.00124565	ELL
1817	A_37_P142086	2.58E-005	TRAF3IP2	5207	A_37_P091946	0.00124596	SARDH
1818	A_37_P264433	2.58E-005	ACCN4	5208	A_37_P020034	0.00124613	PCOLCE2
1819	A_37_P377501	2.59E-005	ZNF551	5209	A_37_P068289	0.00124613	MANSC1
1820	A_32_P113436	2.59E-005	HNRNPA1L2	5210	A_37_P397661	0.00124877	ADAMTS17
1821	A_37_P028217	2.59E-005	C4orf14	5211	A_37_P020998	0.00124896	TGM4
1822	A_37_P085325	2.59E-005	C17orf63	5212	A_37_P256113	0.00125421	MEFV
1823	A_37_P229906	2.60E-005	CDHR2	5213	A_37_P022043	0.00125678	ZNF197
1824	A_37_P042692	2.60E-005	CAPN10	5214	A_37_P019521	0.0012572	CACNA2D2
1825	A_37_P059656	2.60E-005	AMN1	5215	A_37_P310814	0.00126167	RNF112
1826	A_37_P249052	2.60E-005	HADH	5216	A_37_P182212	0.00126255	CSDC2
1827	A_37_P401911	2.61E-005	C15orf33	5217	A_37_P089878	0.00126319	SKAP1
1828	A_37_P152104	2.61E-005	SIGLEC12	5218	A_37_P093412	0.00126554	RPL12
1829	A_37_P073409	2.61E-005	DNASE1L1	5219	A_37_P165884	0.00126637	SNX16

1830	A_23_P56978	2.61E-005	PTK6
1831	A_37_P245117	2.62E-005	RAF1
1832	A_37_P145363	2.62E-005	APBA3
1833	A_33_P3246418	2.62E-005	MDFI
1834	A_37_P068326	2.63E-005	TSPAN31
1835	A_37_P113320	2.63E-005	ALG14
1836	A_37_P190645	2.63E-005	SLC24A3
1837	A_37_P306624	2.63E-005	KSR1
1838	A_37_P155623	2.63E-005	GET4
1839	A_33_P3335606	2.64E-005	FAM58B
1840	A_37_P204126	2.64E-005	EI24
1841	A_37_P202290	2.64E-005	C11orf80
1842	A_37_P061015	2.65E-005	VWF
1843	A_37_P336117	2.65E-005	COL8A2
1844	A_37_P337250	2.66E-005	ALX3
1845	A_37_P079117	2.66E-005	SUMO2
1846	A_37_P427549	2.66E-005	DNHD1
1847	A_37_P435928	2.66E-005	MAP3K11
1848	A_37_P139114	2.66E-005	PPP1R11
1849	A_37_P126471	2.66E-005	CELA2B
1850	A_37_P181894	2.66E-005	CBY1
1851	A_37_P150746	2.67E-005	QPCTL
1852	A_23_P254944	2.68E-005	GSTT1
1853	A_37_P286309	2.69E-005	LETMD1
1854	A_37_P416646	2.69E-005	ADAM8
1855	A_37_P200615	2.69E-005	WDFY4
1856	A_37_P149090	2.69E-005	C19orf29
1857	A_37_P422314	2.69E-005	PANK1
1858	A_37_P299287	2.69E-005	FAM122B
1859	A_37_P368859	2.69E-005	EIF3K
1860	A_37_P382637	2.69E-005	ATXN7L1
1861	A_33_P3263902	2.69E-005	MXI1
1862	A_37_P200847	2.71E-005	ZNF511
1863	A_37_P204803	2.71E-005	FOLR4
1864	A_37_P409642	2.71E-005	UQCR10
1865	A_37_P226242	2.71E-005	FAM174A
1866	A_37_P126498	2.71E-005	TMCO4
1867	A_37_P163582	2.71E-005	SLC37A3
1868	A_37_P080351	2.71E-005	DHRS11
1869	A_37_P028295	2.72E-005	C4orf36
1870	A_37_P141371	2.73E-005	TPD52L1
1871	A_37_P111243	2.73E-005	C1orf187
1872	A_37_P352772	2.73E-005	MAD2L2
1873	A_33_P3217119	2.74E-005	OSCP1
1874	A_33_P3417195	2.74E-005	C17orf82
1875	A_37_P265076	2.74E-005	ANXA4
1876	A_37_P126665	2.75E-005	SMG5
1877	A_37_P211465	2.75E-005	TAF10
1878	A_37_P198406	2.75E-005	PAX2
1879	A_37_P013054	2.75E-005	EIF1B
1880	A_37_P070457	2.76E-005	COL4A5
1881	A_37_P373616	2.77E-005	RPS16
1882	A_37_P090205	2.77E-005	ABCA2
1883	A_37_P039133	2.77E-005	FAM96B
1884	A_37_P255202	2.78E-005	NME3
1885	A_37_P290434	2.78E-005	SETD1B
1886	A_37_P081384	2.79E-005	CYB561
1887	A_37_P111033	2.79E-005	C1orf106
1888	A_37_P270225	2.79E-005	GTDC1
1889	A_37_P185478	2.79E-005	SLC2A11
1890	A_37_P105929	2.80E-005	C14orf138

5220	A_37_P254519	0.0012678	PF4
5221	A_37_P137511	0.0012689	ZDHHC14
5222	A_37_P228554	0.00126985	SLIT3
5223	A_37_P368325	0.00127179	CYP4F22
5224	A_37_P322181	0.00127377	TYRP1
5225	A_37_P164888	0.00127599	WBSCR17
5226	A_37_P093151	0.00127991	RC3H2
5227	A_23_P110851	0.0012831	TERT
5228	A_37_P151542	0.00128838	SIGLEC9
5229	A_37_P366851	0.00128934	BEST2
5230	A_37_P131809	0.00128992	TAGAP
5231	A_24_P239419	0.00129295	EPM2A
5232	A_37_P133814	0.00129333	CYP21A2
5233	A_23_P31945	0.0012949	IL33
5234	A_37_P132598	0.00129707	C2
5235	A_37_P377428	0.00129707	ZNF527
5236	A_33_P3302312	0.00129717	IER5L
5237	A_37_P034464	0.00129941	PKMYT1
5238	A_37_P147567	0.001301	DMKN
5239	A_37_P310786	0.00130226	QRICH2
5240	A_24_P336848	0.00130255	ACYP2
5241	A_37_P124714	0.00130306	ILDR2
5242	A_37_P098436	0.00130362	TTC16
5243	A_37_P159028	0.0013042	MRPS33
5244	A_37_P405836	0.00130688	ALG12
5245	A_37_P194019	0.00130772	ANKRD22
5246	A_37_P372992	0.00130851	NCAN
5247	A_37_P184027	0.00131497	LZTR1
5248	A_37_P236316	0.00131732	DNAH1
5249	A_23_P392126	0.00131815	C17orf108
5250	A_37_P033533	0.00132102	EDC4
5251	A_37_P080462	0.00132309	DNAH2
5252	A_37_P305596	0.00132433	GPRC5C
5253	A_37_P088588	0.00132814	TBKBP1
5254	A_37_P216053	0.00132848	RIPK4
5255	A_37_P086687	0.00132997	ERN1
5256	A_37_P132398	0.00133058	COL11A2
5257	A_37_P311025	0.00133165	ITGA2B
5258	A_37_P121311	0.00133598	MACF1
5259	A_37_P130543	0.00133748	WDR64
5260	A_37_P382032	0.00134032	GRID2IP
5261	A_37_P029159	0.00134867	SLC34A2
5262	A_37_P294027	0.00134878	CLCN4
5263	A_37_P389413	0.00134984	ZNF16
5264	A_37_P227210	0.0013499	NIPAL4
5265	A_37_P411742	0.00135163	NINL
5266	A_37_P413488	0.00135261	OXT
5267	A_37_P086752	0.00135284	INTS2
5268	A_37_P081805	0.00135439	GLTPD2
5269	A_37_P168080	0.00135449	STAR
5270	A_37_P202254	0.00135583	C11orf66
5271	A_37_P236505	0.00135952	DOCK3
5272	A_37_P388894	0.00136063	ZNF138
5273	A_37_P042963	0.00136657	ZNF385B
5274	A_37_P225120	0.00137411	TMEM173
5275	A_37_P041526	0.00138174	ARHGEF4
5276	A_37_P193225	0.00138226	CCNJ
5277	A_37_P196205	0.00138226	LIPN
5278	A_37_P140890	0.00138257	KCNK5
5279	A_37_P095373	0.00138332	SH2D3C
5280	A_37_P172215	0.00138339	TMEM67

1891	A_37_P041969	2.81E-005	CNRIP1
1892	A_37_P176261	2.82E-005	JMJD7-PLA2G4B
1893	A_37_P020606	2.83E-005	STAB1
1894	A_37_P389123	2.83E-005	ZNHIT1
1895	A_37_P091223	2.84E-005	PLIN2
1896	A_37_P290579	2.84E-005	SHMT2
1897	A_37_P099928	2.84E-005	ANKRD29
1898	A_37_P079325	2.85E-005	CBX2
1899	A_37_P316713	2.85E-005	FRMPD1
1900	A_37_P321939	2.86E-005	C9orf30-TMEFF1
1901	A_37_P159024	2.86E-005	MRPS24
1902	A_37_P135777	2.86E-005	ATG5
1903	A_37_P208345	2.86E-005	EEF1G
1904	A_37_P145306	2.87E-005	EVI5L
1905	A_37_P223803	2.87E-005	RNF130
1906	A_23_P125233	2.88E-005	CNN1
1907	A_37_P031502	2.88E-005	SULT1A2
1908	A_37_P078068	2.89E-005	ARSG
1909	A_37_P191408	2.89E-005	NEURL2
1910	A_37_P072206	2.90E-005	IGBP1
1911	A_37_P024092	2.90E-005	CYP4V2
1912	A_37_P279675	2.90E-005	TWIST2
1913	A_37_P364772	2.90E-005	TBP
1914	A_37_P305944	2.90E-005	MPP3
1915	A_37_P432507	2.91E-005	NR1H3
1916	A_37_P103712	2.91E-005	PIGH
1917	A_37_P430001	2.91E-005	LDLRAD3
1918	A_37_P192792	2.91E-005	C10orf57
1919	A_37_P175609	2.91E-005	RPAP1
1920	A_37_P194214	2.92E-005	ANXA11
1921	A_37_P140434	2.92E-005	SLC22A3
1922	A_37_P331463	2.93E-005	SLC38A6
1923	A_37_P152967	2.93E-005	FCGBP
1924	A_37_P331352	2.93E-005	SIVA1
1925	A_24_P17719	2.93E-005	KLHL5
1926	A_37_P414481	2.93E-005	SNAP25
1927	A_37_P214951	2.93E-005	BRWD1
1928	A_37_P023897	2.94E-005	COPS4
1929	A_37_P170400	2.94E-005	OSGIN2
1930	A_37_P207261	2.94E-005	SLC25A45
1931	A_37_P111394	2.94E-005	C1orf66
1932	A_37_P296501	2.94E-005	ZDHHC9
1933	A_37_P439859	2.95E-005	WRB
1934	A_37_P216793	2.95E-005	CDC16
1935	A_37_P076844	2.96E-005	VMA21
1936	A_37_P188432	2.96E-005	KCNK15
1937	A_37_P081770	2.97E-005	TBC1D3B
1938	A_37_P089087	2.97E-005	TOM1L1
1939	A_37_P182535	2.97E-005	ANKRD54
1940	A_37_P211942	2.97E-005	TIRAP
1941	A_37_P024592	2.97E-005	F11
1942	A_37_P174274	2.98E-005	CHAC1
1943	A_37_P036475	2.98E-005	NPRL3
1944	A_23_P104705	2.98E-005	SLC29A2
1945	A_33_P3258265	2.98E-005	SEMA6C
1946	A_37_P287950	2.98E-005	MMP17
1947	A_37_P139263	2.99E-005	TOMM6
1948	A_37_P217370	3.00E-005	FAM70B
1949	A_37_P155791	3.01E-005	PRKAG2
1950	A_37_P035141	3.01E-005	LCMT1
1951	A_37_P001102	3.01E-005	TMEM161B

5281	A_37_P177042	0.00138388	ZNF280D
5282	A_33_P3419321	0.00138428	CHD5
5283	A_37_P121012	0.00138839	HMGCS2
5284	A_33_P3408722	0.00138929	C9orf25
5285	A_37_P115091	0.00139094	USH2A
5286	A_33_P3343872	0.00139185	SIRPG
5287	A_37_P104824	0.0013929	IRF9
5288	A_37_P021066	0.00139342	TM4SF4
5289	A_37_P152572	0.00139347	KHSRP
5290	A_37_P379081	0.00139439	BMPER
5291	A_37_P269183	0.00139601	UXS1
5292	A_37_P065885	0.00139858	NACA
5293	A_37_P033516	0.00140066	E4F1
5294	A_37_P191411	0.00140114	VSTM2L
5295	A_37_P008343	0.0014013	CCNJL
5296	A_37_P392436	0.00140675	KCNU1
5297	A_37_P339285	0.00140876	FBXO6
5298	A_37_P173979	0.00140906	ADAMTS7
5299	A_23_P107963	0.00141111	FUT1
5300	A_37_P072668	0.00141198	HCFC1
5301	A_37_P306101	0.00141302	ITGA3
5302	A_37_P138958	0.00141582	PLG
5303	A_37_P029616	0.00141914	TADA2B
5304	A_37_P100650	0.00141961	DLGAP1
5305	A_37_P156264	0.00142024	LAMB4
5306	A_37_P011188	0.00142281	CACNA1D
5307	A_37_P369471	0.00142305	GAPDHS
5308	A_37_P411414	0.00142305	GMEB2
5309	A_37_P135078	0.00142481	GLP1R
5310	A_37_P423183	0.00142609	SEC24C
5311	A_37_P149154	0.00142875	C19orf51
5312	A_37_P146956	0.00142965	KIR3DL3
5313	A_37_P329150	0.00142975	HEATR4
5314	A_23_P127068	0.00143109	SEMA4G
5315	A_37_P133872	0.00143176	DAAM2
5316	A_33_P3222565	0.00143291	TSPAN14
5317	A_37_P348238	0.00143408	PLEKHN1
5318	A_37_P088863	0.00143645	TMEM104
5319	A_37_P282997	0.00143858	GCN1L1
5320	A_37_P407117	0.00143988	INPP5J
5321	A_37_P148551	0.00144004	Mar-02
5322	A_37_P169748	0.00144079	RPL30
5323	A_37_P266375	0.00144265	SLC4A5
5324	A_37_P065493	0.00144272	PAN2
5325	A_23_P29975	0.00144294	C4orf19
5326	A_37_P055241	0.00145001	FAP
5327	A_37_P077741	0.00145058	KLHL11
5328	A_37_P360338	0.00145067	BAG6
5329	A_33_P3295786	0.00145186	FAM159A
5330	A_37_P436942	0.00145235	UTY
5331	A_37_P085194	0.00145366	C17orf56
5332	A_24_P399980	0.0014576	HEPH
5333	A_37_P199851	0.00145821	SORCS3
5334	A_37_P395745	0.0014593	KCNQ3
5335	A_37_P362924	0.00146047	C6orf138
5336	A_33_P3259183	0.00146256	FAM78B
5337	A_37_P034900	0.00146259	JMJD5
5338	A_37_P081197	0.00146305	TBC1D3F
5339	A_37_P298833	0.0014636	PTCHD1
5340	A_23_P118122	0.00146562	RGS11
5341	A_37_P259200	0.00146922	MTHFSD

1952	A_37_P280991	3.02E-005	ACSS3
1953	A_37_P062728	3.02E-005	DIABLO
1954	A_37_P091106	3.02E-005	C9orf89
1955	A_37_P326468	3.02E-005	ABCD4
1956	A_37_P017308	3.03E-005	TMEM41A
1957	A_37_P303212	3.04E-005	C17orf106
1958	A_37_P350041	3.06E-005	RNF2
1959	A_37_P029869	3.06E-005	TMEM129
1960	A_37_P115563	3.06E-005	FBXO44
1961	A_37_P176434	3.06E-005	DET1
1962	A_37_P141053	3.06E-005	WASF1
1963	A_37_P140960	3.06E-005	WDR27
1964	A_23_P118693	3.08E-005	FOXN1
1965	A_37_P110276	3.09E-005	TDRKH
1966	A_37_P037926	3.10E-005	RAB26
1967	A_37_P005703	3.10E-005	NEURL1B
1968	A_37_P168387	3.10E-005	HGSNAT
1969	A_37_P022781	3.10E-005	UFSP2
1970	A_37_P161157	3.11E-005	PDGFA
1971	A_37_P327268	3.11E-005	CGRRF1
1972	A_37_P441314	3.11E-005	GPR180
1973	A_37_P171157	3.12E-005	PEX2
1974	A_37_P367209	3.12E-005	XRCC1
1975	A_23_P43763	3.13E-005	PLLP
1976	A_37_P151475	3.13E-005	SF3A2
1977	A_37_P019263	3.13E-005	RPUSD3
1978	A_37_P020553	3.14E-005	SS18L2
1979	A_37_P013059	3.14E-005	EIF2A
1980	A_37_P010161	3.15E-005	ABHD14B
1981	A_37_P202158	3.15E-005	C11orf31
1982	A_23_P101871	3.15E-005	FBXO17
1983	A_33_P3241937	3.17E-005	TBC1D16
1984	A_37_P085160	3.17E-005	C17orf37
1985	A_23_P138125	3.17E-005	FAIM3
1986	A_24_P822704	3.17E-005	TMEM198
1987	A_37_P166525	3.18E-005	CHRAC1
1988	A_37_P066650	3.18E-005	POU6F1
1989	A_37_P302491	3.19E-005	C17orf49
1990	A_37_P217781	3.19E-005	ING1
1991	A_37_P143257	3.19E-005	ADAMTS10
1992	A_37_P082830	3.19E-005	PLD6
1993	A_37_P313778	3.21E-005	NINJ1
1994	A_37_P017358	3.21E-005	RFT1
1995	A_23_P320878	3.21E-005	FAM119B
1996	A_37_P371827	3.21E-005	DOCK6
1997	A_37_P123974	3.21E-005	PEAR1
1998	A_37_P148843	3.22E-005	WDR83
1999	A_37_P043111	3.22E-005	CHRNG
2000	A_37_P156412	3.22E-005	NPC1L1
2001	A_37_P168650	3.22E-005	MRPL13
2002	A_37_P202839	3.23E-005	CD44
2003	A_37_P119410	3.24E-005	DLGAP3
2004	A_33_P3278573	3.25E-005	MAGIX
2005	A_37_P323789	3.26E-005	EMILIN2
2006	A_37_P163719	3.26E-005	SSPO
2007	A_37_P068591	3.27E-005	NCKAP5L
2008	A_37_P433020	3.27E-005	IGSF22
2009	A_37_P398961	3.28E-005	TNFAIP8L3
2010	A_37_P039818	3.28E-005	CDH16
2011	A_33_P3229276	3.29E-005	ZSCAN2
2012	A_37_P055342	3.30E-005	THSD7B

5342	A_37_P308264	0.00146923	LPO
5343	A_33_P3266429	0.00147305	SAMD13
5344	A_37_P024643	0.00147336	FAM149A
5345	A_33_P3299220	0.00147399	ADAMTSL4
5346	A_37_P193721	0.00147624	CYP2C18
5347	A_37_P188301	0.0014774	ID1
5348	A_37_P204638	0.00147751	FERMT3
5349	A_37_P016701	0.00147776	TLR9
5350	A_37_P152555	0.00147927	TMEM146
5351	A_37_P080592	0.00147934	DNAH9
5352	A_37_P309140	0.0014811	C17orf76
5353	A_37_P320189	0.0014812	POMT1
5354	A_37_P202182	0.00148147	C11orf42
5355	A_37_P175468	0.00148221	STRC
5356	A_37_P199555	0.00148512	LRRC20
5357	A_37_P090779	0.0014853	CNTNAP3
5358	A_37_P277366	0.00148867	SEMA4F
5359	A_37_P210731	0.00148867	SYVN1
5360	A_37_P171994	0.0014911	LYPD2
5361	A_37_P014154	0.00149557	GRM7
5362	A_37_P300822	0.00149613	CFP
5363	A_37_P087497	0.00149687	CAMKK1
5364	A_37_P007287	0.00150565	IRX4
5365	A_37_P046498	0.00150927	HTRA2
5366	A_37_P208551	0.00151531	SPI1
5367	A_37_P285417	0.00151572	HOXC5
5368	A_37_P011804	0.00151833	CLDN11
5369	A_37_P016327	0.00151951	MUC13
5370	A_37_P155619	0.00152009	C7orf16
5371	A_37_P096593	0.00152316	PTGDS
5372	A_37_P127643	0.00152923	SLAMF8
5373	A_37_P249737	0.00152929	KIAA1109
5374	A_23_P423331	0.00153225	NTNG2
5375	A_23_P139654	0.00153459	KLRC1
5376	A_37_P089044	0.00153677	CD7
5377	A_37_P028147	0.00153902	PIGG
5378	A_37_P375648	0.00153904	SIGLEC11
5379	A_37_P020954	0.00154259	TF
5380	A_37_P059426	0.00154454	CLSTN3
5381	A_37_P246835	0.00154861	ACSL1
5382	A_23_P120472	0.00155006	TFAP2C
5383	A_37_P086900	0.00155193	RAPGEFL1
5384	A_33_P3395804	0.00155468	C20orf26
5385	A_37_P402557	0.00155576	RGMA
5386	A_37_P141295	0.0015563	TMEM63B
5387	A_37_P123844	0.00155736	IGSF9
5388	A_37_P065201	0.00156072	P2RX2
5389	A_37_P319970	0.00156072	ENTPD8
5390	A_37_P153214	0.00156238	MAP4K1
5391	A_37_P276556	0.00156525	REG3G
5392	A_37_P158385	0.00156591	GTF2IRD2
5393	A_37_P111082	0.00156895	C1orf115
5394	A_37_P378284	0.00157421	ADAM22
5395	A_37_P151975	0.00157431	SPRED3
5396	A_37_P306112	0.00157739	ITGB3
5397	A_23_P120103	0.00158211	KCNS3
5398	A_37_P053161	0.00158272	FABP1
5399	A_37_P243165	0.00158454	RPSA
5400	A_37_P121496	0.00158589	DUSP10
5401	A_23_P208866	0.0015918	GMFG
5402	A_37_P359504	0.00159213	TREML2

2013	A_37_P146203	3.30E-005	CCDC159
2014	A_23_P58002	3.30E-005	TCTA
2015	A_37_P360780	3.31E-005	MTRF1L
2016	A_37_P081352	3.31E-005	MMD
2017	A_33_P3311371	3.31E-005	PDLIM2
2018	A_37_P165407	3.32E-005	ZNF862
2019	A_37_P046500	3.32E-005	IAH1
2020	A_37_P022189	3.32E-005	CHDH
2021	A_37_P126858	3.32E-005	RWDD3
2022	A_23_P27734	3.34E-005	NPAS1
2023	A_37_P152178	3.34E-005	SUV420H2
2024	A_37_P119063	3.34E-005	BATF3
2025	A_37_P303376	3.35E-005	CORO6
2026	A_37_P187940	3.36E-005	ERGIC3
2027	A_23_P107283	3.36E-005	HOXB2
2028	A_37_P155667	3.36E-005	C7orf41
2029	A_37_P297274	3.36E-005	SMS
2030	A_37_P434762	3.37E-005	CATSPER1
2031	A_37_P208312	3.37E-005	MUC5B
2032	A_37_P206033	3.37E-005	DENND5A
2033	A_37_P377145	3.37E-005	SIRT6
2034	A_37_P393835	3.38E-005	EEF1D
2035	A_37_P124084	3.39E-005	STK40
2036	A_37_P307269	3.41E-005	SLC16A5
2037	A_37_P077587	3.41E-005	FLOT2
2038	A_23_P91104	3.42E-005	KCNK3
2039	A_37_P062125	3.42E-005	KCNMB4
2040	A_37_P215756	3.42E-005	SIM2
2041	A_37_P182940	3.43E-005	TRMT2A
2042	A_37_P162106	3.43E-005	PRKAR2B
2043	A_37_P438220	3.43E-005	SETD4
2044	A_37_P090224	3.43E-005	ADAMTS13
2045	A_37_P101120	3.43E-005	C18orf56
2046	A_37_P262018	3.44E-005	RUNDC2C
2047	A_37_P266693	3.44E-005	SLC5A6
2048	A_37_P131316	3.45E-005	ZNF692
2049	A_37_P021910	3.46E-005	XYLB
2050	A_37_P124637	3.46E-005	PODN
2051	A_33_P3386364	3.46E-005	FANCF
2052	A_37_P437803	3.46E-005	C21orf33
2053	A_37_P203296	3.46E-005	CRY2
2054	A_37_P295753	3.47E-005	ASMTL
2055	A_37_P202481	3.47E-005	C2CD2L
2056	A_37_P127833	3.49E-005	CASP9
2057	A_37_P430461	3.49E-005	MTMR2
2058	A_37_P159217	3.50E-005	BAIAP2L1
2059	A_37_P088011	3.50E-005	SP2
2060	A_37_P367056	3.50E-005	C19orf40
2061	A_37_P417927	3.50E-005	EIF4EBP2
2062	A_37_P211860	3.50E-005	UCP2
2063	A_33_P3376527	3.51E-005	CHST10
2064	A_23_P350551	3.51E-005	C12orf57
2065	A_37_P355385	3.52E-005	ANKRD6
2066	A_37_P143971	3.52E-005	PRKD2
2067	A_37_P199937	3.52E-005	CCDC3
2068	A_37_P255149	3.52E-005	SLC12A4
2069	A_37_P364198	3.53E-005	RSPH3
2070	A_37_P147724	3.53E-005	SSC5D
2071	A_37_P369280	3.53E-005	GPR108
2072	A_37_P283062	3.53E-005	SLC6A12
2073	A_37_P121027	3.55E-005	SH2D2A

5403	A_37_P037736	0.00159256	ERI2
5404	A_37_P166917	0.00159339	LGI3
5405	A_37_P076113	0.00159393	SYTL5
5406	A_37_P178733	0.00159912	OSTBETA
5407	A_23_P131215	0.0015997	CRYGD
5408	A_24_P212234	0.0016056	SLC6A18
5409	A_37_P087740	0.00160626	CASKIN2
5410	A_37_P211754	0.00160782	VPS37C
5411	A_37_P101405	0.00160855	PIK3C3
5412	A_37_P310259	0.00160884	PPP1R1B
5413	A_37_P022769	0.00160977	ARHGAP24
5414	A_37_P121877	0.00161057	MOSC2
5415	A_37_P210296	0.00161367	IRF7
5416	A_37_P276103	0.00161369	PSD4
5417	A_37_P092964	0.00161503	FOXD4L1
5418	A_37_P378734	0.00161543	ENST00000417078
5419	A_37_P093884	0.00161715	KDM4C
5420	A_32_P222684	0.00161922	PRDM6
5421	A_37_P058609	0.00161965	NOP2
5422	A_37_P106375	0.00161987	MYH6
5423	A_37_P163409	0.00162129	JHDM1D
5424	A_33_P3419557	0.00162214	OR5K3
5425	A_37_P263471	0.00162502	SRL
5426	A_23_P362228	0.00162508	C1orf213
5427	A_37_P105555	0.00162534	SLC25A21
5428	A_37_P239121	0.00162574	LARS2
5429	A_37_P059532	0.00162601	NPFF
5430	A_37_P302647	0.0016276	SLC4A1
5431	A_37_P270777	0.00162882	TFCP2L1
5432	A_33_P3384052	0.00163061	RAX
5433	A_37_P360881	0.00163249	BTNL2
5434	A_37_P405551	0.00163482	TMPRSS6
5435	A_37_P151729	0.00163887	SLC5A5
5436	A_24_P109069	0.00163934	SYT15
5437	A_37_P096173	0.00163999	ENG
5438	A_37_P050305	0.0016406	SCTR
5439	A_37_P226148	0.00164372	FABP6
5440	A_33_P3285299	0.00164533	GPRIN2
5441	A_37_P216916	0.0016461	COG3
5442	A_37_P125725	0.00164679	RAB25
5443	A_33_P3298539	0.00165961	APOA1
5444	A_37_P123516	0.00165972	OLFML3
5445	A_23_P169437	0.00165972	LCN2
5446	A_37_P155601	0.00166428	ZNF479
5447	A_37_P109145	0.0016654	SLAMF1
5448	A_37_P393492	0.00166755	LRRC14
5449	A_37_P001459	0.00166988	GDNF
5450	A_37_P329382	0.00166992	DPF3
5451	A_37_P152293	0.00167249	TBCB
5452	A_37_P168207	0.00167386	GRHL2
5453	A_37_P321322	0.00167564	SLC34A3
5454	A_37_P234980	0.00167884	CAND2
5455	A_37_P019713	0.00168749	POU1F1
5456	A_37_P146066	0.00168795	GRIN2D
5457	A_37_P000290	0.0016902	SEPP1
5458	A_37_P042785	0.00169433	AFF3
5459	A_37_P419839	0.0016955	LDB3
5460	A_37_P146340	0.00169665	HMHA1
5461	A_37_P016788	0.00169884	RPL32
5462	A_37_P041783	0.00171085	CMPK2
5463	A_37_P325638	0.00171315	CCDC11

2074	A_37_P144734	3.56E-005	DDX49
2075	A_37_P183793	3.56E-005	LOC391322
2076	A_37_P195970	3.57E-005	KIAA1274
2077	A_37_P155129	3.58E-005	ARPC1A
2078	A_37_P009057	3.58E-005	CDKN2AIPNL
2079	A_37_P194145	3.58E-005	ECHDC3
2080	A_37_P409323	3.58E-005	SRRD
2081	A_37_P151868	3.60E-005	ARHGAP33
2082	A_37_P171036	3.60E-005	PTK2B
2083	A_37_P103916	3.60E-005	DHRS2
2084	A_37_P129198	3.61E-005	TMEM201
2085	A_37_P299924	3.61E-005	TBC1D25
2086	A_37_P145903	3.64E-005	GLTSCR2
2087	A_37_P413600	3.64E-005	PFDN4
2088	A_37_P134929	3.64E-005	FOXP4
2089	A_37_P190083	3.65E-005	POFUT1
2090	A_37_P170166	3.65E-005	RPL8
2091	A_37_P180965	3.65E-005	UBE2Q2
2092	A_37_P362180	3.66E-005	NT5DC1
2093	A_23_P132175	3.66E-005	RTN4R
2094	A_37_P137983	3.66E-005	MICB
2095	A_33_P3313221	3.67E-005	ZNF691
2096	A_37_P392511	3.67E-005	RECQL4
2097	A_37_P139783	3.68E-005	SLC35B3
2098	A_32_P183904	3.68E-005	SHF
2099	A_37_P382427	3.69E-005	IQCE
2100	A_33_P3355831	3.69E-005	ZNF213
2101	A_37_P157468	3.70E-005	FAM20C
2102	A_37_P317770	3.72E-005	TOR1A
2103	A_37_P261811	3.72E-005	RHBDL1
2104	A_24_P382467	3.73E-005	SLC39A3
2105	A_37_P312905	3.73E-005	TSEN54
2106	A_37_P203239	3.74E-005	ALG9
2107	A_37_P390898	3.75E-005	CSMD1
2108	A_33_P3235975	3.75E-005	CHCHD6
2109	A_37_P071613	3.76E-005	GAGE12B
2110	A_37_P151449	3.77E-005	SEPW1
2111	A_37_P253998	3.77E-005	USO1
2112	A_37_P144865	3.77E-005	NR2F6
2113	A_37_P171972	3.78E-005	FAM86B2
2114	A_23_P17430	3.78E-005	RBM38
2115	A_37_P145882	3.79E-005	GLT25D1
2116	A_37_P130211	3.79E-005	SIPA1L2
2117	A_37_P287884	3.80E-005	MED21
2118	A_37_P215122	3.80E-005	HSF2BP
2119	A_37_P133795	3.81E-005	CYB5R4
2120	A_37_P338722	3.81E-005	CTBS
2121	A_37_P253905	3.82E-005	PPP2R2C
2122	A_37_P282439	3.82E-005	KRT80
2123	A_37_P291393	3.82E-005	POC1B
2124	A_37_P181961	3.82E-005	PI4KA
2125	A_37_P049897	3.82E-005	MYEOV2
2126	A_37_P166176	3.82E-005	XKR5
2127	A_37_P436573	3.82E-005	ZDHHC13
2128	A_37_P005551	3.82E-005	C5orf62
2129	A_37_P031676	3.83E-005	CMTM4
2130	A_37_P152258	3.83E-005	PPP1R12C
2131	A_37_P007972	3.83E-005	SNX24
2132	A_37_P134049	3.85E-005	MICAL1
2133	A_23_P345674	3.85E-005	ZNF71
2134	A_37_P172529	3.86E-005	PRSS55

5464	A_37_P093864	0.00171968	JAK2
5465	A_33_P3209162	0.00172427	APOC4
5466	A_23_P161076	0.00172452	CD2
5467	A_37_P165231	0.00172799	RAPGEF5
5468	A_37_P023692	0.00172885	AGA
5469	A_37_P064828	0.0017297	NOC4L
5470	A_37_P200406	0.00172978	RUFY2
5471	A_37_P113194	0.00173107	CNTN2
5472	A_37_P111834	0.00173167	CADM3
5473	A_37_P006640	0.00173262	POC5
5474	A_37_P080372	0.00173445	NR1D1
5475	A_37_P046840	0.00173584	PLA2R1
5476	A_37_P200445	0.00173673	UTF1
5477	A_37_P368584	0.00173716	DMRTC2
5478	A_37_P199179	0.00173889	RGR
5479	A_37_P123247	0.00173968	NSUN4
5480	(+)E1A_r60_n9	0.00173981	E1A_r60_n9
5481	A_37_P007078	0.00174359	NAIP
5482	A_37_P014416	0.00174436	HRG
5483	A_33_P3345011	0.00174629	ACTL9
5484	A_33_P3301040	0.00175011	HHLA2
5485	A_37_P131589	0.00175411	AIF1
5486	A_37_P182593	0.00175526	EMID1
5487	A_37_P144578	0.00175638	CYP2F1
5488	A_37_P078726	0.00175737	KRT40
5489	A_37_P188931	0.00176029	SPATA2
5490	A_37_P199393	0.00176064	CAMK2G
5491	A_37_P090553	0.00176091	FRMD3
5492	A_37_P014917	0.00176247	ATP2B2
5493	A_37_P305328	0.00176295	GPR179
5494	A_37_P434332	0.0017652	LRP4
5495	A_37_P263030	0.00176557	TEPP
5496	A_37_P099531	0.00176566	LAMA1
5497	A_37_P096997	0.00177114	C9orf72
5498	A_37_P066748	0.00177129	SFSWAP
5499	A_37_P136230	0.00177156	HACE1
5500	A_37_P077398	0.00177677	ACBD4
5501	A_37_P330646	0.00177732	RPS29
5502	A_37_P079156	0.00178046	KRTAP4-1
5503	A_37_P211189	0.0017822	SMTNL1
5504	A_37_P165749	0.00178265	NKX2-6
5505	A_37_P005419	0.00178523	MEGF10
5506	A_33_P3237260	0.00178994	C11orf53
5507	A_37_P047367	0.00179132	MRPS5
5508	A_37_P161063	0.00179329	MKLN1
5509	A_37_P129921	0.0017934	CDCP2
5510	A_37_P038817	0.00179457	PPL
5511	A_37_P080873	0.00179619	ENPP7
5512	A_37_P141085	0.0017972	TCP10
5513	A_37_P165892	0.00179864	ATP6V0D2
5514	A_37_P207397	0.00180423	C11orf35
5515	A_37_P270350	0.00180785	IL1R2
5516	A_23_P108265	0.00181417	OR7C2
5517	A_23_P363316	0.00181607	HOXB5
5518	A_37_P022590	0.00181766	ANK2
5519	A_23_P66137	0.00181771	SOX8
5520	A_37_P287089	0.00181835	NAV3
5521	A_37_P121900	0.00181897	MOV10
5522	A_37_P211351	0.00182054	SPON1
5523	A_37_P265738	0.00182092	WDSUB1
5524	A_37_P045767	0.00182094	GLI2

2135	A_37_P098398	3.88E-005	SNAPC4
2136	A_37_P122613	3.88E-005	C1orf159
2137	A_37_P088072	3.89E-005	SPEM1
2138	A_37_P091419	3.89E-005	CEL
2139	A_37_P056955	3.89E-005	NHEJ1
2140	A_37_P211198	3.90E-005	SNX15
2141	A_37_P190595	3.90E-005	SLC12A5
2142	A_37_P162456	3.90E-005	PON2
2143	A_37_P207601	3.90E-005	DSCAML1
2144	A_37_P052048	3.90E-005	ILKAP
2145	A_37_P355054	3.91E-005	ZP4
2146	A_33_P3237874	3.93E-005	TROAP
2147	A_37_P178610	3.93E-005	NOX5
2148	A_37_P214454	3.94E-005	DOPEY2
2149	A_37_P029865	3.95E-005	TMEM128
2150	A_37_P209834	3.96E-005	PTPMT1
2151	A_37_P112217	3.96E-005	MEGF6
2152	A_37_P051358	3.96E-005	C2orf60
2153	A_37_P153535	3.97E-005	ZNF274
2154	A_23_P139912	3.98E-005	IGFBP6
2155	A_37_P148031	3.98E-005	MUC16
2156	A_37_P341278	3.98E-005	IKBKE
2157	A_24_P143076	3.99E-005	FAM109A
2158	A_33_P3359344	3.99E-005	C1orf86
2159	A_37_P091562	3.99E-005	TOMM5
2160	A_37_P007979	4.00E-005	SPATA24
2161	A_37_P178867	4.00E-005	TMC3
2162	A_37_P405062	4.00E-005	NIPSNAP1
2163	A_37_P063588	4.00E-005	DPY19L2
2164	A_37_P356076	4.01E-005	MEA1
2165	A_37_P006184	4.02E-005	PCBD2
2166	A_37_P169984	4.02E-005	HR
2167	A_37_P265832	4.02E-005	ACVR1C
2168	A_37_P160463	4.02E-005	HIP1
2169	A_37_P114724	4.02E-005	SLC6A9
2170	A_24_P136758	4.04E-005	SAMD4B
2171	A_37_P182391	4.04E-005	DGCR6
2172	A_37_P197570	4.05E-005	LRRC27
2173	A_33_P3329023	4.05E-005	FAM69A
2174	A_37_P321175	4.05E-005	FAM120AOS
2175	A_37_P160503	4.06E-005	PLXNA4
2176	A_23_P256158	4.07E-005	ADRA2C
2177	A_37_P216732	4.07E-005	CARKD
2178	A_37_P387725	4.09E-005	KCP
2179	A_33_P3262020	4.10E-005	C8G
2180	A_37_P064855	4.11E-005	C1RL
2181	A_37_P409316	4.12E-005	SREBF2
2182	A_33_P3381870	4.13E-005	EPB49
2183	A_23_P71379	4.13E-005	PSCA
2184	A_33_P3622472	4.14E-005	NAA10
2185	A_37_P173209	4.14E-005	CIB2
2186	A_37_P369500	4.14E-005	CYP2A7
2187	A_37_P149082	4.15E-005	SH3GL1
2188	A_37_P361723	4.15E-005	MRAP2
2189	A_37_P109128	4.15E-005	ACOT11
2190	A_37_P369339	4.17E-005	FLT3LG
2191	A_37_P012516	4.18E-005	GLT8D1
2192	A_37_P364496	4.19E-005	TAF11
2193	A_37_P146027	4.19E-005	GPX4
2194	A_37_P011160	4.19E-005	ADCY5
2195	A_37_P299431	4.20E-005	SLC25A43

5525	A_37_P115315	0.00182173	FAM167B
5526	A_37_P135709	0.00182452	GSTA4
5527	A_37_P011344	0.00182493	SLC4A7
5528	A_37_P127798	0.00182514	LRRIQ3
5529	A_23_P7535	0.00182658	HRH2
5530	A_37_P298878	0.00183263	RBM10
5531	A_37_P132326	0.00183373	TSPO2
5532	A_37_P186281	0.00183392	NKAIN4
5533	A_32_P203099	0.001835	TCTE1
5534	A_37_P108698	0.00183684	VASH1
5535	A_37_P113961	0.001837	DDR2
5536	A_37_P176103	0.00184612	ATP10A
5537	A_37_P211412	0.00184643	SSH3
5538	A_37_P045520	0.00184977	CYP26B1
5539	A_37_P314603	0.00185341	MED22
5540	A_37_P300655	0.00185777	WWC3
5541	A_37_P410119	0.00186338	SULF2
5542	A_37_P262589	0.00186383	LUC7L
5543	A_37_P079915	0.00186516	ALOX12B
5544	A_37_P213008	0.0018663	ZP1
5545	A_33_P3216458	0.00186791	ADAM23
5546	A_32_P114215	0.00186921	COMMD6
5547	A_23_P142345	0.0018711	PRTN3
5548	A_37_P126053	0.0018718	SYT6
5549	A_37_P362608	0.00187506	PIM1
5550	A_37_P248917	0.00188039	GPR78
5551	A_37_P098034	0.00188083	CCL19
5552	A_37_P188003	0.00188112	APCDD1L
5553	A_33_P3369146	0.00188583	ATP2B3
5554	A_37_P320204	0.00188711	PPAPDC3
5555	A_37_P188347	0.00189203	PLAGL2
5556	A_37_P410527	0.00189819	C20orf186
5557	A_37_P313721	0.00190025	ZZEF1
5558	A_37_P024823	0.00190631	CXCL9
5559	A_24_P324520	0.00190934	MTCP1
5560	A_37_P072368	0.0019097	ITGB1BP2
5561	A_37_P040491	0.00191071	ZSCAN10
5562	A_37_P062345	0.00191343	KIF5A
5563	A_37_P055620	0.00191448	NEB
5564	A_33_P3272479	0.00191472	NPM2
5565	A_37_P032020	0.00191483	C16orf55
5566	A_37_P112208	0.00192049	MEF2D
5567	A_37_P278190	0.00192231	SP140
5568	A_37_P129463	0.00193592	TOE1
5569	A_23_P99076	0.00193739	PRH2
5570	A_37_P033730	0.00193966	LMF1
5571	A_37_P078630	0.001941	KRT31
5572	A_37_P048010	0.00194353	SNTG2
5573	A_37_P088970	0.00195099	TMEM92
5574	A_23_P500886	0.00195292	CLDN15
5575	A_37_P309594	0.00195403	NGFR
5576	A_37_P186179	0.00195452	CHADL
5577	A_37_P103142	0.00195598	C14orf178
5578	A_37_P127479	0.00195668	FAM132A
5579	A_37_P149288	0.00195705	NCCRP1
5580	A_37_P125993	0.00195952	TMEM63A
5581	A_37_P223779	0.00196118	ABLIM3
5582	A_23_P397120	0.00196274	C19orf77
5583	A_37_P033836	0.00196565	PRSS22
5584	A_37_P032741	0.00196639	CIITA
5585	A_37_P439236	0.00196821	PDE9A

2196	A_37_P185977	4.22E-005	CECR5
2197	A_37_P237969	4.23E-005	HDAC11
2198	A_23_P401084	4.23E-005	ZNF575
2199	A_37_P397889	4.23E-005	CCNB2
2200	A_37_P036267	4.23E-005	LYRM1
2201	A_37_P039094	4.27E-005	FAM86A
2202	A_37_P153110	4.27E-005	CDKN2D
2203	A_37_P442212	4.28E-005	C13orf27
2204	A_37_P249407	4.30E-005	IL15
2205	A_37_P017876	4.30E-005	EIF5A2
2206	A_37_P034176	4.31E-005	GINS3
2207	A_37_P181628	4.31E-005	CLTCL1
2208	A_37_P393402	4.32E-005	TM2D2
2209	A_37_P067634	4.32E-005	CCDC92
2210	A_37_P056552	4.32E-005	PECR
2211	A_37_P047083	4.32E-005	KCMF1
2212	A_37_P256164	4.33E-005	TEKT5
2213	A_23_P61551	4.33E-005	CD2BP2
2214	A_37_P410910	4.34E-005	AHCY
2215	A_37_P417830	4.35E-005	DPYSL4
2216	A_37_P146393	4.36E-005	HPN
2217	A_37_P353114	4.36E-005	TNN
2218	A_37_P159947	4.37E-005	BRAF
2219	A_37_P093929	4.37E-005	KCNT1
2220	A_37_P036443	4.37E-005	METRN
2221	A_37_P320266	4.37E-005	INPP5E
2222	A_37_P162950	4.37E-005	SDK1
2223	A_37_P418294	4.38E-005	FAM45A
2224	A_37_P197217	4.38E-005	PYROXD2
2225	A_37_P210386	4.38E-005	RPLP2
2226	A_37_P365050	4.39E-005	KIAA0319
2227	A_37_P354161	4.40E-005	FHL3
2228	A_23_P29153	4.40E-005	RTDR1
2229	A_37_P406809	4.43E-005	GNAZ
2230	A_37_P249146	4.43E-005	PLA2G12A
2231	A_37_P303193	4.44E-005	CD68
2232	A_37_P047223	4.44E-005	HADHA
2233	A_37_P199218	4.44E-005	PCBD1
2234	A_37_P075533	4.45E-005	FAM104B
2235	A_23_P20035	4.45E-005	GPR146
2236	A_24_P51909	4.46E-005	CPLX1
2237	A_37_P157131	4.46E-005	EEPD1
2238	A_37_P284901	4.46E-005	GATC
2239	A_37_P274818	4.47E-005	OSBPL6
2240	A_37_P272900	4.47E-005	RETSAT
2241	A_37_P208045	4.49E-005	OSBPL5
2242	A_37_P108886	4.49E-005	ZNF410
2243	A_23_P371145	4.50E-005	ADPRHL1
2244	A_37_P314747	4.50E-005	C9orf43
2245	A_37_P033998	4.50E-005	FLYWCH2
2246	A_37_P131780	4.50E-005	APOM
2247	A_33_P3254460	4.51E-005	DLK2
2248	A_37_P163892	4.51E-005	KCNH2
2249	A_37_P113451	4.52E-005	CREB3L4
2250	A_37_P116612	4.52E-005	NUDT17
2251	A_37_P109373	4.52E-005	Sep-15
2252	A_37_P435096	4.53E-005	PDDC1
2253	A_37_P381505	4.54E-005	WBSCR27
2254	A_37_P195763	4.55E-005	SFRP5
2255	A_37_P203094	4.56E-005	CORO1B
2256	A_37_P090663	4.56E-005	PHF19

5586	A_37_P080650	0.00197039	DPH1
5587	A_37_P238816	0.00197154	SCN11A
5588	A_37_P181547	0.00197663	APOBEC3H
5589	A_23_P104509	0.00198846	FAM53B
5590	A_23_P29282	0.0019899	NPTXR
5591	A_37_P331396	0.00199303	FAM161B
5592	A_37_P157149	0.00199409	EGFR
5593	A_37_P362896	0.00199679	C6orf118
5594	A_23_P343826	0.00199733	FCHSD1
5595	A_37_P292845	0.00199803	ACRC
5596	A_37_P263967	0.00199814	CES5A
5597	A_37_P076540	0.0019985	RIPPLY1
5598	A_37_P280742	0.00200279	ZRANB3
5599	A_37_P354314	0.00200353	WNT2B
5600	A_37_P179904	0.00200568	SLC24A5
5601	A_37_P031995	0.00200903	C16orf11
5602	A_37_P116939	0.00201415	DCST2
5603	A_37_P012066	0.00201532	ALG3
5604	A_23_P42909	0.00201606	TMEM139
5605	A_33_P3313801	0.00201733	CCDC34
5606	A_37_P037558	0.00202542	PLCG2
5607	A_37_P197764	0.00202978	HSPA12A
5608	A_37_P062608	0.00204097	MSI1
5609	A_37_P252542	0.00204273	SEC24D
5610	A_37_P017478	0.00204298	MYRIP
5611	A_37_P123294	0.00204356	NTRK1
5612	A_37_P056721	0.00204367	CERKL
5613	A_37_P264149	0.00204506	ZNF785
5614	A_37_P226465	0.00204712	ARHGEF37
5615	A_37_P379922	0.00205246	CFTR
5616	A_37_P284083	0.00205315	LIN7A
5617	A_37_P205977	0.0020562	KCNK4
5618	A_37_P145203	0.00205974	EMR1
5619	A_37_P101079	0.00206489	RPL17-C18ORF32
5620	A_37_P014836	0.00206705	ITIH3
5621	A_37_P081405	0.0020675	TMEM106A
5622	A_37_P180764	0.00207107	CDAN1
5623	A_37_P430877	0.0020721	MUC6
5624	A_37_P436529	0.00207555	TRIM5
5625	A_37_P166431	0.00207603	ST18
5626	A_37_P298757	0.0020827	RPS4X
5627	A_37_P144424	0.00208455	CRB3
5628	A_37_P147013	0.00208749	TM6SF2
5629	A_37_P035868	0.00209524	SLX4
5630	A_37_P068863	0.00209557	ZNF140
5631	A_37_P135106	0.00210035	MOCS1
5632	A_37_P054758	0.00210139	LYG1
5633	A_37_P243276	0.00211685	SI
5634	A_24_P227927	0.00211718	IL21R
5635	A_37_P021610	0.00211952	CDHR4
5636	A_37_P143977	0.00212099	CEACAM16
5637	A_37_P033988	0.00212935	FLYWCH1
5638	A_37_P070112	0.00213515	CCDC22
5639	A_37_P087788	0.00213516	RHBDF2
5640	A_37_P091008	0.00213566	C9orf170
5641	A_37_P124549	0.00213971	PLK3
5642	A_37_P132668	0.0021402	GABRR1
5643	A_37_P299526	0.00214153	PCYT1B
5644	A_37_P187451	0.0021453	GGT7
5645	A_37_P123518	0.00214556	OPRD1
5646	A_37_P042389	0.00214669	C2orf62

2257	A_37_P201765	4.58E-005	ATG16L2
2258	A_37_P043082	4.59E-005	CHCHD5
2259	A_37_P132146	4.59E-005	BPHL
2260	A_37_P091057	4.59E-005	C9orf6
2261	A_37_P101411	4.60E-005	PARD6G
2262	A_37_P096306	4.61E-005	SLC2A6
2263	A_37_P184284	4.61E-005	MPST
2264	A_37_P152500	4.61E-005	FBL
2265	A_37_P184776	4.62E-005	C22orf39
2266	A_37_P053981	4.64E-005	FAM176A
2267	A_37_P067040	4.64E-005	SLC48A1
2268	A_37_P414901	4.64E-005	TRIB3
2269	A_37_P237804	4.64E-005	DALRD3
2270	A_37_P132959	4.65E-005	MSH5
2271	A_37_P362851	4.65E-005	RPS6KA2
2272	A_37_P301391	4.66E-005	RNF157
2273	A_33_P3299510	4.66E-005	SCXA
2274	A_37_P432141	4.66E-005	EFEMP2
2275	A_37_P294198	4.67E-005	CSAG1
2276	A_37_P428797	4.67E-005	ARRB1
2277	A_37_P062013	4.68E-005	TSPAN8
2278	A_37_P139534	4.68E-005	PSMB9
2279	A_37_P288220	4.69E-005	MYL6B
2280	A_37_P389708	4.69E-005	CNOT7
2281	A_37_P259884	4.69E-005	LOC81691
2282	A_37_P162146	4.69E-005	PARP12
2283	A_37_P153760	4.70E-005	MATK
2284	A_37_P037932	4.71E-005	NAGPA
2285	A_37_P233720	4.73E-005	CLDND1
2286	A_37_P197534	4.73E-005	DRGX
2287	A_37_P032029	4.73E-005	C16orf59
2288	A_37_P414620	4.74E-005	FAM65C
2289	A_37_P232039	4.74E-005	TGFBI
2290	A_37_P020256	4.75E-005	SLC6A1
2291	A_37_P173392	4.77E-005	NMB
2292	A_37_P219172	4.77E-005	POLR1D
2293	A_37_P155139	4.79E-005	ARPC1B
2294	A_33_P3291176	4.79E-005	VMAC
2295	A_37_P188569	4.80E-005	LBP
2296	A_23_P155890	4.80E-005	NAA11
2297	A_37_P107913	4.81E-005	RABGGTA
2298	A_37_P176256	4.81E-005	ENST00000490848
2299	A_37_P284934	4.81E-005	GDF11
2300	A_37_P063006	4.82E-005	BEST3
2301	A_37_P172975	4.83E-005	ZNF517
2302	A_37_P176787	4.83E-005	HDDC3
2303	A_37_P032688	4.83E-005	TMEM170A
2304	A_37_P313030	4.83E-005	TTYH2
2305	A_37_P141920	4.83E-005	NEU1
2306	A_33_P3303577	4.84E-005	TMEM110
2307	A_33_P3248982	4.85E-005	FAIM2
2308	A_37_P128246	4.85E-005	THEM4
2309	A_37_P306684	4.85E-005	DHRS13
2310	A_33_P3294654	4.86E-005	C1orf89
2311	A_37_P178127	4.86E-005	RPL4
2312	A_37_P419517	4.86E-005	JAKMIP3
2313	A_23_P212400	4.86E-005	NAT6
2314	A_37_P435019	4.88E-005	LTBP3
2315	A_37_P027441	4.88E-005	MTHFD2L
2316	A_37_P067318	4.89E-005	NCOR2
2317	A_37_P215236	4.89E-005	NDUFV3

5647	A_37_P092315	0.00214992	LHX6
5648	A_37_P020177	0.00215042	LRRFIP2
5649	A_37_P428835	0.002156	ART5
5650	A_37_P376116	0.00215709	PPP1R14A
5651	A_37_P041231	0.00215719	ALS2CR8
5652	A_37_P013838	0.00216094	PRSS50
5653	A_37_P249492	0.00216376	PLAC8
5654	A_37_P133013	0.00216638	C6orf70
5655	A_37_P305292	0.00218902	G6PC3
5656	A_37_P265981	0.00220255	C2orf27A
5657	A_37_P033181	0.00220363	CX3CL1
5658	A_37_P186751	0.00220444	C20orf114
5659	A_37_P173142	0.0022109	KIF7
5660	A_37_P144487	0.0022226	ZNF565
5661	A_23_P71480	0.00222331	DEFB1
5662	A_37_P146931	0.00222517	KIR2DL3
5663	A_37_P151927	0.0022287	SPINT2
5664	A_37_P425423	0.00223197	ABTB2
5665	A_24_P76675	0.0022347	MFAP3L
5666	A_37_P336101	0.00224102	CDC14A
5667	A_37_P128501	0.00224272	CELF3
5668	A_37_P186295	0.00224972	SLA2
5669	A_37_P308369	0.00224983	HOXB13
5670	A_37_P167037	0.00225129	DENND3
5671	A_23_P4696	0.00225832	PPAN-P2RY11
5672	A_37_P151072	0.00227436	NAPSA
5673	A_33_P3312743	0.00227565	KRTAP4-3
5674	A_37_P053636	0.00227758	A_37_P053636
5675	A_37_P152032	0.00228171	RHPN2
5676	A_37_P259944	0.0022841	LRRC36
5677	A_37_P269614	0.00228631	GPC1
5678	A_37_P108249	0.00229316	TECPR2
5679	A_23_P336678	0.00229845	GPHB5
5680	A_37_P042194	0.00229911	C2orf15
5681	A_23_P92672	0.00230218	OCLN
5682	A_37_P023039	0.00230461	RASSF6
5683	A_37_P130916	0.00230709	KIF17
5684	A_37_P137167	0.00230978	REPS1
5685	A_37_P116700	0.00231671	SPAG17
5686	A_37_P197438	0.00231913	C10orf93
5687	A_24_P271049	0.00231953	C18orf32
5688	A_37_P084993	0.00231978	MINK1
5689	A_24_P162979	0.00231981	ANKRD53
5690	A_33_P3329444	0.00232156	MAMSTR
5691	A_37_P125953	0.00232307	TNR
5692	A_37_P307427	0.00232436	SLFN12
5693	A_37_P140015	0.00232516	RPL10A
5694	A_37_P005877	0.00233105	TRPC7
5695	A_37_P236649	0.00234365	DZIP3
5696	A_37_P182924	0.00235291	TTLL8
5697	A_37_P192252	0.00235412	FRMD4A
5698	A_37_P182748	0.00235554	MLC1
5699	A_37_P139576	0.00235995	LPA
5700	A_24_P316257	0.00237349	NHLRC4
5701	A_37_P040756	0.00237802	ACMSD
5702	A_24_P399083	0.00237977	TXNL4B
5703	A_37_P191441	0.00238438	WFDC10A
5704	A_37_P219595	0.0023858	RAB20
5705	A_23_P130974	0.00238588	KIAA1683
5706	A_37_P041532	0.00238816	ARID5A
5707	A_37_P282392	0.0023897	KRT76

2318	A_37_P161610	4.89E-005	POLM
2319	A_37_P083239	4.90E-005	YBX2
2320	A_37_P395457	4.91E-005	LSM1
2321	A_37_P126509	4.92E-005	TMED5
2322	A_37_P314832	4.93E-005	C9orf91
2323	A_37_P103921	4.93E-005	DHRS4
2324	A_37_P254590	4.94E-005	ABAT
2325	A_33_P3319886	4.96E-005	C19orf45
2326	A_37_P218147	4.96E-005	USP12
2327	A_33_P3295690	4.96E-005	C16orf90
2328	A_37_P171286	4.97E-005	RBPMS
2329	A_37_P171380	4.97E-005	RHPN1
2330	A_37_P114828	4.98E-005	EFNA3
2331	A_37_P129113	4.98E-005	TINAGL1
2332	A_37_P376329	4.99E-005	TMEM59L
2333	A_37_P206913	5.00E-005	ODZ4
2334	A_37_P005574	5.00E-005	MTRR
2335	A_37_P097006	5.00E-005	TPRN
2336	A_37_P131805	5.00E-005	FRMD1
2337	A_37_P139331	5.00E-005	C6orf168
2338	A_37_P362919	5.01E-005	C6orf130
2339	A_37_P120877	5.01E-005	TM2D1
2340	A_37_P132983	5.01E-005	C6orf26
2341	A_37_P128803	5.02E-005	LYST
2342	A_37_P087569	5.02E-005	SERPINF2
2343	A_37_P071318	5.03E-005	GPM6B
2344	A_37_P118640	5.04E-005	LAMC2
2345	A_37_P163404	5.05E-005	JAZF1
2346	A_37_P100342	5.05E-005	KIAA0427
2347	A_37_P020909	5.05E-005	TDGF1
2348	A_37_P179923	5.07E-005	PCSK6
2349	A_37_P082476	5.07E-005	KDM6B
2350	A_37_P105978	5.08E-005	C14orf149
2351	A_37_P054272	5.08E-005	SMYD5
2352	A_33_P3300308	5.08E-005	MAP1LC3A
2353	A_37_P031685	5.08E-005	ATXN2L
2354	A_37_P237793	5.08E-005	GPX1
2355	A_37_P292043	5.08E-005	TSPAN9
2356	A_37_P005668	5.10E-005	NDST1
2357	A_37_P034350	5.10E-005	GPR97
2358	A_37_P033782	5.12E-005	TK2
2359	A_37_P071596	5.12E-005	FUNDC2
2360	A_37_P058887	5.12E-005	CACNA1C
2361	A_37_P076859	5.13E-005	WAS
2362	A_37_P149508	5.14E-005	TNNT1
2363	A_37_P065608	5.15E-005	PLEKHG6
2364	A_37_P434562	5.17E-005	SLC17A6
2365	A_37_P101026	5.17E-005	MOCOS
2366	A_37_P317780	5.20E-005	TOR2A
2367	A_37_P127928	5.22E-005	RHCE
2368	A_37_P032623	5.22E-005	NOXO1
2369	A_37_P046715	5.23E-005	IMP4
2370	A_37_P020125	5.23E-005	SLC22A13
2371	A_37_P366243	5.23E-005	AMH
2372	A_37_P127111	5.24E-005	SCNN1D
2373	A_37_P266132	5.25E-005	C2orf82
2374	A_37_P407944	5.26E-005	MMP11
2375	A_37_P224255	5.27E-005	ATG10
2376	A_37_P252495	5.27E-005	S100P
2377	A_23_P367899	5.27E-005	EPOR
2378	A_37_P057573	5.28E-005	ANKRD13A

5708	A_37_P204849	0.00239094	FRMD8
5709	A_37_P093240	0.00239278	GPR107
5710	A_37_P008121	0.00239287	STK32A
5711	A_37_P057983	0.0023956	ATN1
5712	A_37_P057371	0.00239804	CIT
5713	A_37_P024938	0.00240783	GPR125
5714	A_23_P166400	0.00241847	RASL10A
5715	A_37_P209085	0.00242562	RPS13
5716	A_37_P134419	0.00243314	CTGF
5717	A_37_P283078	0.00243663	CLEC2D
5718	A_37_P432800	0.00244701	OVOL1
5719	A_37_P393210	0.00245333	OPLAH
5720	A_37_P066816	0.00245645	ITPR2
5721	A_37_P278882	0.00246136	SRBD1
5722	A_37_P327878	0.00247586	AP1G2
5723	A_37_P245926	0.00248019	PFKFB4
5724	A_37_P048452	0.00248019	ENST00000398800
5725	A_37_P147182	0.0024903	LGALS13
5726	A_37_P145429	0.00249247	SLC7A10
5727	A_37_P001677	0.00249346	CRHBP
5728	A_37_P424088	0.0024951	TSPAN15
5729	A_37_P153572	0.00249554	ZNF304
5730	A_37_P255188	0.00249842	A_37_P255188
5731	A_37_P161535	0.00250059	PTPRN2
5732	A_37_P119828	0.00250647	BTBD19
5733	A_37_P113641	0.00251109	CTSE
5734	A_37_P154189	0.00251445	ZNF77
5735	A_32_P191860	0.00251445	SCGBL
5736	A_37_P147167	0.00252143	LENG8
5737	A_37_P006853	0.00252259	PSD2
5738	A_23_P137896	0.00252903	OXCT2
5739	A_37_P281852	0.00253367	ACRBP
5740	A_37_P279993	0.00254027	VAMP5
5741	A_37_P282205	0.00254182	ADAMTS20
5742	A_37_P032581	0.00254213	CENPN
5743	A_37_P016680	0.00254653	TNK2
5744	A_37_P021704	0.00255352	KIAA1524
5745	A_37_P253073	0.00255453	SNX25
5746	A_37_P200914	0.00255952	RAPSN
5747	A_37_P150741	0.00256094	PVRL2
5748	A_37_P020983	0.00256121	TGFBR2
5749	A_37_P295340	0.0025633	GAGE12F
5750	A_37_P054856	0.00256877	STK11IP
5751	A_37_P158007	0.0025698	PKD1L1
5752	A_23_P84359	0.00257057	SSX5
5753	A_37_P280217	0.00257789	WDR69
5754	A_23_P351138	0.00257805	CLDN9
5755	A_23_P22660	0.00258051	CYSLTR1
5756	A_37_P122664	0.00258142	NP1250934
5757	A_37_P106117	0.00258191	C14orf49
5758	A_37_P129125	0.00258259	CD160
5759	A_37_P154917	0.00258512	AMZ1
5760	A_37_P243024	0.00259074	RHO
5761	A_37_P002687	0.00259273	ARAP3
5762	A_37_P064898	0.00259324	CELA1
5763	A_37_P079476	0.00260003	CD300LG
5764	A_37_P002233	0.00260003	EIF4E1B
5765	A_37_P334094	0.00260679	ATP1A2
5766	A_37_P233135	0.00261416	SEMA6A
5767	A_37_P287531	0.00261534	C12orf51
5768	A_37_P053295	0.0026155	RPS7

2379	A_33_P3241269	5.28E-005	CES1
2380	A_37_P329494	5.28E-005	LRRC16B
2381	A_24_P216681	5.29E-005	LSM10
2382	A_37_P153441	5.30E-005	SIX5
2383	A_37_P405981	5.30E-005	CPT1B
2384	A_37_P080027	5.30E-005	GHDC
2385	A_37_P085804	5.31E-005	NEK8
2386	A_37_P062096	5.31E-005	KCNH3
2387	A_37_P265334	5.32E-005	ASAP2
2388	A_37_P145652	5.33E-005	FSD1
2389	A_37_P151160	5.33E-005	RPS28
2390	A_37_P134565	5.34E-005	FAM26F
2391	A_37_P101842	5.36E-005	SLMO1
2392	A_37_P186270	5.36E-005	CHKB
2393	A_37_P168185	5.36E-005	ASAH1
2394	A_37_P299007	5.37E-005	IQSEC2
2395	A_37_P096153	5.37E-005	PDCD1LG2
2396	A_37_P013412	5.38E-005	TKT
2397	A_37_P113012	5.38E-005	CLCN6
2398	A_37_P024170	5.38E-005	CRMP1
2399	A_37_P308739	5.39E-005	MLLT6
2400	A_37_P414963	5.39E-005	TAF4
2401	A_37_P303931	5.39E-005	02/08/12
2402	A_37_P217603	5.40E-005	RCBTB1
2403	A_37_P155392	5.41E-005	BRI3
2404	A_37_P240539	5.41E-005	PLXND1
2405	A_37_P013073	5.41E-005	EIF2B5
2406	A_37_P195858	5.41E-005	KAZALD1
2407	A_37_P037882	5.42E-005	ESRP2
2408	A_37_P068673	5.43E-005	FKBP11
2409	A_37_P210267	5.44E-005	TRPM5
2410	A_33_P3232011	5.45E-005	RAB17
2411	A_37_P305005	5.45E-005	CXCL16
2412	A_37_P218519	5.46E-005	MZT1
2413	A_37_P311107	5.47E-005	SARM1
2414	A_37_P122958	5.47E-005	C1orf59
2415	A_37_P063614	5.51E-005	C12orf11
2416	A_23_P15542	5.52E-005	HSD17B1
2417	A_37_P105596	5.52E-005	BRF1
2418	A_37_P150969	5.54E-005	PBX4
2419	A_37_P000763	5.54E-005	BTF3
2420	A_37_P145505	5.54E-005	FCGRT
2421	A_37_P392605	5.55E-005	PLEC
2422	A_37_P127353	5.56E-005	SETDB1
2423	A_37_P211759	5.56E-005	VWCE
2424	A_37_P214357	5.57E-005	PIGP
2425	A_37_P389743	5.59E-005	BMP1
2426	A_37_P183503	5.60E-005	KREMEN1
2427	A_37_P084240	5.61E-005	LRRC45
2428	A_37_P096799	5.61E-005	C9orf140
2429	A_37_P077274	5.62E-005	ABCC3
2430	A_37_P113182	5.62E-005	PI4KB
2431	A_37_P255070	5.62E-005	ABCC12
2432	A_37_P042563	5.63E-005	SUMO1
2433	A_37_P189700	5.63E-005	C20orf96
2434	A_37_P291844	5.64E-005	KDM2B
2435	A_37_P014809	5.64E-005	ITIH1
2436	A_37_P398645	5.64E-005	CSPG4
2437	A_33_P3303212	5.64E-005	CCDC74B
2438	A_37_P260441	5.64E-005	MT1A
2439	A_37_P101189	5.65E-005	NEDD4L

5769	A_37_P060590	0.00261696	VDR
5770	A_37_P117084	0.00261846	HIPK1
5771	A_37_P355342	0.00261848	AKAP7
5772	A_37_P213844	0.00262149	PRDM15
5773	A_37_P189568	0.00262772	C20orf151
5774	A_37_P076051	0.00263232	STS
5775	A_33_P3294821	0.00263389	OTOP1
5776	A_37_P198173	0.00264622	TTC18
5777	A_37_P086999	0.00265001	RGS9
5778	A_23_P55127	0.00265342	C17orf48
5779	A_33_P3314643	0.00265376	SPEF1
5780	A_37_P340163	0.00265462	DAB1
5781	A_37_P092405	0.00265916	GNAQ
5782	A_33_P3339865	0.00266422	CALML5
5783	A_33_P3334102	0.0026828	SH3D20
5784	A_37_P210990	0.00268811	CASP4
5785	A_37_P202111	0.00269017	C11orf1
5786	A_37_P174753	0.00270414	DLL4
5787	A_37_P169193	0.00270518	SPATC1
5788	A_37_P175569	0.0027053	GANC
5789	A_37_P394278	0.00270759	PGCP
5790	A_37_P028732	0.00270955	RPS3A
5791	A_37_P102072	0.00271505	RTTN
5792	A_37_P224798	0.00271752	C7
5793	A_37_P032083	0.00271786	C16orf70
5794	A_37_P180625	0.0027182	FBN1
5795	A_37_P052289	0.00272223	PRKCE
5796	A_37_P043351	0.00272565	COL4A3
5797	A_37_P037311	0.00273231	OTOA
5798	A_37_P297636	0.00274141	MED12
5799	A_37_P171895	0.00274168	LY6H
5800	A_37_P210816	0.00274473	CARS
5801	A_23_P368711	0.00274884	LILRB3
5802	A_37_P180380	0.00275031	LYSMD4
5803	A_37_P320216	0.00275051	PPP2R4
5804	A_37_P117640	0.00276037	IL6R
5805	A_37_P095806	0.00276037	SLC28A3
5806	A_37_P043574	0.00276702	CPSF3
5807	A_37_P145590	0.0027784	PAPL
5808	A_33_P3392250	0.00277915	FAM178A
5809	A_37_P194706	0.0027839	CUEDC2
5810	A_37_P142785	0.00278398	APLP1
5811	A_37_P213890	0.00279384	ENST00000468282
5812	A_23_P79134	0.00279417	PLEKHF1
5813	A_37_P189085	0.00279421	LPIN3
5814	A_33_P3385993	0.00280198	TTLL9
5815	A_37_P016174	0.00280806	HEG1
5816	A_37_P217156	0.00281589	LHFP
5817	A_37_P020743	0.00282056	STXBP5L
5818	A_37_P130171	0.00282552	UROD
5819	A_37_P313536	0.00284688	Mar-10
5820	A_37_P018425	0.00286486	IL17RD
5821	A_37_P328608	0.0028691	KIAA0284
5822	A_37_P266112	0.00287384	C2orf72
5823	A_33_P3412900	0.00287607	CBLN3
5824	A_37_P252943	0.00287727	FAM184B
5825	A_23_P16110	0.00287808	OR7E24
5826	A_24_P220472	0.00288598	SPATA12
5827	A_37_P117874	0.00288773	GUCA2A
5828	A_37_P167699	0.0028907	FAM83A
5829	A_37_P058244	0.00289961	C12orf26

2440	A_37_P117036	5.66E-005	HHLA3
2441	A_37_P180579	5.68E-005	TM6SF1
2442	A_37_P154038	5.68E-005	ZNF844
2443	A_37_P211789	5.68E-005	TCIRG1
2444	A_37_P227605	5.69E-005	KIAA0141
2445	A_37_P084049	5.69E-005	TMEM220
2446	A_37_P298649	5.69E-005	PLXNB3
2447	A_23_P47924	5.69E-005	PTPRR
2448	A_37_P143751	5.72E-005	CCDC130
2449	A_37_P036514	5.75E-005	C16orf58
2450	A_37_P195101	5.76E-005	PRTFDC1
2451	A_33_P3303015	5.76E-005	RPS9
2452	A_37_P085198	5.77E-005	C17orf58
2453	A_37_P150077	5.78E-005	IL11
2454	A_37_P143764	5.79E-005	CCDC155
2455	A_37_P100641	5.80E-005	BCL2
2456	A_23_P106798	5.80E-005	SIAH1
2457	A_37_P351180	5.81E-005	FAM131C
2458	A_37_P057916	5.82E-005	ARNTL2
2459	A_37_P398094	5.84E-005	MEX3B
2460	A_37_P193706	5.87E-005	CYP26A1
2461	A_37_P035363	5.88E-005	SYCE1L
2462	A_37_P083403	5.89E-005	ULK2
2463	A_37_P097324	5.89E-005	SLC35D2
2464	A_33_P3263666	5.89E-005	ANKRD9
2465	A_37_P336795	5.89E-005	AKR7L
2466	A_37_P087599	5.90E-005	SGCA
2467	A_37_P017607	5.91E-005	MYLK
2468	A_37_P209162	5.91E-005	IGF2
2469	A_37_P032104	5.91E-005	C16orf75
2470	A_37_P258158	5.91E-005	HBQ1
2471	A_37_P070000	5.92E-005	CA5B
2472	A_37_P008349	5.92E-005	CCNO
2473	A_37_P071629	5.93E-005	GAGE13
2474	A_37_P058436	5.94E-005	C12orf75
2475	A_37_P157324	5.94E-005	EPHB6
2476	A_37_P151134	5.96E-005	RPL36
2477	A_37_P209191	5.97E-005	PAK1
2478	A_37_P095100	5.97E-005	EXD3
2479	A_23_P135248	5.99E-005	CCL27
2480	A_37_P079641	6.00E-005	COPZ2
2481	A_37_P348138	6.00E-005	EPHA2
2482	A_37_P421378	6.00E-005	Mar-05
2483	A_37_P368994	6.00E-005	ETV2
2484	A_37_P268552	6.00E-005	EPAS1
2485	A_37_P063480	6.00E-005	SCNN1A
2486	A_37_P367596	6.02E-005	CD33
2487	A_37_P097290	6.02E-005	LPPR1
2488	A_37_P326729	6.03E-005	MDP1
2489	A_37_P140576	6.03E-005	PPIL6
2490	A_23_P64990	6.03E-005	RAD52
2491	A_37_P052325	6.05E-005	PROC
2492	A_37_P066205	6.06E-005	RBMS2
2493	A_37_P413540	6.06E-005	PCIF1
2494	A_37_P021128	6.06E-005	TMEM42
2495	A_37_P410418	6.08E-005	C20orf103
2496	A_37_P314662	6.08E-005	C9orf119
2497	A_37_P210104	6.08E-005	RELT
2498	A_37_P272970	6.08E-005	DPP4
2499	A_37_P041336	6.09E-005	ANO7
2500	A_37_P083272	6.09E-005	WNT3

5830	A_24_P201739	0.00290049	SH2B3
5831	A_23_P53126	0.0029016	LMO2
5832	A_37_P078827	0.00290216	C17orf64
5833	A_37_P182516	0.00291689	GNB1L
5834	A_37_P120201	0.00292092	SPATA21
5835	A_37_P156449	0.00292153	MLL3
5836	A_37_P158175	0.00292381	CYTH3
5837	A_37_P022977	0.002925	ACCN5
5838	A_23_P397308	0.00292987	KLC4
5839	A_33_P3336384	0.00293044	PAOX
5840	A_37_P011490	0.00293415	CD200
5841	A_37_P109208	0.00294208	PGLYRP3
5842	A_37_P130423	0.00294682	FGR
5843	A_37_P193847	0.00294834	ANK3
5844	A_33_P3308764	0.00295423	RXRG
5845	A_37_P038637	0.00295624	SLC5A2
5846	A_37_P139043	0.00296639	POLR1C
5847	A_37_P377875	0.00296668	ZNF682
5848	A_37_P059890	0.00297042	CSAD
5849	A_37_P353326	0.00297273	FBXO2
5850	A_33_P3247392	0.00297305	TPTE
5851	A_37_P006549	0.00298146	A_37_P006549
5852	A_37_P206687	0.00299049	UBE2L6
5853	A_37_P379370	0.00299295	C7orf34
5854	A_37_P137554	0.00299368	HMGCLL1
5855	A_37_P343150	0.00299714	HDGF
5856	A_23_P143734	0.00299989	CYP2D6
5857	A_37_P424720	0.00300673	ACCSL
5858	A_37_P405514	0.00301047	CABIN1
5859	A_37_P077335	0.00301501	ABCA10
5860	A_37_P044200	0.00301784	DIS3L2
5861	A_37_P142676	0.00304255	CLEC4G
5862	A_37_P060789	0.00305733	ESYT1
5863	A_37_P409882	0.0030641	PES1
5864	A_37_P370994	0.00307495	ZFP14
5865	A_37_P199210	0.00308779	LRIT1
5866	A_37_P094400	0.00308899	OBP2B
5867	A_37_P273477	0.00309352	METAP1D
5868	A_37_P305924	0.00309777	PRR15L
5869	A_37_P238757	0.00310296	KCNH8
5870	A_37_P132245	0.00310393	BRPF3
5871	A_37_P187787	0.00311153	DSTN
5872	A_37_P189866	0.00311172	PTPRT
5873	A_37_P274844	0.00311552	SH3YL1
5874	A_37_P061624	0.00312307	HIP1R
5875	A_37_P309995	0.00312627	MYO1D
5876	A_37_P116204	0.00313063	ARID4B
5877	A_37_P216814	0.00313896	SLC15A1
5878	A_37_P070426	0.00314075	PIGA
5879	A_37_P016903	0.00314703	MAP3K13
5880	A_37_P073907	0.00315126	RPL39
5881	A_37_P012280	0.00315327	GHRL
5882	A_37_P111511	0.00316138	C1orf94
5883	A_37_P373189	0.00316464	NLRP5
5884	A_24_P118376	0.00316603	CEACAM20
5885	A_37_P092332	0.00316631	DNAI1
5886	A_23_P89601	0.00316672	KRT32
5887	A_37_P177869	0.0031768	LOXL1
5888	A_37_P288433	0.00317859	A2M
5889	A_37_P040779	0.00318589	ACOXL
5890	A_37_P363804	0.00318947	RSPH9

2501	A_37_P149994	6.09E-005	POLR2I
2502	A_37_P040140	6.09E-005	WDR90
2503	A_37_P210291	6.09E-005	ROM1
2504	A_37_P124328	6.10E-005	EPHA10
2505	A_37_P391347	6.10E-005	SYBU
2506	A_37_P144863	6.10E-005	DLL3
2507	A_37_P270909	6.11E-005	TTN
2508	A_37_P151816	6.11E-005	RABAC1
2509	A_37_P055918	6.12E-005	TTC7A
2510	A_37_P199248	6.13E-005	RGS10
2511	A_23_P101829	6.15E-005	LPAR2
2512	A_37_P088868	6.17E-005	FBF1
2513	A_33_P3286293	6.17E-005	CTNNBIP1
2514	A_37_P372204	6.19E-005	MADCAM1
2515	A_37_P058226	6.19E-005	C12orf10
2516	A_37_P373945	6.19E-005	PNPLA6
2517	A_37_P152243	6.19E-005	PDE4C
2518	A_37_P261657	6.20E-005	PYCARD
2519	A_23_P315892	6.20E-005	ST6GALNAC6
2520	A_37_P263094	6.20E-005	TRADD
2521	A_37_P186099	6.21E-005	XPNPEP3
2522	A_37_P032129	6.22E-005	C16orf86
2523	A_37_P087719	6.22E-005	ABHD15
2524	A_37_P094946	6.22E-005	PTCH1
2525	A_37_P316407	6.22E-005	APBA1
2526	A_37_P219052	6.22E-005	PCOTH
2527	A_37_P385559	6.23E-005	PILRB
2528	A_37_P224306	6.23E-005	Sep-08
2529	A_37_P150003	6.24E-005	PGPEP1
2530	A_37_P109284	6.24E-005	ABCA4
2531	A_37_P135185	6.27E-005	RCAN2
2532	A_23_P330537	6.27E-005	SPSB3
2533	A_37_P207818	6.28E-005	DUSP8
2534	A_37_P116660	6.28E-005	GSTM4
2535	A_37_P400189	6.29E-005	AVEN
2536	A_37_P018704	6.30E-005	PARL
2537	A_37_P182170	6.31E-005	GGT5
2538	A_37_P139914	6.33E-005	PBX2
2539	A_37_P388244	6.34E-005	TSPAN33
2540	A_37_P090297	6.34E-005	ADAMTSL2
2541	A_37_P292233	6.39E-005	USP30
2542	A_37_P049736	6.41E-005	LYPD6B
2543	A_37_P017541	6.41E-005	NBEAL2
2544	A_37_P384914	6.41E-005	ZFAND2A
2545	A_37_P029948	6.44E-005	TMEM165
2546	A_37_P172770	6.44E-005	WDYHV1
2547	A_37_P335055	6.44E-005	C1orf50
2548	A_23_P161727	6.45E-005	HSPB2
2549	A_37_P338558	6.46E-005	TSTD1
2550	A_37_P329811	6.47E-005	C14orf43
2551	A_37_P139120	6.47E-005	C6orf1
2552	A_37_P079073	6.50E-005	SIRT7
2553	A_37_P319418	6.52E-005	NIPSNAP3B
2554	A_37_P191880	6.53E-005	SEPHS1
2555	A_37_P149506	6.53E-005	TNNI3
2556	A_37_P099446	6.55E-005	CABLES1
2557	A_37_P166989	6.57E-005	DECR1
2558	A_37_P218994	6.57E-005	OLFM4
2559	A_37_P185410	6.58E-005	SH3BP1
2560	A_37_P104558	6.59E-005	SLC8A3
2561	A_23_P202219	6.59E-005	CALHM2

5891	A_37_P175537	0.00319322	GABRA5
5892	A_37_P200940	0.00319985	ABCG4
5893	A_37_P213110	0.00320156	DAZ2
5894	A_33_P3323559	0.00321094	CRYAA
5895	A_37_P430906	0.00321136	CDHR5
5896	A_37_P204301	0.00321672	NRXN2
5897	A_37_P146193	0.00321886	NUMBL
5898	A_37_P188295	0.00322086	DDRGK1
5899	A_37_P110744	0.00322395	NOL9
5900	A_37_P382536	0.00322649	ATP6V0A4
5901	A_37_P177980	0.00322759	DUOX2
5902	A_37_P077819	0.00322825	ALOX15B
5903	A_37_P335677	0.00322938	CAPZA1
5904	A_37_P056300	0.00323922	VIL1
5905	A_37_P281020	0.0032436	ACVR1B
5906	A_37_P076988	0.0032457	XG
5907	A_37_P117744	0.00324688	ATP13A2
5908	A_37_P368476	0.00324784	PRODH2
5909	A_37_P160806	0.00324874	HOXA1
5910	A_37_P103117	0.00324906	C14orf166B
5911	A_37_P192005	0.00325817	ANKRD2
5912	A_37_P007552	0.00325848	PCSK1
5913	A_37_P150678	0.00326835	PTOV1
5914	A_37_P204083	0.00327357	ANO9
5915	A_37_P292392	0.00327363	WNT5B
5916	A_37_P110163	0.00327968	MCOLN2
5917	A_37_P060495	0.00328696	ENO2
5918	A_37_P272110	0.00328928	REG3A
5919	A_37_P308002	0.00329528	PLXDC1
5920	A_37_P264391	0.0032968	ABCA12
5921	A_37_P195193	0.00329845	GOLGA7B
5922	A_37_P345384	0.00329891	MFSD4
5923	A_37_P132956	0.00330824	C6orf25
5924	A_37_P335172	0.0033189	C1orf88
5925	A_33_P3335183	0.00332352	LRRC37A3
5926	A_37_P147272	0.00332394	ZFR2
5927	A_37_P029440	0.00332416	CCDC149
5928	A_24_P91140	0.00332444	RPL23A
5929	A_37_P199972	0.00332551	RTKN2
5930	A_37_P203897	0.00333479	DRD4
5931	A_37_P060066	0.00334931	MGP
5932	A_37_P287538	0.00335002	C12orf53
5933	A_37_P192522	0.00335488	WDR11
5934	A_37_P341593	0.00336177	DENND1B
5935	A_37_P269318	0.00336664	GALNT5
5936	A_37_P281032	0.00337126	ACVRL1
5937	A_37_P201228	0.00338406	MCAM
5938	A_37_P210244	0.00339896	ROBO3
5939	A_37_P406019	0.00340204	RBFOX2
5940	A_37_P433179	0.00340323	RPS25
5941	A_37_P053444	0.00342257	CALCRL
5942	A_37_P197288	0.00342722	C10orf67
5943	A_37_P120116	0.00343053	HES2
5944	A_37_P174522	0.00343629	GOLGA6A
5945	A_37_P369828	0.00343892	SUGP1
5946	A_37_P086417	0.00344394	CDRT1
5947	A_37_P305600	0.00344417	GPS1
5948	A_37_P328573	0.00344805	GZMB
5949	A_37_P335165	0.00344984	C1orf84
5950	A_37_P080182	0.00345318	ST6GALNAC1
5951	A_37_P212313	0.00346965	TTC12

2562	A_37_P131605	6.60E-005	AIM1
2563	A_37_P147514	6.63E-005	VRK3
2564	A_37_P016307	6.63E-005	DNAJC19
2565	A_37_P116746	6.64E-005	GUK1
2566	A_37_P252975	6.66E-005	SLIT2
2567	A_33_P3326423	6.66E-005	POLL
2568	A_37_P187364	6.67E-005	COL9A3
2569	A_23_P122068	6.68E-005	C1QTNF3
2570	A_37_P187344	6.68E-005	COL20A1
2571	A_37_P426173	6.68E-005	SERGEF
2572	A_37_P143421	6.70E-005	C19orf66
2573	A_37_P243212	6.70E-005	RPL35A
2574	A_37_P111356	6.71E-005	C1orf53
2575	A_37_P219432	6.73E-005	CAB39L
2576	A_37_P065081	6.74E-005	MYO1A
2577	A_37_P052260	6.75E-005	MYT1L
2578	A_37_P058095	6.77E-005	PRR4
2579	A_37_P434037	6.77E-005	SPTBN2
2580	A_37_P050736	6.77E-005	ICA1L
2581	A_37_P018721	6.77E-005	PPARG
2582	A_37_P239085	6.78E-005	AZI2
2583	A_37_P296396	6.78E-005	ENST00000357566
2584	A_37_P215785	6.78E-005	SLC37A1
2585	A_37_P365492	6.78E-005	UST
2586	A_37_P265910	6.80E-005	C2orf18
2587	A_37_P275062	6.81E-005	C2orf55
2588	A_37_P391167	6.81E-005	ZNF34
2589	A_37_P130269	6.82E-005	VASH2
2590	A_37_P071646	6.86E-005	GAGE12D
2591	A_37_P432987	6.86E-005	PCNXL3
2592	A_37_P184657	6.86E-005	P2RX6
2593	A_37_P424743	6.87E-005	SLC43A1
2594	A_37_P158906	6.90E-005	ZNF3
2595	A_37_P424678	6.93E-005	ACAD8
2596	A_37_P111882	6.94E-005	CAMTA1
2597	A_37_P234717	6.94E-005	C3orf20
2598	A_37_P180773	6.96E-005	MAN2C1
2599	A_37_P260461	6.96E-005	MT1M
2600	A_37_P010960	6.97E-005	C3orf10
2601	A_37_P008949	6.97E-005	UBE2D2
2602	A_37_P087422	6.98E-005	SCPEP1
2603	A_23_P130304	6.99E-005	TXNL4A
2604	A_37_P292108	6.99E-005	UBE3B
2605	A_37_P147752	6.99E-005	ZNF256
2606	A_37_P078889	6.99E-005	C17orf89
2607	A_23_P88678	7.00E-005	C15orf27
2608	A_37_P089340	7.00E-005	TUBG2
2609	A_37_P098864	7.01E-005	FIBCD1
2610	A_37_P116362	7.02E-005	GLTPD1
2611	A_37_P362713	7.02E-005	PNLDC1
2612	A_37_P108125	7.02E-005	CCDC88C
2613	A_37_P100228	7.02E-005	MPPE1
2614	A_37_P427526	7.03E-005	DNAJC4
2615	A_37_P301564	7.03E-005	ANAPC11
2616	A_24_P409494	7.05E-005	BCL2L13
2617	A_37_P106695	7.08E-005	TGFB3
2618	A_37_P171413	7.08E-005	RIPK2
2619	A_37_P186156	7.09E-005	ZDHHC8
2620	A_37_P001373	7.10E-005	CDK7
2621	A_37_P155921	7.11E-005	CAV1
2622	A_37_P428773	7.12E-005	STARD10

5952	A_33_P3377529	0.00347341	HOXA4
5953	A_37_P370367	0.00348999	PLAUR
5954	A_37_P170288	0.00348999	EIF3H
5955	A_37_P178606	0.00349139	SPESP1
5956	A_37_P104408	0.00349352	SLC7A7
5957	A_37_P185941	0.00350538	UPB1
5958	A_37_P217061	0.00351057	LECT1
5959	A_37_P389892	0.00351213	XKR6
5960	A_37_P290306	0.00352948	SCN8A
5961	A_37_P088941	0.00353934	CD300E
5962	A_37_P195901	0.00355269	KIAA0913
5963	A_37_P276316	0.00355895	RAB3GAP1
5964	A_37_P099192	0.00356848	KLHL14
5965	A_37_P126512	0.00357374	ENST00000468651
5966	A_37_P138243	0.00357837	NCR2
5967	A_37_P311727	0.00357975	SPACA3
5968	A_33_P3281273	0.00358064	S1PR4
5969	A_33_P3423969	0.0035983	GBX2
5970	A_37_P013756	0.00361353	VEPH1
5971	A_33_P3409506	0.00361758	C9orf85
5972	A_37_P092722	0.00361818	FAM125B
5973	A_37_P150436	0.00362001	ERCC2
5974	A_33_P3407549	0.00363378	ANKRD43
5975	A_37_P305226	0.00363825	CYGB
5976	A_23_P39755	0.00367574	B3GNT7
5977	A_37_P004161	0.00368154	ZFR
5978	A_37_P331991	0.0036856	FBLN5
5979	A_37_P425210	0.00369502	ANO3
5980	A_33_P3489222	0.00371571	PTPN20B
5981	A_23_P413585	0.00372545	FOXD2
5982	A_33_P3393200	0.00373465	SRRM4
5983	A_37_P439917	0.00373474	FLT1
5984	A_37_P101534	0.00373755	PTPRM
5985	A_37_P143438	0.00373766	TMEM161A
5986	A_37_P171587	0.00374225	TRIM35
5987	A_37_P135279	0.00375135	PKHD1
5988	A_37_P181011	0.00375798	UNC13C
5989	A_37_P210677	0.0037585	SERPING1
5990	A_24_P188878	0.00375963	RPL34
5991	A_37_P181758	0.00376746	C22orf15
5992	A_37_P077003	0.00376888	XK
5993	A_37_P039293	0.00377273	UMOD
5994	A_37_P214944	0.00377297	DNMT3L
5995	A_37_P140153	0.00377626	RWDD1
5996	A_37_P294445	0.0037802	DACH2
5997	A_37_P167087	0.00378022	DEPTOR
5998	A_37_P371386	0.00378559	ODF3L2
5999	A_24_P403459	0.00378775	IFNA4
6000	A_37_P147427	0.00380679	ZNF99
6001	A_23_P39356	0.0038137	FFAR1
6002	A_37_P224291	0.00381883	SLC12A7
6003	A_37_P209422	0.00381888	SYT13
6004	A_37_P118706	0.00381958	LCK
6005	A_37_P136546	0.00382279	BACH2
6006	A_23_P207106	0.00382877	CHRNB1
6007	A_37_P351680	0.00383081	SLC45A1
6008	A_37_P091617	0.00383248	SUSD1
6009	A_37_P376656	0.00383932	RINL
6010	A_37_P018448	0.00384991	C3orf34
6011	A_37_P162305	0.00387059	C7orf60
6012	A_37_P120304	0.00387367	LOC728989

2623	A_37_P021557	7.12E-005	NEK4
2624	A_37_P240129	7.12E-005	MUC4
2625	A_37_P110300	7.14E-005	SSU72
2626	A_37_P418685	7.14E-005	ARHGAP22
2627	A_37_P255651	7.14E-005	WDR59
2628	A_37_P138921	7.14E-005	PKIB
2629	A_33_P3223713	7.16E-005	UBE2E3
2630	A_37_P349158	7.17E-005	PTCHD2
2631	A_37_P087125	7.18E-005	EZH1
2632	A_37_P200922	7.18E-005	PRCP
2633	A_37_P113519	7.19E-005	RORC
2634	A_33_P3650224	7.20E-005	CXorf49B
2635	A_37_P145018	7.21E-005	DOT1L
2636	A_37_P375006	7.21E-005	RYR1
2637	A_33_P3268564	7.21E-005	NCK2
2638	A_23_P47527	7.21E-005	C11orf10
2639	A_37_P071605	7.22E-005	GABRQ
2640	A_33_P3382309	7.22E-005	PRDM16
2641	A_33_P3240767	7.23E-005	CIDEC
2642	A_37_P389149	7.23E-005	ZYX
2643	A_37_P310598	7.23E-005	EVPL
2644	A_33_P3289128	7.23E-005	ZBTB42
2645	A_37_P051758	7.24E-005	PLB1
2646	A_37_P083003	7.24E-005	ZNF232
2647	A_37_P067927	7.27E-005	CD163L1
2648	A_37_P157887	7.28E-005	TSPAN12
2649	A_37_P183457	7.29E-005	DERL3
2650	A_37_P425250	7.29E-005	AP2A2
2651	A_37_P110444	7.29E-005	ACAP3
2652	A_37_P060316	7.29E-005	DYRK4
2653	A_37_P030963	7.29E-005	ABCC6
2654	A_33_P3398331	7.31E-005	MMP24
2655	A_37_P082454	7.32E-005	ATP2A3
2656	A_37_P135607	7.35E-005	HLA-F
2657	A_37_P080643	7.36E-005	PIK3R5
2658	A_37_P408075	7.36E-005	EFCAB6
2659	A_37_P153949	7.37E-005	ZNF749
2660	A_37_P112112	7.37E-005	CCDC24
2661	A_37_P143328	7.38E-005	C19orf33
2662	A_37_P374855	7.42E-005	RPS11
2663	A_37_P245190	7.43E-005	TTC21A
2664	A_37_P369423	7.43E-005	FZR1
2665	A_37_P218271	7.44E-005	SLC25A30
2666	A_37_P341959	7.45E-005	TNFRSF4
2667	A_37_P140372	7.46E-005	SKIV2L
2668	A_23_P91221	7.47E-005	PKIG
2669	A_33_P3347281	7.47E-005	HNMT
2670	A_37_P146769	7.50E-005	ATP5SL
2671	A_37_P031749	7.50E-005	BAIAP3
2672	A_37_P166216	7.51E-005	C8orf33
2673	A_37_P150156	7.51E-005	PLD3
2674	A_37_P128090	7.52E-005	JTB
2675	A_37_P049995	7.54E-005	E2F6
2676	A_37_P145433	7.54E-005	SLC1A5
2677	A_37_P172733	7.55E-005	WDR67
2678	A_37_P091589	7.56E-005	AKNA
2679	A_37_P115031	7.57E-005	EPHA8
2680	A_37_P081338	7.57E-005	FKBP10
2681	A_37_P175303	7.58E-005	FAM81A
2682	A_37_P193436	7.58E-005	SORBS1
2683	A_37_P235557	7.58E-005	CMC1

6013	A_37_P092574	0.0038802	NR6A1
6014	A_37_P069056	0.0038868	ZNF641
6015	A_37_P288732	0.00388904	POLE
6016	A_37_P170042	0.0038923	EBF2
6017	A_37_P130489	0.00389303	KIAA1751
6018	A_23_P119478	0.00389652	EBI3
6019	A_37_P291867	0.00389871	TPCN1
6020	A_37_P398061	0.0038991	CKMT1A
6021	A_37_P067233	0.00390723	SOAT2
6022	A_37_P091948	0.00390812	ROR2
6023	A_37_P165452	0.00391459	ZNF746
6024	A_37_P010612	0.0039157	ATRIP
6025	A_37_P055235	0.00392145	TEKT4
6026	A_37_P107984	0.00393927	CCDC85C
6027	A_37_P182825	0.00394801	FBLN1
6028	A_37_P021321	0.00394947	TRAK1
6029	A_37_P191147	0.00394953	TOX2
6030	A_37_P180885	0.00396174	TTLL13
6031	A_37_P066962	0.00396177	PPFIA2
6032	A_37_P113547	0.00396724	PIGR
6033	A_37_P400611	0.00397176	OCA2
6034	A_23_P89101	0.00397557	Sep-12
6035	A_37_P299461	0.00398241	KDM5C
6036	A_37_P017183	0.00398475	MYH15
6037	A_24_P85775	0.00398549	C1orf38
6038	A_37_P081626	0.0040162	GALR2
6039	A_37_P163214	0.00402125	CARD11
6040	A_37_P318033	0.00402352	ZER1
6041	A_37_P129070	0.00403158	TIE1
6042	A_37_P092755	0.00403797	FAM22G
6043	A_37_P319179	0.00404825	MUSK
6044	A_37_P246209	0.00404973	ALPK1
6045	A_33_P3328726	0.00406605	CCDC33
6046	A_37_P118865	0.00407945	LIN28A
6047	A_37_P096293	0.00409022	PHYHD1
6048	A_37_P138640	0.00410106	PACSIN1
6049	A_37_P435182	0.00410353	STIM1
6050	A_33_P3257703	0.00411691	C9orf131
6051	A_37_P234076	0.00412187	ARPP21
6052	A_37_P165501	0.00412812	MBOAT4
6053	A_37_P011060	0.00412967	C3orf45
6054	A_37_P158378	0.00413721	A_37_P158378
6055	A_37_P044679	0.00413736	EHD3
6056	A_37_P167114	0.00414068	DLGAP2
6057	A_37_P113623	0.00414935	CTRC
6058	A_37_P342914	0.00415206	WNT4
6059	A_37_P001067	0.00415788	COL23A1
6060	A_37_P315341	0.00415885	CORO2A
6061	A_37_P045466	0.00416072	VPS54
6062	A_37_P232845	0.00416603	SEMA5A
6063	A_37_P027670	0.00417091	NIPAL1
6064	A_37_P207375	0.00417126	C11orf21
6065	A_33_P3336113	0.00417323	TIGD3
6066	A_37_P370266	0.00417996	SYCN
6067	A_37_P149542	0.0041857	NRTN
6068	A_37_P005607	0.00418637	MYOT
6069	A_37_P352850	0.00419435	TMCO2
6070	A_37_P231531	0.00420525	ENST00000507527
6071	A_37_P142711	0.00422623	NLRP9
6072	A_37_P024725	0.00424219	FAT4
6073	A_37_P418818	0.00424405	MMRN2

2684	A_37_P205019	7.58E-005	GLB1L2
2685	A_37_P080115	7.60E-005	GIP
2686	A_37_P182581	7.60E-005	EIF3L
2687	A_37_P076372	7.61E-005	TMEM164
2688	A_23_P59613	7.62E-005	FZD9
2689	A_37_P068049	7.63E-005	TMEM5
2690	A_37_P087645	7.64E-005	FAM134C
2691	A_33_P3287800	7.65E-005	LBX2
2692	A_37_P033383	7.65E-005	ZNF434
2693	A_37_P380903	7.66E-005	PION
2694	A_37_P071616	7.67E-005	GAGE1
2695	A_37_P106895	7.67E-005	PLD4
2696	A_37_P169461	7.68E-005	HEY1
2697	A_37_P370521	7.71E-005	KCTD15
2698	A_23_P252082	7.71E-005	TMEM176A
2699	A_37_P095002	7.71E-005	PTGES
2700	A_37_P194730	7.72E-005	FANK1
2701	A_37_P422540	7.72E-005	PNLIP
2702	A_23_P93973	7.73E-005	TRPV5
2703	A_37_P412959	7.75E-005	EEF1A2
2704	A_37_P095266	7.75E-005	MAN1B1
2705	A_37_P151799	7.78E-005	JSRP1
2706	A_37_P092468	7.81E-005	TBC1D2
2707	A_37_P057096	7.81E-005	AACS
2708	A_37_P077471	7.82E-005	ADAM11
2709	A_37_P157112	7.82E-005	MICALL2
2710	A_37_P109528	7.83E-005	SSR2
2711	A_37_P007200	7.84E-005	RGS14
2712	A_37_P333245	7.84E-005	AKIRIN1
2713	A_37_P196049	7.85E-005	KNDC1
2714	A_37_P154553	7.90E-005	ACTR3B
2715	A_37_P176210	7.90E-005	ISL2
2716	A_37_P206605	7.90E-005	DLG2
2717	A_37_P310022	7.92E-005	PCTP
2718	A_37_P203065	7.92E-005	MEN1
2719	A_37_P091113	7.93E-005	C9orf9
2720	A_33_P3316639	7.96E-005	VPS24
2721	A_37_P152642	7.96E-005	TMPRSS9
2722	A_33_P3323448	7.97E-005	HM13
2723	A_33_P3323068	7.99E-005	AGPAT4
2724	A_37_P143090	8.03E-005	BCAM
2725	A_37_P126361	8.03E-005	RNF220
2726	A_37_P098737	8.04E-005	VLDLR
2727	A_37_P432128	8.05E-005	MYO7A
2728	A_37_P045444	8.07E-005	RNF103-VPS24
2729	A_37_P422805	8.10E-005	RASSF4
2730	A_23_P64808	8.11E-005	HOXC13
2731	A_37_P117170	8.11E-005	HMCN1
2732	A_37_P313971	8.12E-005	ADAMTSL1
2733	A_37_P314443	8.14E-005	NOL6
2734	A_37_P131321	8.16E-005	ZNF695
2735	A_37_P005184	8.17E-005	LSM11
2736	A_37_P068766	8.17E-005	TRPV4
2737	A_37_P388914	8.18E-005	ZNF273
2738	A_37_P091724	8.20E-005	COBRA1
2739	A_33_P3356926	8.22E-005	APBB3
2740	A_37_P216785	8.25E-005	CDADC1
2741	A_37_P200563	8.27E-005	VWA2
2742	A_37_P007382	8.27E-005	CAMK2A
2743	A_33_P3312790	8.27E-005	MEGF11
2744	A_37_P100442	8.27E-005	LAMA3
6074	A_37_P216386	0.00427178	TUBA3C
6075	A_37_P119469	0.00427749	MTHFR
6076	A_37_P425490	0.00427933	RASGRP2
6077	A_37_P416384	0.00427938	C10orf129
6078	A_37_P399673	0.00428332	PLA2G4E
6079	A_37_P049880	0.00428822	MARCO
6080	A_37_P141120	0.00429463	TFAP2B
6081	A_37_P150613	0.00429547	SYT3
6082	A_37_P114740	0.00429915	DYRK3
6083	A_37_P172106	0.00431059	TG
6084	A_37_P080248	0.00431099	LGALS9B
6085	A_37_P148622	0.00431725	DUXA
6086	A_37_P201477	0.00432131	ANO1
6087	A_37_P171231	0.00433514	RALYL
6088	A_37_P377197	0.00435508	ZNF227
6089	A_37_P172776	0.00436276	WISP1
6090	A_37_P031913	0.00436282	ACSM1
6091	A_37_P062581	0.00437414	LEPREL2
6092	A_37_P130822	0.00437431	TMOD4
6093	A_37_P230805	0.004375	RASGRF2
6094	A_37_P348549	0.00438723	SPTA1
6095	A_37_P323077	0.00439083	C18orf1
6096	A_37_P412646	0.00441598	PLTP
6097	A_37_P087252	0.00442052	RPL27
6098	A_33_P3376541	0.00442978	LOC285033
6099	A_37_P368301	0.00445811	CYP2S1
6100	A_37_P012451	0.00445819	SLC6A20
6101	A_37_P146248	0.004466	ASPDH
6102	A_37_P435777	0.00446711	TMPRSS4
6103	A_37_P187369	0.00447247	GFRA4
6104	A_37_P113746	0.00447677	ST7L
6105	A_37_P237260	0.00448607	FBXL2
6106	A_37_P092168	0.00449884	GLDC
6107	A_37_P170338	0.0045048	NRG1
6108	A_23_P329924	0.00451157	GPR109A
6109	A_24_P148907	0.00454906	MAB21L2
6110	A_23_P34744	0.00456869	CTSK
6111	A_37_P413791	0.00459077	PLUNC
6112	A_37_P359304	0.00459187	HLA-DRA
6113	A_37_P117592	0.00460427	IL12RB2
6114	A_37_P331172	0.00460686	RPGRIP1
6115	A_23_P71926	0.00461047	PRKACG
6116	A_37_P150083	0.00461365	IL12RB1
6117	A_37_P065068	0.004621	OAS3
6118	A_37_P310082	0.00462282	IKZF3
6119	A_37_P030596	0.00463547	ZAR1
6120	A_37_P217487	0.00464279	FREM2
6121	A_37_P199912	0.00465072	TACR2
6122	A_37_P179315	0.00465651	PATL2
6123	A_23_P130585	0.004659	CIB3
6124	A_37_P132124	0.00466295	BEND6
6125	A_37_P165112	0.00466601	THSD7A
6126	A_37_P195453	0.00468614	PSD
6127	A_37_P118377	0.00469195	KIF26B
6128	A_37_P066704	0.00469717	ITGB7
6129	A_37_P129764	0.00469898	TTC24
6130	A_23_P111088	0.0047003	OR2B2
6131	A_37_P141512	0.00470724	TTBK1
6132	A_37_P099587	0.00470853	CDH20
6133	A_37_P228601	0.00471653	DOK3
6134	A_37_P138586	0.00473591	ELOVL2

2745	A_37_P349094	8.29E-005	PSEN2
2746	A_37_P273297	8.31E-005	LPIN1
2747	A_37_P161788	8.31E-005	SYPL1
2748	A_37_P014584	8.32E-005	IL17RC
2749	A_37_P144920	8.33E-005	GMIP
2750	A_37_P012318	8.35E-005	PIGZ
2751	A_37_P065843	8.36E-005	PRPF40B
2752	A_37_P089356	8.38E-005	TUSC5
2753	A_37_P030087	8.39E-005	PDLIM3
2754	A_37_P337712	8.39E-005	GLIS1
2755	A_37_P139545	8.39E-005	PSMG4
2756	A_37_P141566	8.40E-005	TUBB
2757	A_37_P131950	8.43E-005	FRS3
2758	A_37_P350058	8.44E-005	RNF207
2759	A_37_P224243	8.44E-005	PRELID2
2760	A_37_P203165	8.44E-005	GDPD5
2761	A_33_P3414242	8.45E-005	MOG
2762	A_37_P133358	8.47E-005	SUPT3H
2763	A_37_P039103	8.47E-005	CCDC78
2764	A_37_P072619	8.48E-005	AWAT2
2765	A_23_P56404	8.49E-005	EN1
2766	A_37_P027873	8.49E-005	OSTC
2767	A_37_P391864	8.51E-005	GPR124
2768	A_37_P207940	8.51E-005	METTL12
2769	A_23_P210164	8.53E-005	HOXD8
2770	A_37_P246699	8.53E-005	RNF212
2771	A_24_P678104	8.56E-005	STMN3
2772	A_37_P157937	8.56E-005	GBAS
2773	A_33_P3269728	8.56E-005	ANKRD10
2774	A_23_P106204	8.56E-005	GSTZ1
2775	A_37_P333539	8.58E-005	MTOR
2776	A_37_P082989	8.60E-005	B3GNTL1
2777	A_37_P024688	8.60E-005	FAM47E
2778	A_23_P142878	8.60E-005	ATOH8
2779	A_37_P232517	8.65E-005	PDLIM7
2780	A_37_P321282	8.65E-005	SLC25A25
2781	A_37_P358263	8.66E-005	C6orf94
2782	A_37_P045281	8.68E-005	CXCR4
2783	A_37_P117803	8.68E-005	IQCC
2784	A_37_P033370	8.70E-005	TBC1D10B
2785	A_37_P043881	8.70E-005	D2HGDH
2786	A_37_P045167	8.72E-005	FBLN7
2787	A_37_P318955	8.72E-005	MAMDC4
2788	A_37_P125461	8.75E-005	PTGFRN
2789	A_37_P178713	8.75E-005	IGDCC4
2790	A_37_P152936	8.76E-005	TTYH1
2791	A_37_P217029	8.76E-005	ALG5
2792	A_37_P185177	8.77E-005	PPP6R2
2793	A_37_P186376	8.77E-005	ANKRD5
2794	A_33_P3361442	8.78E-005	C19orf73
2795	A_37_P190253	8.80E-005	PXMP4
2796	A_37_P390618	8.80E-005	TATDN1
2797	A_37_P300562	8.80E-005	VSIG1
2798	A_33_P3424507	8.84E-005	OR51F1
2799	A_37_P076662	8.85E-005	FGD1
2800	A_37_P089471	8.85E-005	SRCIN1
2801	A_37_P104912	8.87E-005	KIAA0391
2802	A_33_P3226665	8.88E-005	ZNF773
2803	A_37_P118031	8.88E-005	KCTD3
2804	A_37_P366286	8.88E-005	NLRP13
2805	A_37_P077959	8.91E-005	Sep-04

6135	A_37_P004502	0.00474234	HEATR7B2
6136	A_37_P306216	0.00475774	KCNH6
6137	A_37_P149639	0.00476179	ZNF560
6138	A_37_P115830	0.00477548	FMO1
6139	A_37_P406146	0.00477973	TBC1D10A
6140	A_23_P257164	0.00478369	AMT
6141	A_37_P069329	0.00479624	FOXP3
6142	A_37_P430387	0.0048151	ENST00000356191
6143	A_37_P215489	0.00482327	PCNT
6144	A_37_P178675	0.00483118	ODF3L1
6145	A_37_P098074	0.00483566	FANCC
6146	A_32_P194821	0.00484379	RPL21
6147	A_37_P055641	0.00484775	CD8B
6148	A_37_P422715	0.00485249	LOXL4
6149	A_37_P109697	0.00485347	A_37_P109697
6150	A_37_P080610	0.00487266	DNAI2
6151	A_37_P067607	0.00487313	SUOX
6152	A_37_P145227	0.00488431	EPS8L1
6153	A_37_P187005	0.00489592	SLC13A3
6154	A_37_P205795	0.00489681	INSC
6155	A_37_P190851	0.00490269	SEL1L2
6156	A_37_P389966	0.00493114	SGK3
6157	A_37_P024288	0.00493338	DOK7
6158	A_37_P394706	0.00493753	SDR16C5
6159	A_37_P033440	0.00493771	DPEP1
6160	A_37_P316230	0.00494538	GOLGA1
6161	A_37_P011835	0.00495706	CLSTN2
6162	A_37_P127683	0.00497831	FAM151A
6163	A_37_P311817	0.00498666	SPNS3
6164	A_37_P425275	0.00498817	APLP2
6165	A_37_P193755	0.0049953	CYP2E1
6166	A_33_P3382331	0.00499921	HSPA6
6167	A_37_P066061	0.00501672	RAB3IP
6168	A_23_P14986	0.00504892	HSD11B2
6169	A_37_P302322	0.00504975	KRT27
6170	A_37_P204540	0.00505114	FAT3
6171	A_37_P123704	0.00506028	PADI6
6172	A_37_P331707	0.00506353	KCNK10
6173	A_37_P166204	0.00506707	C8orf30A
6174	A_37_P006478	0.00506905	IL17B
6175	A_37_P096926	0.00508329	C9orf50
6176	A_33_P3287477	0.0050898	C10orf120
6177	A_37_P429896	0.00510068	HBB
6178	A_37_P217685	0.00510083	RCBTB2
6179	A_37_P397404	0.00511005	ACSBG1
6180	A_37_P180084	0.00511125	SMAD3
6181	A_37_P045021	0.00511484	FAM179A
6182	A_33_P3251462	0.00511816	C20orf141
6183	A_37_P143872	0.00512175	CD22
6184	A_37_P191196	0.00512297	RIMS4
6185	A_37_P075029	0.00513835	SLC35A2
6186	A_37_P139819	0.00514104	RRAGD
6187	A_33_P3311267	0.00515708	KRTAP19-2
6188	A_37_P115861	0.00515869	FMO4
6189	A_33_P3246763	0.00516233	AANAT
6190	A_37_P148464	0.00519843	HOOK2
6191	A_37_P112956	0.00522619	CLCA1
6192	A_37_P214640	0.00523721	HUNK
6193	A_37_P374601	0.00524438	RCN3
6194	A_37_P146938	0.0052478	KIR2DL4
6195	A_37_P197212	0.00526576	C10orf27

2806	A_37_P150959	8.91E-005	CA11
2807	A_37_P001914	8.92E-005	CSF1R
2808	A_37_P144831	8.96E-005	ZNF44
2809	A_37_P049978	8.96E-005	HS1BP3
2810	A_37_P156305	8.97E-005	COPG2
2811	A_23_P75283	8.98E-005	RBP4
2812	A_37_P172862	8.98E-005	TONSL
2813	A_37_P089015	8.99E-005	TMUB2
2814	A_37_P044821	9.00E-005	EML6
2815	A_33_P3260373	9.02E-005	RAB43
2816	A_37_P343500	9.03E-005	PLEKHA6
2817	A_37_P214249	9.06E-005	CHAF1B
2818	A_37_P175976	9.07E-005	PLA2G4F
2819	A_37_P106856	9.08E-005	TGM1
2820	A_33_P3233273	9.08E-005	LRRC61
2821	A_37_P195365	9.09E-005	MORN4
2822	A_37_P335557	9.11E-005	CAMK1G
2823	A_37_P087593	9.11E-005	TMEM11
2824	A_37_P135413	9.16E-005	HCRTR2
2825	A_33_P3403399	9.17E-005	SLC47A1
2826	A_37_P399955	9.18E-005	JMJD7
2827	A_37_P066352	9.20E-005	TUBA1B
2828	A_37_P142385	9.22E-005	MBOAT7
2829	A_37_P391137	9.22E-005	NRBP2
2830	A_37_P077794	9.24E-005	ALOX12
2831	A_37_P085800	9.27E-005	P2RX5
2832	A_37_P096047	9.27E-005	ORM2
2833	A_23_P13713	9.28E-005	PRPH
2834	A_37_P152739	9.29E-005	FBN3
2835	A_37_P204935	9.29E-005	CYP2R1
2836	A_37_P192615	9.30E-005	ANAPC16
2837	A_23_P8571	9.32E-005	SRCRB4D
2838	A_37_P120985	9.32E-005	SPATA6
2839	A_37_P257940	9.32E-005	NTHL1
2840	A_37_P090798	9.33E-005	WDR31
2841	A_37_P014788	9.34E-005	ITGA9
2842	A_37_P015480	9.35E-005	ZMYND10
2843	A_37_P289905	9.35E-005	TMCC3
2844	A_33_P3292297	9.37E-005	COPS7B
2845	A_33_P3394021	9.37E-005	KLHL12
2846	A_37_P026652	9.38E-005	SPARCL1
2847	A_37_P027357	9.38E-005	HSD17B11
2848	A_37_P442937	9.39E-005	N6AMT2
2849	A_37_P430470	9.39E-005	PNPLA2
2850	A_37_P168983	9.41E-005	LETM2
2851	A_37_P039477	9.41E-005	TMEM219
2852	A_37_P191387	9.41E-005	VPS16
2853	A_37_P185719	9.42E-005	TYMP
2854	A_37_P210175	9.43E-005	RIC8A
2855	A_37_P417162	9.44E-005	METTL10
2856	A_37_P067242	9.45E-005	SOCS2
2857	A_37_P375220	9.46E-005	SHD
2858	A_33_P3333156	9.47E-005	C11orf70
2859	A_37_P337275	9.47E-005	NPHP4
2860	A_23_P217009	9.47E-005	C9orf24
2861	A_33_P3410925	9.48E-005	KLF1
2862	A_23_P83298	9.48E-005	PRRX2
2863	A_37_P200345	9.49E-005	TRIM8
2864	A_37_P202904	9.49E-005	SLC15A3
2865	A_37_P182157	9.50E-005	GGT1
2866	A_37_P235970	9.53E-005	ALS2CL

6196	A_37_P113646	0.00527478	LEFTY1
6197	A_37_P142483	0.00528305	KLF16
6198	A_37_P270254	0.00529161	ATG9A
6199	A_33_P3258392	0.00529872	EDN1
6200	A_37_P123275	0.0053078	NTNG1
6201	A_37_P071013	0.00531148	DUSP9
6202	A_37_P189900	0.00532274	PHACTR3
6203	A_37_P158448	0.0053395	HDAC9
6204	A_23_P429998	0.00534138	FOSB
6205	A_37_P079244	0.00534585	RNF43
6206	A_23_P73721	0.00534876	RRAGB
6207	A_37_P388061	0.00535272	FBXL18
6208	A_37_P155322	0.00535283	BBS9
6209	A_37_P313542	0.00536923	PGAP3
6210	A_37_P101227	0.00537163	NFATC1
6211	A_24_P314597	0.0053865	ENST00000335883
6212	A_37_P057503	0.00539546	AMHR2
6213	A_37_P048393	0.00541537	REG1B
6214	A_37_P167987	0.00541637	CYP11B2
6215	A_37_P157233	0.00543024	NRCAM
6216	A_37_P174293	0.00543711	CHD2
6217	A_37_P269459	0.00544955	GKN1
6218	A_37_P008180	0.0054577	C5orf60
6219	A_37_P042913	0.00547421	CCL20
6220	A_37_P023760	0.00547766	GC
6221	A_37_P374590	0.0054853	EXOC3L2
6222	A_33_P3405459	0.00549594	C20orf195
6223	A_23_P375165	0.00549623	TEX19
6224	A_37_P014065	0.00549826	GPR128
6225	A_37_P116152	0.0055181	ARHGEF2
6226	A_37_P004678	0.00555585	A_37_P004678
6227	A_37_P038011	0.00556775	TPSAB1
6228	A_37_P194569	0.0055822	FAM24A
6229	A_37_P193458	0.00558529	GDF10
6230	A_37_P162850	0.0055886	CADPS2
6231	A_37_P013135	0.00560069	LIPH
6232	A_23_P206792	0.00562239	ZNF764
6233	A_37_P044421	0.00562644	ANKRD44
6234	A_37_P424818	0.0056458	ADAMTS15
6235	A_37_P165486	0.00564945	ZPBP
6236	A_37_P065320	0.00566008	PDE1B
6237	A_33_P3304162	0.00567281	COL19A1
6238	A_37_P033653	0.00570576	EME2
6239	A_37_P148758	0.00570607	PNKP
6240	A_37_P095622	0.00571271	NCBP1
6241	A_33_P3305487	0.00571509	REM2
6242	A_23_P309246	0.00571953	ZNF498
6243	A_37_P300423	0.00571981	USP9X
6244	A_37_P045451	0.00576435	FOXN2
6245	A_37_P100195	0.0057671	GNAL
6246	A_37_P195578	0.00579495	HKDC1
6247	A_37_P070161	0.00579495	NOX1
6248	A_37_P202420	0.00581587	TRIM68
6249	A_37_P057806	0.00582576	AQP5
6250	A_37_P354957	0.00582699	CHIT1
6251	A_37_P071301	0.00582837	GPKOW
6252	A_37_P050935	0.0058407	NRP2
6253	A_37_P040511	0.00584475	AAK1
6254	A_37_P338019	0.00586231	DMRTB1
6255	A_23_P131534	0.00587424	GPR45
6256	A_37_P129495	0.00587921	KIAA0319L

2867	A_37_P078307	9.54E-005	BAHCC1
2868	A_37_P075636	9.54E-005	SHROOM2
2869	A_37_P426044	9.55E-005	C11orf9
2870	A_33_P3380051	9.57E-005	MYADML2
2871	A_37_P387740	9.58E-005	CCL26
2872	A_33_P3358208	9.59E-005	PADI1
2873	A_37_P202458	9.60E-005	SNX19
2874	A_37_P150565	9.60E-005	PRKCSH
2875	A_37_P415138	9.63E-005	KIAA1755
2876	A_33_P3320953	9.64E-005	CTXN1
2877	A_33_P3404032	9.65E-005	HIST3H2A
2878	A_33_P3316587	9.65E-005	SLC22A18AS
2879	A_23_P383422	9.68E-005	NFKBID
2880	A_37_P066410	9.69E-005	RPH3A
2881	A_37_P040606	9.69E-005	MBOAT2
2882	A_23_P153676	9.69E-005	TLE2
2883	A_37_P377047	9.70E-005	YJEFN3
2884	A_23_P379794	9.71E-005	PIGW
2885	A_37_P268975	9.71E-005	FKBP1B
2886	A_37_P089736	9.71E-005	PER1
2887	A_37_P248714	9.74E-005	FRAS1
2888	A_37_P401597	9.74E-005	LRRC28
2889	A_33_P3355418	9.75E-005	AGBL1
2890	A_37_P325738	9.75E-005	KDSR
2891	A_37_P170044	9.76E-005	SLURP1
2892	A_37_P208434	9.77E-005	NAALAD2
2893	A_37_P336292	9.77E-005	CELSR2
2894	A_37_P145255	9.78E-005	AP1M2
2895	A_37_P431626	9.79E-005	HRASLS2
2896	A_37_P043638	9.79E-005	PRKRA
2897	A_37_P248195	9.80E-005	EVC
2898	A_37_P133779	9.80E-005	CUL9
2899	A_37_P357639	9.80E-005	DCBLD1
2900	A_37_P411270	9.80E-005	CPXM1
2901	A_37_P115160	9.80E-005	EXTL1
2902	A_37_P430451	9.82E-005	MTL5
2903	A_37_P372129	9.83E-005	LTBP4
2904	A_24_P395814	9.84E-005	CGB
2905	A_37_P209450	9.85E-005	PHRF1
2906	A_37_P426753	9.86E-005	PPP1R14B
2907	A_37_P309644	9.87E-005	NLK
2908	A_37_P021521	9.92E-005	ARPC4-TTLL3
2909	A_37_P122756	9.92E-005	NENF
2910	A_23_P202206	9.93E-005	GSTO2
2911	A_37_P104398	9.94E-005	FLVCR2
2912	A_37_P142899	9.95E-005	ARMC6
2913	A_37_P151947	9.97E-005	CCDC151
2914	A_37_P058158	9.98E-005	BLOC1S1
2915	A_37_P260291	9.99E-005	C16orf7
2916	A_37_P170900	0.00010004	PARP10
2917	A_37_P013448	0.00010025	FAM55C
2918	A_37_P090567	0.00010026	TTC39B
2919	A_37_P079812	0.00010039	GFAP
2920	A_37_P077857	0.00010042	CLDN7
2921	A_37_P131199	0.00010049	PRAMEF4
2922	A_37_P149872	0.0001005	PDCD2L
2923	A_37_P055905	0.0001005	TTC31
2924	A_37_P351017	0.00010056	DRAM2
2925	A_37_P116656	0.00010078	GSTM2
2926	A_33_P3221868	0.00010086	TMEM216
2927	A_23_P41390	0.00010089	SH3TC1

6257	A_37_P016558	0.00592621	RETNLB
6258	A_37_P122143	0.00592938	MTR
6259	A_37_P208254	0.00593833	ENST00000406844
6260	A_37_P117031	0.00595212	HHAT
6261	A_37_P214577	0.00595634	TMPRSS15
6262	A_37_P416006	0.00596316	NMT2
6263	A_37_P165279	0.00596769	TRIM74
6264	A_37_P161887	0.00597751	EPHA1
6265	A_37_P293174	0.00598227	ARSH
6266	A_37_P039946	0.00599324	RANBP10
6267	A_37_P032708	0.00599871	CHTF18
6268	A_37_P184693	0.00601431	C22orf26
6269	A_37_P118505	0.00603626	KMO
6270	A_37_P099882	0.0060856	DCC
6271	A_37_P073690	0.00609022	TMEM27
6272	A_37_P019060	0.00609982	SEC13
6273	A_37_P307789	0.00610771	DOC2B
6274	A_37_P382087	0.00615883	A_37_P382087
6275	A_37_P023140	0.00616039	BST1
6276	A_37_P010235	0.00617715	ABI3BP
6277	A_23_P101642	0.00617734	PTPRH
6278	A_37_P048375	0.00617998	SFTPB
6279	A_37_P005469	0.00619104	STK10
6280	A_37_P012368	0.00619707	LEPREL1
6281	A_37_P190465	0.00620158	RTEL1
6282	A_37_P159010	0.0062188	KRBA1
6283	A_37_P112997	0.00622929	CLCA4
6284	A_37_P256618	0.0062324	CNTNAP4
6285	A_37_P133827	0.00623939	CRISP3
6286	A_37_P426449	0.00627662	CCDC83
6287	A_37_P407565	0.00627879	SYN3
6288	A_37_P111683	0.00628021	COL24A1
6289	A_24_P117410	0.00628246	KLHDC7B
6290	A_37_P230254	0.006312	C5orf25
6291	A_37_P096630	0.00631389	LPAR1
6292	A_37_P167098	0.00631705	CSGALNACT1
6293	A_37_P355465	0.00636172	MCM3
6294	A_23_P114883	0.00636247	FMOD
6295	A_37_P134278	0.00636566	ECT2L
6296	A_37_P020410	0.00639028	FAM3D
6297	A_37_P162439	0.00639256	RAMP3
6298	A_37_P423735	0.0063965	KCNIP2
6299	A_37_P156604	0.00639953	CREB5
6300	A_37_P219367	0.00643576	TPT1
6301	A_37_P209923	0.00647181	PATE2
6302	A_37_P077018	0.00647591	XPNPEP2
6303	A_37_P075669	0.0064785	CASK
6304	A_37_P145549	0.00648601	FGF22
6305	A_37_P154777	0.00648949	ABCB1
6306	A_37_P172993	0.00650876	ZNF696
6307	A_37_P189942	0.00651269	PI3
6308	A_37_P112905	0.00651317	LAMB3
6309	A_37_P045529	0.00657989	CYP27C1
6310	A_37_P088070	0.0065849	TAC4
6311	A_37_P115400	0.00658976	FAM40A
6312	A_37_P405264	0.00662631	APOL1
6313	A_37_P126859	0.00664093	TMEM56-RWDD3
6314	A_37_P212673	0.00665774	VPS11
6315	A_37_P138397	0.00666633	NR2E1
6316	A_37_P160809	0.00668809	HOXA10
6317	A_37_P104964	0.00672342	NYNRIN

2928	A_37_P212387	0.00010099	CDC42BPG
2929	A_37_P224867	0.00010104	CAMLG
2930	A_37_P358392	0.00010119	GPLD1
2931	A_37_P002267	0.00010121	NRG2
2932	A_37_P100872	0.00010125	PTPN2
2933	A_37_P175420	0.00010133	FES
2934	A_37_P205097	0.00010158	SLC22A25
2935	A_37_P050294	0.00010173	MTX2
2936	A_37_P218678	0.00010178	DZIP1
2937	A_37_P021548	0.00010181	SEMA3G
2938	A_37_P107663	0.00010181	VTI1B
2939	A_37_P124094	0.00010184	PGD
2940	A_37_P273060	0.00010191	HJURP
2941	A_37_P137189	0.00010192	SGK1
2942	A_37_P015985	0.000102	BDH1
2943	A_37_P255186	0.000102	RNF166
2944	A_37_P255205	0.00010207	APOB48R
2945	A_37_P121150	0.00010216	MYBPHL
2946	A_23_P78980	0.0001023	B3GNT3
2947	A_37_P339063	0.00010252	FAM19A3
2948	A_37_P137918	0.00010254	TBC1D7
2949	A_37_P014019	0.00010276	MON1A
2950	A_37_P124257	0.00010283	SDF4
2951	A_37_P152457	0.00010309	TLE6
2952	A_37_P007874	0.00010324	SLC36A2
2953	A_37_P119097	0.0001033	ZBTB17
2954	A_37_P215914	0.00010337	CCT8
2955	A_37_P047930	0.00010344	HDAC4
2956	A_37_P041043	0.00010348	CLASP1
2957	A_37_P073624	0.0001035	SLC25A6
2958	A_37_P170972	0.00010364	PREX2
2959	A_37_P374858	0.00010376	RPS15
2960	A_37_P072907	0.00010391	BCAP31
2961	A_37_P057241	0.00010392	ACCN2
2962	A_37_P427246	0.00010403	DAK
2963	A_37_P140533	0.0001041	PPIL1
2964	A_37_P252535	0.00010439	SCOC
2965	A_37_P015154	0.00010447	TNIK
2966	A_37_P257841	0.00010452	PRSS8
2967	A_37_P076321	0.00010457	NDUFB11
2968	A_24_P289178	0.0001046	C16orf74
2969	A_33_P3347330	0.00010468	TOMM7
2970	A_37_P133594	0.00010469	SERPINB1
2971	A_37_P397757	0.00010491	CAPN3
2972	A_37_P183423	0.00010496	ISX
2973	A_37_P186852	0.0001051	C20orf24
2974	A_37_P228256	0.00010513	BOD1
2975	A_37_P287798	0.00010532	HPD
2976	A_37_P204229	0.00010548	EPS8L2
2977	A_37_P178021	0.00010548	MAN2A2
2978	A_37_P084747	0.00010559	MAPK7
2979	A_24_P945181	0.00010566	RBM15B
2980	A_37_P155671	0.00010567	C7orf42
2981	A_37_P241113	0.00010583	SLC26A6
2982	A_37_P320687	0.00010606	RECK
2983	A_37_P073878	0.0001061	MAMLD1
2984	A_37_P004181	0.00010614	MAST4
2985	A_37_P341228	0.00010653	TNFRSF18
2986	A_37_P392039	0.00010656	HEATR7A
2987	A_37_P014145	0.00010657	GRM2
2988	A_37_P015159	0.00010665	PLCD1

6318	A_37_P370504	0.00672854	KCNK6
6319	A_37_P251244	0.00675441	RPL9
6320	A_37_P173376	0.0067842	ANKDD1A
6321	A_37_P407459	0.00679267	BPIL2
6322	A_23_P98744	0.00679465	OR52K2
6323	A_23_P107051	0.00684219	TCAP
6324	A_37_P278336	0.00684907	CCDC148
6325	A_37_P060130	0.00686327	DNAH10
6326	A_37_P154287	0.0068774	ZNF846
6327	A_37_P014222	0.00687785	ASB14
6328	A_37_P370273	0.00688812	IL27RA
6329	A_37_P382496	0.00691827	DENND2A
6330	A_37_P018691	0.00692871	ILDR1
6331	A_37_P148907	0.0069336	OSCAR
6332	A_37_P290252	0.00696086	ITGA5
6333	A_37_P045048	0.00699617	APOB
6334	A_37_P093500	0.00699721	DCTN3
6335	A_37_P007682	0.00700125	SLC22A4
6336	A_37_P353818	0.00700458	MAP3K6
6337	A_37_P107123	0.00702658	PSME1
6338	A_37_P437739	0.00703707	TMPRSS2
6339	A_37_P319974	0.00705544	PHF2
6340	A_37_P128004	0.00706424	SLC6A17
6341	A_37_P104476	0.00706665	FUT8
6342	A_37_P100534	0.00707634	LIPG
6343	A_37_P302782	0.00707773	TEKT3
6344	A_37_P085564	0.00707956	MYL4
6345	A_37_P165470	0.00708046	ZNF777
6346	A_37_P162559	0.00708205	ZNF425
6347	A_37_P057935	0.00709087	ASCL1
6348	A_37_P167867	0.00709211	FKSG2
6349	A_37_P042617	0.00709544	GALNT3
6350	A_37_P072751	0.00710024	NXF3
6351	A_33_P3320017	0.00713399	GALNTL6
6352	A_37_P127823	0.00713795	CCDC163P
6353	A_37_P210536	0.00713905	USP2
6354	A_37_P032220	0.00715261	XYLT1
6355	A_33_P3317797	0.00718211	SLC2A5
6356	A_37_P053150	0.00719301	RPL31
6357	A_37_P174725	0.00719582	DISP2
6358	A_37_P276398	0.00719828	PAX3
6359	A_37_P140970	0.00721812	SYTL3
6360	A_37_P092108	0.0072256	DAPK1
6361	A_37_P004051	0.00722971	HBEGF
6362	A_37_P133704	0.00723564	SLC17A3
6363	A_37_P108721	0.00723635	VSX2
6364	A_37_P058326	0.00724916	C12orf45
6365	A_37_P185573	0.00725138	PPM1F
6366	A_37_P291116	0.0072807	CCDC38
6367	A_37_P404085	0.00729126	LYSMD2
6368	A_37_P030610	0.0073137	Mar-01
6369	A_37_P376839	0.00731867	CEACAM1
6370	A_37_P002716	0.00732051	FGFR4
6371	A_37_P324097	0.0073267	KIAA1468
6372	A_37_P258694	0.00734198	KIAA0513
6373	A_23_P16252	0.00736571	KLK1
6374	A_37_P389877	0.0073747	COL22A1
6375	A_37_P379746	0.00738456	SLC13A4
6376	A_37_P394357	0.00739505	PIWIL2
6377	A_33_P3308137	0.00739855	C7orf46
6378	A_23_P6943	0.00741008	GPR15

2989	A_33_P3235262	0.00010713	PIP5KL1
2990	A_37_P182089	0.0001072	LARGE
2991	A_37_P209768	0.00010768	NAALADL1
2992	A_37_P171927	0.00010779	FAM82B
2993	A_37_P171989	0.00010784	LYNX1
2994	A_37_P338545	0.00010797	EFNA4
2995	A_37_P234795	0.00010805	C3orf52
2996	A_37_P407865	0.00010807	MED15
2997	A_37_P038015	0.0001081	TPSB2
2998	A_37_P434781	0.00010811	SLC35C1
2999	A_23_P4611	0.00010841	SLC27A5
3000	A_37_P054316	0.00010858	SNED1
3001	A_37_P174887	0.00010862	DUOX1
3002	A_37_P042473	0.00010884	ADCY3
3003	A_37_P025599	0.00010916	HTRA3
3004	A_37_P242063	0.00010916	C3orf18
3005	A_37_P110573	0.00011021	ATXN7L2
3006	A_37_P254867	0.0001108	ADCY7
3007	A_37_P034119	0.00011101	GAS8
3008	A_37_P091969	0.0001112	AMBP
3009	A_37_P269247	0.00011129	GAD1
3010	A_37_P058821	0.00011166	CABP1
3011	A_24_P371194	0.00011176	MRPL53
3012	A_37_P034990	0.00011186	KIAA0556
3013	A_23_P49559	0.00011189	GPR142
3014	A_37_P414405	0.0001119	SIGLEC1
3015	A_37_P420881	0.00011201	C10orf128
3016	A_37_P185917	0.00011244	TUBA8
3017	A_37_P141070	0.00011251	TBPL1
3018	A_37_P256239	0.0001126	CDH5
3019	A_37_P187503	0.00011274	CST7
3020	A_37_P106159	0.0001129	C14orf93
3021	A_37_P387972	0.00011292	RADIL
3022	A_37_P204761	0.00011329	CYB5R2
3023	A_37_P274966	0.00011332	C2orf24
3024	A_37_P082122	0.00011352	HEXIM2
3025	A_37_P029277	0.00011372	SMAD1
3026	A_37_P211985	0.00011375	TMEM132A
3027	A_37_P419972	0.00011423	ZCCHC24
3028	A_37_P086655	0.00011425	SDK2
3029	A_37_P079416	0.00011451	CCDC45
3030	A_37_P023768	0.00011462	COMMD8
3031	A_37_P101360	0.00011483	MYO5B
3032	A_37_P083583	0.00011494	BLMH
3033	A_37_P128916	0.00011509	TDRD10
3034	A_37_P163434	0.00011511	LSM5
3035	A_37_P440089	0.00011519	TUBA3D
3036	A_37_P034061	0.00011519	FUK
3037	A_37_P210406	0.00011519	RPS3
3038	A_33_P3220612	0.00011548	FAM89B
3039	A_37_P151988	0.00011571	SPTBN4
3040	A_37_P046430	0.00011583	HOXD9
3041	A_37_P093198	0.0001159	GFI1B
3042	A_37_P365604	0.00011599	VARS2
3043	A_37_P304678	0.00011612	ERBB2
3044	A_37_P003634	0.00011623	SLC9A3
3045	A_37_P199071	0.00011624	PWWP2B
3046	A_23_P112548	0.00011652	HDHD3
3047	A_33_P3306504	0.00011657	ISYNA1
3048	A_37_P101670	0.00011657	RNF138
3049	A_37_P372525	0.00011706	SH2D3A
6379	A_37_P252905	0.0074157	SLC4A4
6380	A_37_P150688	0.00742829	INSR
6381	A_37_P062877	0.00746087	WNT10B
6382	A_37_P296923	0.00749252	DMD
6383	A_37_P175633	0.00749798	GCOM1
6384	A_33_P3364520	0.00750373	KRTAP6-1
6385	A_23_P106661	0.00752491	CMTM1
6386	A_37_P185273	0.00756526	Sep-03
6387	A_37_P389318	0.00757349	ADAM28
6388	A_37_P196704	0.00757549	BLNK
6389	A_37_P126517	0.00760487	ENST00000434802
6390	A_37_P083083	0.0076106	LIG3
6391	A_23_P123424	0.00761844	CHRNB3
6392	A_24_P75190	0.00766344	HBD
6393	A_37_P112375	0.0076723	TARBP1
6394	A_37_P016617	0.00767788	DPPA2
6395	A_37_P264832	0.00768441	NLRC4
6396	A_33_P3285565	0.00769009	CLDN3
6397	A_37_P333264	0.00770219	KLHDC8A
6398	A_37_P114555	0.00770744	GNAT2
6399	A_37_P149573	0.00774064	RFX2
6400	A_37_P249028	0.00774662	ASB5
6401	A_37_P250301	0.00775601	TRPC3
6402	A_37_P001443	0.0077765	CKMT2
6403	A_37_P277586	0.00780618	ITSN2
6404	A_37_P104337	0.00782701	SLC22A17
6405	A_37_P110326	0.00784278	FRRS1
6406	A_37_P142584	0.00785938	ANKLE1
6407	A_37_P099059	0.00786715	ZNF618
6408	A_37_P152089	0.00788743	STK11
6409	A_37_P302770	0.00788874	CA4
6410	A_37_P230975	0.0078941	RNF180
6411	A_37_P202504	0.00790551	GAL3ST3
6412	A_37_P250108	0.00791948	YIPF7
6413	A_37_P267870	0.00796984	LHCGR
6414	A_37_P006657	0.00797534	C5orf4
6415	A_37_P172672	0.00797607	VPS13B
6416	A_37_P393268	0.00801024	WRN
6417	A_37_P003576	0.00802045	ATP10B
6418	A_37_P239060	0.00802611	DGKG
6419	A_37_P022205	0.00807261	ZPLD1
6420	A_37_P185228	0.00807906	SEC14L2
6421	A_37_P154195	0.00811308	ZNF772
6422	A_37_P039428	0.00814284	TMCO7
6423	A_37_P095989	0.00827708	ODF2
6424	A_37_P355173	0.00828218	ABCC10
6425	A_37_P162275	0.00828218	C7orf43
6426	A_37_P350385	0.00829899	RPL5
6427	A_37_P127942	0.0083118	NCF2
6428	A_37_P216777	0.00832917	CCNA1
6429	A_37_P069591	0.00834436	ACE2
6430	A_37_P079736	0.00836973	CNTNAP1
6431	A_37_P071863	0.00838697	ARSE
6432	A_37_P330299	0.00842143	OTUB2
6433	A_37_P409963	0.00842596	ZNRF3
6434	A_37_P323726	0.00844545	DTNA
6435	A_37_P101778	0.00848009	SERPINB7
6436	A_37_P109311	0.00848675	DCAF8
6437	A_37_P371423	0.00849732	ZNF208
6438	A_37_P068342	0.00852956	KIAA0748
6439	A_37_P282762	0.00853824	CCDC64

3050	A_37_P316844	0.00011719	TLE1
3051	A_37_P319124	0.00011723	MRRF
3052	A_37_P146988	0.00011728	KISS1R
3053	A_37_P233467	0.00011743	ACPL2
3054	A_37_P084589	0.00011765	LRRC37B
3055	A_37_P077598	0.00011771	SLC43A2
3056	A_37_P033413	0.00011773	DNASE1
3057	A_37_P040787	0.0001181	ACP1
3058	A_24_P76018	0.00011817	C3orf71
3059	A_24_P109214	0.00011824	APOC1
3060	A_37_P164663	0.00011842	UBE2D4
3061	A_37_P114024	0.00011854	CRTC2
3062	A_37_P233369	0.00011857	RARRES1
3063	A_37_P310766	0.00011862	ARHGAP44
3064	A_37_P061962	0.00011872	ITFG2
3065	A_33_P3410449	0.00011878	SCARF2
3066	A_37_P102967	0.0001188	ENST00000327471
3067	A_23_P77669	0.00011897	ZNF821
3068	A_37_P062493	0.00011905	AVIL
3069	A_37_P371927	0.00011949	UQCR11
3070	A_37_P019473	0.00011961	RPL14
3071	A_23_P324538	0.00011962	LCE3B
3072	A_37_P054913	0.00011962	STK36
3073	A_37_P084757	0.00011962	MAPT
3074	A_37_P190637	0.00011979	VSX1
3075	A_37_P214330	0.00011989	COL6A2
3076	A_37_P320084	0.00012001	PKN3
3077	A_37_P365151	0.0001205	TRIM39-RPP21
3078	A_37_P102085	0.00012099	FBXO15
3079	A_37_P126424	0.00012108	CACNA1S
3080	A_37_P440536	0.00012123	CDK8
3081	A_37_P271013	0.00012124	KLHL30
3082	A_37_P253721	0.00012155	MANBA
3083	A_37_P439532	0.00012161	LSS
3084	A_37_P165141	0.00012161	TAF6
3085	A_37_P185810	0.00012176	TOM1
3086	A_33_P3397785	0.00012183	SYT2
3087	A_33_P3221303	0.00012183	CCR10
3088	A_37_P195943	0.00012185	KIAA1217
3089	A_37_P322098	0.00012192	TRAF2
3090	A_37_P282001	0.00012194	C12orf34
3091	A_37_P120638	0.00012194	PLXNA2
3092	A_37_P145112	0.00012209	MIER2
3093	A_37_P027815	0.0001222	ODZ3
3094	A_23_P87742	0.00012221	IFFO1
3095	A_37_P370758	0.00012244	PLEKHA4
3096	A_37_P020054	0.00012244	SIDT1
3097	A_37_P178844	0.00012256	LOC100505503
3098	A_37_P091830	0.00012281	COL5A1
3099	A_37_P329709	0.00012296	C14orf2
3100	A_37_P310616	0.00012298	RARA
3101	A_37_P136866	0.000123	HDDC2
3102	A_24_P74160	0.00012312	SNRPD2
3103	A_33_P3279276	0.00012317	GRIP2
3104	A_37_P274975	0.00012317	C2orf3
3105	A_37_P098043	0.00012325	VAV2
3106	A_37_P328711	0.00012326	KIAA1409
3107	A_37_P203567	0.00012342	DDB2
3108	A_23_P79108	0.0001236	ATP8B3
3109	A_37_P055491	0.00012373	TMEM182
3110	A_37_P080205	0.00012413	SPECC1
6440	A_37_P191785	0.00856289	ACSL5
6441	A_37_P148506	0.00856888	MAG
6442	A_37_P433237	0.00859403	PLAC1L
6443	A_37_P404414	0.00860666	TMOD2
6444	A_23_P69970	0.00861433	SOX30
6445	A_37_P096900	0.00862193	C9orf46
6446	A_37_P143063	0.00864522	AZU1
6447	A_23_P425681	0.0086524	CCK
6448	A_37_P197697	0.00874286	HPSE2
6449	A_37_P001390	0.00875267	CENPH
6450	A_37_P184873	0.00878421	IL17REL
6451	A_37_P206941	0.0088219	SCN4B
6452	A_37_P309001	0.00885121	MYH3
6453	A_37_P091799	0.00888698	COL27A1
6454	A_23_P7144	0.00889001	CXCL1
6455	A_33_P3398667	0.00889868	ZSCAN30
6456	A_37_P246578	0.00890164	SULT1E1
6457	A_23_P167005	0.00890626	GPR160
6458	A_37_P181739	0.00891535	GAB4
6459	A_37_P093951	0.00896259	KCNV2
6460	A_37_P110607	0.00899217	MCOLN3
6461	A_37_P341328	0.00900165	IL20
6462	A_33_P3332474	0.00905036	GOLGA6L1
6463	A_37_P096399	0.00905964	C5
6464	A_37_P127717	0.00911114	VTCN1
6465	A_37_P160388	0.00912032	HGF
6466	A_37_P284287	0.00914136	NRIP2
6467	A_37_P142257	0.00914686	ZSCAN16
6468	A_37_P053105	0.00916478	LRP1B
6469	A_23_P91764	0.00919008	TNFRSF13C
6470	A_24_P223874	0.00924695	SFTPA1
6471	A_37_P127843	0.00927017	CASQ2
6472	A_37_P368014	0.00935829	CNOT3
6473	A_37_P396841	0.00935898	CHRNA3
6474	A_37_P158169	0.00938035	GNGT1
6475	A_33_P3247473	0.0093888	KRTAP23-1
6476	A_37_P366348	0.00939503	KLK4
6477	A_37_P379177	0.00942102	ACTL6B
6478	A_37_P163497	0.00944738	SNX10
6479	A_37_P001692	0.00945726	PRLR
6480	A_37_P144704	0.00946844	LGALS4
6481	A_37_P366653	0.00947574	ATCAY
6482	A_37_P375402	0.00948272	RSPH6A
6483	A_37_P085792	0.00949562	P2RX1
6484	A_37_P050006	0.00951248	MFSD6
6485	A_37_P439038	0.00953819	C21orf58
6486	A_37_P212971	0.00953844	ZNF408
6487	A_37_P181383	0.00957122	ACR
6488	A_37_P032816	0.0095892	CLUAP1
6489	A_33_P3325547	0.00959858	C10orf53
6490	A_37_P129850	0.00960956	FCAMR
6491	A_23_P27107	0.00963522	TM4SF5
6492	A_37_P014105	0.00965197	GRAMD1C
6493	A_37_P101757	0.00966889	SERPINB13
6494	A_37_P145110	0.00971469	ANO8
6495	A_23_P336506	0.00975826	TAS2R9
6496	A_23_P436138	0.0097606	MAX
6497	A_37_P153384	0.00980131	ZNF101
6498	A_37_P112319	0.0098197	AGT
6499	A_37_P362503	0.0098327	PGBD1
6500	A_37_P300062	0.00993934	TKTL1

3111	A_37_P084819	0.00012418	DVL2
3112	A_37_P153891	0.00012448	ZNF627
3113	A_37_P015521	0.0001245	ZFYVE20
3114	A_33_P3303772	0.0001245	SLC6A3
3115	A_37_P009342	0.00012451	FAM193B
3116	A_37_P087029	0.00012454	RHBDL3
3117	A_37_P033003	0.00012468	COX4I1
3118	A_37_P021172	0.00012471	KIAA0226
3119	A_37_P253228	0.00012502	STBD1
3120	A_37_P259587	0.0001255	SPATA2L
3121	A_37_P147593	0.00012562	DMPK
3122	A_37_P190426	0.0001257	RRBP1
3123	A_37_P195777	0.0001258	ZNF32
3124	A_37_P118534	0.00012617	PDE4DIP
3125	A_37_P185053	0.00012628	TPST2
3126	A_37_P206256	0.00012644	LAYN
3127	A_37_P158974	0.00012657	DGKB
3128	A_37_P053614	0.00012683	PCGF1
3129	A_37_P022072	0.00012716	CHCHD4
3130	A_37_P118441	0.00012737	KLHDC9
3131	A_23_P100392	0.00012755	TCEB2
3132	A_37_P015278	0.00012805	KLHL18
3133	A_37_P057687	0.00012807	CLEC1A
3134	A_37_P124188	0.00012832	PIAS3
3135	A_37_P092672	0.00012832	UBAP2
3136	A_37_P040570	0.00012832	KIF3C
3137	A_33_P3417452	0.00012851	ZGLP1
3138	A_37_P367063	0.00012852	C19orf44
3139	A_37_P123375	0.00012852	OAZ3
3140	A_37_P208520	0.00012852	NAV2
3141	A_37_P145164	0.00012865	LIPE
3142	A_37_P080031	0.00012885	CPSF4L
3143	A_37_P081982	0.00012932	DCAKD
3144	A_32_P8551	0.00012935	NRN1L
3145	A_37_P190741	0.00012937	JPH2
3146	A_37_P144800	0.00012942	DHX34
3147	A_37_P075467	0.0001295	F8
3148	A_37_P038241	0.00012966	RPL13
3149	A_37_P209926	0.00013014	PATL1
3150	A_37_P148482	0.00013022	REXO1
3151	A_37_P084217	0.00013023	BZRAP1
3152	A_37_P067487	0.0001303	TMEM116
3153	A_37_P189457	0.00013035	C20orf112
3154	A_37_P235209	0.00013039	COL7A1
3155	A_23_P35796	0.0001309	PPP2R5B
3156	A_33_P3306264	0.00013112	LYPD3
3157	A_37_P253927	0.00013113	UCHL1
3158	A_23_P79794	0.00013145	TGIF2
3159	A_33_P3588134	0.00013211	PANX2
3160	A_37_P066043	0.00013262	PXMP2
3161	A_37_P151177	0.00013306	CADM4
3162	A_23_P68740	0.00013339	AIRE
3163	A_37_P258541	0.00013346	ITGAL
3164	A_37_P304402	0.00013354	DRG2
3165	A_37_P256346	0.00013354	CETP
3166	A_37_P020403	0.0001342	WNT7A
3167	A_37_P331537	0.00013427	LTBP2
3168	A_23_P94647	0.00013427	OR1L3
3169	A_37_P053481	0.00013441	SAG
3170	A_37_P003069	0.00013453	GPX3
3171	A_37_P184403	0.00013456	MYO18B

6501	A_37_P173224	0.00995044	CILP
6502	A_37_P109930	0.01000368	AQP10
6503	A_37_P428753	0.01000844	GLB1L3
6504	A_37_P010878	0.01004105	SUMF1
6505	A_37_P232775	0.0100591	FGF1
6506	A_37_P001941	0.0100603	MZB1
6507	A_37_P092256	0.01008312	SVEP1
6508	A_23_P114307	0.01014334	P2RY4
6509	A_37_P099308	0.01016585	ASXL3
6510	A_37_P337443	0.01016979	SUSD4
6511	A_23_P207911	0.01021714	TRPV2
6512	A_37_P146868	0.01023018	MAU2
6513	A_37_P359428	0.0102383	GSTA5
6514	A_37_P162703	0.01024472	RNF32
6515	A_33_P3307253	0.0102653	AK5
6516	A_33_P3407529	0.0102696	PRRT4
6517	A_37_P089308	0.01035919	SLC39A11
6518	A_37_P103731	0.01041252	ALKBH1
6519	A_23_P46755	0.01045184	GDF2
6520	A_37_P009617	0.01045589	NCEH1
6521	A_37_P260505	0.0104665	MYLPF
6522	A_37_P167997	0.01051281	FZD3
6523	A_37_P095774	0.01055198	NPR2
6524	A_37_P123675	0.01055678	PADI4
6525	A_37_P143775	0.01056564	CCDC61
6526	A_37_P029370	0.01062659	FAM53A
6527	A_37_P165219	0.01064561	ZNF282
6528	A_37_P409541	0.01066808	PDGFB
6529	A_37_P142613	0.01082022	KLK12
6530	A_37_P046156	0.01090088	GYPC
6531	A_37_P076058	0.01093277	SUV39H1
6532	A_37_P036533	0.01101747	MGRN1
6533	A_37_P186791	0.01104965	C20orf166
6534	A_37_P214052	0.0110563	TMPRSS3
6535	A_37_P080263	0.01106808	A_37_P080263
6536	A_33_P3410650	0.0110689	SPAG8
6537	A_37_P173125	0.01107151	CHRFAM7A
6538	A_37_P145863	0.01107432	SLC7A9
6539	A_37_P025367	0.01113678	RCHY1
6540	A_37_P074361	0.01115125	NLGN3
6541	A_37_P106459	0.01116799	NFATC4
6542	A_37_P177637	0.01126447	LRRC49
6543	A_37_P109445	0.01128677	AGRN
6544	A_37_P075526	0.01128697	SASH3
6545	A_37_P345903	0.01129799	MTX1
6546	A_37_P247928	0.01131578	PARM1
6547	A_37_P327387	0.01134409	COCH
6548	A_37_P099221	0.01140246	ANKRD30B
6549	A_37_P301717	0.01147568	ARHGEF15
6550	A_37_P261728	0.01150449	TPSG1
6551	A_37_P060341	0.01150736	GOLGA2B
6552	A_37_P307488	0.01151349	RPL23
6553	A_37_P184785	0.0115192	C22orf42
6554	A_37_P022467	0.01159064	ALB
6555	A_37_P140663	0.01160214	SOBP
6556	A_37_P200830	0.01170813	ZNF485
6557	A_37_P114212	0.01171653	GLT25D2
6558	A_37_P187644	0.01174306	DEFB123
6559	A_37_P210870	0.01179889	SLC22A10
6560	A_37_P029992	0.0118108	PDGFC
6561	A_33_P3226099	0.01185113	OTOP2

3172	A_37_P060357	0.00013466	ANO2
3173	A_37_P102066	0.00013498	TNFRSF11A
3174	A_37_P312691	0.00013505	TMEM97
3175	A_37_P109640	0.00013523	FMO5
3176	A_37_P003080	0.00013525	GRAMD3
3177	A_33_P3414799	0.00013543	SH3BP2
3178	A_37_P434863	0.00013549	SLC6A5
3179	A_37_P327394	0.00013563	COX16
3180	A_37_P369403	0.00013622	FXYD5
3181	A_37_P260528	0.0001364	PRSS53
3182	A_37_P215196	0.00013662	POFUT2
3183	A_37_P327534	0.00013676	CYP46A1
3184	A_37_P094447	0.000137	NDOR1
3185	A_37_P317107	0.00013713	HABP4
3186	A_37_P075375	0.00013721	CACNA1F
3187	A_37_P403146	0.00013751	STRA6
3188	A_37_P339967	0.00013757	RPE65
3189	A_37_P256704	0.0001376	CORO1A
3190	A_37_P184225	0.00013767	MIOX
3191	A_37_P275038	0.00013784	C2orf54
3192	A_37_P128662	0.00013818	LYSMD1
3193	A_37_P146667	0.00013823	ATP1A3
3194	A_37_P103296	0.0001385	TRIM9
3195	A_33_P3369567	0.00013852	LSP1
3196	A_37_P202604	0.00013856	CAPN5
3197	A_23_P106806	0.00013902	PRSS27
3198	A_37_P030780	0.00013925	NHEDC2
3199	A_23_P47058	0.00013934	CUZD1
3200	A_37_P187867	0.00013942	CST2
3201	A_37_P139848	0.00013991	RIMS1
3202	A_37_P205101	0.00014037	SLC22A6
3203	A_37_P056171	0.00014051	KIAA1310
3204	A_23_P4798	0.00014064	ZNF581
3205	A_37_P100541	0.0001407	ZFP161
3206	A_37_P386904	0.00014075	FAM131B
3207	A_23_P48109	0.00014113	NINJ2
3208	A_37_P305418	0.00014148	GCGR
3209	A_37_P071560	0.00014149	ARHGAP4
3210	A_37_P046045	0.00014163	GRB14
3211	A_37_P169227	0.00014182	DLC1
3212	A_37_P171077	0.00014187	C8orf42
3213	A_37_P285666	0.00014192	ISCU
3214	A_37_P014423	0.00014233	NUP210
3215	A_37_P163781	0.00014239	STAG3
3216	A_37_P079467	0.00014249	NOTUM
3217	A_37_P117634	0.00014262	IL24
3218	A_37_P081018	0.00014291	EVPLL
3219	A_37_P050871	0.00014341	TBC1D8
3220	A_37_P395140	0.00014363	NAPRT1
3221	A_23_P1981	0.00014377	INS
3222	A_37_P308407	0.00014386	MAP2K6
3223	A_37_P112482	0.00014399	GBP2
3224	A_37_P107197	0.00014421	POMT2
3225	A_37_P272544	0.00014441	DNMT3A
3226	A_37_P044600	0.00014444	DYSF
3227	A_37_P363109	0.00014457	C6orf192
3228	A_37_P175950	0.00014462	PLA2G4D
3229	A_37_P036975	0.00014486	NECAB2
3230	A_37_P174986	0.00014501	ANPEP
3231	A_23_P302568	0.00014505	SLC30A3
3232	A_37_P368114	0.00014509	CPT1C
6562	A_37_P009026	0.01186734	UNC5A
6563	A_23_P126031	0.01187975	OR10J5
6564	A_23_P147025	0.01203645	RAB33A
6565	A_37_P337600	0.01216761	DCDC2B
6566	A_33_P3283237	0.01217295	YY2
6567	A_37_P125418	0.01223293	PTBP2
6568	A_37_P117011	0.01228712	ASTN1
6569	A_37_P130612	0.01229565	WNT3A
6570	A_37_P154746	0.01231163	AHCYL2
6571	A_37_P075498	0.01243321	SAGE1
6572	A_37_P412417	0.01244769	ZBP1
6573	A_37_P136902	0.01266883	DLL1
6574	A_23_P51039	0.01269087	INHA
6575	A_37_P163324	0.0127778	SLC29A4
6576	A_37_P098996	0.01280394	TRAF1
6577	A_37_P409398	0.01282305	TBX1
6578	A_37_P235523	0.01286924	CLDN18
6579	A_37_P038546	0.01294976	CASKIN1
6580	A_37_P351111	0.01304876	ITLN2
6581	A_23_P121282	0.01305133	TMEM89
6582	A_37_P335705	0.01311495	CASQ1
6583	A_37_P061705	0.01312586	HOXC4
6584	A_37_P375546	0.01312586	CCDC114
6585	A_37_P373325	0.01313999	ZNF541
6586	A_37_P151628	0.0132481	VSTM1
6587	A_37_P043395	0.0132588	NPHP1
6588	A_37_P147432	0.01338427	SHISA7
6589	A_37_P213205	0.01340572	EIF1AY
6590	A_23_P346384	0.01346952	MRPL43
6591	A_37_P050049	0.01355922	MLPH
6592	A_37_P104626	0.01367337	ASB2
6593	A_37_P104779	0.01374968	IL25
6594	A_37_P393225	0.01377302	OPRK1
6595	A_23_P44569	0.01378105	ABCC2
6596	A_37_P327953	0.01382385	CTSG
6597	A_24_P7600	0.01392472	FBXL7
6598	A_37_P193156	0.0139439	CCDC147
6599	A_37_P002214	0.01398495	EGFLAM
6600	A_37_P205970	0.01400331	KCNC1
6601	A_37_P285648	0.01425108	IRAK4
6602	A_37_P027801	0.01425673	ODAM
6603	A_37_P132649	0.01439655	GABBR1
6604	A_33_P3317928	0.01440236	KRTAP20-3
6605	A_37_P091978	0.01441397	FAM75C2
6606	A_37_P366319	0.01449811	KLK11
6607	A_37_P155690	0.01458123	C7orf51
6608	A_37_P003225	0.01458772	SCGB3A1
6609	A_37_P183479	0.01461085	DGCR2
6610	A_37_P311602	0.01472051	FAM20A
6611	A_37_P023444	0.01476556	C4orf44
6612	A_37_P108409	0.01481653	TSHR
6613	A_37_P351162	0.01482325	SGIP1
6614	A_37_P074182	0.0149794	ZDHHC15
6615	A_37_P208062	0.01504013	MOGAT2
6616	A_37_P061564	0.01513085	HELB
6617	A_37_P114963	0.01515071	ELF3
6618	A_37_P276755	0.01542166	TTC21B
6619	A_37_P107566	0.01545447	SERPINA5
6620	A_37_P036316	0.01553108	PMFBP1
6621	A_37_P103932	0.01554504	DHRS4L2
6622	A_37_P179326	0.01555116	EXD1

3233	A_37_P395397	0.00014517	SLC25A37
3234	A_37_P208623	0.00014521	SCUBE2
3235	A_37_P374877	0.00014522	RPS5
3236	A_37_P121834	0.00014531	HSPB7
3237	A_37_P201042	0.00014533	RNF141
3238	A_37_P200732	0.00014535	ZFYVE27
3239	A_37_P165212	0.0001455	ZNF277
3240	A_23_P122724	0.00014558	VNN2
3241	A_37_P077305	0.00014564	AATK
3242	A_37_P180206	0.00014564	SPTBN5
3243	A_37_P376299	0.00014641	SIGLEC6
3244	A_37_P176331	0.00014666	PLCB2
3245	A_37_P017693	0.00014675	NFKBIZ
3246	A_37_P169051	0.00014685	ZFAND1
3247	A_37_P093527	0.00014709	ASTN2
3248	A_33_P3311740	0.00014735	ZNF774
3249	A_37_P208827	0.00014756	NTM
3250	A_37_P165863	0.00014758	ASH2L
3251	A_37_P107678	0.00014783	SLC24A4
3252	A_37_P078055	0.00014805	ACACA
3253	A_37_P297295	0.00014807	RENBP
3254	A_37_P082358	0.00014888	RDM1
3255	A_37_P187083	0.00014907	CDC25B
3256	A_37_P204162	0.0001493	EIF3F
3257	A_37_P030601	0.0001494	MAPKSP1
3258	A_37_P074238	0.00014957	EFHC2
3259	A_37_P154226	0.00014977	ZNF792
3260	A_37_P124371	0.00014982	PAQR6
3261	A_37_P216563	0.00015028	C13orf16
3262	A_37_P089101	0.00015068	TP53I13
3263	A_23_P33326	0.00015068	ADRA1B
3264	A_23_P133902	0.00015084	PSORS1C1
3265	A_33_P3228190	0.00015107	KCNAB2
3266	A_37_P016093	0.00015133	RPL22L1
3267	A_37_P344157	0.00015152	RERE
3268	A_37_P231193	0.00015167	SERF1A
3269	A_37_P248880	0.00015182	GLRB
3270	A_37_P370511	0.00015226	KCNN1
3271	A_37_P019238	0.00015229	IQSEC1
3272	A_37_P123208	0.00015238	NR5A2
3273	A_37_P441428	0.0001525	GRTP1
3274	A_37_P219119	0.00015273	PHF11
3275	A_37_P144720	0.0001528	SLC6A16
3276	A_37_P127859	0.00015292	CASZ1
3277	A_37_P266006	0.00015294	ERLEC1
3278	A_33_P3235856	0.00015303	RORB
3279	A_37_P045664	0.00015316	GCKR
3280	A_37_P336852	0.00015319	AKT3
3281	A_37_P432913	0.00015346	EML3
3282	A_37_P082000	0.00015368	GUCY2D
3283	A_37_P331819	0.00015371	SYNE2
3284	A_37_P170587	0.00015382	TMEM55A
3285	A_37_P355559	0.00015437	TUBB2A
3286	A_37_P154633	0.00015454	ADCY1
3287	A_37_P115644	0.00015459	FDPS
3288	A_37_P211178	0.00015519	SMPD1
3289	A_37_P408814	0.00015553	TXNRD2
3290	A_37_P012186	0.00015554	CPNE9
3291	A_37_P261875	0.0001557	RNF151
3292	A_37_P053927	0.00015598	FAM150B
3293	A_37_P438744	0.00015605	TFF2
6623	A_33_P3214948	0.01561003	SPOCK2
6624	(+)E1A_r60_n11	0.01566258	E1A_r60_n11
6625	A_37_P084797	0.01571404	MYH1
6626	A_37_P257630	0.01573816	PRSS33
6627	A_37_P069092	0.01574589	SRPX
6628	A_33_P3226080	0.01580431	C17orf87
6629	A_37_P172383	0.01586579	FBXO16
6630	A_37_P006036	0.01595069	ELOVL7
6631	A_37_P303049	0.01603154	USH1G
6632	A_23_P345692	0.01608813	IL17D
6633	A_37_P130026	0.01613337	FCRL1
6634	A_37_P151612	0.01613719	RAB3A
6635	A_37_P098849	0.01628814	WDR38
6636	A_37_P419052	0.01652727	GRID1
6637	A_37_P423396	0.0166522	SLC29A3
6638	A_37_P134633	0.01677744	CUL7
6639	A_37_P062387	0.01679837	SYCP3
6640	A_37_P217325	0.01681594	F10
6641	A_37_P010874	0.01685095	PRICKLE2
6642	A_33_P3241081	0.01686578	AKNAD1
6643	A_37_P146920	0.01690844	ZNF296
6644	A_37_P018640	0.01695971	IL5RA
6645	A_37_P379325	0.01696736	C7orf10
6646	A_24_P93896	0.01704376	CNNM2
6647	A_37_P304191	0.01717309	ENST00000360606
6648	A_37_P115469	0.01719652	SLC1A7
6649	A_37_P141315	0.01722101	TNF
6650	A_24_P80776	0.01724407	LOC221710
6651	A_37_P398657	0.01724716	PPIP5K1
6652	A_37_P141752	0.01739986	UNC93A
6653	A_37_P043857	0.01747544	CYP20A1
6654	A_37_P018525	0.01749979	C3orf64
6655	A_37_P137491	0.01751357	MLLT4
6656	A_37_P014618	0.01756159	IL20RB
6657	A_23_P55281	0.01759173	HOXB7
6658	A_37_P320496	0.01761058	IPPK
6659	A_37_P158247	0.01775135	GRB10
6660	A_37_P091867	0.01775615	LCN15
6661	A_37_P090442	0.01783042	A_37_P090442
6662	A_37_P339933	0.01792473	GBP6
6663	A_37_P209166	0.01807539	PANX1
6664	A_37_P004504	0.01811384	SPEF2
6665	A_37_P133116	0.0181694	CAPN11
6666	A_37_P396720	0.01825326	ZNF707
6667	A_23_P363778	0.01839361	FRZB
6668	A_24_P272310	0.01845986	MUSTN1
6669	A_37_P255251	0.01853397	ARHGDIG
6670	A_37_P062530	0.01875371	LAG3
6671	A_37_P387736	0.01882137	CCL24
6672	A_23_P168592	0.01882383	CCDC126
6673	A_37_P199631	0.01884342	SEC31B
6674	A_37_P015435	0.01885845	LEKR1
6675	A_37_P219184	0.01886238	EPSTI1
6676	A_33_P3261973	0.01921282	AP4B1
6677	A_37_P138908	0.01933082	IL20RA
6678	A_37_P303251	0.01940806	AIPL1
6679	A_37_P112578	0.01947003	GBP7
6680	A_37_P127662	0.0194824	RGS8
6681	A_37_P089704	0.0195608	XYLT2
6682	A_37_P148657	0.01966808	MED25
6683	A_23_P94296	0.01972264	ADAM7

3294	A_37_P030544	0.00015642	WHSC1
3295	A_37_P293122	0.0001568	ARL13A
3296	A_37_P047449	0.00015681	REEP1
3297	A_37_P295862	0.00015688	MPP1
3298	A_23_P171270	0.00015694	H2AFB2
3299	A_37_P201450	0.00015731	ABCC8
3300	A_37_P181415	0.00015743	ADRBK2
3301	A_37_P168148	0.00015748	GPIHBP1
3302	A_37_P075417	0.00015767	RPL10
3303	A_37_P018272	0.00015767	PHF7
3304	A_24_P56837	0.00015775	ASB16
3305	A_37_P007783	0.00015786	SLC34A1
3306	A_33_P3359373	0.00015786	ENST00000403810
3307	A_37_P166511	0.00015786	CHMP4C
3308	A_37_P166212	0.00015808	C8orf31
3309	A_37_P086210	0.00015839	IFT20
3310	A_37_P085620	0.00015848	MYO15A
3311	A_37_P370031	0.0001585	PLA2G4C
3312	A_37_P329706	0.00015857	C14orf182
3313	A_37_P100281	0.00015899	ATP5A1
3314	A_37_P061341	0.00015911	NTN4
3315	A_37_P262152	0.00015911	SETD1A
3316	A_37_P108393	0.00015919	TRAF3
3317	A_37_P005767	0.00015922	NKD2
3318	A_37_P179639	0.00015935	SCAMP5
3319	A_37_P403343	0.00015943	CALML4
3320	A_37_P098158	0.00015947	CDK20
3321	A_37_P035848	0.00015956	DOC2A
3322	A_23_P207154	0.00015959	CSH2
3323	A_33_P3341105	0.00015961	PDE6G
3324	A_37_P076795	0.00015988	KDM6A
3325	A_23_P164196	0.00015991	DLX4
3326	A_37_P439169	0.00015997	PCBP3
3327	A_23_P164258	0.00016015	PIPOX
3328	A_37_P238405	0.00016018	IRAK2
3329	A_37_P028205	0.0001602	SLC2A9
3330	A_37_P013915	0.0001603	SCAP
3331	A_37_P112188	0.00016106	PHTF1
3332	A_37_P092353	0.00016111	DNAJB5
3333	A_37_P111362	0.00016185	C1orf54
3334	A_37_P214129	0.00016338	C21orf7
3335	A_37_P008937	0.00016346	PPP2R2B
3336	A_23_P67198	0.00016348	CPAMD8
3337	A_37_P188632	0.00016362	ZFP64
3338	A_37_P061710	0.00016373	HOXC6
3339	A_37_P311319	0.00016375	SGSM2
3340	A_37_P292627	0.00016375	RAPGEF3
3341	A_37_P408780	0.00016375	RGL4
3342	A_37_P363571	0.00016385	RING1
3343	A_37_P029168	0.00016388	FAM175A
3344	A_37_P094029	0.00016422	C9orf174
3345	A_37_P035196	0.00016486	MSLNL
3346	A_37_P370180	0.00016496	ICAM1
3347	A_37_P107710	0.00016532	JAG2
3348	A_37_P242736	0.00016535	ETV5
3349	A_37_P155012	0.00016567	AP4M1
3350	A_37_P420590	0.00016594	OGDHL
3351	A_37_P327894	0.00016604	ESRRB
3352	A_37_P202855	0.00016619	CD5
3353	A_37_P143374	0.00016639	C19orf53
3354	A_37_P085948	0.00016642	NME2

6684	A_37_P180065	0.01998147	RHCG
6685	A_37_P443444	0.0200488	SPATA13
6686	A_37_P171698	0.02012165	SLC26A7
6687	A_23_P144490	0.02015102	DCHS2
6688	A_37_P358469	0.02028669	GPR110
6689	A_37_P377373	0.02031331	PEX11G
6690	A_37_P421136	0.02060944	C10orf92
6691	A_37_P117449	0.02081274	IFI16
6692	A_37_P056694	0.02081531	ZAP70
6693	A_37_P057753	0.02084943	ABCD2
6694	A_37_P081368	0.02089427	C17orf104
6695	A_23_P57036	0.02089684	CD40
6696	A_37_P078453	0.02093378	KRT16
6697	A_37_P352417	0.02103562	TAS1R1
6698	A_37_P136093	0.02109116	ZNF311
6699	A_37_P341245	0.02126525	IGFN1
6700	A_23_P45955	0.02127641	TEKT2
6701	A_33_P3239487	0.02134061	NCRNA00175
6702	A_37_P151599	0.02142967	CARD8
6703	A_37_P071687	0.02145468	ARHGEF6
6704	A_37_P215167	0.02151207	MX1
6705	A_37_P028025	0.0216397	PDGFRA
6706	A_37_P115411	0.02183505	GON4L
6707	A_37_P313605	0.02186656	CHAD
6708	A_37_P069840	0.02223341	GAB3
6709	A_37_P003202	0.02226517	GZMA
6710	A_37_P266053	0.02246361	C2orf39
6711	A_37_P298527	0.02247416	SYP
6712	A_33_P3385762	0.02275589	OR52W1
6713	A_37_P181745	0.02284965	SULT4A1
6714	A_37_P353967	0.02311052	VANGL2
6715	A_37_P034867	0.02312813	ITGAX
6716	A_37_P341668	0.02333555	ENST00000489935
6717	A_37_P354430	0.02361417	ZBTB40
6718	A_37_P190979	0.0238108	TCF15
6719	A_37_P186589	0.02396369	BIRC7
6720	A_37_P406835	0.02410761	MORC2
6721	A_37_P147008	0.02420167	KLHL26
6722	A_33_P3244643	0.02432912	FAM183A
6723	A_37_P048518	0.02432935	BOLL
6724	A_37_P319953	0.02445905	ENTPD2
6725	A_37_P135661	0.02446999	GSTA1
6726	A_37_P000753	0.02464947	ACSL6
6727	A_37_P026474	0.02470953	DKK2
6728	A_37_P418888	0.02478091	CDNF
6729	A_37_P162115	0.02483653	PRKRIP1
6730	A_33_P3375358	0.02519419	GPR31
6731	A_37_P025503	0.02543992	HERC6
6732	A_37_P042094	0.02544934	COBLL1
6733	A_37_P142808	0.0259105	APOE
6734	A_23_P56146	0.0271691	RAX2
6735	A_37_P054941	0.02810218	KCNH7
6736	A_37_P031427	0.02814702	A_37_P031427
6737	A_37_P183223	0.02820023	GTPBP1
6738	A_37_P062508	0.02835668	KRT86
6739	A_37_P359154	0.02865756	GRM4
6740	A_37_P292348	0.02902325	WDR66
6741	A_33_P3314301	0.02911435	SV2C
6742	A_37_P247292	0.02933352	ADH4
6743	A_37_P335389	0.02973709	C8A
6744	A_37_P211870	0.02993318	UCP3

3355	A_33_P3252414	0.00016642	TH
3356	A_37_P048087	0.00016656	DLX2
3357	A_23_P1819	0.00016695	OR8B8
3358	A_37_P203279	0.00016713	CREB3L1
3359	A_37_P364609	0.00016726	SYNGAP1
3360	A_37_P093797	0.00016735	PLAA
3361	A_37_P069894	0.0001674	GABRE
3362	A_37_P427953	0.00016755	UNC93B1
3363	A_37_P372399	0.00016769	MEGF8
3364	A_37_P021442	0.00016771	THPO
3365	A_37_P348072	0.00016785	PKP1
3366	A_37_P214994	0.00016808	TFF1
3367	A_37_P132819	0.00016823	ATAT1
3368	A_37_P430617	0.00016828	SLC25A22
3369	A_37_P205379	0.0001685	PLA2G16
3370	A_23_P64611	0.00016857	P2RY6
3371	A_37_P181871	0.00016866	CACNA1I
3372	A_37_P191381	0.0001687	MAVS
3373	A_37_P157010	0.00016893	DNAH11
3374	A_37_P068750	0.00016938	XRCC6BP1
3375	A_37_P339502	0.00016955	CYB5RL
3376	A_37_P183021	0.00016976	GGA1
3377	A_37_P294821	0.00016981	CTAG2
3378	A_37_P352117	0.00017013	CCDC17
3379	A_37_P083769	0.00017017	SCN4A
3380	A_37_P350961	0.00017026	SEMA4A
3381	A_37_P106605	0.00017096	OXA1L
3382	A_37_P019693	0.00017107	ULK4
3383	A_37_P040958	0.0001711	AGAP1
3384	A_37_P109810	0.0001714	SLC45A3
3385	A_37_P138599	0.0001716	ELOVL5
3386	A_37_P174381	0.00017164	LARP6
3387	A_37_P209511	0.00017175	PKNOX2
3388	A_37_P187686	0.00017185	DLGAP4
3389	A_37_P427056	0.00017192	C1QTNF5
3390	A_37_P098286	0.00017233	CD72

6745	A_37_P059559	0.03001441	COQ10A
6746	A_37_P298747	0.03054044	PPP1R3F
6747	A_37_P428883	0.03145571	GRIK4
6748	A_37_P193026	0.03187528	CHST15
6749	A_37_P034439	0.03211895	HBA1
6750	A_23_P211643	0.03272027	PPARA
6751	A_37_P403815	0.03293346	CT62
6752	A_37_P190487	0.03319628	UCKL1
6753	A_37_P389396	0.0332454	ADAMDEC1
6754	A_37_P256342	0.03407829	CES4A
6755	A_33_P3315284	0.03424187	KRT74
6756	A_37_P142931	0.034633	PRDX2
6757	A_37_P402206	0.03470577	NEIL1
6758	A_37_P107042	0.0348855	PRKCH
6759	A_37_P339461	0.03521857	NT5C1A
6760	A_37_P072815	0.03528828	ZC4H2
6761	A_37_P057588	0.03531226	FOXN4
6762	A_37_P019536	0.035498	RPP14
6763	A_37_P262120	0.03597045	SEC14L5
6764	A_37_P052016	0.03639885	CXCR1
6765	A_37_P421967	0.03710999	NRG3
6766	A_37_P061520	0.03763034	GRIP1
6767	A_37_P134688	0.03765358	SLC22A16
6768	A_37_P440290	0.0379108	UGGT2
6769	A_37_P022888	0.03830469	ATP10D
6770	A_37_P112874	0.03860981	CHI3L2
6771	A_37_P121240	0.04004242	LY9
6772	A_37_P063985	0.04030933	LRRK2
6773	A_37_P078606	0.04065854	KRT26
6774	A_37_P314313	0.04095553	ENST00000424271
6775	A_37_P134860	0.04130404	GPR116
6776	A_37_P121670	0.04170269	MFSD2A
6777	A_37_P302495	0.04303248	C17orf50
6778	A_33_P3416882	0.04445742	ARL9
6779	A_37_P031039	0.0453895	ACSM3
6780	A_23_P201022	0.04549424	PKLR
6781	A_37_P034497	0.0465805	PLA2G10
6782	A_23_P50276	0.04728569	ANGPTL6
6783	A_37_P179713	0.04901168	TTC23
