# Supplementary material for: LARP1 post-transcriptionally regulates mTOR and contributes to cancer progression
Source: Oncogene. 2014 Dec 22;34(39):5025–36. doi: 10.1038/onc.2014.428 (PMC4430325; doi:10.1038/onc.2014.428)
Supplement: Supplementary Table 3 [file onc2014428x13.pdf]

Term	KEGG Pathway	Count	Fold Enrichment	p-Value	Benjamini-Hochberg Corrected
hsa04512	ECM-receptor interaction	40	2.277792008	1.50E-08	2.86E-06
hsa04510	Focal adhesion	68	1.65215847	2.73E-06	2.60E-04
hsa04360	Axon guidance	38	1.822233607	5.35E-05	0.003380312
hsa05412	Arrhythmogenic right ventricular cardiomyopathy (ARVC)	28	2.040901639	5.74E-05	0.002723909
hsa04142	Lysosome	47	1.679313324	7.77E-05	0.002948841
hsa04070	Phosphatidylinositol signaling system	29	1.677611892	0.002537246	0.077297314
hsa04810	Regulation of actin cytoskeleton	56	1.35159049	0.008408672	0.204830814
hsa04010	MAPK signaling pathway	77	1.281388016	0.008750621	0.188395315
hsa05410	Hypertrophic cardiomyopathy (HCM)	26	1.606036399	0.008897199	0.171940545
hsa05414	Dilated cardiomyopathy	28	1.569924338	0.009142807	0.160133501
hsa00230	Purine metabolism	35	1.386482092	0.02733238	0.380397195
hsa04520	Adherens junction	21	1.56191452	0.028358194	0.365865504
hsa04666	Fc gamma R-mediated phagocytosis	29	1.428237151	0.031434543	0.372996949
hsa04370	VEGF signaling pathway	23	1.496834748	0.034807373	0.381713251
hsa04062	Chemokine signaling pathway	31	1.394796094	0.0356724	0.368783665
hsa05200	Pathways in cancer	74	1.20937477	0.040537022	0.38823429
hsa00561	Glycerolipid metabolism	16	1.619763206	0.044842553	0.401163252
hsa00520	Amino sugar and nucleotide sugar metabolism	16	1.619763206	0.044842553	0.401163252
hsa04610	Complement and coagulation cascades	18	1.56191452	0.044962313	0.38467384
hsa05222	Small cell lung cancer	26	1.414270859	0.047896502	0.387872899
hsa04662	B cell receptor signaling pathway	23	1.445219757	0.051353055	0.393970428
hsa00430	Taurine and hypotaurine metabolism	5	3.037056011	0.051633359	0.381000848
hsa00052	Galactose metabolism	11	1.822233607	0.052749841	0.373760472
hsa04150	mTOR signaling pathway	17	1.548898566	0.056589686	0.381976314
hsa00051	Fructose and mannose metabolism	14	1.645888419	0.05677984	0.370464573
hsa04020	Calcium signaling pathway	45	1.261546343	0.059650275	0.373386922
hsa05217	Basal cell carcinoma	15	1.56191452	0.071947924	0.420533031
hsa00310	Lysine degradation	13	1.579269126	0.092032973	0.493082153
hsa00563	Glycosylphosphatidylinositol(GPI)-anchor biosynthesis	11	1.670380806	0.093138901	0.484909173
hsa00562	Inositol phosphate metabolism	19	1.413160756	0.097039391	0.487664749
hsa04640	Hematopoietic cell lineage	19	1.413160756	0.097039391	0.487664749
