# Supplementary material for: LARP1 post-transcriptionally regulates mTOR and contributes to cancer progression
Source: Oncogene. 2014 Dec 22;34(39):5025–36. doi: 10.1038/onc.2014.428 (PMC4430325; doi:10.1038/onc.2014.428)
Supplement: Supplementary Table 4 [file onc2014428x14.pdf]

**Beyond LARP1 interactome**	**LARP1 interactome**
14.3.3-beta	ACVRL1
14.3.3-epsilon	Bak
53BP1	Bcl-xL
alpha-Catenin	Beclin
AMPK-alpha	Bid
AR	Caveolin
CD31	Claudin-7
CDK1	Collagen-VI
Chk1	C-Raf
c-Kit	Cyclin-E1
c-Met	Dvl3
Cyclin-D1	eIF4E
eEF2K	ERCC1
eIF4G	Fibronectin
GATA3	FOX03a
HER2	FoxM1
HER3	GSK3-alpha-beta
INPP4B	IGFBP2
N.Cadherin	Lck
N-Ras	Notch1
p27	N-Ras
p38-MAPK14	pan-Akt
p53	Rab25
p70S6K	Rad51
PEA15	Raf
PI3K-p85	Rb
PR	SETD2
PTEN	Smad1
Rad50	XRCC1
SF2	
Smad4	
STAT5	
Stathmin-1	
Syk	
VHL	
YAP	
